# Human biomarker navigator

**DOI:** 10.1002/imt2.70157

**Published:** 2026-08-20

**Authors:** Meng‐Yao Li, Qian Zhang, Zijie Wang, Zeping Gui, Jing Liu, Jingda Li, Maojun Jiang, Zukang Chang, Qingqing Han, Bendong Yang, Tariq Saeed, Jun Li, Jinghan Jia, Chenghu Song, Tong Shao, Tianhuan Peng, Xueying Zhang, Pingping Guo, Jun Luo, Gangao Yang, Yuan Wan, Gokhan Zengin, Quan Yuan, Lingru Li, Xiaohui Fan, Yang Luo, Lvyun Zhu, Yanfei Zheng, Wenjun Mao, Wenlong Sun

**Affiliations:** ^1^ Innovation Institute of Integrated Traditional Chinese and Western Medicine, Shandong First Medical University & Shandong Academy of Medical Sciences Jinan China; ^2^ Renji Hospital Affiliated to Shanghai Jiao Tong University School of Medicine Shanghai China; ^3^ College of Pharmacy, Inner Mongolia Medical University Hohhot China; ^4^ Department of Urology Jiangsu Key Laboratory of Urological Disease Prevention and Treatment, The Second Affiliated Hospital of Nanjing Medical University, Nanjing Medical University Nanjing China; ^5^ School of Life Sciences, Yangtze University Jingzhou China; ^6^ School of Life Sciences and Medicine, Shandong University of Technology Zibo China; ^7^ Department of Diet and Nutritional Sciences Ibadat International University Islamabad Pakistan; ^8^ Department of Thoracic Surgery the Affiliated Wuxi People's Hospital of Nanjing Medical University, Wuxi People's Hospital, Wuxi Medical Center, Nanjing Medical University Wuxi China; ^9^ College of Science, National University of Defense Technology Changsha China; ^10^ Molecular Science and Biomedicine Laboratory (MBL), State Key Laboratory of Chemo and Biosensing, College of Biology, Hunan University Changsha China; ^11^ The Pq Laboratory of BiomeDx/Rx, Department of Biomedical Engineering Binghamton University Binghamton New York USA; ^12^ Department of Biology Science Faculty, Selcuk University Konya Turkey; ^13^ College of Chemistry and Molecular Sciences, Wuhan University Wuhan China; ^14^ National Institute of TCM Constitution and Preventive Medicine, Beijing University of Chinese Medicine Beijing China; ^15^ State Key Laboratory of Chinese Medicine Modernization, College of Pharmaceutical Sciences, Zhejiang University Hangzhou China; ^16^ School of Pharmaceutical Sciences, Zhejiang Chinese Medical University Hangzhou China; ^17^ Department of Laboratory Medicine Chongqing Center for Clinical Laboratory, Chongqing Academy of Medical Sciences, Chongqing General Hospital, School of Medicine, Chongqing University Chongqing China

**Keywords:** Biomarker navigator, human health, cross‐system interaction network, cross‐organ interaction network, cross‐disease interaction network, multi‐omics integration, precision medicine

## Abstract

Biomarkers play a pivotal role in contemporary medical research, particularly in the early diagnosis of diseases and personalized treatment. Although previous studies have systematically reviewed markers across various disease domains, an integrated framework that connects major physiological systems, encompasses multiple organs, and spans a broad spectrum of diseases is still lacking. In the context of modern health challenges, marked by the high prevalence of chronic diseases and widespread comorbidities, establishing a panoramic biomarker navigation system is imperative. This review offers the initial comprehensive elucidation of the biomarker interaction networks across diverse systems, organs, and diseases, and delineates the operational framework for establishing a “biomarker navigation system”. The core contribution of this work is a transition from ‘knowledge enumeration’ to a more integrated, systems‐level approach. This provides new perspectives in systems biology for understanding the shared pathological foundations of comorbidities. Furthermore, it provides a theoretical basis for a reorientation in medicine, from a focus on ‘treating existing diseases’ to ‘preventive interventions’ and from ‘single‐disease management’ to a comprehensive, systems‐based approach. To facilitate the exploration and application of this system–organ–disease–biomarker network, we developed the “Human Biomarker Navigator” web platform (http://www.hbiomarker.com), which allows researchers and clinicians to efficiently retrieve the functional characteristics and clinical significance of diverse biomarkers across different disease contexts.

## Highlights

- A comprehensive biomarker atlas encompassing nine human physiological systems was developed for the first time, transitioning from localized to holistic perspectives.
- An innovative framework for a “biomarker navigation system” was introduced, evolving it from a static knowledge base to a dynamic clinical decision support tool.
- The intricate networks of biomarkers across different systems, organs, and diseases, along with their regulatory mechanisms, were systematically detailed.
- A web platform termed “Human Biomarker Navigator” (http://www.hbiomarker.com) was launched to offer tools for streamlined retrieval and thorough analysis of biomarker–disease associations.

## Graphical abstract

The Human Biomarker Navigator integrates the disease continuum, biomarker dynamics, cross‐organ biomarker networks, biomarker classification, and technology‐driven paradigms. It maps how biomarkers link multi‐system physiology and pathology across the nervous, respiratory, endocrine, circulatory, immune, digestive, urinary, reproductive, and musculoskeletal systems. Within the cross‐organ network, representative biomarkers span inflammation and tissue remodeling (SAA, PTX3, MMPs), pulmonary function (FEV1/FVC), angiogenesis and vascular injury (VEGF, PlGF), oncology and minimal residual disease (cfDNA, CTC, MRD, *FLT3‐ITD*, *KRAS*, PD‐L1, CEA, CA125, CA19‐9, GPC1/GPC3), cardiac injury and heart failure (hs‐cTn, CK‐MB, NT‐proBNP, Gal‐3), hepatic metabolism and injury (ALT, AST, GGT, ALP, DCP, bile acids), renal function and damage (eGFR, UACR, cystatin C, KIM‐1, NGAL), neuroimmunity (Aβ42/40, MOG‐IgG, LGI1 antibodies), and bone turnover (PINP, OPG, OC, β‐CTX, DKK‐1). By enabling integrative detection, it supports early diagnosis, stratification, and treatment monitoring.

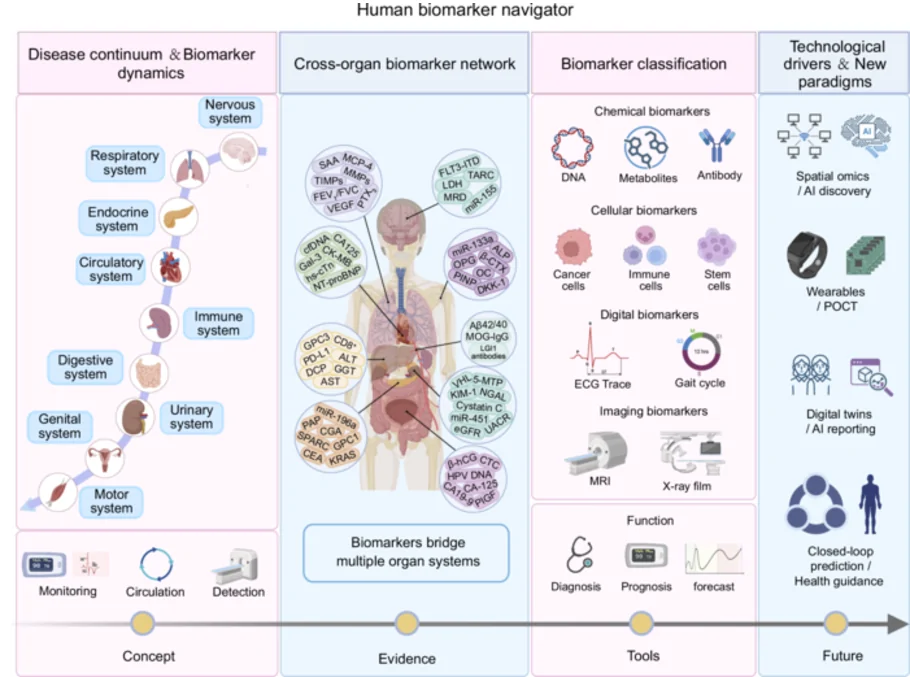

## INTRODUCTION

Throughout life, environmental exposure, lifestyle, infections, chronic inflammation, metabolic remodeling and aging continuously affect the body [1]. The ability to maintain core health metrics, namely, barrier integrity, circadian rhythm, immune balance, metabolic flexibility, and neurocognitive function, determines the trajectory from health to subhealth and ultimately to disease. Traditional medicine's reliance on static assessments focused on “disease presence” or “symptom occurrence” is increasingly inadequate for addressing modern challenges characterized by high chronic disease prevalence, widespread comorbidities, and an expanding disease spectrum [2]. Most major chronic diseases remain latent in asymptomatic or mildly imbalanced stages. Quantifiable early abnormalities can appear years before clinical events [3]. Thus, accurately identifying risks, assessing health resilience, and implementing proactive interventions before clinical manifestations have become central challenges in biomedicine and public health [4].

Biomarkers link biological processes, health status and clinical outcomes. In a broad sense, they include traditional clinical indicators, histological features, and multidimensional information such as genes, epigenetic modifications, proteins, cytokines, metabolites, microbiota, images, electrophysiology, and digital behavioral signals [5]. To provide a clear conceptual framework, biomarkers can be classified into several key types: imaging, protein, metabolite, biochemical, immune, and genetic markers. Each category serves distinct clinical purposes, including diagnosis, prognosis, treatment response prediction, disease monitoring, and risk stratification. This classification connects the conceptual understanding of biomarkers and the subsequent discussion on cross‐system integration. However, current biomarker studies face two major structural challenges [6]. “Disease siloing” confines most research to single diseases or organs and lacks systematic integration of common pathological nodes across systems [3]. In addition, “single‐modality bias” limits generalizability in real‐world multimorbidity contexts. Aging and multimorbidity are the norm and are driven by inflammatory, metabolic, immune, and neuroendocrine networks that cause polyorgan chain damage through highly coupled mechanisms [6]. Therefore, a higher‐dimensional, more systematic framework is urgently needed.

Against this backdrop, this review proposes holistic “human biomarkers,” grounded in dynamic homeostasis and resilience. It covers nine major systems and multiple critical organs and tissues [4] and synthesizes biomarker evolution pathways for 65 representative diseases. The 65 diseases were selected on the basis of multiple criteria: high prevalence or mortality, substantial contribution to global disease burden, maturity of biomarker research, representation of diverse organ systems and key pathological processes, and clinical significance in diagnosis, prognosis or management. This curated set provides a practical framework for cross‐system integration and illustrates the feasibility of dynamic biomarker navigation across health, subhealth and disease stages. While existing reviews and single‐disease databases offer valuable insights into specific conditions, they are typically limited to static descriptions or isolated analyses. In contrast, the present work adopts a holistic, cross‐system perspective, integrating biomarkers across multiple organs and diseases while emphasizing dynamic navigation along the health–subhealth–disease continuum. This approach facilitates the identification of shared pathological hubs, bolsters cross‐disease comparisons, and builds an interactive framework for multidimensional biomarker knowledge, which represents a conceptual advancement over previous single‐disease or system‐focused resources.

Moreover, through the integration of biomarkers for various diseases, we developed the “Human Biomarker Navigator” website (http://www.hbiomarker.com). This website serves to delineate the biomarker–disease–human health map and to construct the biomarker network. In contrast to the biomarker AI navigation platform of AstraZeneca, which is a website that leverages AI to extract and organize biomarker knowledge from large‐scale datasets, the “Human Biomarker Navigator” platform emphasizes the integration and cross‐system navigation of established cross‐organ and cross‐disease biomarker knowledge. The combined utilization of the two platforms indicates that computational discovery and the integration of structured knowledge can synergistically improve our understanding of biomarkers in the health–impairment–disease continuum.

**FIGURE 1:**
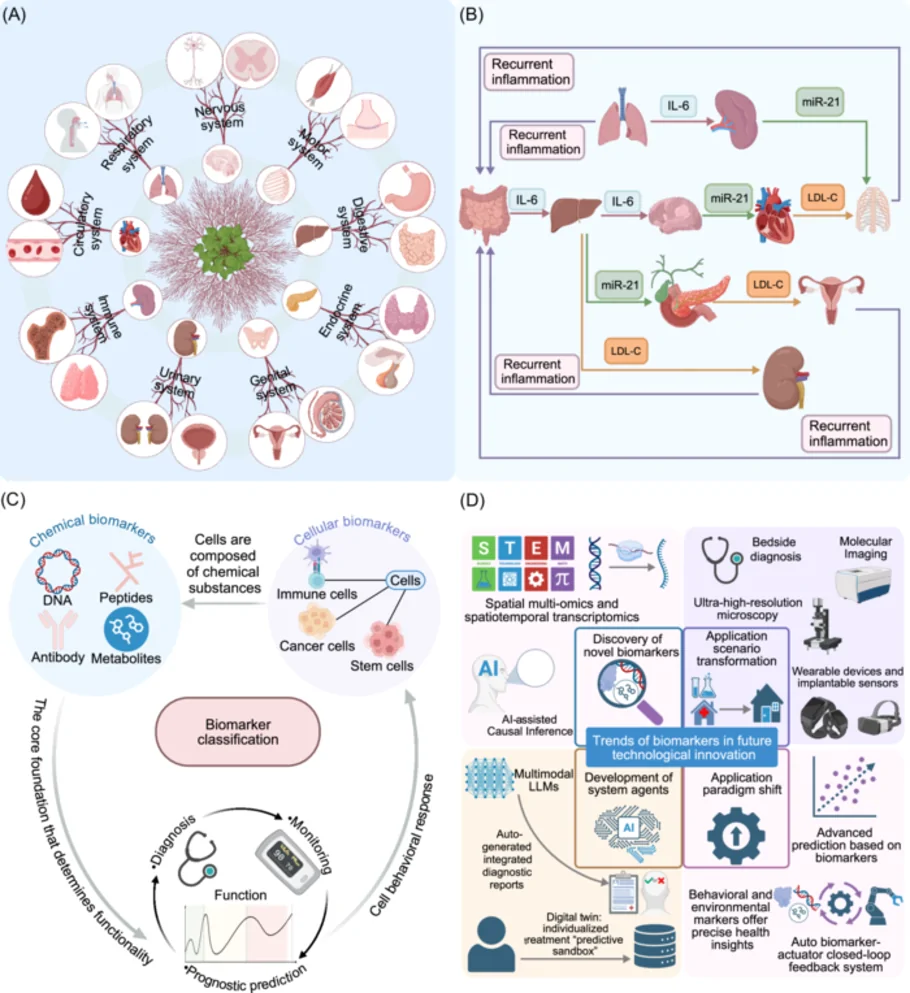
Integrative multi‐omics and biomarker classification for precision health and disease monitoring. (A) Shows a radial diagram linking key systems, including the nervous, cardiovascular, and immune systems, to central biomarker networks. (B) Depicts the pathways of inflammation recurrence and its impact on various organs, such as the lungs, heart, and pancreas, highlighting the role of IL‐6, miR‐21, and LDL‐C as key mediators. (C) Categorizes biomarkers into chemical and cellular types, focusing on their composition and functional significance in cellular behavior and response. (D) Outlines the future of biomarker research, emphasizing technological advancements in spatial multi‐omics, AI‐assisted causal inference, and the development of system agents for personalized health management.

The novel contribution of this work lies in the establishment of the first “pan‐disease model” framework that enables cross‐system, cross‐organ and cross‐disease integration of biomarkers. This approach transcends the traditional reductionist paradigm of “one disease‐one marker” and instead builds a unified panorama linking the health continuum, comorbidity networks and multimodal biomarkers [7]. Such integration provides a foundational platform for building a translatable and scalable population health risk management system (Figure 1A–D).

## PLATFORM OVERVIEW: HUMAN BIOMARKER NAVIGATOR

In recent decades, biomarker research has produced significant results, with a multitude of molecular markers identified that have diagnostic, prognostic, or therapeutic significance. However, these findings are often dispersed across various literature and databases pertaining to different disease domains, organ systems, and technical platforms, leading to a “data silo” phenomenon. Researchers seeking a comprehensive understanding of the expression patterns and clinical implications of a specific biomarker across multiple diseases often need to consult numerous publications simultaneously, a process that is inefficient and makes ensuring information completeness and timeliness challenging. Similarly, clinicians face difficulties in rapidly retrieving all relevant biomarkers from a single‐organ or single‐disease perspective when dealing with patients who present with complex comorbidities, making systematic judgments challenging. This fragmented status quo significantly impedes the shift in precision medicine from a “single‐disease focus” to a “systemic whole‐body assessment.” Consequently, there is an urgent need to construct an open‐access platform that incorporates data integration, intelligent retrieval, and visual analytics to overcome current bottlenecks in biomarker research and application.

### Platform positioning and guiding philosophy

The Human Biomarker Navigator (http://www.hbiomarker.com/) has been developed to address this need. It is not only a comprehensive biomarker data repository but also an intelligent retrieval and analysis platform designed for researchers and clinicians. The core concept of this platform can be encapsulated by three key terms: multidimensionality, cross‐system integration, and interactivity. Multidimensionality refers to the characterization of each biomarker across multiple layers, including molecular identity, functional roles, disease associations, organ localization, pathological mechanisms, clinical applicability, and dynamic annotations. Cross‐system integration denotes the bridging of information barriers between different organs and diseases, facilitating bidirectional tracing from biomarkers to organs to diseases and networked associations. Interactivity is demonstrated through various search interfaces and output formats, enabling users to engage in flexible dialog with the platform and integrate fragmented knowledge points into systematic knowledge graphs. Ultimately, this platform aims to propel biomarker research from “static knowledge enumeration” toward “dynamic navigation assistance,” providing a robust information infrastructure for precision medicine and systems medicine.

### Deep structured analysis of a single biomarker

In the Human Biomarker Navigator, each biomarker is associated with a highly standardized and comprehensive profile. This profile extends beyond a mere list of fields to offer an in‐depth characterization across seven interconnected dimensions. The first dimension provides identifier information, encompassing the biomarker's standardized name; prevalent abbreviations; molecular type, such as protein, nucleic acid, or metabolite; molecular formula; and molecular weight, thereby ensuring precise identification and localization. The second dimension delineates core functionality, specifying whether the biomarker is predominantly employed for early disease diagnosis, prognostic assessment, targeted therapy guidance, or prediction of treatment response in clinical settings. The third dimension elucidates the disease context by enumerating all related diseases and their subtypes, aiding users in understanding its pathological significance. The fourth dimension furnishes details on organ and sample specifics, highlighting the primary organs involved or affected; sample types for detection, such as serum, urine, cerebrospinal fluid, or tissue sections; and pertinent technical platforms, such as enzyme‐linked immunosorbent assays, polymerase chain reactions, or high‐throughput sequencing. The fifth dimension articulates pathobiological mechanisms, succinctly describing biological dysfunctions indicated by aberrant biomarker levels, such as uncontrolled cell proliferation, immune evasion, metabolic reprogramming, or neurodegenerative alterations. The sixth dimension emphasizes the clinical availability status, summarizing crucial aspects such as inclusion in clinical guidelines, approval of relevant diagnostic kits, participation in drug development pipelines, or orphan drug designation status. The seventh dimension offers annotations and dynamic updates, integrating recent research insights into biomarker behavior under particular treatments and its synergistic effects during combination therapies.

### Three core retrieval modes and cross‐system navigation

To cater to the varied usage habits and unique requirements of different users, the platform has developed three distinct yet complementary retrieval modes. These modes utilize biomarkers, organs, and diseases as search entry points and function independently of each other.

In the biomarker retrieval mode, upon inputting a known biomarker name or abbreviation, the platform yields all associated organ and disease information linked to that specific biomarker. This mode's unique advantage is its ability to reveal how a single biomarker concurrently engages in pathological processes across various organs and diverse diseases, thereby pinpointing common hub molecules shared among different pathologies. For example, the levels of certain inflammatory factors are elevated not only in cardiovascular diseases but also in neurodegenerative disorders and metabolic syndrome. This retrieval method visually demonstrates their “one cause, multiple effects” network characteristics, offering mechanistic insights into systemic inflammation and comorbidity mechanisms.

In the organ retrieval mode, upon inputting a specific organ name, the platform presents an array of diseases associated with that organ along with their respective biomarkers. Users have the option to delve deeper by clicking on individual biomarker links, which leads them to comprehensive profiles brimming with detailed information. Moreover, the system allows users to choose multiple biomarkers related to a single organ and generate an organ–biomarker network diagram effortlessly with just a click. This visual representation positions the organ at its core, branching out to interconnected biomarker nodes, thereby elucidating the intricate relationship between localized organ pathologies and broader systemic biomarker patterns. Such visualizations offer invaluable insights for comprehending synergistic variations among multiple biomarkers in organ‐specific diseases, discovering new biomarker combinations, and enhancing multidisciplinary consultations.

In the disease retrieval mode, upon inputting a specific disease name, the platform presents the primary organs affected by the disease and organizes associated biomarkers into categories on the basis of stages of disease progression, pathological subtypes, or functional markers. Users can also select multiple biomarkers and generate a disease–biomarker network diagram. This visual representation focuses on the disease, cohesively linking biomarkers with diverse functions across various stages and underscoring the inherent heterogeneity of the disease and the evolving patterns of biomarkers throughout the clinical trajectory. Such insights offer invaluable guidance for molecular disease classification, crafting personalized treatment strategies, and evaluating prognoses.

### Network visualization and systemic association analysis

The core outputs of the three retrieval modes described above can all be presented in the form of a dynamically generated system–organ–disease–biomarker network. This relationship map is not a static preset picture but an interactive network that is constructed in real time according to user input queries. Users can explore the strength and directionality of connections between nodes within the network through dragging, zooming, highlighting, and other operations. Additionally, the platform supports the export of network diagrams into high‐resolution images for direct use in academic papers, conference presentations, or teaching materials. The value of this network visualization function lies in its ability to transform multidimensional associations originally hidden within the textual literature into spatial topological structures that are easy to understand intuitively. This capability enables researchers to quickly discover previously overlooked cross‐system connections, generate testable scientific hypotheses, and establish a shared language and coordinate system for interdisciplinary collaborative research. From a systems biology perspective, this network‐based analytical approach embodies the “cross‐system, cross‐organ, cross‐disease integration” philosophy advocated by this review, thereby translating theoretical frameworks into operationalizable practical applications.

### Data update mechanisms, quality control, and long‐term maintenance

To ensure the timeliness, accuracy, and sustainability of platform information, the Human Biomarker Navigator has established a comprehensive data update mechanism and quality control system. In terms of data updates, the platform employs a strategy that combines periodic batch updates with continuous incremental updates. A quarterly full‐literature search and database synchronization are conducted to ensure timely inclusion of newly published high‐quality research findings. Moreover, for biomarker entries already included in the database, the platform continuously tracks emerging disease associations, clinical guideline revisions, and drug approval progress, completing information iteration within weeks according to priority levels. With respect to quality control, all the newly entered data must pass through two checkpoints: automated format validation and expert manual review. Automated validation checks data integrity, format standardization, and cross‐entry consistency, whereas manual review involves domain experts verifying and correcting core information. For information with controversies or insufficient evidence levels, confidence level indicators are marked in corresponding fields for user evaluation. Additionally, the platform maintains an open user feedback channel, allowing researchers to submit corrections, supplementary data, or new integration proposals. These submissions undergo the same review process and are updated promptly upon confirmation. For long‐term maintenance, supported by stable academic institutional backing and sustained research project funding, the platform has developed a technical roadmap spanning more than 5 years. Maintenance activities include optimizing underlying database structures, upgrading retrieval algorithms, improving user interface usability, and expanding new data types. Through this systematic updating and maintenance framework, the Human Biomarker Navigator is committed to becoming a trustworthy, iterative, and sustainable biomarker knowledge infrastructure.

### Present state of platform applications

The Human Biomarker Navigator has been increasingly utilized in biomedical research, with numerous teams leveraging the platform to identify diagnostic biomarkers for various diseases, screen novel therapeutic targets, and validate prognostic indicators. The platform's highly structured data and flexible visualization tools significantly facilitate cross‐domain information integration, promoting a shift from a “single‐disease” perspective to a “whole‐body systemic evaluation.” This review systematically outlines the theoretical framework of cross‐system navigation for biomarkers, using the Human Biomarker Navigator as a practical embodiment of this framework. These two elements complement each other: the review provides conceptual guidance, while the platform offers data resources and technical instruments. We invite researchers and clinicians globally to explore this platform (http://www.hbiomarker.com), supported by its extensive database and intelligent search functionality, to conduct their own systematic biomarker explorations and contribute to the advancement of precision health and systems medicine.

## NERVOUS SYSTEM

Literature retrieval was conducted in PubMed and Web of Science using Boolean logic with field‐restricted search strings, with all queries limited to title/abstract fields. The detailed Boolean strings for each disease were as follows: for Alzheimer's disease (AD): (“Alzheimer's Disease” OR “AD”) AND (“Aβ42/40 ratio” OR “phospho‐tau 181” OR “p‐tau181” OR “phospho‐tau 217” OR “p‐tau217” OR “neurofilament light chain” OR “NfL” OR “glial fibrillary acidic protein” OR “GFAP”); for Parkinson's disease (PD): (“Parkinson's disease” OR “PD”) AND (“alpha‐synuclein seed amplification assay” OR “α‐syn SAA” OR “CSF oligomers” OR “GBA1 gene mutation”); for amyotrophic lateral sclerosis (ALS): (“amyotrophic lateral sclerosis” OR “ALS”) AND (“plasma neurofilament light chain” OR “plasma NfL” OR “TDP‐43 protein aggregation”); for frontotemporal dementia (FTD): (“frontotemporal dementia” OR “FTD”) AND (“plasma neurofilament light chain” OR “plasma NfL” OR “MAPT gene mutation” OR “GRN gene mutation” OR “C9orf72 gene mutation”); for major depressive disorder (MDD): (“major depressive disorder” OR “MDD” OR “depression”) AND (“brain‐derived neurotrophic factor” OR “BDNF” OR “BDNF levels” OR “C‐reactive protein” OR “CRP” OR “interleukin‐6” OR “IL‐6” OR “hypothalamic‐pituitary‐adrenal axis” OR “HPA axis” OR “hypothalamic‐pituitary‐adrenal axis activity”); for schizophrenia: (“schizophrenia” OR “schizophrenic disorder” OR “schizophrenia spectrum disorders”) AND (“neurodevelopment‐related genes” OR “neurodevelopmental risk genes” OR “STAG1 gene” OR “ZNF136 gene” OR “SLC6A1 gene” OR “KLC1 gene” OR “PCLO gene” OR “ZMYND11 gene” OR “BSCL2 gene” OR “CGREF1 gene” OR “NDEL1 gene” OR “inflammatory cytokine profile” OR “inflammatory markers” OR “Interleukin‐1β“ OR “IL‐1β“ OR “interleukin‐6” OR “IL‐6” OR “interleukin‐4” OR “IL‐4” OR “interleukin‐10” OR “IL‐10” OR “tumor necrosis factor‐α“ OR “TNF‐α“ OR “interferon‐γ“ OR “IFN‐γ“ OR “neutrophil‐lymphocyte ratio” OR “NLR” OR “C‐reactive protein” OR “CRP” OR “homocysteine” OR “HCY”); for glioblastoma (GBM): (“glioblastoma” OR “glioblastoma multiforme” OR “GBM”) AND (“IDH1 mutation status” OR “IDH2 mutation status” OR “IDH1/2 mutation status” OR “MGMT promoter methylation” OR “MGMT met” OR “O6‐methylguanine DNA methyltransferase promoter methylation” OR “1p19q codeletion” OR “1p/19q co‐deletion” OR “1p and 19q codeletion”); for meningioma: (“meningioma” OR “sporadic meningioma” OR “intracranial meningioma” OR “ectopic meningioma”) AND (“NF2 gene mutation” OR “neurofibromatosis type 2 gene mutation” OR “NF2 pathogenic mutation” OR “NF2 germline mutation” OR “NF2 somatic mutation” OR “NF2 loss of heterozygosity” OR “NF2 LOH” OR “22q12 NF2 mutation”); for multiple sclerosis (MS): (“multiple sclerosis” OR “MS”) AND (“CSF oligoclonal bands” OR “cerebrospinal fluid oligoclonal bands” OR “OCB” OR “CSF OCB” OR “neurofilament light chain” OR “NfL” OR “CSF neurofilament light chain” OR “CSF NfL” OR “serum neurofilament light chain” OR “serum NfL”); and for epilepsy: (“epilepsy” OR “epileptic seizures” OR “refractory epilepsy”) AND (“anti‐NMDAR antibodies” OR “anti‐LGI1 antibodies” OR “anti‐CASPR2 antibodies” OR “anti‐GABAA receptor antibodies” OR “anti‐AMPAR antibodies” OR “anti‐DPPX antibodies” OR “anti‐IgLON5 antibodies” OR “anti‐mGluR1 antibodies” OR “anti‐mGluR5 antibodies” OR “neuronal autoantibodies” OR “specific neuronal antibodies” OR “electroencephalogram patterns” OR “EEG patterns” OR “electroencephalography findings” OR “EEG features”). The search time frame was set from January 1, 2019 to December 31, 2025. Only articles published in English were considered. Both original research articles and systematic or narrative reviews were included, with priority given to classic milestone reviews that systematically elucidate pathological mechanisms. For research articles, only human clinical studies, including cohort, observational, and confirmatory studies, were considered. A two‐step screening process was adopted. Initially, retrieved results were filtered to retain high‐quality reviews focusing on biomarkers of neurological diseases. Subsequently, based on the publication dates of these reviews, further high‐quality clinical studies published thereafter were identified. Only those that performed clinical validation of core biomarkers and provided clear diagnostic or prognostic indicators were included, whereas in vitro experiments, animal studies, conference abstracts, and redundant reports were excluded. The final set of diseases encompassed major neurological categories, including neurodegenerative, neuropsychiatric, central nervous system tumors, autoimmune inflammatory, and epileptic disorders, all characterized by high incidence, substantial disease burden, mature biomarker systems, typical pathological features, and strong clinical translational value.

### Alzheimer's disease

Alzheimer's disease (AD) is the most prevalent neurodegenerative dementia worldwide, characterized by progressive cognitive decline and hallmark pathologies including amyloid‑β plaques and tau neurofibrillary tangles [8]. With clinical diagnosis still predominantly relying on cognitive assessments and imaging, which are approaches prone to misdiagnosis and delayed intervention, biomarkers have emerged as essential tools for early detection, accurate diagnosis, and therapeutic evaluation [9]. Recent advancements in liquid biopsy and multi‐omics technologies have propelled AD biomarker research, transitioning from cerebrospinal fluid analysis to non‐invasive samples like blood and urine, and evolving from singular pathological indicators to comprehensive multi‐dimensional analyses (Table S1) [10].

#### The development process of AD biomarkers

The initial era encompassed conventional pathological and imaging biomarkers. Research on AD biomarkers originated from the observation of post‐mortem brain tissue pathology, where Aβ plaques and neurofibrillary tangles were considered the gold standard for diagnosis [11]. With the advancement of imaging technology, computed tomography (CT) and magnetic resonance imaging (MRI) were gradually applied in clinical practice, providing structural evidence for the diagnosis and staging of AD by quantifying the degree of atrophy in key areas such as the hippocampus and medial temporal lobe [12]. The emergence of positron emission tomography (PET) further advanced the development of imaging biomarkers. Amyloid PET allowed visualization of Aβ deposition in the brain, while tau PET accurately depicted the distribution of neurofibrillary tangles. Together, they became core tools for confirming AD pathology *in vivo* [13]. Despite significant progress in imaging technology, biomarker detection at this stage still relied on invasive pathology or costly imaging methods, making it difficult to be widely used in early screening and population surveys [14].

The exploration of fluid biomarkers is driven by advances in multi‐omics technologies. With the vigorous development of multi‐omics technologies such as proteomics, genomics and epigenomics, AD biomarker research has entered a new stage of non‐invasive and precise studies [15]. Cerebrospinal fluid, which directly interfaces with the brain microenvironment, has become a core focus of research. Indicators such as Aβ42/40 ratio, total tau (t‐tau) and phosphorylated tau (p‐tau181, p‐tau217, p‐tau231) have been confirmed to accurately reflect the pathological process of AD. At the same time, blood, due to its advantages of non‐invasiveness and easy accessibility, has become the focus of research. Breakthroughs in ultrasensitive detection technologies have made it possible to accurately quantify low abundance biomarkers such as Aβ, p‐tau and neurofilament light chain in plasma [16]. In addition, the continuous emergence of new biomarkers such as epigenetic markers, non‐coding RNAs and neuroinflammation‐related proteins further expands the boundaries of understanding the complex pathological mechanisms of AD [15, 16]. The progress of this stage successfully overcomes the limitations of traditional biomarkers and opens up a feasible path for early screening and accurate diagnosis of AD [10].

Recently, the field has progressed to omics integration and clinical translation. Current research on AD biomarkers has entered a new phase that emphasizes both multi‐dimensional integration and clinical translation. The focus of research has shifted from validating single biomarkers to constructing multi‐biomarker combination models. By integrating multi‐dimensional information such as Aβ deposition, tau pathology, neuroinflammation, and synaptic dysfunction, researchers have significantly enhanced diagnostic accuracy and prognostic prediction capabilities [9]. For instance, the diagnostic specificity of the combined detection model using plasma Aβ42/40 ratio and p‐tau217 exceeded 90%. In terms of clinical translation, blood biomarker detection systems are becoming increasingly mature. Reagents like PrecivityAD2 have entered clinical validation, providing practical tools for screening for AD in grassroots medical institutions [17]. Concurrently, biomarkers are playing an increasingly important role in clinical trials, serving as a key basis for subject stratification and efficacy assessment, thereby effectively promoting the development of disease‐modifying therapies. Overall, the core progress of this stage is the systematic leap from basic research to the clinical application of biomarkers, laying a solid foundation for the precision medicine system of AD.

#### The main types and typical cases of AD biomarkers

These diagnostic and screening biomarkers are primarily used for the early identification and pathological confirmation of AD. Core indicators focus on abnormal Aβ metabolism and tau‐related molecules. The ratio of Aβ42/40 is one of the most representative diagnostic markers. The CSF Aβ42/40 ratio assay, which reflects cerebral amyloid‐β pathology by measuring the selective decrease of Aβ42 in the presence of stable Aβ40, and serves as a core diagnostic biomarker for Alzheimer's disease, received FDA De Novo clearance in 2022 for use in adults aged 55 and older presenting with cognitive impairment. Due to the abnormal accumulation of Aβ42 in the brain, its levels in cerebrospinal fluid and plasma decrease accordingly, while Aβ40 remains relatively stable. Therefore, a reduction in this ratio reliably reflects the Aβ pathological burden [13]. The sensitivity and specificity of the CSF Aβ42/40 ratio for diagnosing AD were 87% and 88.2%, respectively, and the accuracy of plasma detection could also reach 82%–97%, which was highly consistent with amyloid PET imaging [10]. Regarding tau pathology, phosphorylated tau proteins are direct manifestations of neurofibrillary tangles. Among them, p‐tau217 exhibited the best performance, not only effectively distinguishing AD from other neurodegenerative diseases but also demonstrating superior specificity compared to p‐tau181. Notably, plasma p‐tau217 has already increased during the preclinical stage of AD and has even shown abnormalities as early as 24 years prior in familial AD, making it the preferred indicator for preclinical screening [8, 18].

At the genetic level, *APOEε4* is the most important genetic susceptibility marker for AD. It significantly increases the risk of disease by influencing Aβ clearance and lipid metabolism. The risk in ε4 homozygous carriers is approximately 34‐fold higher than that in noncarriers [8]. *APOE* genotyping based on whole blood can be used to stratify risk but cannot serve as an independent diagnostic basis because it only reflects genetic predisposition [16]. In addition, epigenetic markers such as abnormalities in promoter methylation of *APP* and *PSEN1* genes regulate gene expression and affect Aβ production. Changes in these markers in peripheral blood may indirectly reflect pathological conditions in the brain, especially in familial AD, where this effect is more pronounced [15].

These prognostic and monitoring biomarkers are designed to assess the rate of disease progression and treatment response, with an emphasis on neuronal injury and neuroinflammation progression. neurofilament light chain (NfL) is a molecule associated with axonal damage. It is significantly elevated in cerebrospinal fluid and plasma from AD patients and positively correlated with cognitive decline rates and brain atrophy [12]. Although NfL also exists in other neurological diseases such as multiple sclerosis and amyotrophic lateral sclerosis (ALS), it lacks disease specificity; However, it has been explored as a pharmacodynamic indicator in clinical trial research of AD to evaluate the potential effects of neuroprotective treatments [9]. Glial fibrillary acidic protein (GFAP) serves as a marker of astrocyte activation. It increases before the onset of AD symptoms and is closely related to Aβ pathology. It can predict the conversion of mild cognitive impairment to AD, and its dynamic changes even precede some tau markers [14].

In the context of neuroinflammation, YKL‐40 is primarily secreted by activated astrocytes and exhibits significantly elevated levels in both the cerebrospinal fluid and plasma of patients with AD. This protein is thought to participate in the tau pathological process that may be influenced by Aβ and can be utilized as an auxiliary indicator for evaluating the extent of neuroinflammation [19]. The soluble form of myeloid cell triggering receptor 2 (sTREM2) serves as a crucial marker for microglial cell activation. Its concentration in the cerebrospinal fluid peaks during the stage of mild cognitive impairment, exhibiting a characteristic “inverted U‐shaped” kinetic pattern. Individuals who carry the *TREM2* R47H variant face a 3‐4 fold increased risk of developing AD, thereby positioning this receptor as a potential therapeutic target [20].

Synaptic dysfunction is one of the core mechanisms of cognitive impairment in AD. Relevant markers can effectively reflect the degree of synaptic integrity damage. SNAP‐25, as a key protein for synaptic vesicle fusion, may be released into cerebrospinal fluid and plasma following synapse injury. Its level has increased in the preclinical stage of AD and positively correlated with Aβ pathology. It is an early sensitive indicator reflecting synaptic degeneration [18]. Neurogranin (Ng) is mainly expressed in dendritic spines and participates in regulating synaptic plasticity. The Ng level in the cerebrospinal fluid of AD patients increases and is closely related to the severity of tau pathology and the rate of memory decline. In addition, through PET imaging of synaptic vesicle glycoprotein 2A (SV2A), brain synaptic density can be directly evaluated. The binding rate of SV2A in the hippocampus and temporal lobe cortex of AD patients generally decreases and positively correlates with cognition [13].

In addition to the aforementioned indicators, GAP‐43 and synaptotagmin‐1 have also been implicated in AD‐induced synaptic damage. Alterations in their cerebrospinal fluid levels may serve as supplementary markers for disease progression [18].

Among the imaging biomarkers and digital biomarkers, amyloid PET and tau PET are core imaging biomarkers for confirming AD pathology in vivo. Amyloid PET allows for the visualization of Aβ deposition, whereas tau PET precisely portrays the distribution of neurofibrillary tangles. Collectively, they have emerged as indispensable tools for clinical diagnosis and the selection of patients for anti‐Aβ therapies [21]. Structural MRI, by quantifying atrophy in the hippocampus and medial temporal lobe, provides critical structural evidence for diagnosis and staging [22].

#### Current challenges and future development trends

Although significant progress has been made in AD biomarker research, it still faces multiple challenges. Firstly, AD is highly heterogeneous and can be divided into familial and sporadic types as well as Aβ‐dominant and tau‐dominant subtypes. It is difficult for a single biomarker to comprehensively capture the pathological characteristics of each subtype [17]. For example, some sporadic AD patients show negative results for Aβ‐related biomarkers and only exhibit tau pathology abnormalities, resulting in reduced efficacy of traditional Aβ‐based diagnostic models [9]. Secondly, existing fluid biomarkers generally lack disease specificity and may increase in other neurodegenerative diseases, limiting their independent application in differential diagnosis [14]. Thirdly, clinical translation remains an obstacle. Most new biomarkers are still in the research stage, lacking unified detection standards and reference thresholds, and the stability and cross‐platform consistency of blood biomarkers also need improvement [10]. Finally, early diagnosis is still facing bottlenecks. Patients in the preclinical stage of AD often have no obvious symptoms, and there is currently a lack of high‐sensitivity biomarker combinations that can identify high‐risk individuals before significant brain functional damage occurs [17].

Future research on AD biomarkers will focus on the following directions. First, precision classification involves the application of multi‐omics technologies to identify subtype‐specific biomarkers and to develop diagnostic models for distinct AD subtypes, including amyloid‐beta‐negative AD, tau‐dominant AD, and familial AD [9]. Second, technology‐driven innovation: combining spatial omics and artificial intelligence with other cutting‐edge methods to integrate clinical, imaging, molecular and other multidimensional data, developing composite biomarker systems with high sensitivity and specificity [23]. At the same time, new technologies such as exhaled volatile organic compound detection and exosome biomarker analysis are also expected to further expand the boundaries of non‐invasive detection [24]. Third, integration and dynamic monitoring: establishing a full‐cycle “risk screening‐diagnosis confirmation‐prognosis assessment‐efficacy monitoring” biomarker system, promoting the development of markers that can dynamically reflect disease activity and treatment response [25]. For example, jointly detecting the dynamic changes of plasma p‐tau217, GFAP, and NfL to evaluate the therapeutic response of anti‐Aβ drugs [16]. Fourth, targeted application and transformation: prioritizing the clinical transformation of early screening biomarkers, developing low‐cost and easy‐to‐operate test kits to achieve large‐scale community‐level screening [16]; at the same time, strengthening the research on specific biomarkers for mechanisms such as neuroinflammation and synaptic repair, providing key support for the development of next‐generation targeted drugs [19].

### Parkinson's disease

Parkinson's disease (PD), the second most common neurodegenerative disorder, is defined by progressive dopaminergic neuron loss and α‑synuclein aggregation into Lewy bodies [26, 27]. Because clinical symptoms emerge only after substantial neuronal loss, and with diagnosis still relying on clinical presentation, biomarkers are critically needed for early diagnosis, disease monitoring, and guidance of targeted therapies [26, 28]. In recent years, integration and application of technologies such as metabolomics, genomics and imaging have promoted researches on PD biomarkers from the detection of single indicators to a new stage of multi‐dimensional and integrated biomarker system (Table S2) [29].

#### The development process of PD biomarkers

The early stage was a traditional detection and preliminary exploration period. From the late 20th to early 21st century, research on PD biomarkers primarily relied on conventional imaging and basic laboratory tests. In terms of imaging, PET was initially employed to visualize neuroinflammation in the brain. The first‐generation translocator protein (TSPO)‐targeted tracer [24] PK11195 could label activated microglial cells [30, 31], but it had limitations such as low resolution and insufficient specificity. Laboratory tests mainly used cerebrospinal fluid and blood samples. It was found that decreased total α‐synuclein levels in cerebrospinal fluid could distinguish PD patients from healthy individuals [26, 27], and serum inflammatory factors such as IL‐6 and TNF‐α levels were associated with disease activity [28, 30]. During this stage, biomarkers were mostly single‐level indicators with limited diagnostic sensitivity and specificity and had not yet achieved routine clinical application.

The next stage is the multi‐omics technology‐driven era. With the emergence of multi‐omics technologies such as proteomics, metabolomics and genomics, PD biomarker research has entered a systematic screening stage [32]. Proteomics discovered phosphorylated α‐syn (pS129) and α‐syn seed aggregate forms, whose detection performance was significantly better than total α‐syn; metabolomics revealed abnormal metabolites such as uric acid, indole lactic acid and canuline, which reflected pathological states such as oxidative stress and gut–brain axis disorders; genomics identified pathogenic gene mutations such as *LRRK2*, *GBA1* and *SNCA*, providing a basis for genetic risk research; transcriptionomics found noncoding RNAs such as miR*‐*124 involved in regulating α‐syn metabolism [33, 34]. This stage broke through the limitations of single indicators and revealed the pathogenesis of PD from multiple dimensions, laying a foundation for developing combined biomarkers.

Finally, the omics integration and clinical translation phase. Currently, PD biomarker research has entered a new stage of multi‐omics integration and clinical translation. By integrating multidimensional data such as proteins, metabolites, nucleic acids, and imaging, composite biomarker models are constructed to significantly improve diagnostic accuracy. For example, combined detection of α‐syn seeds and plasma neurofilament light chain can achieve early diagnosis and disease progression monitoring [35, 36], while [^18^F]‐FEPPA PET imaging combined with cerebrospinal fluid inflammatory factors can assess the state of neuroinflammation [30, 31]. The application of artificial intelligence and organoid technology further accelerates clinical translation, such as metabolic panel diagnosis based on machine learning with sensitivity up to 83% [32], and exosome α‐syn detection entering clinical trials [37]. The core goal of this stage is to realize the transformation of biomarkers from experimental research to clinical routine, supporting the establishment of precision medicine systems.

#### The main types and typical cases of PD biomarkers

Protein biomarkers in PD are centered on α‐synuclein and its derivatives. Total α‐syn can be detected in cerebrospinal fluid and plasma by ELISA, SIMOA, and other techniques. The level of α‐syn in the cerebrospinal fluid of PD patients decreased by about 30%, while abnormalities in plasma could appear 15 years earlier than clinical manifestations in familial PD patients [26, 27]. Phosphorylated α‐syn (pS129), as a key modified form of α‐syn aggregation, can be detected in plasma and red blood cells by IP‐MS, with a specificity of up to 89% for differentiating PD from multiple system atrophy [27]. The α‐syn seed amplified and detected by RT‐QuIC technology has a diagnostic sensitivity of 92% and specificity of 90% in cerebrospinal fluid samples, which has been associated with conversion risk in clinical studies [35, 36]. The α‐synuclein seed amplification assay (αSyn‐SAA), validated in April 2023 by the Parkinson's Progression Markers Initiative (PPMI), received a letter of support from the FDA in September 2024, encouraging its use in clinical trials for Parkinson's disease and related synucleinopathies. Neurofilament light chain, an indicator associated with axonal damage, showed positive correlations with motor symptom progression and has been used as an efficacy evaluation index in clinical trials of disease‐modifying treatments for this disease [28, 37]; GFAP reflects astrocyte activation, and a 30% increase in plasma levels predicted cognitive decline [30, 38].

Among the enzyme markers and cytokine markers, glucosylceramidase is a key enzyme marker, and its activity is reduced due to mutations in the *GBA1* gene, thereby hindering the clearance of α‐syn [38]. Enzyme activity determination and gene sequencing have been investigated in research for risk assessment and as exploratory pharmacodynamic markers in clinical trials of the small molecule activator ambroxol [38]. Cytokine markers primarily reflect the state of neuroinflammation. IL‐6 is thought to contribute to dopaminergic neuron damage, potentially via the JAK/STAT pathway, and its levels are significantly elevated in the cerebrospinal fluid of patients with PD and dementia [28, 30]; TNF‐α is associated with microglial activation and may be involved in neuronal apoptosis, and its cerebrospinal fluid level positively correlates with nigral neuron loss [30, 35]; High‐sensitivity C‐reactive protein serves as an indicator of systemic inflammation. An elevated level (>3 mg/L) is associated with a 1.8‐fold increased risk of PD onset, making it suitable for initial screening of high‐risk populations [29, 30].

Metabolic biomarkers are predominantly detected using LC‐MS/GC‐MS techniques. Uric acid exhibits antioxidant effects; individuals with plasma levels below 260 μmol/L demonstrate a 2.1‐fold increased risk of PD, establishing it as a potential non‐invasive screening indicator [27, 29]. Indole lactic acid, a metabolite derived from intestinal flora, shows a 40% reduction in the plasma of PD patients and is associated with gut‐brain axis dysfunction; probiotic intervention can modulate its concentration [27, 29]. Quinoline acid, resulting from an imbalance in the kynurenine pathway, possesses neurotoxic properties, and Its ratio to kynurenine reflects the degree of inflammation [27, 29]. Regarding nucleic acid biomarkers, *LRRK2* mutations elevate PD risk by inducing mitochondrial dysfunction and have been incorporated into genetic research for familial PD [26, 38]. *SNCA* mutations promote α‐syn aggregation, leading to an earlier disease onset by 10–15 years in carriers [27]. miR‐124 regulates α‐syn metabolism through *BACE1* inhibition; its plasma level is reduced by 40% in PD patients, and exogenous supplementation mitigates α‐syn aggregation in animal models [33, 34].

Imaging markers allow non‐invasive visualization of pathological changes in PD. [^18^F]‐FEPPA, a second‐generation TSPO‐targeted PET tracer, can specifically localize neuroinflammation in the striatum and differentiate PD from multiple system atrophy with an accuracy of 88% and dynamically monitor the efficacy of anti‐inflammatory treatment [30, 31]; retinal nerve fiber layer thickness measured by optical coherence tomography is reduced by 10% in PD patients compared to healthy individuals, correlates with dopaminergic neuronal loss in the substantia nigra, and is suitable for longitudinal follow‐up [31, 36]. Exosome‐related markers have become a research focus in recent years. Exosomal α‐synuclein participates in pathological intercellular transmission, and its plasma detection level positively correlates with disease stage; non‐invasive detection techniques have entered clinical trial phases [26, 37].

#### Current challenges and future development trends

Research on PD biomarkers still faces multiple bottlenecks. The disease is highly heterogeneous, and PD is actually a syndrome caused by multiple factors. It is difficult for a single biomarker to cover all subtypes [26]; the specificity is not high enough, as candidate biomarkers such as hs‐CRP and IL‐6 are mostly systemic indicators that also exist in other neurodegenerative or inflammatory diseases [30]; it is difficult to transform because most biomarkers are still in the research stage, lacking large‐scale multicenter validation and standardized detection procedures; early diagnostic sensitivity is limited, and there is still a lack of sensitive biomarkers that can identify high‐risk populations before symptoms appear. Most existing indicators only show significant changes in the middle stage of the disease [32].

Future research will move towards precision, technology‐driven approaches and integration. In terms of precision, marker combinations will be developed based on genetic backgrounds, pathological subtypes and clinical phenotypes, such as the combined detection of glucocerebrosidase activity and exosomal α‐syn in *GBA1*‐related PD [38]; regarding technology‐driven approaches, multi‐omics technologies, artificial intelligence and breath volatile organic compound analysis will facilitate the discovery of novel markers, such as metabolite‐protein joint models based on machine learning [32]; concerning integration and dynamic monitoring, multidimensional models will be constructed by integrating clinical, imaging and molecular data, and dynamic disease monitoring can be achieved through wearable devices or noninvasive detection tools [34]; for targeted applications, early screening, acute exacerbation prediction and targeted treatment guidance will be emphasized, such as using α‐syn seed detection to evaluate the efficacy of anti‐α‐syn drug clinical trials [35, 36]. With continuous technological advancements, PD biomarkers are expected to play a central role in precision medicine systems, providing support for early intervention and individualized treatment.

### Amyotrophic lateral sclerosis

ALS is a fatal neurodegenerative disease characterized by selective degeneration of upper and lower motor neurons, with rapid progression and a median survival of only 3‐5 years [39, 40]. Early symptoms are highly heterogeneous and often misdiagnosed, leading to delays of 8–15 months, underscoring the urgent need for biomarkers to aid early identification and disease tracking [40, 41]. Currently, there is no cure for ALS, making biomarkers invaluable for early identification, prognosis assessment, disease tracking, and guiding targeted treatment [42]. Recent advancements in proteomics, genomics, and neuroimaging technologies have propelled research on ALS biomarkers from single‐indicator detection to multi‐dimensional integrated systems (Table S3).

#### The development process of ALS biomarkers

The first stage was the traditional detection and preliminary exploration period. During this time, the focus was on conventional laboratories and basic imaging examinations. In laboratory testing, creatine kinase and its isoenzyme CK‐MB were found to be elevated in the serum of approximately 53.8% of ALS patients as markers of muscle damage, with particularly high levels observed in the spinal‐onset group [43]; neurofilament protein was initially discovered to be associated with axonal injury, and the level of pNfH in cerebrospinal fluid could aid in differentiating ALS from other neurodegenerative diseases [44]. In terms of imaging, conventional MRI could reveal high signals along the corticospinal tract on T2WI and FLAIR sequences, indicating upper motor neuron damage [45]. The markers during this phase were mostly single‐level indicators with limited diagnostic sensitivity and specificity, had not yet been applied clinically, but laid the groundwork for subsequent research.

The next stage is the multi‐omics technology driving era, with the emergence of multi‐omics technologies such as proteomics, genomics and transcriptomics, ALS biomarker research has entered a systematic screening stage [46]. Proteomics identified TDP‐43 protein aggregation as the core pathological marker of ALS, which exists in 95% of patients; proteins related to neuroinflammation, such as YKL‐40 and GFAP, are associated with disease progression [39]. Genomics clarified that *C9orf72* hexanucleotide repeat expansion and *SOD1* mutation are key genetic markers for familial ALS, and poly‐GP levels are significantly elevated in cerebrospinal fluid from *C9orf72* mutation carriers [47]. Transcriptomics found that non‐coding RNAs such as miR‐206 were abnormally expressed in the skeletal muscle and plasma of patients and involved in regulating myocyte differentiation [48]. In terms of imaging, diffusion tensor imaging can accurately assess microstructural damage to the corticospinal tract by quantifying fractional anisotropy reduction; [^18^F]‐FDG PET can track abnormal brain glucose metabolism and assist in pathological localization [42].

The next stage is the period of omics integration and clinical translation. Current studies have entered a phase of multi‐dimensional integration and clinical translation. By integrating protein, nucleic acid, and imaging data to construct composite biomarker models, diagnostic accuracy has been significantly improved [42]. For example, combining NfL and cTnT achieved an accuracy rate of 86% in differentiating ALS from pseudodiseases [43]. DTI parameters combined with ALSFRS‐R scores can predict disease progression rates [42]. Artificial intelligence and machine learning have accelerated biomarker transformation, such as SVM classifier based on FDG‐PET effectively distinguishing ALS from healthy controls [42]; RT‐QuIC technology increased sensitivity for detecting TDP‐43 aggregates in cerebrospinal fluid to 89%, entering clinical validation [45]. Additionally, non‐invasive detection technologies are rapidly advancing, with urinary p75ECD and plasma microRNA becoming research hotspots aimed at promoting systematic transition of biomarkers from laboratory research to routine clinical application [39, 45].

#### The main types and typical cases of ALS biomarkers

Among the protein markers, neurofilament light chain is a general neurodegenerative marker, which is significantly elevated in plasma and cerebrospinal fluid of ALS patients and closely related to disease progression and survival time. It has been investigated as a pharmacodynamic indicator in clinical trial research [41]. Its phosphorylated subtype, pNfH, is more specific, with levels in cerebrospinal fluid up to 14 times higher than those in healthy individuals, and can distinguish between spinal and bulbar onset subtypes [49]. TDP‐43 protein aggregation is considered a core pathological feature of ALS; it can be detected in cerebrospinal fluid, serum, and skin samples by immunoblotting and RT‐QuIC. Its level shows a positive correlation with the degree of RNA metabolic disorder [39]. Creatine kinase MB reflects denervation atrophy of muscles, with an increased serum level in approximately 53.8% of patients, and its combination with cTnT can improve the accuracy of myopathy differentiation [43]. In addition, YKL‐40 is secreted by activated microglia, and its level in cerebrospinal fluid is positively correlated with the degree of neuroinflammation [50]; GFAP indicates astrocyte activation, with higher plasma levels in *C9orf72* mutation type patients compared to sporadic cases [46].

Among the nucleic acid markers, the *C9orf72* hexanucleotide repeat expansion is the most prevalent cause of familial ALS and can be identified in whole blood using long‐read sequencing. mutation carriers exhibit a 3.2‐fold increase in poly‐GP levels in their cerebrospinal fluid, potentially offering a decade's advance warning of disease onset [39, 47]. *SOD1* mutations, which exacerbate oxidative stress and induce neuronal apoptosis, serve as exploratory markers in clinical trials of Tofersen [41, 48]. Among non‐coding RNAs, miR‐206 shows elevated expression in both skeletal muscles and plasma of patients, playing a role in neuromuscular junction repair; its external supplementation has been shown to enhance muscle fiber function in animal models. Additionally, *hsa_circ_0007099* and other circular RNAs display abnormal expression patterns in muscle tissues, correlating with disease progression [45, 46].

On structural MRI, voxel‐based morphometry can quantify the gray matter atrophy in motor cortex, and diffusion tensor imaging shows that fractional anisotropy of corticospinal tract is decreased and radial diffusivity is increased, suggesting axonal and myelin damage [51]; quantitative magnetization transfer imaging can detect iron deposition in motor cortex and its degree positively correlates with neuroinflammation [45]. On functional imaging, [^18^F]‐FDG PET demonstrates hypometabolism in motor cortex and prefrontal lobe, which correlates with disease severity [42]; TSPO‐targeted PET can localize microglial activation, providing a basis for monitoring targeted neuroinflammatory treatment [50]. In addition, optical coherence tomography detects retinal nerve fiber layer thickness whose changes are synchronous with spinal motor neuron loss, making it suitable for longitudinal follow‐up [42].

Diffusion tensor imaging (DTI) quantifies microstructural damage to the corticospinal tract through fractional anisotropy reduction, aiding pathological assessment. [^18^F]‐FDG PET tracks abnormal brain glucose metabolism and assists in pathological localization [52]. On conventional MRI, the “motor band sign” (hyperintensity along the corticospinal tract on T2WI and FLAIR sequences) indicates upper motor neuron damage [53].

Machine learning algorithms, such as support vector machine (SVM) classifiers based on FDG‑PET, effectively distinguish ALS from healthy controls [54]. Combining DTI parameters with ALSFRS‑R scores can predict disease progression rates, representing a form of digital biomarker integration [55].

### Other types of markers

Among the cellular markers, neutrophil‐to‐lymphocyte ratio (NLR) is a systemic inflammatory marker. The survival time of Patients with NLR > 3 was shortened by 42%, which can be easily detected by routine blood tests [40]; the decrease in number and dysfunction of Tregs IS positively correlated with disease progression, making them potential targets for immunotherapy [46]. In terms of metabolites, increased expression of pyruvate dehydrogenase kinase 4 reflects muscle mitochondrial dysfunction, and inhibiting its activity improves muscle function in model animals [41]; decreased uric acid levels are associated with an increased risk of ALS and have the potential to be used as noninvasive screening [47]. Significantly elevated urinary nerve growth factor receptor p75 extracellular domain level can reflect disease severity and provide a new method for noninvasive monitoring [39, 46].

#### Current challenges and future development trends

Research on ALS biomarkers still faces several bottlenecks. The disease is highly heterogeneous, and genetic and environmental factors contribute to different subtypes, making it difficult for a single biomarker to cover all aspects comprehensively [47]; the specificity is insufficient, as biomarkers such as NfL and YKL‐40 are also found in other diseases like FTD and multiple sclerosis [49]; clinical translation is challenging because most biomarkers lack largDiffusion tensor imaginge‐scale multicenter validation and standardized detection procedures [41]; early diagnosis sensitivity is low, and there remains a lack of sensitive biomarker combinations that can identify high‐risk individuals before symptoms appear [45]; some tests are invasive, and the inconvenience of cerebrospinal fluid collection limits their routine application [43].

Future research will focus on precision, technology‐driven approaches, and multidimensional integration. In terms of precision, marker combinations based on genetic background and clinical subtypes will be developed, such as the combined detection of poly‐GP and NfL in *C9orf72*‐related ALS [47]. In terms of technology‐driven aspects, spatial transcriptomics, single‐cell proteomics, and artificial intelligence will facilitate new marker discovery and modeling of multimodal data fusion [51]; breath volatile organic compound analysis and noninvasive nucleic acid testing are expected to promote minimally invasive detection applications. In terms of integration and dynamic monitoring, wearable devices and liquid biopsy technologies will be integrated to achieve real‐time tracking of disease progression and synchronous analysis of multiple markers [41]. At the target level, emphasis will be placed on early screening, deterioration prediction, and targeted treatment guidance, such as TDP‐43‐based efficacy evaluation [50]. With continuous technological evolution, ALS biomarkers are expected to play a central role in precision medicine systems, providing support for early intervention and individualized treatment.

### Frontotemporal dementia

FTD encompasses a group of neurodegenerative disorders characterized by progressive frontal and temporal lobe atrophy, with clinical presentations including behavioral and language variants [56]. As the second most common early‑onset dementia, FTD is highly heritable yet often misdiagnosed as psychiatric illness, leading to substantial diagnostic delays and highlighting the need for reliable biomarkers [39, 57]. The traditional diagnosis depends on clinical manifestations and structural imaging, which makes it difficult to achieve early identification and pathological classification. Biomarkers, as objective biological indicators that reflect the pathological process of the disease, play an important role in the early differentiation, subtype division, and prognosis evaluation of FTD [58, 59]. In recent years, molecular biology combined with imaging technology has greatly promoted the research progress of FTD biomarkers. However, there are still many challenges in their clinical transformation (Table S4).

#### The development process of FTD biomarkers

Research on FTD biomarkers has progressively deepened with the advancement of neuroscientific technologies, progressing through three primary stages. Initially, investigations centered on pathological and genetic markers. Given the substantial heterogeneity in FTD pathology, where tau protein lesions account for approximately 45%, TDP‐43 protein lesions make up around 50%, and FUS protein lesions constitute about 5%, early diagnosis was largely dependent on autopsy findings [58]. As gene sequencing technology evolved, mutations in genes such as *MAPT*, *GRN*, and *C9orf72* were identified as the core genetic underpinnings of familial FTD, enabling in vivo genetic diagnosis and risk stratification [56, 60].

The second stage focuses on the development of fluid and imaging biomarkers. Proteins such as neurofilament light chain and TDP‐43 in cerebrospinal fluid have been demonstrated to effectively reflect neuronal damage and pathological aggregation [58, 61]; breakthroughs in plasma detection technologies now enable non‐invasive quantification of biomarkers, including neurofilament light chain and progranulin [62, 63]. In functional imaging, [^18^F]‐FDG PET facilitates differentiation between FTD and AD through identification of hypometabolism in frontal and temporal lobes [64], while tau PET tracers enable specific visualization of tau deposits in the brains of *MAPT* mutation carriers [65].

In the third stage, multimodal integration and precision research are emphasized. Researchers integrate multiple omics markers, such as genetics, body fluids, and imaging to construct diagnostic and prognostic models [66]; artificial intelligence and machine learning further enhance classification and prediction capabilities. Non‐invasive detection techniques and cutting‐edge omics technologies have been integrated, opening up new avenues for early screening and mechanism analysis of FTD [67, 68].

#### The main types and typical cases of FTD biomarkers

Nucleic acid markers are primarily based on genetic mutations and used for risk stratification, subtype identification, and etiological confirmation. The hexanucleotide repeat expansion in *C9orf72* is the most common genetic cause of familial FTD, accounting for 40% of familial cases. It causes TDP‐43 accumulation through the production of toxic dipeptide repeat proteins via RAN translation. It can be detected by long‐read sequencing or PCR and has been applied to genetic counseling [39, 60]. *MAPT* mutation leads to abnormal phosphorylation of tau protein and formation of neurofibrillary tangles, often associated with behavioral variant or nonfluent aphasia phenotypes, especially in early‐onset FTD [63]. *GRN* mutation results in progranulin haploinsufficiency, and plasma progranulin precursor level <15 ng/mL suggests the presence of this mutation, which is closely related to semantic variant and right temporal lobar variant [58, 59]. In addition, *TARDBP* and *FUS* genes underpin the diagnosis of rare FTD subtypes [69].

Among the protein markers, neurofilament light chain is a marker of neuronal axonal damage, and its levels are elevated across all FTD subtypes. Plasma or cerebrospinal fluid levels >25 pg/mL have been associated with rapid disease progression. When combined with MRI, the diagnostic accuracy can be increased to 89% [65]. TDP‐43 protein aggregates are the core pathological markers of the FTD‐TDP subtype. Using cerebrospinal fluid or skin biopsy in combination with immunoblotting and RT‐QuIC techniques achieves sensitivity and specificity of 89% and 98%, respectively. It is currently at the stage of clinical translation [61]. Poly (GP), as a specific biomarker for *C9orf72* mutations, shows an approximately 2.8‐fold increase in cerebrospinal fluid levels in symptomatic carriers and is expected to serve as a monitoring indicator for assessing the efficacy of antisense oligonucleotide therapy [70]. In addition, GFAP can assist in differentiating FTD from AD; phosphorylated tau181 is elevated in AD but does not change significantly in FTD, making it a key indicator for differential diagnosis between the two diseases [71].

Among the imaging biomarkers and digital biomarkers, [^18^F]‐flortaucipir tau PET can specifically image brain tau aggregates. The positive rate is 95% in *MAPT* mutation carriers and about 60% in sporadic FTD‐tau, which can predict the speed of cognitive decline [72]. [^18^F]‐FDG PET shows that the metabolic activity of the orbitofrontal cortex in patients with behavioral variant FTD decreased by 40%, while the metabolic activity of the left temporal lobe in patients with semantic variant FTD decreased by about 35%. The metabolic level was negatively correlated with plasma neurofilament light chain and has been widely used in clinical differentiation [65, 73]. Diffusion tensor imaging can evaluate the integrity of white matter fiber tracts. In patients with behavioral variant FTD, diffusion abnormalities are often seen in the fasciculus cuneofrontalis and corpus callosum; arterial spin labeling (ASL) effectively distinguishes FTD from early Alzheimer's disease through changes in cerebral perfusion [74].

In terms of other types of markers, the NLR, a systemic inflammation indicator, is elevated in FTD patients due to microglial cell activation. Patients with a ratio exceeding 3.5 exhibit a survival period that is 42% shorter. This metric is currently employed routinely for prognostic assessment [74]. MicroRNA‐124 plays a role in neurogenesis and the regulation of synaptic function. Its plasma level in FTD patients is diminished, and it presents an area under the curve of 0.87 when differentiating FTD from Alzheimer's disease. At present, this biomarker remains in the research phase [58].

#### Current challenges and future development trends

Research on FTD biomarkers still faces several bottlenecks. First, serum biomarkers such as neurofilament light chain and GFAP can also increase in AD and ALS, with insufficient specificity to limit their independent diagnostic value [58]; secondly, long‐read sequencing and RT‐QuIC technologies are complex and costly, making them difficult to be widely used at the grassroots level [69]; at the same time, most of the new biomarkers are still in the verification stage, lacking unified detection standards and reference thresholds [39]; finally, most existing studies are based on european populations, and the differences in biomarker performance among races remain unclear, limiting their global application [57].

Future research will focus on three major directions: precision, technology‐driven, and integration. In terms of precision, stratified biomarker combinations based on genetic background, pathological subtypes, and clinical phenotypes will be developed, such as the combined detection of polyglutamin proline and neurofilament light chain in *C9orf72* mutation carriers [39, 60]. In terms of technology‐driven, single‐cell proteomics, spatial transcriptome, and artificial intelligence will jointly promote the discovery of new biomarkers and multimodal data integration [68]; non‐invasive techniques such as fundus imaging and breath volatile organic compound analysis are expected to optimize early screening pathways [67]. In terms of integration and dynamic monitoring, wearable devices combined with liquid biopsy technology will enable real‐time tracking of disease progression [56]. In addition, warning biomarkers for asymptomatic mutation carriers, predictors of acute exacerbation, and targeted therapeutic monitoring tools will become research hotspots [59]. The establishment of cross‐ethnic cohorts and unified testing standards is key to promoting the clinical transformation and global application of FTD biomarkers.

### Major depressive disorder

MDD is a highly heterogeneous mental disorder characterized by persistent low mood and anhedonia, often accompanied by cognitive and autonomic dysfunction. With a lifetime prevalence of 17% and a trend toward earlier onset, MDD is a leading cause of disability worldwide, yet diagnosis remains reliant on subjective clinical scales [75, 76, 77, 78]. Current diagnostic methods primarily rely on subjective clinical symptom scales. This not only leads to potential confusion with anxiety disorders and bipolar affective disorders, but also hampers early identification, prognosis judgment, and prediction of treatment response [79]. Biomarkers, which are quantifiable indicators that objectively reflect pathological physiological processes, provide a crucial basis for the precise classification and treatment of depression [80]. The rapid advancement of molecular biology, multi‐omics technologies, and neuroimaging has expanded research on depression biomarkers from traditional inflammatory and hormone indicators to a new stage of multi‐dimensional and multimodal integration (Table S5).

#### The development process of MDD biomarkers

Research on depression biomarkers is inextricably linked to the advancement of life science technology and can be categorized into three key stages. Early studies focused on inflammatory responses and neuroendocrine abnormalities, uncovering significant peripheral inflammatory activation and dysfunction of the hypothalamic‐pituitary‐adrenal axis in patients with depression. Biomarkers such as C‐reactive protein, interleukin‐6, and cortisol garnered widespread attention [81, 82]. During this phase, traditional techniques like enzyme‐linked immunosorbent assay were predominantly utilized, establishing the critical roles of inflammation and stress in the pathogenesis of depression.

The second stage is the rapid development period of fluid and nucleic acid markers. With the popularization of single‐molecule array technology, real‐time fluorescent quantitative PCR, and other methods, new markers such as plasma neurofilament light chain, brain‐derived neurotrophic factor (BDNF), microRNA have been successively discovered [83]. The rise of metabolomics further reveals abnormalities in tryptophan dopamine metabolic pathway, among which the ratio of tryptophan/dopamine has become an important indicator reflecting the interaction between nervous system and immune system [76]. During this stage, multi molecular level research enriched the understanding of pathological mechanism of depression and laid a foundation for subtype differentiation.

Current research has entered a phase of multimodal integration and precision. The combined application of genomics, transcriptomics, and metabolomics in conjunction with artificial intelligence algorithms has enabled systematic screening and validation of biomarker [84]. Integrated analyses of neuroimaging and fluid‐based biomarkers have deepened the understanding of disease mechanisms, while exploration of novel biomarkers such as the endocannabinoid system and extracellular vesicles has opened new avenues for noninvasive diagnosis and targeted treatment monitoring [85].

#### The main types and typical cases of MDD biomarkers

Protein‐based markers are the most widely used type in depression research, covering multiple aspects such as neuroprotection, inflammatory response, and nerve damage [79]. BDNF, which is involved in neural plasticity, is significantly reduced in plasma/serum levels in depressed patients and increases after treatment, with its levels showing a positive correlation with treatment response. It is currently in clinical validation phase [86]. Neurofilament light chain, as a marker of axonal damage, increases particularly significantly in depressed patients with cognitive impairment. It can be detected by single‐molecule array technology to provide reference for disease assessment [87]. C‐reactive protein, as a classic indicator of systemic inflammation, is positively correlated with the severity of depression and has been used in research for prognostic exploration [82]. GFAP reflects astrocyte activation and is elevated in cerebrospinal fluid in patients, which helps to distinguish depression from other neurodegenerative diseases and is currently under investigation [84].

Among the hormone and neurotransmitter markers, hormonal and neurotransmitter dysregulations are among the core pathological features of depression [88]. Cortisol, the primary hormone secreted by the hypothalamic‐pituitary‐adrenal axis, exhibits a disrupted circadian rhythm that is strongly associated with impaired hippocampal neuroplasticity, making it useful for disease monitoring. Adrenocorticotropic hormone plays a role in the stress response by modulating cortisol release; its combined assessment with cortisol aids in differentiating depressive subtypes [79]. 5‐Hydroxytryptamine, a crucial neurotransmitter, shows decreased concentrations in the synaptic cleft, impacting the limbic system‐frontal cortex pathway's function and underpinning the mechanism of action for serotonin reuptake inhibitors [89]. Diminished melatonin secretion is intricately linked to sleep disturbances and represents a potential therapeutic target [89, 90].

Among the nucleic acid markers, nucleic acid‐based markers, including microRNAs and DNA methylation, offer molecular insights into the etiology of depression and its subtype differentiation [91]. Specifically, microRNA‐146a plays a role in neuroinflammation by modulating pro‐inflammatory factor expression. Its altered levels in peripheral blood exosomes can serve as predictors for treatment outcomes. Aberrant DNA methylation, notably that of the glucocorticoid receptor gene, disrupts HPA axis regulation and is linked to early risk factors [83]. Moreover, genetic polymorphisms in genes like *C9orf72* and *GRN* have shown associations with depression susceptibility, paving the way for novel approaches in genetic risk evaluation [92].

Metabolic biomarkers reflect the dysregulated metabolic pathways in patients with depression and have great potential for clinical application. The decreased ratio of tryptophan to kynurenine and the accumulation of quinolinic acid were associated with cognitive decline [89]. Abnormal levels of acylcarnitines, which reflect fatty acid metabolism disorders, can be used as indicators to differentiate metabolic subtypes [93]. Endogenous cannabinoids are involved in stress regulation and emotional processing, and changes in their levels are closely related to anxiety symptoms [75].

Among the inflammation‐related markers, the inflammatory response plays a crucial role in the onset of depression, with relevant markers including cytokines and cell ratios [81]. Interleukin‐6 is involved in the pathological process by affecting neurogenesis and HPA axis function [80]. The elevated level of tumor necrosis factor‐α (TNF‐α) is related to blood‐brain barrier disruption and neurotransmitter imbalance, providing new targets for refractory depression treatment [94]. NLR as a simple systemic inflammation indicator has been used in research for prognostic exploration [77].

#### Current challenges and future trends

Research into depression biomarkers continues to encounter numerous challenges. The specificity of these biomarkers is inadequate, as many exhibit abnormal results in other mental disorders as well [79]. Detection techniques are complex and costly, hindering their widespread adoption in grassroots medical institutions [95]. Clinical translation lags behind due to the absence of unified detection standards and reference thresholds [78]. Furthermore, the disease heterogeneity is pronounced, with significant variations in the expression profiles of biomarkers across different subtypes [76]. Additional limiting factors include insufficient sample size, racial differences, and comorbidity interference, which restrict the promotion and validation of these biomarkers [96].

Future research will continue to advance in the directions of precision, technology‐driven approaches, and multimodal integration [95]. In terms of precision, specific biomarker combinations based on genetic backgrounds and pathological subtypes will be developed [78]; in the aspect of technology‐driven, single‐cell sequencing, spatial transcriptomics and artificial intelligence will facilitate the discovery and verification of new biomarkers [97]; in the aspect of multimodal integration, serum biomarkers combined with neuroimaging, clinical scales and wearable device data will construct a more comprehensive evaluation system [85]. Meanwhile, studies on inflammatory pathway and endocannabinoid system biomarkers will provide key evidence for targeted therapy [81]. Enhancing cross‐ethnicity cohort studies and establishing standardized testing procedures will accelerate the clinical transformation and global application of biomarkers.

### Schizophrenia

Schizophrenia (SCZ) is a severe mental disorder with positive, negative, and cognitive symptoms, affecting approximately 1% of the population worldwide [98]. Diagnosis currently depends solely on clinical assessment, lacking objective biological markers, which complicates differential diagnosis and impedes early intervention [99]. Biomarkers are emerging as fundamental components for precision diagnosis and treatment in schizophrenia [100]. Advancements in multi‐omics technologies, neuroimaging, and molecular biology have expanded biomarker research from conventional inflammatory markers and neurophysiological measurements to novel approaches including non‐invasive matrix detection and peripheral vesicle nucleic acid analysis (Table S6) [101].

#### The development process of SCZ biomarkers

The development of biomarkers for schizophrenia has evolved in tandem with advancements in life science and technology, progressing through three primary stages. Early research mainly focused on inflammatory activation and aberrant neurophysiological functions, revealing that patients exhibited systemic inflammatory responses coupled with alterations in brain electrical activity. Consequently, C‐reactive protein and the auditory P300 potential emerged as representative biomarkers during this phase [102]. These insights were primarily derived from conventional techniques such as immunoturbidimetry and electroencephalography, underscoring the pivotal role of neuroimmune irregularities in the pathogenesis of schizophrenia [102].

The second stage witnessed the widespread application of multi‐omics technologies. With the popularization of techniques such as enzyme‐linked immunosorbent assay and real‐time fluorescent quantitative PCR, multiple markers in dimensions such as inflammatory factor profiles, neurotrophic factors, neurophysiological indicators, and metabolites were systematically validated [103]. Advancements in neuroimaging technology further revealed correlations between retinal structural changes and diseases, providing a new direction for noninvasive detection [104]. During this stage, research at the multimolecular level deepened the understanding of disease mechanisms and laid the foundation for clinical subtype differentiation.

Current research has entered a phase of multimodal integration and clinical translation. The cross‐fertilization of genomics, metabolomics, and neuroimaging with artificial intelligence algorithms has enabled systematic screening and validation of biomarkers [100]. Innovations in non‐invasive detection techniques and novel methods such as extracellular vesicle nucleic acid analysis have significantly enhanced the clinical applicability of biomarkers, providing new technical support for early diagnosis and individualized treatment [99, 101, 105].

#### The main types and typical cases of SCZ biomarkers

Among the inflammation‐related markers, inflammatory response plays a pivotal role in the pathogenesis of schizophrenia, with associated markers such as cytokines and acute‐phase proteins. C‐reactive protein is notably elevated during both the acute and stable phases of the disease and correlates closely with symptom severity. It has been routinely employed for prognostic evaluation [102]. Interleukin‐6 has been implicated in neurogenesis, potentially through its effects on microglial cell activity; its levels are markedly elevated in treatment‐resistant patients, offering a rationale for anti‐inflammatory adjuvant therapy [103]. TNF‐α contributes to blood‐brain barrier disruption, and its genetic polymorphisms are associated with therapeutic response, highlighting the potential of targeted interventions.

Protein‐based markers encompass various facets, including neuroprotection and neuronal damage. A reduction in BDNF levels is intricately linked to compromised neuroplasticity; the subsequent restoration of these levels post‐treatment serves as a valuable metric for gauging treatment efficacy [106]. An elevation in GFAP signifies the activation status of astrocytes, aiding in the differential diagnosis between schizophrenia and other neurological disorders [104]. The expression pattern of fatty acid binding protein 4 within hair follicles offers an innovative method for non‐invasive genetic evaluation [101].

Neurophysiological indicators directly reflect dysfunction in neural circuitry and have good clinical handleability. Abnormal MMN reflects N‐methyl‐d‐aspartate receptor impairment and is associated with the degree of cognitive impairment, which may serve as an exploratory indicator for therapy monitoring in research settings [98]. Abnormal 40‐Hz ASSR indicates dysregulation of gamma band neural oscillations that underlie core neural circuit dysfunction [100]. Retinal nerve fiber layer thinning correlates with disease severity and has both state and trait properties [107].

In terms of metabolites and trace elements, abnormal levels of metabolites and trace elements reflect metabolic pathway disorders in disease. As a coagonist of the N‐methyl‐d‐aspartate receptor, decreased d‐serine levels are associated with negative symptoms, and supplementation therapy can improve clinical symptoms [105]. The imbalance of copper/zinc ratios in hair reflects long‐term oxidative stress states and provides a reference for assessing disease progression [101]. An abnormal tryptophan/carnitine ratio is related to neurotoxic metabolite accumulation and is an important biological basis for cognitive impairment [108].

Nucleic acid‐based markers provide molecular evidence for the exploration of disease causes. MicroRNA‐146a is involved in the pathogenesis by regulating the expression of inflammatory factors, and its level changes can predict treatment response [99]. The increase in extracellular vesicle‐derived cell‐free DNA (cfDNA) reflects the degree of tissue damage and has important noninvasive diagnostic value [99]. Abnormal DNA methylation leads to dysregulation of the hypothalamic‐pituitary‐adrenal axis, which is closely related to early risk of disease [109].

#### Current challenges and future development trends

The research still faces multiple challenges. The disease heterogeneity is high, and the existing markers cannot cover different clinical subtypes [108]; the specificity is insufficient, as many indicators show abnormal results in other mental disorders [102]; detection technology is complex and difficult for grassroots medical institutions to popularize [99]; clinical transformation lags behind, and there is a lack of standardized detection system [98]; confounding factors have great interference and affect the accuracy of results [105]. In addition, problems such as poor sample representativeness and racial differences also limit the application and promotion of markers [107].

Future research will focus on three directions. Regarding precision, specific biomarker combinations will be developed based on genetic background and clinical subtypes [102]; regarding technology‐driven approaches, single‐cell sequencing, spatial transcriptomics, and artificial intelligence will facilitate the discovery of novel biomarkers and methodological innovations [107]; concerning integrated applications, multimodal data integration and the establishment of dynamic monitoring systems will enable comprehensive disease management throughout the entire course [98]. Concurrently, enhancing interdisciplinary collaboration, establishing standardized testing protocols, and promoting multicenter validation studies will accelerate the clinical translation and global implementation of biomarkers [101].

### Glioblastoma

Glioblastoma (GBM) is the most aggressive primary brain tumor, characterized by marked heterogeneity, invasiveness, and a high recurrence rate [110]. Despite maximal treatment, median survival remains only 14–16 months, highlighting the critical need for biomarkers to improve early detection and therapeutic guidance [111]. The current diagnosis mainly relies on imaging and tissue biopsy, which have limitations such as subjectivity, difficulty in early identification, and insufficient efficacy prediction capability [112]. Biomarkers, as indicators that can objectively quantify the biological behavior of tumors, play an important role in the early detection, type identification, efficacy evaluation, and prognosis judgment of GBM [113]. In recent years, molecular biology, liquid biopsy, and artificial intelligence technology have promoted the research of GBM biomarkers from single molecular indicators to a new stage of multi‐omics integration (Table S7) [114].

#### The development process of GBM biomarkers

The research history of GBM biomarkers has been closely aligned with technological advancements and can be categorized into three primary stages. Early studies focused on conventional pathological and biochemical markers, emphasizing glial cell activation and associated inflammatory responses. During this phase, GFAP and carcinoembryonic antigen (CEA) emerged as representative biomarkers [115]. Methodologies primarily encompassed immunohistochemistry and enzyme‐linked immunosorbent assay, establishing a preliminary correlation between glial cell activity and tumor progression [116].

With the widespread use of technologies such as next‐generation sequencing and real‐time fluorescent quantitative PCR, research has entered a period of rapid development driven by multiomics [117]. Genetic markers such as *IDH1/2* mutation, *MGMT* promoter methylation, and 1p/19q codeletion have been confirmed to be closely related to patient prognosis and treatment response [118]; protein markers such as BDNF and FABP4 and nucleic acid markers such as miR‐21 and *MALAT1* have also been systematically verified [119]. Meanwhile, advances in neuroimaging technology have revealed potential associations between retinal structural changes and tumor invasion, providing new perspectives for noninvasive assessment [120].

Current research has entered the stage of integrated application and clinical transformation. The combination of multi‐omics platform and liquid biopsy technology enables non‐invasive acquisition and dynamic monitoring of biomarkers [113]. Artificial intelligence further optimizes the construction of combined models of multiple biomarkers, which significantly improves the accuracy of diagnosis and prognosis judgment [121]; the discovery of new biomarkers such as EVs‐cfDNA and POFUT1 provides a new direction for eklarly screening and targeted therapy [122].

#### The main types and typical cases of GBM biomarkers

Among the molecular genetic markers, *IDH1/2* mutations are molecular markers of secondary GBM and are thought to contribute to tumor development through 2‐hydroxyglutarate accumulation and interference with histone demethylation. Mutant patients have a significantly better prognosis than wild‐type patients, and detection can be performed using next‐generation or Sanger sequencing [118]. *MGMT* promoter methylation enhances temozolomide chemotherapy sensitivity by silencing DNA repair mechanisms, extending the median survival of positive patients to 15–18 months [110]. The *BRAF* ^V600E^ mutation activates the MAPK/ERK signaling pathway, and combination therapy with its inhibitor achieves an objective response rate (ORR) of 27% [119]. The 1p/19q co‐deletion is rare in GBM but indicates a more favorable therapeutic response and prognosis [123].

Among the protein markers, the level of BDNF is associated with neurocognitive function, and its post‐treatment increase may reflect therapeutic response. GFAP δ demonstrates specific expression at the tumor invasion front, which may aid in research on surgical margin assessment [110]. Apolipoprotein E influences disease progression through regulation of lipid metabolism and the tumor immune microenvironment, with the *APOE4* isoform being linked to poorer clinical outcomes [124]. Syntenin‐1 may help maintain tumor stem cell characteristics, potentially through activation of the PI3K/AKT pathway, suggesting its inhibitors could represent a potential therapeutic strategy [125].

Among the nucleic acid markers, long non‐coding RNA *MALAT1* promotes angiogenesis and epithelial‐mesenchymal transition through epigenetic regulation, with plasma levels associated with risk of recurrence [111]. miR‐21 promotes tumor proliferation by inhibiting expression of tumor suppressor genes such as *PTEN*, while oligonucleotide therapies targeting miR‐21 show antitumor effects in preclinical models [123]. *LINC00467* upregulates *SOX9* by competitively binding to miR‐145, thereby enhancing the traits of cancer stem cells [126]. Extracellular vesicle‐derived cfDNA can harbor driver mutations such as *IDH1* and *EGFR*, providing a means for noninvasive monitoring of tumor burden and progression [113].

In terms of markers related to inflammation and metabolism, macrophage migration inhibitory factor promotes microenvironment remodeling and angiogenesis through NF‐κB pathway activation, with its inhibitor demonstrating enhanced therapeutic efficacy when combined with temozolomide [127]. As a key inflammatory mediator within the tumor microenvironment, IL‐6 correlates with treatment resistance and represents a critical target for combination therapy [128]. Elevated expression of glucose transporter 1 reflects active tumor glycolysis, indicating potential sensitivity to metabolism‐targeted therapeutic strategies [122]. Alterations in copper/zinc ratios within hair may provide a non‐invasive biomarker for assessing long‐term exposure and oxidative stress levels [119].

#### Current challenges and future development trends

Research on biomarkers for GBM continues to encounter significant challenges. The high degree of tumor heterogeneity and the substantial variation in mechanisms across molecular subtypes make it difficult for any single biomarker to provide a comprehensive representation [110]. Moreover, many candidate biomarkers lack disease specificity and may also be altered in other neurological conditions [127]. Technical and practical limitations further impede progress. Current detection methods are often complex, costly, and not readily applicable in routine clinical settings [127]. Additionally, the absence of standardized detection protocols and established cutoff values for novel biomarkers hinders their integration into standard diagnostic and therapeutic pathways [111]. Patient‐specific factors, including individual differences and comorbid conditions, further complicate the interpretation of biomarker results [119]. Finally, constraints such as limited sample availability, insufficient racial diversity in cohorts, and a lack of multicenter validation studies undermine the external validity and generalizability of proposed biomarkers [120].

Future research will focus on precision, technological integration, and system consolidation. Regarding precision, individualized biomarker combinations will be constructed based on molecular typing and genetic background [118]. Technologically, single‐cell sequencing, spatial transcriptomics, and artificial intelligence will jointly drive the discovery of new biomarkers and the modeling of multi‐omics data [112], while the optimization of liquid biopsy technology will promote the popularization of non‐invasive screening [113]. Concerning integration, the multi‐dimensional fusion of clinical, imaging, and molecular data will enable dynamic disease monitoring and real‐time assessment of treatment responses [114]. Concurrently, strengthening cross‐center cooperation and establishing a standardized testing system will accelerate the clinical translation and global application of biomarkers.

### Meningioma

Meningioma is the most common primary intracranial tumor, accounting for over 36% of cases; although 80% are benign (WHO grade I), the remaining 20% exhibit significant invasiveness and high recurrence risk [129, 130]. With its insidious onset and diagnosis relying largely on imaging and histopathology, biomarkers are urgently needed for molecular classification and prognostic prediction [131, 132]. Advances in genomics, epigenetics, and liquid biopsy technologies have led to a transition in meningioma biomarker research, from single molecular indicators toward a multi‐omics integrated approach (Table 8) [133].

#### The development process of meningioma biomarkers

The research process of meningioma biomarkers is inextricably linked to advancements in life science and technology, and can be categorized into three primary stages. Early studies mainly relied on traditional pathological and cytogenetic techniques, identifying genetic and molecular events such as the deletion of chromosome 22q and anomalous expression of GFAP. These findings established a preliminary correlation between genetic variations and tumor occurrence, thereby laying the groundwork for future investigations [134].

With the application of high‐throughput technologies such as next generation sequencing and methylation chip, research has entered a rapid development period driven by multi‐omics. The mutation of *NF2* gene was confirmed as a core genetic event with an incidence rate up to 60–70% in meningioma [131]; the discovery of non‐*NF2* related mutations such as *TRAF7*, *AKT1*, *KLF4* further revealed the molecular heterogeneity of meningioma [129]. In addition, epigenetic markers such as *H3K27me3* deletion and *MGMT* promoter methylation were confirmed to be related to patient prognosis [133]. RNA‐based markers such as miR‐219‐5p and *LINC00460* gradually entered the verification stage, expanding the dimension of marker research [135].

Current research has entered a phase of integrated application and clinical translation. Breakthroughs in liquid biopsy technology, particularly the analysis of plasma exosomes and (ctDNA), have propelled the development of non‐invasive biomarkers [129]. The establishment of a DNA methylation classification system allows for precise prediction of recurrence risk in meningiomas, demonstrating superior efficacy compared to the traditional WHO grading system [136]. Artificial intelligence algorithms further integrate multi‐omics data to construct highly accurate diagnostic and prognostic models [137]. Novel biomarkers such as TXNDC16 autoantibodies and EVs‐MMP‐9 offer promising avenues for early screening [130].

#### The main types and typical cases of meningioma biomarkers

Among the genetic mutation biomarkers, *NF2* gene mutation is the most common genetic alteration in meningiomas and is thought to contribute to tumor proliferation and invasion, potentially through loss of Merlin protein and subsequent activation of PI3K/AKT and Hippo signaling pathways. The recurrence rate within 2 years for patients with mutant was 16.77%, and this indicator has been used in clinical molecular classification and prognostic evaluation [138]. *TRAF7* mutations are often co‐mutated with *AKT1*/*KLF4* and involved in the regulation of NF‐κB pathway, more common in WHO grade I tumors, and the 5‐year survival rate of patients was over 90% [129]. *TERT* promoter mutation is an independent adverse prognostic marker regardless of WHO grade, and the median progression‐free survival of patients with mutant was only 10.1 months [136]. homozygous deletion of *CDKN2A/B* occurs in more than 50% of WHO grade III tumors, indicating a risk of early recurrence and providing a potential target for CDK4/6 inhibitor treatment [131].

Among the epigenetic biomarkers, the loss of H3K27me3 has been associated with malignant tumor progression, potentially through silencing of tumor suppressor genes. Its incidence reaches 37% in WHO grade III tumors and is significantly associated with recurrence risk [138]. *MGMT* promoter methylation enhances sensitivity to temozolomide chemotherapy, resulting in a 40% increased response rate among methylated patients [136]. *TIMP3* methylation correlates with tumor aggressiveness, showing an incidence exceeding 60% in high‐grade meningiomas [133]. The DNA methylation classification system shows association with patient outcomes in research cohorts, with the 5‐year survival rate approaching zero for the MC mal subtype [136].

Among the RNA biomarkers, miR‐*219‐5p* regulates stemness properties by modulating *SOX9* and its expression level is negatively correlated with tumor grade. It can be used as a potential non‐invasive monitoring marker [131]. *LINC00460* sponges miR*‐*539 to upregulate MMP‐9 expression, promoting tumor invasion. Patients with high expression have a three‐fold increased risk of metastasis [137]. Extracellular vesicle‐derived cfDNA can carry gene mutations such as *NF2* and *TERT* with detection sensitivity of 82% and predict recurrence 2–3 months earlier than imaging [129]. miR‐497 was significantly downregulated in WHO grade III tumors and closely related to patient prognosis [132].

Among the proteins and inflammation‐related biomarkers, PD‐L1 expression is linked to the effectiveness of immune checkpoint inhibitors (ICIs), with an ORR of 27% in patients exhibiting high expression levels [130]. The *APOE* gene polymorphism influences tumor behavior through lipid metabolism regulation, and notably, the *APOE4* subtype correlates with a poorer prognosis. IL‐6 fosters tumor angiogenesis and peritumoral edema formation, displaying a positive correlation with both tumor grade and recurrence risk [129]. Additionally, the TXNDC16 autoantibody demonstrates commendable diagnostic attributes, boasting a sensitivity of 83.7% and a specificity of 90% [132, 135].

#### Current challenges and future development trends

Research on meningioma biomarkers is still faced with many challenges. The tumor has a strong heterogeneity, and the biological behavior of different molecular subtypes varies greatly [136]; some biomarkers lack disease specificity; detection techniques are demanding and expensive, limiting their clinical application [129]; there is a lack of standardized detection procedures and verification systems for new biomarkers [137]; most studies are based on single‐center retrospective data, which causes sample bias [131]. In addition, the complexity of the tumor microenvironment and the influence of treatment measures on biomarker levels also increase the difficulty of research [130].

Future research will focus on refining classification systems, fostering technological innovation, and achieving multimodal integration. Classification systems based on molecular characteristics will be further refined to enable individualized treatment strategies [131]. Emerging technologies such as single‐cell sequencing and spatial transcriptomics are expected to identify novel biomarkers, while artificial intelligence algorithms will facilitate the deep integration and model optimization of multi‐omics data [135]. Advancements in liquid biopsy technology will promote the widespread adoption of non‐invasive screening [132]. The establishment of a dynamic monitoring system that integrates clinical, imaging, and molecular characteristics holds promise for the real‐time assessment of disease progression [130]. Concurrently, enhancing multi‐center collaboration and establishing standardized testing protocols will accelerate the clinical translation and global application of these biomarkers [133].

### Multiple sclerosis

Multiple Sclerosis (MS) is an autoimmune disease driven by chronic inflammatory demyelination and neurodegeneration, and is a leading cause of non‐traumatic neurological disability in young adults. Diagnosis and management are complicated by its clinical heterogeneity, making biomarkers essential for early differentiation and treatment monitoring [139]. MS is highly heterogeneous, and its major clinical subtypes include relapsing‐remitting type (RRMS, accounting for 85%), secondary progressive type (SPMS), primary progressive type (PPMS, accounting for 10–15%), as well as pre‐clinical stages such as clinical isolated syndrome (CIS) and radiologically isolated syndrome (RIS) (Table S9) [139].

#### The development process of MS biomarkers

The evolution of MS biomarkers, in step with the development of detection technologies and a deeper understanding of disease mechanisms, can be divided into three stages. The early stage laid the foundation for traditional biomarkers, centered on cerebrospinal fluid oligoclonal bands (OCBs) and neurofilament light chain (NfL), initially establishing the diagnostic and monitoring system for MS [139, 140]. OCBs, as specific markers of intrathecal immunoglobulin synthesis, are key to diagnosing MS, and negative results significantly reduce the risk of conversion to clinically definite MS diagnosis [140]; NfL, an indicator of axonal injury, was primarily detected in cerebrospinal fluid and its levels were closely associated with disease activity [141]. Additionally, hormone level assessments and routine MRI lesion evaluations provided a preliminary basis for disease classification [139].

The middle stage was characterized by breakthroughs in omics and imaging technologies. With the development of immunohistochemistry and multi‐omics techniques, biomarker research has entered a precision era. Transcription factors aided subtype identification; noncoding RNAs have become a research hotspot, with decreased serum exosomal miR‐126 levels linked to blood‐brain barrier damage, while circRNA is involved in inflammation regulation; epigenetic marks such as *MEG3* methylation are associated with disease progression [142]. In terms of imaging, the discovery of MRI markers such as paramagnetic rim lesions (PRL) and slowly expanding lesions (SEL) provided new tools for assessing chronic inflammation and neuroaxonal damage [143].

The contemporary phase is characterized by the integration of multi‐dimensional biomarkers, with liquid biopsy and molecular imaging propelling the transition of these biomarkers into clinical practice. NfL, when detected using the SIMOA technology, offers high sensitivity and has emerged as a pivotal non‐invasive monitoring tool [144, 145, 146]. Biomarkers derived from gut microbiota, notably the reduced abundance of *Akkermansia muciniphila*, exhibit significant associations with immune dysregulation. Moreover, short‐chain fatty acid (SCFA) levels show an inverse relationship with disease activity [147]. TSPO‐PET imaging facilitates the visualization of microglial activation, allowing for a quantitative assessment of chronic inflammation [139]. Additionally, exosomal proteins, such as MMP1, and circulating tumor DNA (ctDNA) present promising avenues for early diagnosis and real‐time monitoring [142].

#### The main types and typical cases of MS biomarkers

Diagnostic biomarkers are used for the classification, early screening, and differential diagnosis of MS. Among traditional markers, cerebrospinal fluid OCBs have a diagnostic sensitivity of up to 85%, and combining with the kappa free light chain index can improve the diagnostic efficacy [139]; serum MOG‐IgG could effectively distinguish MOG antibody associated diseases from MS by cell‐based assays [140]; AQP4‐IgG is a specific marker for neuromyelitis optica spectrum disorder (NMOSD), and positive results could rule out MS. Regarding imaging markers, PRL have a specificity of over 90% on 3T MRI and could differentiate MS from other neuroimmune diseases [143]; slowly expansive lesions require confirmation by multiple longitudinal MRIs and have significant predictive value for the transition from RIS to clinically definite MS [139]. Among molecular markers, serum NfL has an AUC of 0.82 in diagnosing RRMS and could improve the prediction accuracy of CIS‐to‐MS conversion when combined with GFAP [144]; HLA‐DRB1*15:01 allele is the most important genetic susceptibility factor in European populations [139].

Prognostic assessment biomarkers are used to predict the aggressiveness of the disease, relapse risk and long‐term outcomes. Among protein biomarkers, chitinase 3‐like protein 1 (CHI3L1) was significantly higher in PPMS than in RRMS and could predict the rate of disease progression and positively correlated with the degree of GM atrophy [139]; GFAP is associated with astrocyte activation, and elevated serum levels have been correlated with chronic active lesions and disability progression [142]. Regarding imaging and genetic markers, combined assessment of SEL and PRL can comprehensively reflect the activity of chronic lesions and predict the risk of disability progression over 9 years [3, 143]; *Ki‐67* proliferation index 3% suggests high‐risk RRMS, and the risk of recurrence is significantly increased; HLA‐DRB1*15:01 positive patients have an increased risk of progressing from RRMS to SPMS [139]. In terms of gut microbiota and metabolic markers, *Akkermansia muciniphila* abundance was decreased and associated with Th17/Treg immune imbalance, and patients lacking it had an increased 5‐year relapse rate; serum SCFA levels were negatively correlated with neurodegeneration severity [147].

Biomarkers for therapeutic effect monitoring are used to assess treatment response in real‐time and guide adjustment of the therapeutic regimen. Among core markers, serum NfL reduction by ≥30% within 3–6 months after effective therapy indicates that disease activity is under control; its persistent increase warns about relapses and disability progression [144, 148]; GFAP levels decrease in response to anti‐inflammatory treatment and may be explored as a marker for neuroprotective therapy research [142]. Regarding imaging and immunological markers, PRL count declines and SEL expansion rate slows down in RRMS patients following therapy, suggesting relief from chronic inflammation [139, 143]; OCB normalization or titer reduction correlates with immunomodulatory treatment efficacy [140]; return of peripheral blood Th17/Treg ratio to normal level positively correlates with disease stability. Among emerging markers, exosomal miR*‐*126 level increases by 15% upon effective treatment, which relates to blood‐brain barrier repair [142]; *Akkermansia muciniphila* abundance returns to healthy levels, indicating effectiveness of probiotic or dietary intervention [147].

Among the imaging biomarkers and digital biomarkers, the development of multi‑marker combined models integrating serum NfL, MRI‑PRL, gut microbiota, and immune indicators is proposed to increase early diagnostic accuracy to 92%. Artificial intelligence is also envisioned to integrate radiomics and biomarker data to improve lesion detection efficiency [149].

#### Current challenges and future development trends

MS biomarker research still faces multiple challenges, particularly a lack of specificity. For example, CRP levels can also increase during infections and other autoimmune diseases. Moreover, when the MOG‑IgG titer is low, it is necessary to combine clinical and MRI features to achieve an accurate differentiation [140]. Detection standardization remains poor. For instance, MRI assessments of PRL and SEL rely on high‑field strength equipment, and variations in laboratory procedures often result in inconsistent findings. Disease heterogeneity is strong. For example, the expression profiles of biomarkers vary significantly among different MS subtypes. Higher levels of CHI3L1 are observed in PPMS than in RRMS, and no universal indicators have been identified [139]. And marker sensitivity during the prodromal phase (RIS/CIS) is insufficient, making ultra‐early intervention difficult to achieve [150].

Future research should focus on several key areas. First, the development of a multi‐marker combined model that integrates serum NfL, MRI‐PRL, gut microbiota, and immune indicators aims to increase the accuracy of early diagnosis to 92% while reducing prognostic prediction error [139, 143, 147]. Second, new biomarkers must be identified to expand the scope of liquid biopsy targets; potential candidates include lipofuscin as an intersectional indicator of oxidative stress and neurodegeneration, exosomal circRNA, and ctDNA methylation sites [142]. Third, personalized intervention strategies should be promoted, involving the formulation of precise treatment plans based on subtypes, genetic background, and gut microbiota characteristics, such as dietary fiber supplementation to elevate SCFA levels or the application of microglia‐targeting immunomodulators [139, 147]. Finally, technological innovation and clinical translation should be advanced by using artificial intelligence to integrate radiomics and biomarker data, thereby improving lesion detection efficiency, and by enabling the synchronous and rapid detection of NfL, MOG‐IgG, and AQP4‐IgG via microfluidic chips to facilitate the establishment of bedside diagnostic systems [140, 144].

### Epilepsy

Epilepsy affects approximately 70 million people worldwide, with a prevalence of 1−2% and increased rates among both the young and the elderly [151, 152]. Characterized by recurrent unprovoked seizures and often accompanied by cognitive and psychiatric comorbidities, epilepsy imposes substantial socioeconomic burdens, underscoring the need for biomarkers to improve early detection, subtyping, and therapeutic monitoring [153, 154]. In recent years, advances in genomics, epigenetics, and liquid biopsy technologies have shifted biomarker research from the investigation of single‐parameter indicators toward integrated multi‐omics analyses (Table S10) [155].

#### The development process of epilepsy biomarkers

The research process of epilepsy biomarkers is highly consistent with the profound development of life science and technology. It can be roughly divided into three key stages. The early exploration stage mainly focused on traditional electrophysiological and biochemical indicators. Electroencephalogram (EEG) patterns such as 3 Hz spike‐and‐wave complexes and periodic discharges (PEDs) became core diagnostic indicators for absence epilepsy and status epilepticus [156]. At the same time, it was initially discovered that serum S100B protein was related to brain injury, laying a foundation for subsequent molecular marker research [157]. This stage primarily relied on EEG monitoring and basic biochemical testing, employing relatively simple technologies [158].

The second stage was the rapid discovery period of molecular markers. With the application of technologies such as NGS and enzyme‐linked immunosorbent assay (ELISA), a large number of molecular level markers were identified [152]. Inflammation‐related markers, such as IL‐1β, IL‐6, and HMGB1, have been confirmed to participate in the occurrence and development of epilepsy [154]; autoantibodies related to autoimmune epilepsy, such as LGI1 antibody and GABA‐B receptor antibody, were successfully identified [151]; genetic mutation markers, such as *BRAF* ^V600E^ and *IDH1 R132H*, which play roles in brain tumor‐associated epilepsy (BTRE), were clarified [151]; abnormal expressions of microRNAs (miRNAs), such as miR*‐*128 and miR‐146a, also entered the verification stage [159]. At the same time, prognostic‐related epigenetic markers, such as *H3K27me3* deletion and *MGMT* promoter methylation, were revealed [152].

In the third stage, the multi‑omics integration and clinical translation phase, breakthroughs in liquid biopsy technologies including plasma exosome vesicles, ctDNA, and saliva testing have driven the development of noninvasive biomarkers [160]. The integrated application of multi‐omics technology (genomics, transcriptomics, metabolomics) has revealed the complex molecular network underlying epileptogenesis [151]; artificial intelligence algorithms integrating EEG and molecular data have improved the accuracy of diagnostic and prognostic models [156]; novel biomarkers such as P2X7R and abnormal abundance of intestinal flora provide new directions for the diagnosis of drug‐resistant epilepsy and etiological diagnosis [161]; monitoring antiepileptic drug concentrations in saliva has become an innovative alternative to therapeutic drug monitoring (TDM) [160].

#### The main types and typical cases of epilepsy biomarkers

Genetic mutation markers are an important basis for the molecular classification of epilepsy, especially in brain tumor‐related epilepsy. The *BRAF* ^V600E^ mutation is thought to activate the MAPK/mTOR pathway and may contribute to both neuronal epileptogenesis and glial cell tumor proliferation. Tumor tissue detection by NGS or ARMS‐PCR is currently under clinical validation. It is highly prevalent in low‐grade brain tumors in children, and *BRAF* inhibitors can inhibit both tumor growth and seizure occurrence [151]. *R132H* mutation results in abnormal isocitrate dehydrogenase function, excessive production of D‐2‐hydroxyglutaric acid (D2HG), and enhanced neuronal excitability. Tumor tissue detection by NGS or immunohistochemistry is currently under clinical validation. This mutation occurs in more than 70% of patients with low‐grade glioma, and preoperative epilepsy risk is three times higher in carriers than in wild‐type individuals [151]. *TERT* promoter mutations enhance telomerase activity and are independent adverse prognostic indicators regardless of WHO classification. Tumor tissue detection by pyrosequencing is currently under clinical validation. The median progression time of carriers is only 10 months [152]. The discovery of these markers provides a molecular basis for precision diagnosis and treatment of BTRE.

Among the epigenetic and RNA biomarkers, epigenetic and RNA‐based markers hold significant value in the pathogenesis and monitoring of epilepsy. The absence of H3K27me3, which promotes malignant progression by silencing tumor suppressor genes, has been found to increase in incidence with the grade of epilepsy‐related tumors, reaching 37% for grade III tumors. This absence also increases the risk of recurrence for patients by 1.7 times [153, 154]. The methylation of the *MGMT* promoter silences DNA repair genes and enhances sensitivity to temozolomide. In the verification stage of the study, the use of methylation‐specific PCR to detect tumor tissues revealed that the response rate of chemotherapy combined with anti‐epileptic treatment increased by 40% for patients positive for this methylation [152]. miR‐128, which is thought to target and regulate genes related to neuronal excitability such as *SOX9*, shows downregulation in association with hippocampal sclerosis and epilepsy recurrence. During the verification stage of the study, detection of plasma/cerebrospinal fluid using qRT‐PCR showed a positive correlation with the postoperative recurrence risk of patients with temporal lobe epilepsy (TLE), suggesting its potential use as a non‐invasive monitoring indicator [151]. miR‐146a, which regulates the TLR4/NF‐κB pathway, is upregulated, exacerbating neuroinflammation. A meta‐analysis conducted during the verification stage of the study, using qRT‐PCR to detect plasma/cerebrospinal fluid, revealed that the level in the cerebrospinal fluid of TLE patients increases by 1.8 times and is related to the severity of the disease [159].

Among the proteins and inflammation‐related biomarkers, proteins and inflammatory factors related to inflammation are involved in the regulation of neuroinflammation and pathological process of epilepsy. P2X7R, as an immune checkpoint‐related protein, activates NLRP3 inflammasome, promotes IL‐1β release, mediates ASMs efflux, resulting in drug resistance. The specific antagonist JNJ‐47965567 entered Phase II clinical trial, which can reverse drug resistance in some patients [161]. IL‐1β activates NF‐κB/MAPK pathway, enhances glutamate release in hippocampus, inhibits GABA receptor function, aggravates excessive neuronal excitation. ELISA or qPCR was used to detect serum/brain tissue in clinical verification stage. In clinical verification, cerebrospinal fluid level of refractory TLE patients is 2.1 times higher than that of sensitive patients. Caspase‐1 inhibitor could reduce seizure frequency [154]. IL‐6 activates JAK/STAT pathway, promotes neuroinflammation, reduces sensitivity of ASMs. ELISA or qPCR was used to detect serum/brain spinal fluid in clinical verification stage. In clinical verification, cerebrospinal fluid level of drug resistant epilepsy patients is 2.3 times higher than that of sensitive patients. IL‐6 receptor antagonist could improve seizures in some patients [154]. S100B participates in neuroinflammation and blood brain barrier destruction after activation by astrocytes, reflects the degree of brain injury. ELISA was used to detect serum in clinical verification stage. In clinical verification, peak level was reached within 24 h after seizure in TLE patients, positively correlated with the degree of hippocampal sclerosis [162].

Autoantibodies and electrophysiological pattern markers are important tools for diagnosing epilepsy. LGI1 antibodies target the neuronal surface LGI1 protein, disrupt the voltage‐gated potassium channel complex, and induce limbic encephalitis‐related epilepsy. They can be detected in serum or cerebrospinal fluid using indirect immunofluorescence or ELISA with a clinical specificity of 92% and sensitivity of 88%, and respond to immunoglobulin treatment at a rate exceeding 70% [151]. GABA‐B receptor antibodies bind to GABA‐B receptors, inhibit inhibitory neural transmission, and cause excessive excitation of neuronal networks. They can be detected in serum or cerebrospinal fluid using ELISA or cell‐based detection, and have been clinically validated as often being accompanied by limbic encephalitis and associated with refractory epilepsy [151]. The 3 Hz spike‐and‐wave complexes on electroencephalography result from abnormal synchronous discharges of the thalamocortical circuit and are the gold standard diagnostic indicator for absence epilepsy. Scalp video‐EEG monitoring is used, and it has been clinically applied. AI‐assisted analysis can increase recognition accuracy to over 95% [156, 163]. High‐frequency oscillations (HFOs, 80‐500 Hz) on electroencephalography are specific electrical activities of the epileptic focus that reflect abnormal synchronization of neurons. Long‐term intracranial or scalp EEG monitoring is used. intraoperative monitoring is under investigation and may improve accuracy in research settings and reduce postoperative recurrence rates [164].

Clinical imaging biomarkers are routinely used in epilepsy for noninvasive lesion detection, epileptogenic zone localization, and presurgical evaluation. Structural MRI identifies common structural causes including hippocampal sclerosis, cortical dysplasia, and tumors. Advanced MRI techniques, 18F‐FDG PET, and fMRI further characterize microstructural, metabolic, and functional abnormalities associated with epileptogenic networks [165], facilitating surgical planning and prognostic assessment [166].

#### Current challenges and future development trends

Research on epilepsy biomarkers still faces many difficulties. The disease is highly heterogeneous, with a wide variety of epilepsy subtypes, and the pathological mechanisms of different subtypes are quite different. A single biomarker is difficult to cover all populations [152, 167]; some biomarkers lack specificity, such as inflammatory indicators IL‐6 and CRP, which also increase in other neurological diseases and lack disease specificity [161]; detection techniques have limitations, NGS and methylation chip operation is complex and costly, it is difficult for grassroots medical institutions to carry out widely, and the detection standardization of some biomarkers is not high; clinical transformation lags behind, most new biomarkers lack unified detection standards and reference thresholds, and have not been included in routine diagnosis and treatment processes, such as liquid biopsy biomarkers in clinical application lack guidelines support [168]; sample bias exists, most studies are single center retrospective analysis, small sample size, lack of racial diversity, affecting result extrapolation; besides, the complexity of tumor microenvironment, treatment interference with biomarker levels, etc., also increase research difficulty [169].

Future research will advance toward precision, technology‐driven approaches, and multimodal integration. Regarding precision, stratified studies based on molecular subtypes are expected to become mainstream, exemplified by the analysis of marker differences between *BRAF* mutant and *IDH1* mutant in BTRE, which will enhance diagnostic accuracy and treatment response prediction [151, 168]. At the level of technological innovation, single‐cell sequencing and spatial transcriptomics are poised to uncover novel markers [170]; artificial intelligence algorithms that integrate genomic, epigenomic, and imaging data will facilitate the construction of multi‐dimensional assessment models [158]; and optimization of liquid biopsy technologies (such as saliva TDM and urine miRNA detection) will promote the widespread adoption of early screening [160]. Concerning multimodal integration and dynamic monitoring, efforts will focus on establishing risk scoring systems by combining clinical, imaging, and molecular data, alongside the development of markers for the dynamic monitoring of disease activity [152]; for targeted therapy monitoring, inflammatory markers will guide combination anti‐inflammatory treatments, while immune markers will predict the efficacy of ICIs [169]. Concurrently, strengthening multi‐center cohort studies and establishing unified detection standards and reference intervals will be crucial for promoting the global clinical application of these markers [151].

### Integration of common biomarkers for neurological diseases

Although neurological diseases have diverse clinical manifestations, they often share underlying pathophysiological pathways. Through systematic analysis of the biomarker profiles of major neurodegenerative disorders such as AD, PD, ALS, and MS, we identified a core network of shared biomarkers that are interconnected through complex regulatory relationships converging on central mechanisms, including aging, neuroinflammation, axonal damage, and protein misfolding.

Aging represents a predominant shared risk factor for multiple neurodegenerative disorders. Accumulating clinical and preclinical evidence supports the utility of combinatorial biomarker panels for risk stratification, early differential diagnosis, and prognostic evaluation of aging‐related conditions. In longitudinal cohorts of early PD patients, combined assessment of cerebrospinal fluid (CSF) α‑synuclein (αSyn), NfL, GFAP, and soluble TREM2 enables improved prediction of cognitive decline during disease progression [171]. Furthermore, a multiplex panel targeting synaptic and lysosomal dysfunction markers, such as VGF, neuronal pentraxin receptor (NPTXR), secretogranin‑2, and syntaxin‑7, effectively discriminates de novo PD and isolated REM sleep behavior disorder (iRBD) patients from healthy controls in long‑term follow‑up studies [172]. For cross‑disease differential diagnosis among aging‑related dementias, serum proteomic panels integrating NfL, phosphorylated tau (p‑tau) isoforms, *CD276*, and inflammatory signatures show robust performance in distinguishing AD, LBD, FTD, and progressive supranuclear palsy (PSP) [173]. In AD, machine learning‐derived CSF protein panels and plasma combinations of Aβ40/42, p‑tau181, p‑tau217, and NfL yield high diagnostic accuracy for identifying preclinical and overt AD, even across diverse detection platforms [174]. These combined biomarker panels offer several key advantages over single‐marker detection. They improve diagnostic accuracy, especially in the preclinical stages of aging‐related neurodegeneration, and enable better differentiation among phenotypically similar disorders. Multiplex strategies also support dynamic risk stratification and longitudinal monitoring, making them highly suitable for clinical trials and population‐level screening.

NfL is a critical component of the neuronal axonal cytoskeleton and is involved in maintaining axonal structural stability and signal transmission. Upon axonal injury or neuronal apoptosis, NfL is released in substantial quantities into both cerebrospinal fluid and blood. Given its high specificity and sensitivity, NfL has emerged as a pivotal dynamic biomarker for assessing the severity and progression of various neurological disorders [175]. In AD patients, serum NfL levels are significantly positively correlated with tau protein deposition and brain atrophy rates. Notably, these levels are abnormally elevated prior to the manifestation of clinical symptoms, suggesting their potential use in early screening [176]. In PD, elevated NfL levels are strongly correlated with the extent of damage to dopaminergic neurons in the substantia nigra, effectively predicting accelerated motor function decline and an increased risk of cognitive impairment [177]. In MS, NfL serves as a sensitive marker of disease activity and can predict treatment response [144]. This cross‐disease applicability positions NfL as a central biomarker bridging diverse neurological conditions, offering an objective, standardized quantitative measure for comparative mechanism studies, patient stratification, and clinical research through its representation of axonal damage as a common pathological endpoint.

GFAP is a specific intermediate filament protein of astrocytes and is considered a core biomarker of neuroinflammation, and its levels are associated with astrocyte activation and proliferation. Changes in its level in body fluids can accurately capture the temporal characteristics and intensity of the inflammatory response [178]. In the early stage of AD, the upregulation of GFAP expression even precedes Aβ peptide deposition, suggesting that astrocyte activation‐mediated neuroinflammation plays a promoting role in the initial stage of the disease [179]. In the acute phase of MS, GFAP levels are significantly correlated with blood–brain barrier disruption, active inflammatory lesions, and the extent of demyelination and can be used to quickly assess disease activity [180]. In ALS, dynamic changes in GFAP expression are synchronous with the rate of motor neuron damage and can effectively predict disease progression [181]. Notably, the expression of GFAP is not only elevated in patients with neurodegenerative diseases but also highly expressed in patients with acute brain injury and systemic inflammatory diseases, further confirming the universality of neuroinflammation as a common pathological basis of neurological diseases [182].

In addition to protein markers, at the molecular regulation level, the miRNA network is another important common regulatory mechanism. miRNAs are intimately involved in disease pathological processes through their ability to target and regulate gene expression [183, 184]. For example, miR‐146a, a core molecule involved in inflammatory feedback regulation, is abnormally expressed in AD, PD, and MS and is thought to inhibit the release of proinflammatory factors through negative regulation of the NF‐κB signaling pathway; imbalanced expression may contribute to the vicious cycle of neuroinflammation [185]. The expression of miR‐124, a neuron‐specific miRNA, is downregulated in various neurodegenerative diseases and can cause abnormal expression of genes related to synaptic plasticity, thus disrupting synapse formation and functional maintenance and aggravating neuronal damage [186, 187]. These shared miRNA regulatory networks reveal the profound commonality of neurological diseases at the epigenetic level.

In terms of the core pathological mechanism, protein misfolding and abnormal aggregation are other key common features of neurodegenerative diseases. Although abnormal proteins such as Aβ peptides, α‐synuclein, and TDP‐43 have disease‐specific distributions, they share highly similar prion‐like transmission characteristics; that is, they can induce conformational changes in normal homologous proteins, form aggregates, and spread between cells, thus gradually expanding the scope of pathological damage [188, 189]. Moreover, these abnormal proteins may activate the innate immune response, potentially involving the Toll‐like receptor 4 (TLR4) signaling pathway on microglia, which may initiate downstream NF‐κB cascade reactions and promote the release of proinflammatory factors such as interleukin‐1β (IL‐1β) and tumor necrosis factor‐α (TNF‐α), enhancing the cascade effect of neuroinflammation [190]. This common pattern of pathology across diseases provides clear targets for developing broad‐spectrum neuroprotective agents targeting protein folding regulation, abnormal protein transmission, or the TLR4 pathway.

From a clinical translational perspective, the aforementioned common markers complement each other to form a “biomarker matrix” for neurological diseases. Longitudinal dynamic monitoring of NfL can accurately track the process of axonal damage and quantify the rate of disease progression, providing an objective basis for evaluating intervention effects. Monitoring GFAP levels can reflect the activity of neuroinflammation in real time, which is helpful in differentiating acute inflammation from chronic persistent states, thus guiding the timing and intensity of anti‐inflammatory treatment, while detecting disease‐specific proteins such as Aβ and α‐synuclein provides a core basis for subtype identification and etiological diagnosis. This multidimensional integrated strategy not only improves the accuracy of early differential diagnosis but also provides guidance for individualized targeted therapy [179]. For example, persistently elevated NfL levels indicate rapid progression of nerve damage and may inform research directions for neuroprotective or anti‐inflammatory strategies (Table S11).

Mendelian randomization (MR) studies provide robust genetic evidence to clarify the causal relationships rather than mere correlations between biomarkers and aging‐related neurodegenerative diseases. For AD, two‐sample proteome‐wide MR confirmed the causal associations of plasma proteins, including *BLNK*, *CD2AP*, *GRN*, *PILRA*, and *PILRB*, with AD risk, independent of confounding factors [191]. Further mitochondrial‐related MR also verified DMPK and LACTB2 as causally linked to AD pathogenesis [192], supporting their roles as genuine pathological drivers rather than just diagnostic indicators. For PD, bidirectional MR demonstrated a causal inverse relationship between body mass index (BMI) and PD risk, with a genetic predisposition to a higher BMI associated with a lower PD risk [193]. Moreover, mitochondrial MR confirmed the causal involvement of NDUFAF2, BCKDK, MALSU1, and TTC19 in PD onset and progression [192], strengthening the causal relevance of mitochondrial dysfunction and metabolic imbalance in PD pathogenesis. These MR findings confirm that key biomarkers are not only associated with disease but also likely participate in causal pathological pathways, reinforcing their value as mechanistically meaningful targets for risk assessment and intervention.

Spatial omics technologies further refine our understanding of locally resident biomarkers by preserving their anatomical distribution and cellular microenvironment. High‐resolution spatial transcriptomics (CosMx and Stereo‐seq) has been used to characterize the amyloid plaque niche in AD, revealing region‐specific glial responses and locally enriched signaling networks, including plaque‐induced genes (PIGs), that reflect microglia–astrocyte crosstalk adjacent to Aβ deposits [194]. In MS, single‐cell spatial transcriptomic profiling has revealed pathogenic inflammatory niches in chronic active lesions, identifying locally accumulated CD8 + T cells and lipid‐laden dysfunctional microglia (GPNMB+ foamy microglia) as core locally resident signatures linked to sustained neuroinflammation and impaired remyelination [195]. These spatially resolved approaches enable precise mapping of region‐specific biomarker expression and improve the specificity of locally resident signatures for disease stratification.

Furthermore, racial and ethnic heterogeneity represents an important consideration for the generalizability of biomarker reference intervals. In AD patients, plasma concentrations of core biomarkers, including p‐tau181, p‐tau217, the Aβ42/40 ratio, and NfL, differ significantly across ancestral groups, including African, American, Hispanic, and non‐Hispanic white populations, even after adjustment for age, *APOEε4* status, and cognitive level [196]. Additionally, neighborhood socioeconomic disadvantage intersects with racial disparities and is associated with elevated levels of CSF YKL‐40 and tau, highlighting that social and demographic factors further modify biomarker profiles in underrepresented groups [197]. These findings emphasize that most current reference thresholds are derived from predominantly European ancestry cohorts and that validation across diverse racial and ethnic populations is essential to ensure the equitable and accurate clinical application of biomarker‐guided neurological assessment.

### Deep analysis of the nervous system as a hub of cross‐system networks

The nervous system establishes a complex bidirectional communication network with various bodily systems via multiple biological axes. This interplay allows biological markers of the nervous system to not only indicate brain disorders but also function as “sentinels” for overall health monitoring.

The gut–brain axis is the most extensively studied cross‐system communication pathway. As a central conduit in the nervous system, it facilitates bidirectional regulation between the gut and the brain through three interconnected pathways, namely, the neural, endocrine, and immune systems, and significantly affects neural homeostasis and disease progression [198]. The vagus nerve acts as a direct neural link, sensing mechanical stimuli from the gut and chemical signals such as bacterial metabolites and relaying them to the brainstem's solitary nucleus. This process modulates brain functions, influencing cognition, emotions, and movements of the higher cortex [199]. Clinical studies indicate that individuals with PD often suffer from chronic constipation for up to a decade prior to exhibiting motor symptoms. Their gut microbiota displays distinct imbalances, such as reduced *Prevotella* species and an overabundance of *Enterococcus*. Such dysbiosis is associated with compromised intestinal mucosal barrier function and increased intestinal permeability and may contribute to the misfolding and aggregation of α‐synuclein within the enteric nervous system. This aggregate may then propagate via the vagus nerve to the brain, potentially serving as a contributor to PD pathology [200, 201]. Within the endocrine pathway, SCFAs, notably butyric acid, derived from gut microbiota metabolism not only fortify the blood–brain barrier but also influence microglial maturation and function through epigenetic alterations [202]. In the immune system, lipopolysaccharides entering the bloodstream due to dysbiosis can initiate systemic inflammation, impair the blood–brain barrier, and enhance the penetration of inflammatory agents into the central nervous system, intensifying neuroinflammation and establishing a cross‐system pathological feedback loop [203].

The neuro‐immune axis constitutes another important interactive interface that cooperates with the gut–brain axis to support the cross‐system hub function of the nervous system. An imbalance in their bidirectional regulation is a key pathological link for neurological diseases. The central nervous system interacts with the immune system through multiple mechanisms: chemokine receptors on the surface of the blood–brain barrier selectively regulate the entry of immune cells into the brain; resident immune cells in the brain and microglia participate in neural network reconstruction by synaptic pruning and secrete cytokines to regulate neural functions bidirectionally; and abnormal antigens in the brain can leak to the periphery, activate specific immune responses, and form a central–peripheral immune link [204]. MS is a typical example of a disrupted neuro‑immune axis. Peripherally activated autoreactive T cells are thought to cross the blood–brain barrier, recognize myelin antigens, and contribute to inflammatory demyelinating damage [205]. Notably, this immune dysregulation is not unique to MS; abnormal activation of microglia and infiltration of peripheral immune cells have also been observed in AD and PD, suggesting that neuro‐immune axis imbalance is a common feature of neurodegenerative diseases [206].

The neurovascular axis establishes a communication bridge between the brain and systemic circulation through the unique structure of the blood–brain barrier. The blood–brain barrier is a dynamic interface with active regulatory functions. Transporters, tight junction proteins, and metabolic enzymes on endothelial cells work together to precisely regulate substance transport [207]. In the early stage of AD, the integrity of the blood–brain barrier is covertly undermined, resulting in abnormal leakage of plasma proteins such as fibrinogen into the brain, which activates microglia and amplifies neuroinflammation [208]. Conversely, brain‐derived Aβ peptides and tau protein can also leak out to the periphery through the damaged barrier [209]. This bidirectional material exchange deeply couples the nervous system with the circulatory system, explaining why vascular risk factors such as hypertension and diabetes increase the risk of neurodegenerative diseases by damaging the blood–brain barrier [210].

The neurometabolic axis works in conjunction with the aforementioned axes to form a finely tuned regulatory network through multiple hormones and metabolites, achieving dynamic energy coordination between the brain and peripheral organs. The hypothalamus, as the central hub for metabolic regulation, receives key metabolic signals such as leptin and insulin to regulate appetite and energy homeostasis [211, 212]. This regulation is often disrupted in neurodegenerative diseases: AD patients exhibit significant brain insulin resistance (so‐called “type 3 diabetes”), which is associated with increased Aβ deposition and tau phosphorylation and may contribute to accelerated cognitive decline; PD patients frequently experience progressive weight loss and metabolic disorders because of impaired hypothalamic function resulting from damage to substantia nigra neurons [213, 214]. Notably, peripheral metabolic markers can predict the rate of cognitive decline, suggesting that metabolic dysregulation is among the core drivers of neuropathological processes.

These cross‐system axes are intricately interwoven and work in concert to regulate each other, making the nervous system a core network hub that integrates physiological functions across the entire body. Clinically, abnormal neurobiomarkers often emerge before specific symptoms and have significant early warning value. For example, elevated serum NfL levels can be detected 10 years prior to the onset of cognitive symptoms in patients with familial AD; abnormal cardiac autonomic nerve function frequently precedes motor symptoms of PD [215, 216]. This “presymptomatic” characteristic establishes neurological biomarkers as sentinels for monitoring overall health. By dynamically tracking core biomarkers such as NfL and GFAP, it may be possible not only to identify risks for neurological diseases at an early stage but also to gain insight into the functional stability of cross‐system axes, potentially providing quantitative evidence for comprehensive health screening and supporting the transformation of medical models from “treating the ill” to “early detection and risk stratification for disease prevention,” fully demonstrating the clinical translational value of the nervous system as a cross‐system hub (Figure S1) [217, 218].

## CARDIOVASCULAR SYSTEM

Systematic literature searches were performed in the PubMed and Web of Science databases, employing Boolean logic with field‐restricted search strings targeting the title/abstract fields. The following Boolean queries were constructed for each target cardiovascular disease: for coronary heart disease and myocardial infarction, (“coronary disease” OR “myocardial infarction”) AND (“hs‐cTnT/I” OR “CK‐MB”) AND (“biomarkers”); for heart failure, (“heart failure”) AND (“BNP” OR “sST2” OR “galectin‐3”) AND (“biomarkers”); for hypertension, (“hypertension”) AND (“renin” OR “aldosterone” OR “endothelin‐1”) AND (“biomarkers”); for myocarditis, (“myocarditis”) AND (“cardiac autoantibodies” OR “inflammatory biomarkers”) AND (“biomarkers”); and for angiosarcoma, (“angiosarcoma”) AND (“TP53” OR “POT1” OR “MYC” OR “PTPRB” OR “NF1” OR “ARID1A”) AND (“biomarkers”). The retrieval period was defined as January 1, 2020 through December 31, 2025. Only articles published in English were considered. Both original research articles and systematic or narrative reviews were eligible for inclusion. A tiered screening strategy was subsequently implemented. Priority was assigned to high‐impact, highly cited review articles; the reference lists of these selected reviews were manually examined to retrieve additional high‐quality studies. Retrieved records were then screened by title and abstract, and articles deemed irrelevant to cardiovascular biomarker research, lacking full‐text availability, or containing unreliable data were excluded. The remaining full texts were then thoroughly re‐evaluated for final eligibility. The selected disease spectrum includes coronary heart disease, myocardial infarction, heart failure, hypertension, myocarditis, and angiosarcoma, all representing conditions with high prevalence, considerable disease burden, well‐established biomarker frameworks, and substantial clinical translational relevance.

### Coronary heart disease/myocardial infarction

Research on biomarkers, ranging from conventional enzymatic markers to contemporary molecules like non‐coding RNA and metabolites, has consistently propelled advancements in diagnostic and therapeutic approaches for coronary heart disease (Table S12) [219].

#### The development process of biomarkers

The research history of biomarkers for coronary heart disease can be divided into three key phases. The first phase was characterized by the era of traditional enzymology and protein‐based biomarkers, exemplified by markers such as creatine kinase isoenzymes and myoglobin [220]. During this period, diagnosis relied primarily on detecting enzymes or structural proteins released following myocardial injury. However, these early biomarkers were limited by relatively low specificity and insufficient sensitivity in the early stages of infarction [221]. With continued technological progress, cardiac troponin emerged as a pivotal advancement due to its superior myocardial tissue specificity, ultimately establishing it as the gold standard for diagnosing acute myocardial infarction and signifying the maturation of this initial developmental stage [222].

The second stage was the exploration period driven by multi‐omics technology [223]. The rise of proteomics, transcriptomics, and metabolomics technologies pushed research to shift from single biomarkers to systematic screening [224]. In this stage, new biomarkers such as microRNAs, trimethylamine N‐oxide, and apolipoprotein Eε4 emerged, which revealed pathological mechanisms of coronary heart disease in multiple dimensions, including inflammation, metabolism, and genetics [225]. For example, genes like *KLRD1* and *FOSL2* were found to provide new targets for plaque stability assessment [226].

The third stage was the omics integration and clinical translation period [227]. Current research focuses on multi‐dimensional data integration, using artificial intelligence, spatial omics, and large cohort validation to build accurate diagnostic and prognostic models that integrate traditional and novel biomarkers [228]. The development of biosensors further promotes the realization of bedside rapid detection, significantly accelerating the transformation of research results into clinical applications [219].

#### Main types and typical cases of biomarkers

Cardiac troponin serves as the primary indicator for diagnosing acute myocardial infarction [222]. High‐sensitivity detection methods can effectively screen for this condition within 3 h of symptom onset, boasting a notably high negative predictive value [229]. Creatine kinase isoenzymes act as supplementary markers, offering insights into the magnitude of the infarction. Myoglobin, due to its low molecular weight, elevates within an hour post the onset of acute myocardial infarction, making it a crucial tool for ultra‐early exclusion diagnosis [230]. Nonetheless, its use in tandem with cardiac troponin is essential to address its limited specificity. The cardiac‐type fatty acid binding protein is released 2–3 h earlier than cardiac troponin, bridging the gap in the ultra‐early diagnostic window [219].

Among the emerging biomarkers, heart‐specific non‐coding RNAs such as miR‐208a/b have demonstrated exceptional performance [221]. Notably, miR‐208a exhibits a detection rate of 100% within 4 h post onset and possesses remarkable specificity, positioning it as a promising early diagnostic indicator [231]. Additionally, Glycogen phosphorylase BB is promptly released during myocardial ischemia. Its diagnostic sensitivity surpasses that of myoglobin between 1 and 4 h after onset, offering a valuable alternative for challenging cases.

B‐type brain natriuretic peptide (BNP) and its precursor are induced by increased ventricular pressure load, accurately reflecting myocardial remodeling and the degree of heart dysfunction. As such, they have become core indicators for stratifying heart failure and assessing the risk of acute coronary syndrome [230]. The N‐terminal B‐type natriuretic peptide precursor exhibits superior stability, making it suitable for long‐term follow‐up [228]. CRP, an inflammatory marker, promotes plaque lipid deposition and exacerbates the atherosclerotic process. When combined with low‐density lipoprotein cholesterol, high‐sensitivity CRP can optimize the risk stratification model. Lipoprotein‐associated phospholipase A2 specifically reflects vascular wall inflammation and can predict plaque stability, suggesting its potential inclusion in screening systems for high‐risk populations [232].

The core pathological mechanism of coronary heart disease/myocardial infarction is the formation and rupture of atherosclerotic plaques, and the inflammatory response plays a crucial role in this process. Mendelian randomization studies provide strong evidence for the causal relationship between hs‐CRP and coronary heart disease. By selecting genetic variations that regulate the expression of hs‐CRP as instrumental variables to eliminate the interference of confounding factors such as smoking and high blood lipids, the study confirmed that the increase in hs‐CRP is not a secondary inflammatory stress response of coronary heart disease, but a causal factor driving the progression of atherosclerosis. This conclusion is consistent with the pathological mechanism mentioned in the document that hs‐CRP “promotes lipid deposition in plaques and accelerates the process of atherosclerosis,” and Mendelian randomization further clarifies the causal nature of this association rather than a simple correlation, providing core evidence for using hs‐CRP as a risk stratification and anti‐inflammatory treatment target for coronary heart disease.

Among metabolite markers, trimethylamine‐N‐oxide has been shown to accelerate atherosclerosis by promoting macrophage foaming. Notably, the level of this marker increases by 62% when it exceeds 5 μmol/L [225]. Furthermore, alterations in the expression of KLRD1, LILRB3, and FOSL2 serve as effective predictors for plaque progression and recurrence risk. The combined detection of these three markers yields an area under the curve of 0.938 [226].

Apolipoprotein Eε4 plays a crucial role in maintaining cardiovascular homeostasis by modulating vascular dilation and sodium–water excretion. This protein is significantly associated with the genetic predisposition to coronary heart disease. Additionally, the Inflammatory‐related gene *SOCS3* contributes to plaque progression through its regulation of platelet activity and inflammatory response. The expression level of *SOCS3* correlates with the severity of the disease [226].

Lipid metabolism markers, such as lysophosphatidylcholine and ceramides, contribute to the progression of atherosclerosis by facilitating lipid peroxidation and impairing endothelial function. Notably, specific ceramide profiles can effectively predict the risk of cardiovascular death, myocardial infarction, and stroke within a year [230]. Acylcarnitine, an intermediate product of fatty acid metabolism, exhibits abnormal levels that reflect disturbances in myocardial energy metabolism and are closely associated with the onset and progression of coronary heart disease [220].

While conventional and omics‐derived circulating biomarkers provide a systemic view of coronary artery disease, they often lack resolution regarding the localized pathological events within the atherosclerotic plaque. Emerging spatial omics technologies, including spatial transcriptomics and proteomics, have enabled the precise mapping of local resident markers within distinct plaque regions. For instance, heart‐type fatty acid binding protein (h‐FABP) exhibits localized high expression specifically within the damaged cardiomyocytes of the ischemic area, demonstrating spatial consistency with the extent of myocardial necrosis rather than merely reflecting global ischemia. Furthermore, the integration of Mendelian randomization studies has robustly established the causal relationship between inflammatory markers, specifically high sensitivity C reactive protein (hs CRP) and lipoprotein‐associated phospholipase A2 (Lp PLA2), and the progression of coronary heart disease. Spatial omics complements this causal inference by revealing that the aberrant activation and spatial distribution of these local resident markers are direct drivers of plaque instability and subsequent myocardial injury. This convergence of spatial biology and causal genetics provides refined molecular targets for developing therapeutic strategies aimed at modulating local plaque inflammation and enhancing myocardial protection.

Emerging evidence underscores that the diagnostic and prognostic performance of myocardial infarction biomarkers exhibits significant sex based heterogeneity, particularly regarding optimal threshold definitions. A prospective investigation involving 1941 patients with suspected acute coronary syndrome demonstrated that the application of sex specific high sensitivity cardiac troponin I thresholds, defined as greater than 16 ng/L for women and greater than 34 ng/L for men, yielded a 30% relative increase in the diagnosis of type 1 myocardial infarction among women compared with a uniform threshold approach, while the increment in men was merely 4.9%. Notably, among women reclassified by the sex specific cutoff, 74% presented with typical ischemic symptoms, including chest pain with radiation to the left arm or back and associated nausea, a clinical profile highly concordant with that of initially diagnosed patients. From a quantitative perspective, both baseline median and peak median concentrations of high‐sensitivity cardiac troponin I are substantially lower in women than in men with confirmed type 1 myocardial infarction, yet the predictive value and prevalence of typical symptomatology remain higher in female patients. The incidence of typical presenting symptoms reached 77% in women with a positive likelihood ratio of 1.18, compared with 59% in men with a positive likelihood ratio of 1.09. After adjustment for age and comorbidities, each incremental typical symptom conferred a graded increase in the odds ratio for myocardial infarction diagnosis in women (two symptoms: 4.0; three symptoms: 5.9; four symptoms: 6.9), whereas no significant association was observed between symptom count and diagnostic likelihood in men. These findings collectively indicate that biomarker expression profiles and their clinical correlates exhibit pronounced sex dimorphism. Adoption of sex specific diagnostic thresholds, in conjunction with symptom cluster characterization, substantially enhances diagnostic accuracy and provides a foundation for individualized management strategies across diverse patient populations.

#### Current challenges and future trends

Research on biomarkers for coronary heart disease continues to face numerous challenges. The disease's strong heterogeneity makes it difficult for a single biomarker to comprehensively reflect the pathological characteristics of different clinical phenotypes [233]; specificity is lacking, as many inflammatory biomarkers also increase in cases of infection and other conditions [229]; clinical translation proves challenging due to the lack of large‐scale validation and standardized detection protocols for new biomarkers; and the efficacy of early diagnosis is limited, as existing indicators fail to identify high‐risk populations before significant organ function impairment occurs [234].

Future research will advance in the directions of precision, technology‐driven approaches, and integration. Precision medicine will see a shift from the “one‐size‐fits‐all” approach to molecular classification based on biomarker profiles [235]; Technologically, multi‐omics integration and artificial intelligence will refine the optimization of biomarker combination models. Additionally, non‐invasive detection technologies, such as breath volatile organic compound analysis, are expected to broaden their application scope. The emphasis will be on integration and dynamic monitoring, leading to the construction of a comprehensive assessment system that combines clinical, imaging, and molecular data [225]. Central to future research will be the development of biomarkers for early screening, acute risk prediction, and guidance for targeted treatments [231].

### Heart failure

Heart failure is a clinical syndrome characterized by an abnormal structure or function of the heart, primarily marked by impaired ventricular filling or ejection capacity. Its primary clinical manifestations encompass dyspnea, fatigue, and fluid retention [236]. Biomarkers, which offer quantifiable insights into pathological and physiological states, are indispensable in the early detection, risk assessment, prognostic evaluation, and therapeutic guidance for heart failure [237]. Research into these biomarkers, ranging from traditional natriuretic peptides to novel inflammatory, fibrotic, genetic, vocal, and metabolomic indicators, significantly aids in refining diagnostic and therapeutic precision (Table S13) [238].

#### The development process of biomarkers

The initial phase was characterized by the use of traditional biomarkers and imaging assistance. Research on cardiac failure biomarkers primarily focused on evaluating clinical symptoms and basic physiological indicators. The 1990s witnessed a significant breakthrough with the discovery of B‐type BNP and its N‐terminal precursor (NT‐proBNP). These became the first blood biomarkers widely used in diagnosing heart failure, particularly in elderly patients, aiding in distinguishing cardiac dyspnea from respiratory causes [236, 237]. During this period, research predominantly centered on single molecular indicators, complemented by imaging techniques like echocardiography, which successfully differentiated heart failure from other diseases [236]. Concurrently, cardiac troponin, indicative of myocardial injury, was progressively incorporated into prognostic assessment systems, facilitating the early identification of high‐risk patients [237]. Inflammatory biomarkers, notably CRP, also garnered attention during this phase, setting the stage for further exploration into inflammatory mechanisms in heart failure [239]. The hs‐CRP is a protein biomarker that reflects low‐level chronic inflammation throughout the body. It is mainly used for assessing the risk of atherosclerosis and judging the prognosis of cardiovascular diseases. As a cardiovascular risk prediction marker, hs‐CRP was approved by the US FDA in 2003 for evaluating the risk of coronary heart disease and stroke in asymptomatic individuals. Since then, it has been recognized by multiple regulatory agencies and authoritative guidelines in various countries and can be used for prognostic stratification of patients with heart failure, indicating the deterioration of cardiac function driven by inflammation and the risk of adverse events. It is a widely used cardiovascular inflammation biomarker in clinical practice.

Subsequently, the field entered a phase propelled by multi‐omics technologies. As techniques such as proteomics, transcriptomics, metabolomics, and genomics advanced, heart failure biomarker research transitioned into systematic screening [238]. Proteomics has identified molecules such as soluble ST2 (sST2) and galectin‐3 (Gal‐3), both of which are closely associated with myocardial fibrosis. For example, sST2 inhibits the cardioprotective IL‐33/ST2L pathway, thus exacerbating fibrosis [240]. Transcriptomics unveiled miRNAs including miR‐132, miR‐503‐5p, and miR‐4649‐3p, offering novel targets for subtype categorization and prognostic predictions [240, 241]. Metabolomics facilitated the development of a cardiac lipid profile and identified ceramides as prospective biomarkers, facilitating the detection of early asymptomatic heart failure [242]. Genomic studies elucidated the significance of genetic biomarkers and polygenic risk scores in predisposing to and advancing heart failure, particularly in familial cardiomyopathies [243]. The integration of multi‐omics methodologies has substantially broadened the spectrum of biomarkers and enhanced understanding of the pathological mechanisms underlying heart failure, including those related to inflammation, fibrosis, metabolism, and genetics [243].

The third stage is the omics integration and clinical translation. Current studies are moving toward a new stage of multi‐omics integration and clinical translation [244]. By integrating data from multiple dimensions, such as proteomics, transcriptome, metabolomics, genomics, and using artificial intelligence algorithms and large sample cohort verification, composite biomarker models with better diagnostic and prognostic performance were constructed [237, 238]. For example, combining NT‐proBNP with high‐sensitivity cardiac troponin and sST2 can significantly optimize risk stratification in patients with heart failure with preserved ejection fraction (HFpEF) [236, 239]. The application of emerging technologies such as organoids and spatial omics further accelerated the process of clinical transformation of biomarkers [245]. Novel non‑invasive biomarkers include voice‐based markers such as maximum phonation time (MPT) and mel frequency cepstral coefficient (MFCC). These biomarkers can be collected via smartphones and have shown great potential for remote monitoring and congestion assessment, achieving an accuracy of up to 98% and offering a novel solution for home management [246]. The combination of biomarkers and targeted therapy, such as SGLT2 inhibitors regulating Gal‐3 levels and anticoagulants modulating miRNA‐related pathways, also provided molecular evidence for efficacy evaluation.

#### Main types and typical cases of biomarkers

Firstly, there are diagnostic and screening biomarkers that are primarily used for the early identification and differential diagnosis of heart failure. NT‐proBNP is currently the most widely used biomarker in clinical practice and is classified as a Class I recommendation by the ESC/AHA guidelines [236, 239]. Its cutoff value for ruling out heart failure is <300 pg/mL, with age adjustment required in acute settings. This indicator has a long half‐life and high plasma stability, making it more suitable for long‐term monitoring of chronic heart failure, especially in elderly patients [247]. Soluble *CD146*, as a specific marker of venous congestion, can improve diagnostic accuracy when NT‐proBNP falls within the “gray zone” and is unaffected by renal function, making it suitable for patients with chronic kidney disease (CKD) [237]. Inflammatory biomarkers, such as hs‐CRP and IL‐6, are closely associated with the incidence and progression of HFpEF. Mendelian randomization studies support the causal role of IL‐6 in the development of HFpEF. Meanwhile, hs‐CRP levels equal to or greater than 2 mg/L indicate a high residual inflammatory risk [248]. cfDNA reflects the extent of myocardial injury and provides auxiliary diagnostic value in acute heart failure, with its level positively correlated with cardiac troponin I and significant predictive value for 30‐day mortality. Specialized biomarkers such as ceramides and GlycA also show promise. Ceramides predict cardiovascular events by promoting lipid peroxidation, while GlycA correlates with subclinical myocardial dysfunction in type 1 diabetes [249]. Lung congestion biomarkers play an important role in the diagnosis of acute heart failure, aiding in the identification of pulmonary congestion and injury [246].

The second major category is prognostic evaluation biomarkers, which are mainly used to evaluate the risk of adverse outcomes such as death and rehospitalization in patients. sST2 inhibits myocardial protection pathways and promotes fibrosis; its level is not affected by age, renal function or BMI, has low biological variability, and admission >35 ng/mL suggests a significantly increased 1‐year mortality rate [239, 250]. Gal‐3 participates in the activation of the inflammation‐fibrosis axis and increases more significantly in HFpEF patients, with levels >17 ng/mL suggesting an increased 2‐year mortality risk [248]. miR‐132 promotes myocardial hypertrophy and remodeling, and when its level increases more than fivefold, the 1‐year mortality risk increases 2.9‐fold, and related antagonists have entered clinical trials [241]. Hemodynamic congestion biomarkers reflect the degree of volume overload and thereby enable effective prediction of rehospitalization risk.

Biomarkers of myocardial injury and fibrosis mainly reflect cardiomyocyte damage, fibrosis, and the extent of ventricular remodeling. High‐sensitivity cardiac troponin can detect minor myocardial injury, and a slight increase is associated with adverse outcomes in patients with chronic heart failure [249]. It improves risk stratification accuracy when combined with NT‐proBNP in patients with HFpEF. In ischemic heart failure, its level is significantly higher than that in nonischemic types, which helps to differentiate etiology [251]. Biomarkers related to the transforming growth factor‐β (TGF‐β) pathway mediate fibroblast activation and are involved in the process of myocardial fibrosis, closely related to disease progression [241, 243]. The cfDNA can be used to monitor donor‐derived DNA after heart transplantation and diagnose acute rejection episodes with sensitivity and specificity of 81% and 85%, respectively, significantly reducing the need for invasive biopsies [250].

Congestion and metabolism‐related biomarkers are primarily used to assess volume status and metabolic state, guiding decisions such as diuretic therapy. Carbohydrate antigen 125 (CA125) reflects the degree of extravascular congestion; >35 U/mL in acute heart failure indicates residual congestion, with a 2.1‐fold increased risk of rehospitalization within 6 months if not returned to normal. Mesocortisol precursor has a long half‐life and high stability, can effectively evaluate the state of vascular congestion, and has better prognostic value than BNP in patients with low BMI [252]. The cardiac lipid panel analyzes lipid components such as sphingomyelin and triglycerides to identify asymptomatic reduced ejection fraction heart failure patients 6‐12 months earlier than clinical symptoms [238].

In addition to molecular biomarkers, clinical imaging and quantitative digital thresholds play a critical role. Left ventricular ejection fraction detected by echocardiography serves as the key imaging marker for differentiating HFpEF from HFrEF and assessing ventricular remodeling. Cardiac magnetic resonance (CMR) provides supplementary evaluation of myocardial fibrosis and injury extent. Regarding digital markers, established clinical thresholds include NT‐proBNP <300 pg/mL for excluding heart failure, sST2 > 35 ng/mL indicating elevated short‐term mortality risk, Gal‐3 > 17 ng/mL suggesting increased long‐term mortality risk, and CA125 > 35 U/mL reflecting residual congestion in acute heart failure. The urine microalbumin/creatinine ratio (UACR) is utilized for prognostic assessment in cases with concurrent renal impairment. Furthermore, investigational digital markers such as MPT and MFCC can be non‐invasively collected via smartphones to distinguish congestion states with up to 98% accuracy, demonstrating significant potential for remote monitoring and clinical translation.

Traditional circulating biomarkers such as B type natriuretic peptide (BNP) and its N terminal precursor (NT proBNP) predominantly reflect systemic hemodynamic stress rather than the regional heterogeneity of myocardial pathology. Notably, BNP received FDA approval in 2000 as the first biomarker for differential diagnosis of acute heart failure, yet even this gold standard indicator cannot fully capture localized myocardial alterations. The application of spatial omics has begun to address this gap by identifying distinct regional signatures within the failing myocardium. Spatial metabolomics and transcriptomics have pinpointed abnormal local distributions of metabolites such as acylcarnitines, which cluster specifically in areas of myocardial contractile dysfunction and align spatially with regions of impaired energy metabolism. Complementing these findings, Mendelian randomization studies have substantiated a causal role for interleukin 6 (IL‐6) in the pathogenesis of HFpEF. Spatial omics further elucidates the region‐specific expression patterns of fibrosis‐related markers, including sST2 and galectin 3 (Gal 3), demonstrating a spatiotemporal correlation with the progression of ventricular remodeling. This integrated evidence underscores that the synergistic dysregulation of localized fibrosis and energy metabolism constitutes a core mechanism in heart failure progression, thereby offering a more refined and spatially informed basis for the development of targeted myocardial therapies.

The integration of continuous physiological monitoring with circulating biomarkers has led to a transition in heart failure management, from reactive approaches toward proactive early warning. In patients discharged following acute decompensated heart failure, the Corsano CardioWatch 287 2 wearable device enables continuous acquisition of multidimensional time series data including heart rate, activity level, and respiratory rate at a maximum sampling frequency of 1 Hz. These dynamic streams are integrated with NT proBNP levels, applying an abnormal threshold of ≥1000 pg/mL, and the clinical EVEREST congestion score. Through self supervised contrastive learning, a risk index model is constructed whereby heart rate fluctuations exceeding 15% and a daily activity decline of 50% or greater serve as real time alert signals. The model extracts baseline physiological features during periods of clinical stability and triggers an alert when real time feature dissimilarity exceeds a predefined risk threshold. This combined approach was validated within the CONAN study framework and demonstrated a sensitivity of 85% for identifying acute decompensation events within a 90‐day window, effectively capturing subclinical congestion prior to symptomatic deterioration. For elderly high‐risk patients aged over 70 years with comorbid hypertension and diabetes, long‐term monitoring with an implantable loop recorder provides complementary prognostic insight. The integration of continuous resting sinus heart rate data with baseline plasma NT proBNP, cardiac troponin T (cTnT), and BMI has enabled the construction of an acute decompensation early warning model. Specific combined thresholds include resting heart rate slowing below 62 beats per minute, NT proBNP exceeding 12 pmol/L, cTnT exceeding 12 ng/L, and BMI of 26 kg/m^2^ or higher. The LOOP study, encompassing 40 months of longitudinal surveillance, demonstrated that this multimarker model improved the area under the curve (AUC) for predicting heart failure decompensation secondary to atrial fibrillation to 0.79 compared with baseline models incorporating only demographic and comorbidity data. These approaches collectively illustrate the clinical utility of integrating wearable or implantable device data with traditional circulating biomarkers to enable proactive and personalized heart failure management.

#### Current challenges and future trends

Research on biomarkers for heart failure still faces many challenges [243]. The disease is highly heterogeneous, and the pathogenesis of different phenotypes, such as HFpEF and HFrEF, is not identical; genetic factors further increase this heterogeneity, making it difficult for a single biomarker to comprehensively reflect the condition of the disease [251]. A major issue is insufficient specificity. Most candidate biomarkers are systemic indicators that can also become abnormal in other diseases such as infection or tumor. For example, hs‐CRP levels rise in both heart failure and pneumonia [250]. Difficult clinical transformation, many potential biomarkers because of complex detection technology, high cost or lack of unified standards and other reasons, it is difficult to apply them to clinical routine. lack of early diagnostic biomarkers, existing indicators are mostly increased after the appearance of symptoms, it is difficult to achieve early identification of high‐risk groups [251].

Future research will advance in the directions of precision, technology‐driven discovery, and integration. Precision medicine will involve the development of individualized biomarker combinations based on patient phenotypes, comorbidities, genetic profiles, and demographic factors such as sex and age. For instance, sex‐specific miRNA panels have been shown to improve subtype classification accuracy [251], and cardiovascular biomarkers are increasingly utilized for precision diagnosis [237]. Technologically, advancements in multi‐omics technologies, single‐cell sequencing, artificial intelligence, and machine learning will facilitate the discovery and validation of novel biomarkers; AI models that integrate proteomics and clinical data have demonstrated a 15% improvement in prognostic accuracy compared to single biomarkers [237, 238]. Furthermore, the emergence of non‐invasive detection technologies, such as vocal biomarkers and breath volatile organic compound analysis, will broaden the scope of application scenarios [237]. The integration of clinical, imaging, and molecular data will enable the construction of comprehensive assessment systems, while wearable devices and non‐invasive techniques will allow for the real‐time monitoring of disease progression [237, 238]. Research will also be targeted toward early screening, the prediction of acute exacerbations, and companion diagnostics for targeted drug therapy [240, 241]. Exercise intervention, which modulates inflammatory biomarkers and enhances cardiac function, may emerge as a significant adjunctive therapeutic strategy [241, 253, 254]. Moreover, anticoagulation therapies guided by miRNA biomarkers could offer new therapeutic avenues for treating heart failure complicated by thrombosis [240]. Finally, a comprehensive quality analysis of both conventional and novel biomarkers is essential for further optimizing diagnostic and prognostic systems [248].

### Hypertension

Hypertension, the most prevalent chronic non‐communicable disease globally, is characterized by a consistently elevated arterial blood pressure. Its pathogenesis encompasses an interplay of various factors including genetics, environment, and metabolic disorders [255]. Traditional blood pressure measurement methods can diagnose hypertension, but they fall short in accurately assessing disease heterogeneity, predicting prognosis, or tailoring individualized treatment plans. In recent years, advancements in understanding pathological processes such as inflammation [256], disturbances in the renin–angiotensin–aldosterone system (RAAS) [257], and oxidative stress have propelled research in hypertension biomarkers. This has paved the way for innovative theoretical frameworks and practical tools for precise disease management (Table S14).

#### The development process of blood biomarkers for hypertension

The first stage was the era of traditional physiological and imaging indicators, during which early studies primarily focused on directly measurable physiological parameters and morphological changes. Blood pressure served as a diagnostic cornerstone; however, while it could identify hypertension, it failed to effectively predict prognosis [258]. Imaging techniques offered crucial supplementary information: echocardiography revealed left ventricular hypertrophy and thickening of the carotid intima‐media, indicative of early target organ damage due to hypertension [252, 255]; electrocardiograms were employed for an initial assessment of myocardial ischemia and electrophysiological abnormalities. Furthermore, conventional renal function markers such as serum creatinine and urea nitrogen could indirectly indicate renal damage associated with hypertension [257]. In this phase, these markers predominantly described the disease state, lacking both specificity and predictive capabilities.

The second stage was the exploration of biomarkers driven by single omics technology. With the development of molecular biology techniques, research entered a single‐omics‐based exploratory phase that focused on molecules related to key pathological pathways. In the inflammatory pathway, hs‐CRP, IL‐6, and TNF‐α were shown to be closely associated with the onset of hypertension [256], and elevated levels indicated activation of vascular endothelial inflammation; vascular cell adhesion molecule‐1 and intercellular adhesion molecule‐1 participated in vascular remodeling by mediating immune cell infiltration [255]. The RAAS‐related molecules became another focus of investigation, and renin, aldosterone, and the aldosterone/renin ratio have become core bases for differentiating primary aldosteronism. Additionally, oxidative stress markers such as superoxide dismutase and malondialdehyde also revealed the role of oxidative damage in the progression of hypertension. This stage provided a molecular basis for mechanistic analysis but did not fully encompass disease complexity [255, 258].

The third stage is the multi‐omics integration and clinical translation phase. Current studies are moving towards a new phase of multi‐omics integration and clinical transformation [259]. By using multidimensional technologies such as genomics, transcriptomics, proteomics, and metabolomics, researchers have identified multiple molecular features associated with hypertension, such as salt sensitivity‐related gene loci and metabolic profiles related to vascular remodeling [255]. High‐throughput transcriptome analysis further revealed novel immune cell subpopulations, providing possibilities for precise intervention in inflammation‐associated hypertension [256]. The application of artificial intelligence and big data analytics has achieved deep integration of biomarkers with clinical and imaging data to construct predictive value‐based risk models for hypertension. The central goal of this phase is to translate scientific discoveries into clinical applications, such as guiding treatment decisions on mineralocorticoid receptor antagonists based on renin levels or predicting cardiovascular event risk by combining multiple biomarkers [258].

#### Main types and typical cases of biomarkers for hypertension

The aldosterone‑to‑renin ratio (ARR) is a key indicator of RAAS activation. A Mendelian randomization study used genetic variations that influence aldosterone secretion as instrumental variables and confirmed that an abnormally elevated ARR has a direct causal relationship with essential hypertension. This finding is consistent with the clinical application of ARR as the preferred screening indicator for primary aldosteronism. By excluding reverse causality, the study demonstrated that autonomous aldosterone hypersecretion is a driver of hypertension rather than a consequence of disease progression, thereby providing solid causal evidence for the precise use of RAAS inhibitors in hypertension treatment.

Biomarkers for diagnosis and screening are primarily used to identify hypertension at an early stage, differentiate its etiology, and classify its subtypes. The aldosterone/renin ratio is the preferred screening indicator for primary aldosteronism [258], as its elevation suggests excessive autonomous secretion of aldosterone, providing vital clues for diagnosing secondary hypertension [257]. This ratio test can diagnose approximately 10−20% of patients with refractory hypertension. hs‐CRP significantly increases in patients with primary hypertension and positively correlates with blood pressure classification [256], making it suitable for initial screening of high‐risk populations. Vascular cell adhesion molecule‐1 and intercellular adhesion molecule‐1 mediate the interaction between white blood cells and endothelial cells, participating in vascular inflammation [258]. An increase in their circulating levels can indicate early damage to endothelial function, providing a reference for the early diagnosis of vascular lesions [259].

Prognostic and predictive biomarkers also exist, which aim to assess the risk of disease progression and clinical outcomes in hypertensive patients. Serum aldosterone levels not only serve as diagnostic indicators but also hold significant prognostic value. An increase in these levels is significantly associated with left ventricular hypertrophy, declining renal function, and an elevated risk of cardiovascular events [258]. sST2, a biomarker for myocardial fibrosis and inflammatory activation, increases in patients with hypertension and heart failure, effectively predicting long‐term mortality. BNP and its N‐terminal precursor, by reflecting ventricular load, hold significant value in the prognostic assessment of hypertension‐induced heart disease, with their continuous increase indicating an elevated risk of heart failure [257]. Additionally, the UMCR serves as an early marker of renal injury, with its increase closely related to the risk of cardiovascular complications in hypertensive patients, making it a routine clinical prognostic assessment indicator [258].

Guidance biomarkers for treatment provide a basis for individualized treatment of hypertension, facilitating the optimization of treatment regimen and monitoring of therapeutic efficacy. Renin, as a key molecule in the RAAS, has guiding significance in drug therapy for primary aldosteronism [252]. After treatment with mineralocorticoid receptor antagonist, the level of renin increases to non‐suppressed state, which can significantly reduce the risk of cardiovascular events. Serum eosinophil count can be used to guide the treatment of patients with hypertension complicated with asthma or allergic diseases. In such patients, its change can reflect the response to glucocorticoids or leukotriene receptor antagonists [252, 257]. In addition, serum potassium level, as a safety monitoring indicator for mineralocorticoid receptor antagonist treatment, maintaining it within the normal range is essential to ensure treatment tolerance, and its dynamic changes can also indirectly reflect the therapeutic effect [258].

Clinical assessment of hypertension heavily relies on imaging and numerical thresholds. Echocardiography is essential for detecting left ventricular hypertrophy and carotid intima‐media thickness (IMT), which are key indicators of early target organ damage. Renal ultrasound aids in evaluating parenchymal injury, while electrocardiography serves as a preliminary screening tool for myocardial ischemia. Fundamental digital diagnostic markers include the thresholds of systolic blood pressure ≥140 mmHg and diastolic blood pressure ≥90 mmHg, with graded values used for disease stratification. The ARR is a core screening parameter for secondary hypertension, the UACR alerts clinicians to early kidney damage, and serum potassium levels serve both as a safety monitoring parameter and an indirect indicator of antihypertensive treatment efficacy.

#### Current challenges and future trends

Research on hypertension biomarkers still faces multiple challenges. First, the disease is highly heterogeneous, encompassing essential and various secondary subtypes with different pathological mechanisms [255, 257]. A single biomarker cannot comprehensively reflect the whole picture of the disease. Second, most existing biomarkers lack high specificity. For example, CRP and BNP can also increase in other diseases, making them unsuitable as specific diagnostic criteria for hypertension [259]. Third, clinical translation is hindered by obstacles. Many potential biomarkers have not been translated into routine practice due to complex detection methods, high costs or a lack of large‐scale clinical validation [39]. Finally, there is a lack of early diagnostic biomarkers. Currently, no sensitive indicators are available to identify high‐risk populations or detect early vascular damage before significant blood pressure elevation [256].

Future research will further develop in the direction of refinement, technology‐driven and integration. In terms of refinement, hypertension classification based on biomarkers will become a trend. For example, aldosterone renin ratio and inflammatory markers level can be used to divide subtypes such as renin angiotensin aldosterone system activation type and inflammation driven type [256]. This will enable individualized treatment. In terms of technology‐driven, multi‐omics technologies combined with artificial intelligence and wearable devices will facilitate new biomarker discovery and dynamic monitoring [259]. For instance, volatile organic compounds can be analyzed through breath metabolomics or blood pressure related physiological signals can be captured in real time by using wearable devices [255]. In terms of integration, constructing a multidimensional model that integrates clinical indicators, imaging data and molecular biomarkers will significantly improve the accuracy of risk prediction and treatment guidance [260]. At the same time, future work will focus on developing early screening biomarkers, predicting the risk of acute complications and monitoring the efficacy of targeted drugs, so as to provide systematic support for the whole process management of hypertension [258, 261].

### Myocarditis

Myocarditis is a disease characterized primarily by inflammation of the heart muscle tissue. Its etiology encompasses various immune‐related factors, including viral infections, autoimmune reactions, and therapies involving ICI [262]. Biomarkers, given their non‐invasive nature, quantifiable attributes, and ease of continuous monitoring, are central to the effective clinical management of myocarditis [263]. In recent times, advancements in molecular biology, multi‐omics technologies, and imaging have propelled the evolution of biomarker research in myocarditis. This progression has broadened the scope from conventional indicators of myocardial damage to sophisticated molecular and multimodal biomarker systems that offer enhanced specificity and functional integration, paving the way for precision diagnostics and therapeutic strategies (Table S15) [262, 263].

#### The development process of myocarditis biomarkers

The first stage was the era of traditional biomarkers, which mainly relied on myocardial injury markers such as troponin (cTnI/cTnT) and creatine kinase isoenzyme (CK‐MB), detecting specific proteins released by necrotic cardiomyocytes to achieve preliminary screening of the disease [264]. High‐sensitivity troponin (hs‐cTnT), due to its extremely high sensitivity, has become an important tool for early identification of myocarditis [262]. In ICI‐related myocarditis, approximately 94% of patients showed elevated high‑sensitivity troponin levels before clinical symptoms appeared [262, 265]. However, these biomarkers have limited specificity and struggle to effectively differentiate between myocarditis and other myocardial injury diseases such as acute coronary syndrome and cardiomyopathy, nor can they reveal the inflammatory nature and immune mechanisms of the disease [266].

The second stage is the multi‐omics technology‐driven exploration period. With the development of technologies such as transcriptome, metabolome and exometabolome, the research has entered a multi‐dimensional mechanism exploration stage [267]. A series of more specific molecular markers have been discovered, including microRNAs, inflammatory factors, inflammatory integrated indicators, exosomal derived RNA and so on. For example, exosomal miR‐34a‐5p can promote myocardial cell senescence by targeting *PNUT* gene and participate in the pathogenesis of ICI‐related myocarditis [264, 268]; while miR‐155 specifically increased in patients' serum and its level was positively correlated with the degree of myocardial inflammation, showing good potential for disease differentiation [265]. This stage realized the transformation from injury markers to mechanism markers, laying a foundation for etiological classification and targeted treatment [269].

The third stage is marked by the integration of omics with clinical translation. Current research has shifted toward synthesizing multi‐dimensional data for practical clinical application [268]. By integrating clinical parameters, imaging features, and molecular biomarkers, and further enhanced by artificial intelligence algorithms, highly accurate diagnostic and prognostic prediction models are being developed [262]. For example, combining troponin, miR‐155, NLR, and CMR imaging parameters can significantly improve early identification rates of ICI‐associated myocarditis [262, 263]. Concurrently, advances in dynamic multi‐marker monitoring panels provide technical support for real‐time assessment of disease activity, treatment response, and prognostic risk, thereby driving the evolution of myocarditis management toward individualized and comprehensive care models [263, 266].

#### Main types and typical cases of myocarditis biomarkers

Diagnostic and screening biomarkers of this type are designed to rapidly identify myocardial inflammation and damage. Troponin (cTnI/cTnT) is a core indicator, and its elevation closely correlates with the severity of myocardial injury [264]. In ICI‐related myocarditis, baseline troponin levels in deceased patients were significantly higher than those in survivors [262]. For example, Muğlu et al. reported that four patients had cTnT levels as high as 500 ng/mL, far beyond the normal range of less than 0.014 ng/mL, providing an important basis for diagnosis [265]. Inflammatory markers such as NLR and CRP, although not organ‐specific, are significantly associated with the risk of major adverse cardiovascular events. Among molecular biomarkers, miR‐155 was highly expressed in both viral and immune myocarditis [268]; exosomal miR‐34a‐5p was upregulated after ICI treatment and expected to be an early screening tool for immune‐related myocarditis.

Prognostic and predictive biomarkers of this type are used to assess disease progression, treatment response, and long‐term outcomes. sST2 has superior diagnostic efficacy to cTnI and NT‐proBNP in fulminant myocarditis and can effectively predict the risk of short‐term mortality [266]. The continuous increase in cytokines such as IFN‐γ and TNF‐α often indicates a severe inflammatory response and is associated with myocardial fibrosis and poor prognosis [262]. Imaging biomarkers such as late gadolinium enhancement (LGE) on CMR can directly show the extent of myocardial fibrosis, and patients with positive LGE have significantly increased long‐term risks of heart failure and malignant arrhythmia. In clinical practice, Sánchez‐Camacho et al. [269] reported a case of MMM overlap syndrome that provided an integrated basis for disease assessment and treatment adjustment by combining cTnI, elevated creatine kinase, and CMR characteristics [262].

Imaging and quantitative indices are indispensable for myocarditis management. CMR is the central imaging modality, with LGE directly visualizing the extent of myocardial fibrosis and serving as a key prognostic indicator for long‐term outcomes. CMR features of myocardial edema and inflammatory infiltration also facilitate early diagnosis and inflammation grading. Clinically, significant elevation of cardiac troponin (cTnI/cTnT) remains the cornerstone for diagnosing myocardial injury, with a normal reference value for cTnT typically defined as <0.014 ng/mL. The NLR provides an auxiliary assessment of inflammatory severity and prognosis. Moreover, investigational digital models incorporating the quantitative expression of serum miR‐155 and exosomal miR‐34a‐5p represent potential early identification tools for ICI‐related myocarditis.

#### Current challenges and future trends

Research on myocarditis biomarkers is still faced with the following main bottlenecks: The disease has a strong heterogeneity, and the expression profiles of biomarkers corresponding to different etiologies are quite different [262]. A single indicator cannot cover all subtypes; traditional biomarkers such as troponin have low specificity, while new molecular biomarkers such as miR‐155 have clear mechanisms but lack large‐scale clinical verification; most new biomarkers remain in basic or small sample research stage, lacking detection standards and consensus guidelines for clinical application; early diagnosis system is not perfect, lacking high sensitivity biomarker combination that can identify high‐risk groups before significant damage of myocardial function.

Future research should focus on several key areas: enhancing precise classification and developing biomarker panels based on etiology and phenotype, such as the “cTnT + miR‐34a‐5p + NLR” panel for ICI‐induced myocarditis [268]; leveraging technology to integrate multi‐omics, artificial intelligence, and single‐cell technologies in the search for highly specific biomarkers like exosomal proteins and immune cell subsets; establishing a comprehensive “molecular‐imaging‐clinical” system for dynamic monitoring throughout the disease course [262, 269]; and broadening targeted applications to guide immunomodulatory therapy and high‐risk population screening, thereby improving diagnostic efficacy and patient prognosis in myocarditis.

### Angiosarcoma

Angiosarcoma tissue morphology frequently overlaps with benign or other malignant vascular lesions such as hemangiomas and epithelioid hemangioendotheliomas, complicating differential diagnosis. Biomarkers, which serve as crucial molecular signals indicative of disease onset and progression, are integral to early detection, classification, prognostic evaluation, and therapeutic guidance [270]. Recent advancements in pathological techniques, multi‐omics analysis, and molecular biology methods have greatly propelled biomarker research for angiosarcoma, offering novel avenues for enhancing clinical diagnosis and treatment approaches (Table S16) [271].

#### The development process of biomarkers

The first stage was the era of traditional pathological diagnosis, in which early diagnosis primarily relied on histological observation and immunohistochemical detection. The focus was on identifying endothelial cell‐specific proteins to confirm the tissue origin of the tumor. Representative markers included *CD31*, *CD34*, and vascular hemophilia factor, among others [270]. Notably, *CD31* exhibited a positive expression rate of 91% in angiosarcoma, establishing it as a crucial basis for differentiating this tumor from other soft tissue tumors. Additionally, FLI‐1, an ETS family transcription factor, demonstrated a nuclear expression sensitivity of 97%, further corroborating the diagnosis of endothelial origin [270, 271]. However, these markers posed challenges in distinguishing between benign and malignant vascular lesions and failed to offer prognostic or treatment guidance information.

The second phase was the multi‐omics‐driven exploration stage. With the development of multi‐omics technologies such as genomics, transcriptome, and metabolomics, the research on biomarkers of angiosarcoma entered the systematic screening stage, a series of gene variations, gene fusion, and abnormal signaling pathways related to the occurrence and development of the disease were revealed [272]. At the genome level, high‐frequency events such as *TP53* mutation, *MYC* amplification, and *KDR* mutation were confirmed [271, 273]. Transcriptome studies identified new gene fusions such as *TEK::GAB2* and *KHDRBS1::NTRK3*, promoting the establishment of molecular classification system; metabolic and signal pathway analysis revealed that vascular endothelial growth factor (VEGF)/VEGFR, PI3K/AKT/mTOR, MAPK/ERK, and other pathways were abnormally activated, and their key proteins became potential therapeutic targets [270, 271, 274].

The third stage is the omics integration and clinical translation era, where current studies have entered a new phase of parallel multi‐omics integration and clinical translation, focusing on integrating multidimensional molecular data and clinical information to construct composite biomarker models with diagnostic, prognostic, and therapeutic guidance value [272]. The technical means include CRISPR‐mediated molecular diagnosis, spatial omics, artificial intelligence, organoid model, etc. For example, the CRISPR‐Dx system can achieve high‐sensitivity detection of specific miRNA and gene mutation; a comprehensive model based on *TP53* mutation, Ki‐67 index, and VEGFR2 expression could effectively predict targeted therapy response. Organoid platform establishment provides key technical support for biomarker function validation and translational research.

#### Main types and typical cases of biomarkers

Diagnostic and screening biomarkers are pivotal for early identification, differential diagnosis, and molecular classification due to their high sensitivity and specificity [273]. Notably, among genetic biomarkers, the *MYC* amplification exhibits a positive rate exceeding 90% in radiation‐related secondary angiosarcomas. Significantly, it does not overlap with benign lesions, establishing it as a crucial criterion for differentiating primary from secondary tumors. Additionally, the *TEK::GAB2* fusion gene has been identified in pediatric pelvic soft tissue sarcomas. This gene activates the PI3K/AKT/mTOR pathway, driving tumor formation and offering molecular diagnostic evidence for cases at rare sites [270].

Among protein biomarkers, ERG nuclear expression exhibits a remarkably high positive rate of 96%. It is seldom found in non‐epithelial tumors, thus demonstrating significant diagnostic specificity [271]; Claudin‐5 displays diffuse membranous expression in angiosarcomas with an impressive sensitivity of 97%. This remains consistent even in poorly differentiated cases, thereby aiding in confirming challenging pathological diagnoses. *CD117* presents a positive rate of approximately 90% in soft tissue angiosarcomas and can serve as a supplementary differentiation indicator [275].

Prognostic and predictive biomarkers are employed to evaluate the risk of disease progression, forecast therapeutic response, and estimate survival outcomes. At the genetic level, amplification of *FLT4* is associated with an unfavorable prognosis, and patients carrying this alteration show improved responses to VEGFR inhibitor therapy [271]. Meanwhile, *PIK3CA* mutations occur more frequently in primary vascular sarcomas of the breast and are correlated with greater tumor invasiveness and an elevated risk of recurrence [271, 275].

At the protein level, the average Ki‐67 proliferation index is 68%, and patients with high expression (>33%) have a significantly increased risk of distant metastasis, with a median disease‐free survival time of only 3.4 months [271]; the PD‐L1 expression rate was approximately 37%, and its positivity was associated with an immunosuppressive microenvironment; high expression of PD‐L1 led to better response to ICI therapy in patients [273]; PAI‐1 inhibitors were involved in disease progression through mechanisms such as promoting angiogenesis and immunosuppression, and showed potential efficacy in paclitaxel resistance models [275].

At the pathway level, abnormal activation of VEGF/VEGFR pathway is a core feature of this type of tumor, and patients with high expression of VEGFR2 respond better to anti‐angiogenic drugs [271]; high expression of p‐*AKT* and p‐S6K in PI3K/AKT/mTOR pathway indicates that the tumor has proliferative and metastatic tendency, and such patients may be sensitive to mTOR inhibitor treatment [273, 276].

Given the histopathological nature of angiosarcoma, clinical assessment relies heavily on quantitative pathological and molecular digital markers. A Ki‐67 proliferation index exceeding 33% is a critical digital threshold indicating a significantly elevated risk of distant metastasis. Positive expression rates of endothelial markers such as *CD31*, FLI‐1, and ERG serve as the quantitative basis for pathological differential diagnosis. The expression rate of PD‐L1 can stratify patients for immunotherapy efficacy. Furthermore, quantitative detection of *MYC* amplification, *TP53* mutation status, and pathway protein expression levels represent potential digital biomarkers for molecular classification, etiological differentiation, and prediction of targeted therapy response.

#### Current challenges and future trends

The study of biomarkers in vascular sarcoma faces several challenges. First, the tumor heterogeneity is pronounced, with molecular characteristics varying significantly based on the disease's location, etiology, and subtype [271]. It is challenging for a single biomarker to represent the essence of the disease. Second, current biomarkers such as *CD31* and *CD34* are also prevalently expressed in benign and other vascular‐derived tumors, leading to inadequate diagnostic specificity. Third, most biomarkers remain in the research phase, lacking extensive clinical validation; only PD‐L1 and VEGFR2 have been successfully implemented [273]. Fourth, there is a dearth of early diagnostic biomarkers, resulting in the majority of patients being diagnosed at an advanced stage [271].

Future research will focus on three major directions: In terms of precision, an integrated “molecular classification‐biomarkers‐treatment regimen” system will be constructed to promote individualized treatment [275]; in terms of technology‐driven, CRISPR‐Dx, spatial transcriptomics and artificial intelligence methods will be used to improve detection sensitivity and model prediction efficiency; in terms of integration and dynamic monitoring, a multidimensional evaluation system will be constructed by integrating clinical, imaging and molecular data, and dynamic monitoring biomarkers such as circulating tumor DNA and circulating miRNA will be developed to achieve real‐time assessment of treatment response and adjustment of treatment plans [272]. Meanwhile, the development of early screening, risk prediction and targeted drug companion diagnostic biomarkers, as well as the establishment of a multicenter collaboration and data sharing mechanism, will jointly promote the clinical transformation and application of vascular sarcoma biomarkers [277].

#### Internal network of cardiovascular biomarkers

Research on cardiovascular biomarkers has evolved from conventional single‐molecule functional descriptions to a new era of integrated analysis within systems biology. Traditional studies have focused primarily on the association between individual biomolecules and diseases, an approach that cannot fully elucidate complex regulatory mechanisms. The concept of systems biology offers a robust analytical framework for this purpose [278]. This section focuses on high‐incidence cardiovascular diseases with intricate mechanisms, such as coronary heart disease, heart failure, hypertension, and atherosclerosis. It systematically reviews the characteristic biomarker spectra at various stages of each disease, encompassing proteins, nucleic acids, and metabolites, among others. It elucidates their expression patterns, interactions, and clinical correlations [279]. Building on this foundation, a multidimensional, multinode, and multilevel integrated network is constructed by integrating molecular regulation, cell responses, and tissue pathological changes. The aim is to provide a more comprehensive and precise theoretical and technical paradigm for an in‐depth analysis of the pathogenesis of cardiovascular diseases, the optimization of early diagnosis, the development of targeted treatment, and the assessment of prognosis (Table S17).

#### Clusters of core injury stress markers

Myocardial injury and stress markers constitute the foundational tier of this integrated network, providing a crucial entry point for examining the pathophysiological processes of cardiovascular diseases [280]. Myocardial troponin (cTn) and its high‐sensitivity detection (hs‐cTn) are central indicators. These markers not only serve as specific diagnostic criteria for acute myocardial infarction but also display distinctive dynamic change patterns across various myocardial injury contexts, including myocarditis, stress‐induced cardiomyopathy, and acute exacerbation of heart failure [281]. The release kinetics can indicate the extent and severity of myocardial necrosis, while sustained low‐level elevations may suggest potential microvascular damage, aiding in the early detection of subclinical myocardial lesions. Concurrently, the natriuretic peptide system (BNP/NT‐proBNP), which acts as a stress‐sensitive marker of the ventricular wall, significantly increases in conditions such as abnormal pressure load from hypertensive heart disease and volume load imbalance due to valvular disease. The magnitude of this increase is positively correlated with the severity of ventricular remodeling. Collectively, these markers form the base tier of the network, establishing a core foundation for subsequent multidimensional integrated analysis [280].

#### Inflammatory fibrosis regulatory network

Markers of inflammation and fibrosis form the second tier of this integrated network, serving as crucial intermediaries in the progression of cardiovascular diseases. Hs‐CRP, a primary marker of low‐grade chronic inflammation, can stimulate the expression of vascular endothelial adhesion molecules, enhance oxidative stress responses, and compromise the integrity of the endothelial barrier, thus promoting and exacerbating atherosclerosis. Gal‐3 and sST2, key mediators of myocardial fibrosis, accurately reflect fibroblast activation and collagen deposition levels. By modulating the transforming growth factor‐β (TGF‐β) signaling pathway, they expedite myocardial interstitial remodeling and intensify ventricular systolic and diastolic dysfunction [282]. Notably, these markers are involved in cross‐disease pathway sharing. For instance, IL‐6 not only contributes to the instability of atherosclerotic plaques via an inflammatory cascade but also plays a pivotal role in the neuroinflammatory damage observed in AD, offering significant insights into the mechanisms underlying cardiocerebral comorbidity.

### The cardiovascular system as a cross‐system network hub

With its comprehensive circulatory network, the cardiovascular system facilitates effective two‐way communication with all bodily organs. This central role in cross‐system biomarker integration is achieved through the interconnection of multiple biological axes, such as the neuro‐humoral–metabolic axis. This enables signal transmission across organs and coordinated regulation of biomarkers [283]. Pathological physiological changes within the cardiovascular system can transport characteristic biomarkers to target organs such as the lungs, kidneys, and brain via circulation, triggering a cascade reaction. Conversely, dysfunction in other organs can release signaling molecules into the bloodstream, which are then recycled to regulate biomarker expression within the cardiovascular system (Table S18) [281].

#### Multidimensional interactive network of the heart, kidney, nerve, and endocrine systems

The heart–kidney axis is closely coupled through hemodynamic and neurohormonal pathways, indicating cross‐system marker integration. In heart failure, reduced cardiac output results in inadequate renal perfusion, activating the RAAS and the sympathetic nervous systems (SNSs). This activation prompts increased sodium and water reabsorption by the kidneys to sustain blood volume but paradoxically intensifies the cardiac load [281]. Conversely, during renal insufficiency, disturbances in water and sodium excretion lead to a significant increase in volume load. The accumulation of uremic toxins harms myocardial cells and the vascular endothelium, establishing a bidirectional pathological feedback loop between the heart and kidneys. In this context, the kidney injury markers neutrophil gelatinase‐associated lipocalin (NGAL) and kidney injury molecule‐1 (KIM‐1) increase synchronously with the cardiac function marker BNP. Their combined detection can overcome the limitations of individual markers, facilitating early warning and assessment of heart‐kidney syndrome [283]. Simultaneously, the neuro‐endocrine axis promotes communication between the cardiovascular system and the autonomic nervous system via pressure reflex sensitivity and heart rate variability. Its malfunction is not only a consequence of cardiovascular events such as myocardial injury but also amplifies ventricular remodeling and arrhythmias because of disrupted neural regulation. This makes it a pivotal factor in disease progression, further elucidating the complexity of the cross‐system integration network [281].

#### Integration of the vascular–metabolic–immune inflammatory axis system

The vascular system serves as a central interface for metabolic and immune interactions, with the vascular endothelium playing a pivotal role in establishing communication pathways for multisystem regulation. The integrity of endothelial function is intrinsically linked to metabolic homeostasis and immune equilibrium. Dysfunctional markers, such as asymmetric dimethylarginine, are associated with insulin resistance and increased arterial stiffness because of inhibited nitric oxide synthesis and disrupted vascular dilation. These markers serve as early indicators of metabolic cardiovascular diseases [283]. In patients with type 2 diabetes (T2D), erratic blood glucose levels lead to an abnormal accumulation of advanced glycation end products (AGEs), which specifically activate vascular inflammatory pathways. This results in elevated expression of inflammatory factors such as IL‐6 and TNF‐α. Such factors not only intensify endothelial damage but also hasten lipid deposition and plaque formation, collectively advancing atherosclerosis [284]. Notably, this axis shares foundational inflammatory underpinnings with the nervous system. For example, the CRP level is predictive not only of coronary heart disease risk and prognosis but also of the neuroinflammatory processes of vascular dementia, offering fresh insights into the molecular associations of metabolic‐cardiovascular‐neural comorbidities [285].

#### Cross‐system identification of common biomarker networks

On the basis of the aforementioned cross‐system interaction mechanism, systematic integration analysis can pinpoint core biomarker clusters shared by the cardiovascular system and other systems. Notably, inflammatory hub biomarkers such as IL‐6, TNF‐α, and CRP are central to cross‐system inflammatory regulation. These biomarkers not only indicate the instability of plaques in cardiovascular diseases but also play pivotal roles in neuroinflammation associated with Alzheimer's disease, metabolic inflammation in diabetes, and immune disorders in autoimmune conditions [286]. The metabolic integration biomarker HbA1c, recognized as the gold standard for long‐term blood glucose control, serves as a significant risk indicator for cardiovascular complications in diabetes patients. Its abnormal elevation is closely linked to vascular cognitive impairment because of its detrimental effects on the cerebral vascular endothelium and its ability to induce neuroinflammation [283]. Stress response biomarkers, including catecholamines and cortisol, exhibit characteristic increases during both psychological stress and cardiovascular events. They show a pathological link between mental stress and cardiac damage via the sympathetic–adrenal–medullary axis. Oxidative stress biomarkers, reactive oxygen species (ROS) and oxidized low‐density lipoprotein collectively contribute to the pathogenesis of atherosclerosis, neurodegenerative diseases, and insulin resistance by causing cell damage and intensifying inflammatory responses, thus driving multisystem pathological processes in a coordinated manner (Figure S2) [287].

## DIGESTIVE SYSTEM

Comprehensive literature searches were executed in the PubMed and Web of Science databases using Boolean logic with field‐restricted search strings. For PubMed, searches combined MeSH terms and title/abstract fields, whereas for Web of Science, topic field searches were applied. The detailed search strategies for each disease were as follows: for metabolic dysfunction‐associated steatotic liver disease, (“MASLD” OR “metabolic associated steatotic liver disease” OR “MAFLD” OR “metabolic associated fatty liver disease” OR “NAFLD” OR “nonalcoholic fatty liver disease”) AND (“biomarker” OR “biomarkers” OR “biological marker” OR “biological markers”); for viral hepatitis, (“viral hepatitis”) AND (“biomarker” OR “biomarkers” OR “biological marker” OR “biological markers”); for liver cirrhosis, (“liver cirrhosis”) AND (“biomarker” OR “biomarkers” OR “biological marker” OR “biological markers”); for inflammatory bowel disease, (“inflammatory bowel disease” OR “IBD”) AND (“biomarker” OR “biomarkers” OR “biological marker” OR “biological markers”); for pancreatitis, (“pancreatitis”) AND (“biomarker” OR “biomarkers” OR “biological marker” OR “biological markers”); for esophagus cancer, (“esophagus cancer” OR “esophageal cancer”) AND (“biomarker” OR “biomarkers” OR “biological marker” OR “biological markers”); for gastric cancer, (“gastric cancer” OR “stomach cancer”) AND (“biomarker” OR “biomarkers” OR “biological marker” OR “biological markers”); for colorectal cancer, (“colorectal cancer” OR “colon cancer” OR “rectal cancer”) AND (“biomarker” OR “biomarkers” OR “biological marker” OR “biological markers”); for hepatocellular cancer, (“hepatocellular cancer” OR “hepatocellular carcinoma” OR “HCC” OR “hepatoma”) AND (“biomarker” OR “biomarkers” OR “biological marker” OR “biological markers”); for pancreatic cancer, (“pancreatic cancer” OR “pancreatic carcinoma”) AND (“biomarker” OR “biomarkers” OR “biological marker” OR “biological markers”); and for cholangiocarcinoma, (“cholangiocarcinoma” OR “CCA” OR “bile duct cancer”) AND (“biomarker” OR “biomarkers” OR “biological marker” OR “biological markers”). The retrieval period was defined as January 1, 2020 through December 31, 2025. Only articles published in English were considered. Both original research articles and systematic or narrative reviews were included. Retrieved records were screened by titles and abstracts; articles deemed irrelevant to digestive disease biomarkers, lacking full‐text availability, or containing incomplete data were excluded, and the remaining full texts were subjected to thorough evaluation. The selected disease spectrum encompasses metabolic dysfunction‐associated steatotic liver disease, viral hepatitis, liver cirrhosis, inflammatory bowel disease (IBD), pancreatitis, esophageal cancer, gastric cancer, colorectal cancer, hepatocellular carcinoma, pancreatic cancer, and cholangiocarcinoma, all representing conditions with high incidence, substantial disease burden, well‐established biomarker frameworks, and strong clinical translational relevance.

### Metabolic dysfunction‐associated steatotic liver disease

MASLD, a metabolic liver disease linked to insulin resistance and genetics, ranges from steatosis to MASH, cirrhosis, and hepatocellular carcinoma. The pathogenesis of MASLD is multifaceted, influenced by factors such as genetics, environment, and lifestyle [288]. Its heterogeneity complicates early diagnosis. Despite liver biopsy being the gold standard, limitations have driven interest in non‐invasive biomarkers [289]. recent years have seen significant advancements in biomarker research on MASLD, thanks to the integrated application of lipidomics, metabolomics, and molecular biology technologies. These developments provide novel methods and tools for clinical management (Table S19) [290].

#### The development process of biomarkers

Research on MASLD biomarkers has evolved in parallel with advancing disease understanding and technological progress, broadly categorized into three stages. In the initial phase, reliance was primarily placed on traditional clinical indicators such as liver function parameters and metabolic markers. However, their lack of specificity made it challenging to effectively distinguish MASLD from other liver or metabolic diseases [288]. As comprehension of the pathological mechanisms underlying MASLD deepened, a second stage witnessed the emergence of more markers rooted in pathophysiological processes, including molecules associated with inflammation and lipid metabolism. The application of lipidomics and mass spectrometry analysis techniques facilitated the discovery of lipid molecules like phosphatidylcholine and polyunsaturated fatty acids, whose level alterations are closely linked to the onset and progression of MASLD [289]. The third stage marks an era of multi‐dimensional integration. The amalgamation of multi‐omics technologies with innovative detection methods, including machine learning and extracellular vesicle analysis, has given rise to diagnostically valuable composite markers and new markers. This phase emphasizes the differentiation of disease subtypes, prognostic assessment, and individualized treatment [290]. Furthermore, as the nomenclature of the disease has evolved from MASLD to MAFLD and back to MASLD, biomarker research has increasingly concentrated on specific indicators related to metabolic disorders [288].

#### The main types of biomarkers and typical cases

Lipid metabolic disorders are the core pathological features of MASLD. Lipidomics studies have shown that the levels of polyunsaturated fatty acid phospholipids and non‐esterified fatty acids in the plasma and liver tissues of patients with MASLD generally decrease (1−9%), among which the decrease of phosphatidylcholine level in the liver is a typical manifestation. Taking *TM6SF2* gene mutation carriers as an example, the levels of triglycerides and phosphatidylcholine polyunsaturated fatty acids in their liver decreased, while the levels of saturated and monounsaturated fatty acids increased [289]. Such changes in lipid profile are closely related to fat accumulation in the liver. In addition, the ratio of very low density lipoprotein ceramide [Cer (d18:1/16:0)/Cer (d18:1/24:0)] was positively correlated with triglyceride accumulation in the liver, and could be used as a potential marker for disease progression in MASLD. This type of marker can not only reflect the state of the disease, but also directly participate in key pathological processes such as hepatic lipid deposition and inflammatory response.

Chronic inflammation is a key driver in the progression of MASLD [291]. Novel inflammatory markers, namely the neutrophil percentage to albumin ratio (NPAR) and the neutrophil to albumin ratio (NAR), are significantly elevated in patients with MASLD. The standardized mean difference (SMD) for NPAR is 0.28, while that for NAR is 0.69. These markers collectively reflect systemic inflammation and liver nutritional status. The combined sensitivity of NPAR for diagnosing MASLD is 69.5%, with a specificity of 63.1% and an area under the curve (AUC) of 76.05%, indicating potential value for early screening [291]. Furthermore, traditional inflammatory factors such as C‐reactive protein, interleukin‐6, and TNF‐α also correlate with the severity of MASLD [288].

Hepatic fibrosis is a critical stage in the progression of MASLD to liver cirrhosis and hepatocellular carcinoma [288]. Commonly used fibrosis scoring systems include FIB‐4, MASLD fibrosis score, and BARD score. Notably, FIB‐4 has an AUC of 0.80 and a specificity of 98% when assessing advanced fibrosis. The MASLD fibrosis score also demonstrates a high predictive value for advanced fibrosis (AUC = 0.82). Additionally, extracellular matrix‐related molecules such as type III procollagen amino‐terminal peptide, hyaluronic acid, and tissue inhibitor of metalloproteinase‐1 are frequently utilized in fibrosis evaluation. Specifically, hyaluronic acid has an AUC of 0.89, with a sensitivity of 85% and a specificity of 80%, in diagnosing advanced liver fibrosis [288].

With the advancement of technology, novel markers such as extracellular vesicle‐related markers, hepatocyte factors and microRNA are constantly emerging [290]. The proteins and microRNA carried by extracellular vesicles are tissue specific. Their quantity and content in serum significantly change in patients with MASLD. For example, the AUC of miR‐128‐3p in serum extracellular vesicles for differentiating MASH from healthy individuals can reach 0.989. The AUC of GLUT1 on the surface of hepatocyte‐derived extracellular vesicles was 0.85 for differentiating MASLD from MASH. Fibroblast growth factor‐21 (FGF‐21), a hepatocyte factor regulating lipid metabolism, has significantly increased circulating levels in patients with MASLD, which further increases during MASH and liver cirrhosis stages [292]. In addition, changes in lipid profiles related to ANGPTL3 inhibitors and metabolites derived from gut microbiota also show potential biomarker value.

When exploring the causal relationship between non‐alcoholic fatty liver disease (NAFLD) and lung function, traditional observational studies are vulnerable to interference from confounding factors and reverse causal relationships. A mendelian randomization study utilized genetic variation as an instrumental variable to evaluate the causal effect of lung function on NAFLD [293]. The study found that for every one standard deviation increase in the forced expiratory volume in the first second and forced vital capacity predicted by genes, the risk of NAFLD was significantly reduced. After adjusting for height, the independent protective effect of FEV1 on NAFLD still exists. Reverse analysis did not find a causal effect of NAFLD on lung function. This study provides genetic evidence for the causal relationship between lung function impairment and the risk of NAFLD, suggesting that FEV1 can serve as a potential reference indicator for NAFLD risk assessment.

#### Current challenges and future development trends

Research on MASLD biomarkers still faces multiple challenges. First, the specificity and sensitivity of existing biomarkers in differentiating subtypes of MASLD and MASLD caused by different etiologies are not satisfactory [288, 289]. Second, disease heterogeneity leads to limited applicability of markers among people with different genetic backgrounds, metabolic states, and ethnic groups [289, 291]. Third, detection methods lack standardization, thresholds are not uniform, and results are difficult to compare and promote across studies. Fourth, most markers remain at the research stage, lacking large‐scale multicenter clinical validation and having low efficiency in clinical translation. Fifth, the value of markers in prognostic evaluation and treatment response monitoring has not been fully verified, making it difficult to meet the needs of individualized medicine [292].

Future research should prioritize several key directions. First, multi‐dimensional data from lipidomics, metabolomics, and genomics should be integrated with machine learning algorithms to construct composite marker panels that enhance diagnostic and staging accuracy. Second, specific markers for different causes, stages, and subtypes of MASLD should be developed to facilitate precision medicine. Third, non‐invasive detection technologies utilizing blood, fecal, and urine samples should be advanced by integrating microfluidics and biosensors to improve the convenience and accessibility of these methods. Fourth, the application of these markers should be expanded to include prognostic assessment and therapeutic monitoring to better guide clinical decision‐making [292]. Fifth, international multi‐center collaboration is essential for establishing standardized testing procedures and reference thresholds, thereby promoting the clinical translation and widespread adoption of these markers [288, 291]. Furthermore, as the understanding of disease mechanisms deepens, mechanism‐oriented markers targeting key pathological processes such as lipotoxicity, inflammation, and fibrosis are expected to become a major focus of research.

### Viral hepatitis

Viral hepatitis comprises types A, B, C, D, and E. Among these, chronic hepatitis B (CHB) and chronic hepatitis C (CHC) are the primary causes of liver cirrhosis and hepatocellular carcinoma. Globally, more than 250 million individuals are infected with HBV and 70 million with HCV. The early and accurate identification of liver injury and fibrosis progression is crucial for improving patient prognoses. As an invasive procedure, traditional liver biopsy is challenging to implement on a large scale. Biomarkers, detected through non‐invasive sampling, can objectively reflect the risks of liver damage, viral replication, fibrosis, and carcinogenesis, and have thus become core tools for clinical management (Table S20).

#### The development process of biomarkers

The development of biomarkers for viral hepatitis can be categorized into three stages. Initially, the focus was on traditional liver function enzymatic indicators such as ALT, AST, ALP, and GGT. Among these, ALT serves as a sensitive marker for hepatocyte injury and is extensively used in screening and monitoring. The AST/ALT ratio aids in assessing the risk of severe liver injury and cirrhosis. However, at this stage, while these markers are easy to operate and cost‐effective, they lack etiological specificity and cannot differentiate between viral hepatitis and other liver diseases [294]. Subsequently, the discovery of serological markers like hepatitis B surface antigen (HBsAg) and hepatitis B core antibody (anti‐HBC) facilitated the etiological diagnosis of viral hepatitis. The detection of HBV‐DNA and HCV‐RNA has become the primary indicators for evaluating viral replication activity and have been incorporated into global guidelines for the diagnosis and treatment of viral hepatitis [294, 295]. In the mid‐term phase, non‐invasive markers of liver fibrosis, such as FIB‐4, ELF, and PLT, have become the preferred tools for primary screening [296]; virological markers like serum HBV RNA and HBcrAg, which reflect the transcriptional activity of cccDNA, are utilized for treatment evaluation and guiding drug withdrawal; AFP combined with ultrasound improves the efficiency of early HCC screening; and factors such as FGL1, FGL2, and SCFA offer new perspectives on gut–liver axis research [296]. In the recent period, multi‐omics, machine learning, and single‐cell sequencing have driven the development of composite markers. Quantitative HBsAg subtypes, HBV‐DNA integration, and ctDNA have shown potential in staging and individualized treatment [296, 297]. The 2025 EASL guidelines will incorporate quantitative HBsAg, HBcrAg, and HBV RNA into disease stratification and treatment decision‐making. Markers will shift from single detection to a combination of multiple indicators, and their applications will expand to include prognosis prediction, determination of treatment endpoints, and new drug development, gradually achieving precise management [294].

#### The main types of biomarkers and typical cases

Virological markers are the gold standard for etiological diagnosis, assessment of replication activity and monitoring of therapeutic effects in viral hepatitis. They can be divided into three categories: viral nucleic acids, viral antigens and subtypes of antigens. HBV‐DNA and HCV‐RNA as markers of viral nucleic acids are direct evidence of the presence and replication activity of viruses. Their load reflects the degree of infection and is the core basis for guiding antiviral drugs and judging treatment response. The sensitivity and specificity of detection are extremely high, and they have been included in all guidelines for the diagnosis and treatment of viral hepatitis [294, 295]. HBsAg is the gold standard for the diagnosis of HBV infection. Persistent positive suggests chronic infection. Its quantitative detection (qHBsAg) can predict the response to interferon therapy and guide the withdrawal of nucleoside (acid) analogs. HBsAg subtypes (LHB, MHB, SHB) are involved in the processes of viral adsorption and immune escape. The change in ratio can indicate the infection stage and treatment response, which is a core evaluation index for functional cure. HBcrAg contains proteins such as HBcAg and HBeAg, which have high correlation with cccDNA levels. It can stably reflect the status of viral replication, predict the risk of liver fibrosis, liver cirrhosis and HCC, and is suitable for rapid detection in resource‐limited areas [294, 295]. Serum HBV RNA, as a noninvasive alternative marker of cccDNA transcriptional activity, can predict serum conversion of HBeAg/HBsAg and recurrence after treatment, making up for the shortcomings of traditional virological markers.

Hepatic fibrosis is a critical stage in the progression of chronic viral hepatitis to liver cirrhosis. Non‐invasive fibrosis markers offer an alternative to liver biopsy for extensive screening and ongoing follow‐up. FIB‐4, a composite index derived from AST, ALT, platelet count, and age, is both straightforward and cost‐effective. A FIB‐4 score below 1.45 suggests an absence of significant fibrosis, while a score above 3.25 indicates a heightened risk of fibrosis, making it the favored non‐invasive assessment tool in clinical settings. ELF, on the other hand, is determined by levels of hyaluronic acid, type III procollagen amino‐terminal peptide, and tissue inhibitor of metalloproteinase 1. This marker provides a direct indication of liver fibrosis activity, predicts potential complications of liver cirrhosis, assesses HCC risk, and offers greater accuracy than FIB‐4. Additionally, as a cellular marker, PLT decreases in instances of liver cirrhosis due to hypersplenism. A PLT value under 100 × 10^9^/L signals a potential risk of portal hypertension. When paired with the AST/ALT ratio, its precision in fibrosis evaluation increases, establishing it as a vital screening instrument in primary healthcare contexts [294].

With the development of multi‐omics technology, novel proteins and metabolic markers have become research hotspots, expanding the assessment dimensions of viral hepatitis. FGL1 is involved in hepatocyte proliferation and liver regeneration while also promoting liver cancer, thus exhibiting dual functionality. FGL2 exists in both membrane‐bound and soluble forms, which are respectively implicated in thrombosis, immunosuppression, and regulation of disease progression. SCFAs are produced through dietary fiber metabolism by the intestinal flora. They inhibit hepatic inflammation and maintain the intestinal barrier via GPR41/GPR43 receptors. A reduction in their levels indicates gut–liver axis dysfunction and negatively correlates with the severity of NAFLD and liver cirrhosis. Consequently, they serve as an indicator for evaluating the efficacy of microbiota modulation therapies [296].

A cross‐sectional serosurvey of 300 blood donors in South Africa (100 each black, mixed race and white) showed that anti‐HAV positivity was 61% overall, highest among blacks (86%) and increasing with age, consistent with fecal–oral spread in areas of poor hygiene. Anti‐HEV IgG was 26% overall, but the distribution differed: mixed race 33%, whites 23%, blacks 20%, also increasing with age (5% under 21 to 46% over 46). No HEV IgM was detected, indicating past infection only. This contrasts with HAV and suggests a different mode of transmission for HEV. The authors suggest that higher pork consumption by the mixed race and white populations may increase the risk of zoonotic infection with HEV genotype 3, supported by previous detection in HIV patients and transplant recipients. Thus, HEV in South Africa is likely to be mainly zoonotic rather than waterborne. The study highlights the need to include racial, socioeconomic and behavioral factors in the epidemiology of hepatitis in order to optimize prevention [298].

#### Current challenges and future development trends

Although advancements have been made in the study of biomarkers for viral hepatitis, their clinical application remains fraught with challenges. The majority of these biomarkers lack adequate specificity and sensitivity, and standardized detection methods for emerging biomarkers are yet to be established [294, 295]. Disease heterogeneity, influenced by factors such as genotype and infection stage, impacts the universal applicability of these markers [296, 297]. Furthermore, there is a dearth of standardized detection protocols and quality control measures, leading to difficulties in result replication. The efficiency of translating these findings into clinical practice remains suboptimal, compounded by an absence of extensive long‐term validation using large sample sizes. Additionally, multi‐index joint evaluation models have not gained widespread acceptance. The process of detecting HBV–DNA integration is both intricate and expensive, and its underlying mechanisms remain partially understood [297]. These limitations hinder precise disease staging and personalized treatment approaches.

In the future, biomarkers for viral hepatitis are expected to evolve towards multi‐omics integration, precision, and non‐invasiveness. Key development directions include: constructing a multi‐index joint detection panel to improve diagnostic accuracy; utilizing high‐sensitivity technologies such as microfluidics and digital PCR; establishing an internationally standardized testing system; extending applications to prognosis assessment and drug withdrawal guidance; developing gut–liver axis targeted markers; promoting the clinical application of HBV‐DNA integration markers for early liver cancer screening; and formulating specific marker combinations based on individual patient characteristics to achieve precise management [294, 296].

### Liver cirrhosis

Liver cirrhosis, the terminal stage of chronic liver disease, is characterized by extensive hepatocellular damage, tissue remodeling, and excessive deposition of the extracellular matrix [299]. It frequently progresses to liver failure or hepatocellular carcinoma, posing a major public health burden. With the rising prevalence of chronic liver diseases, early identification, dynamic monitoring, and precise prognostic assessment of cirrhosis are essential to improve outcomes [300]. Liver biopsy is limited by invasiveness, sampling error, and low accessibility, making non‐invasive, repeatable biomarkers essential. Integrating lipidomics, metabolomics, and molecular biology has advanced biomarker research into a multi‐dimensional, mechanism‐driven phase covering lipid metabolism, immune inflammation, fibrosis, and novel molecular targets (Table S21) [301].

#### The development process of biomarkers

The research process of liver cirrhosis biomarkers has evolved in tandem with pathological understanding and technological innovation, which can be divided into three typical stages [301]. In the early stage, studies focused on traditional liver function indicators, metabolic parameters and systemic inflammatory markers. Although these indicators are easy to operate, they lack specificity for liver fibrosis response and have difficulty achieving early diagnosis and etiological differentiation of liver cirrhosis. In the second stage, with the deepening of pathogenesis research, technology platforms based on lipidomics and mass spectrometry analysis promoted the emergence of a batch of pathologically specific markers. Lipid metabolites such as phosphatidylcholine and polyunsaturated fatty acids were revealed to be closely related to the progression of liver cirrhosis. Composite inflammatory indexes such as neutrophil‐to‐albumin ratio (NAR) and NPAR were gradually applied in clinical evaluation; Fibrosis‐related molecules such as hyaluronic acid (HA) and amino‐terminal peptide of type III procollagen (PIIINP) were also systematically verified. In addition, Golgi protein 73 (GP73), as a new serum marker, showed excellent diagnostic potential [300]. In the third stage, the research entered a period that equally emphasized multi‐omics integration and clinical translation. The integration of lipidomics, transcriptomics, proteomics and machine learning algorithms has led to a variety of composite marker panels. Micrornas and hepatokines carried by extracellular vesicles are prominent in staging and prognostication [301]. As the concept of disease progresses towards metabolic dysfunction‐associated fatty liver disease (MAFLD/MASLD), biomarker development is increasingly focused on core mechanisms of metabolic dysregulation. In addition, exploration of markers associated with immune typing of “hot” versus “cold” fibrosis lays the foundation for targeted immunomodulatory therapy [302].

#### The main types of biomarkers and typical cases

Lipid metabolism disorders pervade the entire course of liver cirrhosis [301]. Typically, there is a decrease in polyunsaturated fatty acid levels by 1−9% in both plasma and liver tissues of patients. In individuals with *TM6SF2* gene mutations, there is a significant reduction in triglycerides and phosphatidylcholine rich in polyunsaturated fatty acids, while an increase in the proportion of saturated and monounsaturated fatty acids promotes fat accumulation and fibrosis progression. Phosphatidylcholine plays a crucial role in the assembly of liver lipoproteins. Disruption in its synthesis results in an increase in specific plasma subtypes, indicating impaired lipid transport function. The ratio of ceramides in very low‐density lipoprotein (Cer(d18:1/16:0)/Cer(d18:1/24:0)) positively correlates with triglyceride deposition in the liver, suggesting worsening fibrosis [303]. Triglycerides, a routine clinical indicator, abnormally increase in liver cirrhosis due to enhanced intrahepatic synthesis and peripheral mobilization. Angiopoietin‐like protein 3 contributes to the process of liver cirrhosis through regulation of lipoprotein metabolism, and inhibitors of this protein have entered the stage of clinical exploration [303].

Chronic inflammation is closely associated with the activation and fibrosis process of hepatic stellate cells [300]. The NAR and NPAR integrate inflammation and nutritional status [301]. Meta‐analysis showed that they were significantly elevated in patients with liver cirrhosis (NPAR‐SMD = 0.28; NAR SMD = 0.69), and the area under the curve of NPAR for diagnosing liver cirrhosis reached 76.05%. Classical inflammatory factors such as CRP, IL‐6, and TNF‐α are closely related to the severity of liver cirrhosis. IL‐6 and TNF‐α promote collagen deposition by activating HSCs, and their levels gradually increase with disease progression [299]. Soluble macrophage markers such as *CD16*, *CD14*, and *CD163* can reflect Kupffer cell and infiltrating macrophages status and have important prognostic value in alcoholic and MASLD‐related liver cirrhosis. GP73 is a cross‐biomarker of inflammation‐fibrosis, its serum level is consistent with the severity of the disease, and it has been used as a candidate indicator for non‐invasive diagnosis [300].

Hepatic fibrosis is the core pathological process of liver cirrhosis, and its markers focus on extracellular matrix metabolism and stellate cell activation. Common integrated systems such as FIB‐4 (AUC = 0.80) and MASLD fibrosis score (AUC = 0.82) are widely used for advanced fibrosis screening and risk stratification due to their simplicity and efficiency [304]. ELF panel has an excellent diagnostic efficacy in advanced fibrosis (AUC 0.80−0.90). At a molecular level, procollagen III amino‐terminal peptide directly reflects collagen synthesis rate; Hyaluronic acid was used to evaluate hepatic sinusoidal endothelial cell injury and progression of fibrosis, with AUC of 0.89 for diagnosis of advanced liver cirrhosis. Tissue inhibitor of metalloproteinase 1 promotes fibrosis by inhibiting collagen degradation [303]. Cytokeratin‐18 M30 fragment specifically marks hepatocyte apoptosis and positively correlates with steatosis, inflammation, and fibrosis severity. Mac‐2 binding protein glycosylation isomer, as a novel glycoprotein marker, showed good diagnostic performance in liver cirrhosis with various etiologies (AUC = 0.81).

The AUC of exosomal miR‐128‐3p was as high as 0.989 in differentiating metabolism‐related steatohepatitis from healthy controls. The AUC of GLUT1 on the surface of hepatocyte vesicles for differentiating fatty liver disease from MASH was 0.85, which showed great potential for early diagnosis [301]. FGF‐21, a hepatokine regulating lipid metabolism, had significantly increased circulating levels (SMD = 0.61) and positively correlated with the severity of disease in patients with liver cirrhosis13. In addition, noninvasive markers such as gut microbiota metabolites and microRNA‐34a also demonstrated good diagnostic and prognostic values [305]. YKL‐40, a dual‐function marker of inflammation and fibrosis, was significantly overexpressed in virus‐related liver cirrhosis and associated with hepatocellular carcinoma risk [305]. Abnormal adiponectin level reflected liver metabolic disorders and fibrotic process [304].

At the metabolomics level, the development of spatial metabolomics technology provides a brand‐new perspective for analyzing the metabolic reprogramming during the progression of liver cirrhosis to liver cancer. A study employed air flow‐assisted desorption electrospray ionization mass spectrometry imaging (AFADESI‐MSI) to conduct spatial metabolomics analysis on healthy controls, hepatitis B‐related liver cirrhosis, and liver cancer tissues, achieving in situ and high‐throughput detection of hundreds of metabolites in the tissues [306]. The results showed that both liver cirrhosis and liver cancer tissues had significant metabolic heterogeneity, and different pathological regions presented differentiated metabolic characteristics.

In terms of amino acid metabolism, significant changes occur in pathways such as β‐alanine metabolism, arginine and proline metabolism, and alanine/aspartic acid/glutamic acid metabolism in liver cirrhosis and liver cancer tissues. Among them, spermidine shows a continuous downward trend during the progression from liver cirrhosis to liver cancer, especially with the most significant decline from the pseudolobular area to the cancer lesion area of liver cancer. Carbamoyl phosphoric acid, on the other hand, shows a continuous upward trend. Carbamoyl phosphate is a metabolic product of the first step of the urea cycle. The overexpression of its synthase, carbamoyl phosphate synthase 1 (CPS1), can promote pyrimidine synthesis, which is closely related to tumor proliferation. In addition, taurine shows a continuous increase in the central venous area and the adjacent area of liver cirrhosis and liver cancer tissues. Considering that taurine has the effect of inducing tumor cell apoptosis, its increase may reflect the compensatory response of the body during the process of liver injury. In terms of lipid metabolism, the glycerophospholipid metabolic pathway undergoes significant alterations in the regions related to liver cancer. Compared with non‐cancerous areas, the levels of phosphatidylcholine (PC), lysophosphatidylcholine (LPC), and glycerophosphatidylcholine in cancerous foci and precancerous lesion areas were significantly increased. Glycerolipin is an important component of cell membranes, and its upregulation in synthesis is closely related to the proliferation requirements of tumor cells. In addition, the key enzyme for the conversion of LPC to PC‐lysophosphatidylcholine acyltransferase 1 (LPCAT1)—is overexpressed in various tumors, further supporting the significant role of glycerol phospholipid metabolic remodeling in the occurrence of liver cancer. It is worth noting that through the analysis of the receiver operating characteristic curve, the above‐mentioned differential metabolites have a relatively high predictive efficacy (AUC approximately 0.9) in differentiating healthy tissues from diseased tissues, but their efficacy decreases (AUC approximately 0.7) when differentiating liver cirrhosis from liver cancer, suggesting the continuity of metabolic changes between liver cirrhosis and liver cancer. These findings not only deepen the understanding of metabolic reprogramming during the progression of liver cirrhosis to liver cancer, but also provide new candidate targets for the development of early diagnostic markers based on metabolites [306].

#### Current challenges and future development trends

Research on biomarkers for liver cirrhosis still faces many challenges. First, the specificity and sensitivity of markers are insufficient to distinguish different causes and clinical stages [307]. Second, the disease is highly heterogeneous. Genetic background, comorbidities, and racial differences significantly affect marker expression [308]. Third, testing lacks standardization, leading to variations in methods, thresholds, and sample processing among centers, which hinders result comparison and promotion. Fourth, clinical translation lags behind. Most markers remain at the validation stage and lack support from multicenter and prospective studies [301]. Fifth, there is insufficient evidence linking markers to hard endpoints, making it difficult to guide individualized treatment.

Future research should focus on several key areas: the construction of multi‐omics fusion marker panels that integrate lipid spectroscopy, inflammatory indicators, and fibrotic molecules, leveraging artificial intelligence to enhance diagnostic and staging accuracy [300]; the development of etiology‐ and stage‐specific markers for the precise classification of metabolic, viral, and alcoholic liver cirrhosis; the creation of non‐invasive, real‐time detection technologies based on microfluidics and biosensors to improve accessibility in primary medical care settings [309]; the expansion of marker applications for early warning of complications and monitoring treatment response, such as using FGF‐21 to evaluate the efficacy of targeted drugs; the strengthening of international multi‐center collaborations to establish a standardized testing system and a shared database [308]; and the deepening of mechanism‐oriented marker research, with a focus on core processes like hepatic stellate cell activation and macrophage polarization, thereby supporting coordinated innovation in diagnosis and therapy.

### Inflammatory bowel disease

IBD is a complex group of diseases characterized by chronic and recurrent inflammation of the gastrointestinal tract, primarily comprising two subtypes: Crohn's disease and ulcerative colitis [310]. Its pathogenesis involves multiple factors, including genetic susceptibility, intestinal flora imbalance, abnormal immune regulation, and environmental factors. IBD diagnosis and management face challenges. Endoscopy, the gold standard, is invasive, costly, and has low compliance. Clinical heterogeneity complicates the assessment of disease activity. Biomarkers, which are objective and measurable indicators, demonstrate indispensable value in early screening, disease classification, treatment response, and prognosis [310]. With rapid advancements in molecular biology, omics technologies, and biosensing techniques, research on IBD biomarkers has expanded from conventional serological indicators to multi‐dimensional fields such as fecal markers, intestinal microbiota markers, and genetic and epigenetic markers, providing novel technical support for achieving precision diagnosis and treatment (Table S22).

#### The development process of biomarkers

In the early period, studies in this phase primarily focused on conventional clinical indicators. Serological markers such as CRP and erythrocyte sedimentation rate (ESR) were extensively employed, but they lacked disease specificity and struggled to differentiate IBD from other IBDs. Although fecal calprotectin and lactoferrin were initially investigated, their diagnostic efficacy remained limited. During this time, research into the intestinal microbiota was still nascent. Only a reduction in microbiota diversity was observed in IBD patients, with no specific markers identified [311]. The fecal calprotectin detection system was approved by the US FDA for marketing in 2006. It is used for the in vitro quantitative detection of calprotectin concentration in human feces as an in vitro diagnostic reagent to assist in the diagnosis of IBD, and to distinguish IBD from irritable bowel syndrome. In the second phase, as understanding of the pathological mechanisms of IBD deepened, research on markers broadened towards disease specificity [312]. Inflammatory indicators such as the neutrophil‐lymphocyte ratio and S100A12 were confirmed to correlate with disease activity. Fecal calprotectin has been widely validated as a core noninvasive inflammatory marker. Breakthroughs occurred in microbiome research. The reduced abundance of beneficial bacteria such as *Faecalibacterium prausnitzii* and *Akkermansia muciniphila*, along with increased abundance of pathogenic bacteria like *Escherichia coli*, were established as key microbial markers. GWAS identified multiple susceptibility genes such as *IL23R* and *NOD2*, while epigenetic regulation, including DNA methylation and microRNA, also began to garner attention. Technological advancements, including 16S rRNA sequencing and ELISA, have underpinned marker screening methodologies. Since 2021, research has transitioned into a phase characterized by multi‐dimensional integration and precision. The synergistic use of multi‐omics offers a comprehensive understanding of disease mechanisms. Emerging biomarkers like HMGB1 and Gal‐3 demonstrate notable potential in both diagnosis and prognostic evaluations. Innovations in biosensing technology enhance detection convenience, while wearable devices and microfluidic chips facilitate real‐time monitoring of indicators such as calprotectin [313]. Consequently, the application spectrum of these markers is continually broadening, commencing with diagnostic utilities.

#### The main types of biomarkers and typical cases

CRP elevates markedly under inflammatory conditions and correlates positively with IBD disease activity. However, its clinical utility is limited, as approximately 50% of patients with ulcerative colitis can exhibit normal CRP levels during active disease. ESR, while simple to measure, is susceptible to various confounding factors and is often used only as an auxiliary indicator. Cytokines such as IL‐6 and TNF‐α are key mediators of inflammatory responses, and their levels closely reflect disease severity. Composite indices derived from routine blood tests, including NLR and PLR, hold unique value in disease assessment. Among newer markers, LRG shows a stronger correlation with endoscopic scores than CRP and is not influenced by CRP levels. HMGB1 mediates inflammation via the TLR4/RAGE pathway, serving as both a potential diagnostic marker and a therapeutic target [314].

Calprotectin, a neutrophil‐derived protein, exhibits a diagnostic sensitivity of 88% and specificity of 80% [315]. It effectively differentiates IBD from irritable bowel syndrome and predicts disease recurrence and mucosal healing. Its critical value is influenced by various factors and should be interpreted in conjunction with clinical contexts. Fecal lactoferrin offers a diagnostic specificity of 95% and demonstrates commendable stability. S100A12 presents a higher disease specificity (96%), but the supporting research remains limited. Regarding gut microbiota markers, individuals with IBD show diminished microbiota diversity and an imbalanced structure. The presence of beneficial bacteria like *Faecalibacterium prausnitzii* negatively correlates with disease activity. Conversely, pathogenic bacteria such as *Escherichia coli* increase, and their ratio to *F. prausnitzii* can differentiate disease subtypes. Additionally, decreased levels of microbiota metabolites, including SCFAs, hold potential as biomarkers [310].

Over 200 IBD susceptibility genes have been identified through Genome‐Wide Association Studies (GWAS). Notably, the *IL23R* gene is strongly associated with Crohn's disease, while the *HLA* gene is closely linked to ulcerative colitis. These genes predominantly function in pathways related to the epithelial barrier and immune activation. Their polymorphisms influence both disease susceptibility and treatment outcomes. In terms of epigenetic regulation, there are aberrant methylation patterns observed in specific genes within the intestinal mucosa of IBD patients. Furthermore, alterations in the expression of microRNAs such as miR‐155 and miR‐223 have been implicated in the pathogenesis by modulating T cell differentiation and the secretion of inflammatory factors [315].

Composite indicators such as the neutrophil‐albumin ratio, which integrate inflammation and nutritional status, yield an area under the curve of 76.05% for the early diagnosis of IBD. As a core factor of Th17 cells, elevated IL‐17 levels are closely correlated with disease activity. A decreased ratio of regulatory T cells to Th17 is a hallmark of immune imbalance [311]. Gal‐3 influences disease progression by regulating intestinal barrier function and immune cell activity; Its expression patterns in serum and tissues are subtype‐specific, making it a potential diagnostic and prognostic marker [314].

Systems such as FIB‐4 and MASLD fibrosis score can assess the risk of liver fibrosis. The area under the curve for extracellular matrix molecules, such as hyaluronic acid, in diagnosing advanced liver fibrosis is 0.89 [312]. VCAM‐1 levels are positively correlated with disease activity and the degree of fibrosis, and it is also a potential marker of colorectal cancer. Osteopontin is involved in disease progression by mediating inflammatory and fibrotic processes [314].

miR‐128‐3p carried by extracellular vesicles showed high diagnostic efficacy in disease differentiation [310]. The level of FGF‐21 was associated with the severity of the disease. Circular RNAs regulate gene expression through miRNA sponge action and their stability and specificity endow them as important potential markers. Histone modifications and epigenetic markers, such as long noncoding RNAs, also provide new targets for diagnosis.

#### Current challenges and future development trends

The lack of specificity in markers limits their clinical application. Most existing markers struggle to accurately differentiate IBD from other inflammatory diseases. Disease heterogeneity results in varying expressions of biomarkers across different populations and subtypes. The standardization of detection methods is lacking, leading to poor comparability of results across different platforms [310]. Furthermore, the efficiency of translating these findings into clinical practice is low, with most new biomarkers not undergoing large‐scale validation. Their potential value in predicting treatment outcomes and assessing prognosis remains underutilized. There is also a deficiency in integrating multi‐dimensional data, resulting in a scarcity of comprehensive marker panels.

Multi‐omics integration and the development of composite markers will become a focal point, with high‐precision diagnostic models being constructed through machine learning algorithms [310]. Investigations into individualized markers will advance the practice of precision medicine, enabling predictions of treatment responses based on genetic and microbiota characteristics. Detection technology is evolving towards convenience and real‐time capabilities, with wearable devices and instant detection technologies enhancing clinical accessibility. The range of application scenarios is continually expanding to encompass full‐cycle management, including treatment monitoring and early warning of complications. International cooperation and standardization efforts are accelerating the clinical translation of these markers. Mechanism‐oriented research is deepening our understanding of the biological functions of these markers and fostering their development towards integrated diagnosis and treatment [312].

### Pancreatitis

Pancreatitis, classified as acute or chronic, involves pathogenic mechanisms including enzyme activation, inflammation, oxidative stress, and fibrosis, posing a health threat [316]. Significant progress has recently been made in the research of biomarkers for pancreatitis, facilitated by rapid developments in lipidomics, transcriptomics, and molecular biology techniques. This research encompasses a multi‐type biomarker system that includes inflammatory factors, enzyme indicators, nucleic acid molecules, and complex models (Table S23) [317].

#### The development process of biomarkers

The evolution of research on biomarkers for pancreatitis is closely related to the understanding of disease mechanisms and technological innovation, which can be divided into three stages. In the early stage, studies focused on the clinical application of traditional enzyme markers. Amylase and lipase, as key enzymes released after pancreatic acinar cell injury, have become core indicators in the diagnosis of AP. The level of amylase rises rapidly within 2−12 h after the onset of the disease. Although it is easy to detect, its specificity is limited and easily interfered with by diseases of the salivary gland and gastrointestinal tract. Lipase has gradually become the preferred indicator for the diagnosis of AP due to its high pancreatic specificity and long window period (lasting 7−14 days) and detection rate of 90%−95% in severe AP. Additionally, inflammatory factors such as TNF‐α and IL‐6 began to be used to assess the degree of inflammatory activity, but their association with disease severity remains unclear [318]. In the second stage, with an in‐depth study of pathological mechanisms, marker research expanded to multiple dimensions [317]. Inflammatory and immune‐related indicators, such as the NAR and red blood cell distribution width to albumin ratio (RAR), have been confirmed to be significantly associated with mortality and severity of AP. Fibrosis‐related markers, such as TGF‐β1 and TIMP‐1, provide new tools for assessing fibrosis in CP [319]. Meanwhile, nucleic acid markers, such as miRNAs, are emerging, and their expression changes are closely related to chronic inflammation and fibrosis processes. The application of mass spectrometry and high‐throughput sequencing technologies has facilitated systematic screening and validation of markers [317]. In the third stage, research entered a phase of multidimensional integration and development of novel biomarkers. tRNA‐derived small RNAs (tsRNAs), such as tRF3‐Thr‐AGT, were involved in the pathogenesis of AP by regulating pyroptosis and ferroptosis, demonstrating diagnostic potential [320].

#### The main types of biomarkers and typical cases

Amylase and lipase are the most widely used traditional markers in clinical practice. Serum pancreatic amylase peaks at 2−12 h after onset of AP, with high sensitivity but limited specificity. Lipase is secreted almost exclusively by the pancreas and has significantly better specificity than amylase, with a longer window period up to 7−14 days and a positive diagnostic rate for severe AP exceeding 90% [318]. In CP, serum lipase levels gradually decrease as acinar cells atrophy and exocrine function declines, which can be used to assess functional impairment.

TNF‐α and IL‐6 are the core inflammatory mediators. TNF‐α aggravates tissue damage by activating the NF‐κB pathway [319]. The level of IL‐6 is positively correlated with the severity of AP, which can be used to dynamically monitor the treatment effect. New composite indicators such as NAR and RAR integrate inflammation and nutritional status; meta‐analysis shows that elevated RAR is significantly associated with mortality risk in AP patients. In CP, IL‐6 and sCD16*3* respectively reflect chronic inflammation and macrophage activation, and participate in the fibrosis process [319].

FIB‐4 and other scores. such as NFS, effectively evaluate the degree of CP fibrosis by integrating age, platelets, and liver function parameters. Among them, FIB‐4 has a specificity of 98% for advanced fibrosis. The AUC of extracellular matrix‐related molecules, such as HA, was 0.89 in diagnosis of advanced fibrosis. TGF‐β1 and TIMP‐1 promote fibrosis by promoting activation of stellate cells and inhibiting degradation of matrix, and their serum levels are positively correlated with lesion severity [319]. An elevated MMP‐9/TIMP‐1 ratio indicates an increased risk of fibrosis progression.

miRNAs are involved in the disease process by regulating the expression of genes related to inflammation and fibrosis. miR‐19a, miR‐143, and miR‐374‐5p were abnormally expressed in CP earlier than imaging changes. tsRNAs affect the cell death process by regulating the NLRP3 inflammasome and p53 pathway, exhibiting high stability and tissue specificity [317]. In addition, gene mutations such as *PRSS1* and *SPINK1* are associated with hereditary CP risk and have been used for familial screening.

#### Current challenges and future development trends

Although the research on pancreatitis biomarkers has made significant progress, it still faces many challenges. The main problem is that the diagnostic performance of existing markers remains limited. Most markers are difficult to accurately distinguish different clinical subtypes and etiological heterogeneity of pancreatitis. For example, amylase and lipase can also be elevated in salivary gland and biliary diseases, which seriously affects the specificity of diagnosis. At the same time, the high heterogeneity of the disease leads to significant individual differences in marker expression. The expression level and clinical significance of markers vary among different genetic backgrounds, metabolic states, and ethnic groups [319]. On a technical level, detection methods and judgment thresholds have not yet been standardized. Differences in antibodies, reagents, and operational procedures across platforms make horizontal data comparison challenging and hinder their promotion in clinical practice. More critically, most novel markers such as tsRNAs and extracellular vesicle‐related indicators remain at the basic research stage, lacking large‐scale multicenter clinical validation, with transformation processes significantly lagging behind [317, 320]. Additionally, the prognostic value and treatment response monitoring capability of these markers have not been fully established, making it difficult to support actual needs for personalized diagnosis and treatment.

Research on pancreatitis biomarkers is trending toward systematization, precision, and practicality. Multi‐omics integration and the development of composite markers have emerged as key areas of focus. The fusion of multi‐dimensional data from lipidomics, metabolomics, and genomics, combined with machine learning algorithms to construct comprehensive diagnostic models, can significantly enhance the ability to distinguish disease stages and subtypes. Researchers are increasingly focusing on the exploration of disease subtype‐specific markers and developing differentiated marker combinations for various etiologies and disease stages, such as alcoholic and hereditary forms, to advance the practice of precision medicine [317]. Concurrently, technological innovation is also advancing. Non‐invasive detection technologies utilizing platforms such as liquid biopsy, microfluidics, and biosensors enable rapid and convenient analysis of readily accessible samples like blood and urine. Regarding their scope of application, the utility of these markers is expanding from diagnosis to other clinical decision‐making processes, including prognostic assessment, therapeutic monitoring, and severe illness risk prediction, thereby facilitating comprehensive disease management. Furthermore, strengthened international multi‐center collaboration and the establishment of unified technical standards and validation systems will substantially accelerate the clinical translation of these markers [320]. As the understanding of the pathological mechanisms of pancreatitis deepens, mechanism‐oriented markers derived from key pathological events, such as inflammatory activation, fibrosis progression, and novel cell death patterns like ferroptosis and pyroptosis, are anticipated to become a significant research direction in the future. These markers might offer valuable new targets for targeted therapy.

### Esophagus cancer

Esophageal cancer, a prevalent digestive tract malignancy, includes squamous cell carcinoma and adenocarcinoma. The concept of precision medicine has driven the gradual emergence of biomarkers as key tools for enhancing early screening, pathological classification, treatment decision‐making, and prognosis assessment of esophageal cancer. This provides a new direction for overcoming the current diagnostic and therapeutic bottlenecks (Table S24) [321].

#### The development process of biomarkers

Research on biomarkers for esophageal cancer has evolved from morphological assessment to multi‐omics integration, encompassing three key stages. The initial stage primarily relied on pathological morphological markers, including tumor differentiation grade, depth of invasion, and lymph node metastasis status. While these indicators provide a fundamental basis for clinical staging and prognostic evaluation, their limited sensitivity and specificity are insufficient for early diagnosis and precision treatment [321]. For example, although HE staining can assess tumor malignancy, it is challenging to predict patient responses to chemotherapy or immunotherapy using this method [322]. During the intermediate development stage, advances in molecular biology techniques shifted research focus toward single‐gene or single‐protein markers. Landmark discoveries included key molecules such as HER2 and PD‐L1. HER2 emerged as an early biomarker for targeted therapy in esophageal cancer, while PD‐L1 facilitated the clinical implementation of immunotherapy [323, 324]. Additionally, novel markers like FGFR2b and KLK13 were validated through clinical trials, further expanding targeted therapy options [323, 325]. The current developmental stage is characterized by multi‐omics integration and cross‐modal fusion. Researchers systematically investigate potential markers with causal relationships, such as HPSE and ST3GAL1, through integrated analyses combining genomics, transcriptomics, proteomics, and microbiomics [325]. Multimodal deep learning models now enable comprehensive analysis of pathological images, CT scans, and clinical data, significantly improving prediction accuracy for PD‐L1 expression and immunotherapeutic efficacy [322]. Concurrently, esophageal and intestinal microbiota have emerged as promising biomarker sources, demonstrating increasing clinical applicability.

#### The main types of biomarkers and typical cases

Molecular markers are the most mature category in esophageal cancer research, mainly including two types of markers: protein and gene. As a key marker for immunotherapy, PD‐L1 inhibits T cell activity by binding to PD‐1, thereby facilitating immune evasion by tumors [324]. Its expression level can be detected using immunohistochemistry and is commonly quantified by the tumor area positive score (TAP) and combined positive score (CPS). Studies have shown that both scoring methods exhibit concordance at multiple critical values (Cohen's *κ* = 0.64–0.85) and effectively identify populations benefiting from immunotherapy in tislelizumab treatment. The multimodal deep learning model integrates pathological, imaging, and clinical information, achieving non‐invasive prediction of PD‐L1 with an AUC of 0.836 [322]. FGFR2b is overexpressed in approximately 38% of advanced esophageal cancers and promotes tumor progression by activating the PI3K/AKT/mTOR pathway. It can be detected using the VENTANA FGFR2b kit, which demonstrates good sensitivity and specificity [323]. The phase II FIGHT clinical trial demonstrated that bemarizumab combined with chemotherapy significantly prolonged progression‐free survival and overall survival in patients with high FGFR2b expression (≥10%), with a median overall survival of 24.7 months. *SECTM1*, as an immune‐related genetic marker, is highly expressed in esophageal cancer tissues and enhances malignant tumor behavior by promoting M2 macrophage polarization and *CCL5* expression. The prognostic model constructed based on *SECTM1*, *MAP3K8*, and *IGLV7‐43* demonstrated stable efficacy in both training and validation cohorts, with predicted AUCs for 1–3 year survival rates ranging from 0.669 to 0.736. Additionally, genetic markers such as *HPSE*, *ST3GAL1*, *CEL*, *KLK13*, and *GNRH2* have been identified through Mendelian randomization studies as being causally associated with esophageal cancer [325].

Microbial markers, as an emerging research direction, have shown great potential in the diagnosis and treatment of esophageal cancer. *Fusobacterium nucleatum* was significantly enriched in ESCC tissues and positively correlated with tumor stage progression and poor prognosis. It promotes tumor progression by activating NOD1/RIPK2/NF‐κB pathway and upregulating IL‐32/PRTN3 expression, and can induce myeloid‐derived suppressor cell recruitment, leading to chemotherapy resistance [321]. This bacterium also regulates PD‐L1 expression and inhibits antitumor immunity. Its tissue abundance can be used to predict immunotherapy response. *Porphyromonas gingivalis* is also abundant in ESCC. It inhibits apoptosis of epithelial cells through PI3K/AKT pathway, promotes epithelial‐mesenchymal transition, and immune cell recruitment. The activation of TGF‐β/Smad pathway further enhances its carcinogenic ability. Clinical studies have confirmed that positive expression of this bacterium is closely related to advanced tumor staging and poor prognosis. In intestinal microbiota, *Streptococcus* and *Bacteroides* are increased while *Helicobacter* and *Faecobacter* are decreased in feces from ESCC patients. These microorganisms affect the function of intestinal barrier and immune microenvironment by regulating metabolites such as short chain fatty acids, thus participating in tumorigenesis. Besides, patients with high *Pyramidobacter* and *Butyricimonas* were more likely to achieve pathological complete response after neoadjuvant immunochemotherapy.

Imaging and multimodal markers offer novel technical approaches for the precise diagnosis and treatment of esophageal cancer. Pathological image markers, leveraging deep‐learning algorithms, extract morphological features from HE sections. These markers can predict tumor grade and PD‐L1 expression with an AUC value exceeding 0.75. They can also assess the immune cell infiltration status within the tumor microenvironment. CT image markers, through radiomics analysis, extract features such as tumor shape, density, and texture, which are associated with PD‐L1 expression and therapeutic response. The rates of change in tumor diameter and density before and after treatment exhibit a significant correlation with progression‐free survival and overall survival in patients. Multimodal markers integrate pathology, imaging, and clinical information to build high‐precision prediction models. The multimodal deep‐learning model developed by Liu et al. demonstrated outstanding performance in predicting immunotherapy response and survival outcomes, with an AUC value of 0.809 [322]. Specifically, the model utilized a ResNet50 encoder pre‐trained via adversarial contrastive learning (AdCo) to extract patch‐level features from H&E‐stained whole‐slide images. Meanwhile, radiomics features were extracted from CT‐defined regions of interest using PyRadiomics. A LASSO algorithm was employed to select the most informative radiomic and clinical features, followed by a self‐attention module to adaptively weight the fused multimodal representations. This framework enables non‐invasive prediction of PD‐L1 expression (AUC = 0.836) and immunotherapy response, offering a comprehensive foundation for formulating individualized treatment plans.

Beyond traditional molecular markers, Mendelian randomization studies have provided causal evidence linking gut microbiota to esophageal cancer risk. When exploring the causal relationship between gut microbiota and esophageal cancer, traditional observational studies are vulnerable to interference from confounding factors and reverse causality. A Mendelian randomization study utilized large‐scale genome‐wide association research data and genetic variations as instrumental variables to evaluate the causal association between 150 types of gut microbiota and esophageal cancer [326]. The results show that there is a causal relationship between the increased abundance of seven genera, such as *Gordonibacter*, *Oxalobacter*, *Coprobacter*, *Veillonella*, *Ruminiclostridium* 5, *Ruminococcus*, and *Senegalimassilia*, and the increased risk of esophageal cancer. The increased abundance of four bacterial genera, namely *Turicibacter*, *Eubacterium oxidoreducens* group, *Romboutsia* and *Prevotella* 9, was associated with reduced risk. This study provides genetic evidence for the regulation of esophageal cancer occurrence by the gut microbiota and reveals potential preventive intervention targets.

#### Current challenges and future development trends

Despite significant advancements in the research of biomarkers for esophageal cancer, their clinical application continues to face numerous challenges. The current standardization system for detection remains imperfect, with discrepancies in results across various platforms and reagent kits compromising the consistency of marker interpretation. A majority of existing markers lack disease specificity, as they are widely cross‐expressed in digestive tract tumors. The high heterogeneity of tumors further complicates matters, making it difficult for local biopsies to fully encapsulate molecular characteristics. Moreover, most novel biomarkers are still in the research phase, lacking multi‐center clinical validation and standardized testing procedures. This deficiency restricts their clinical promotion and application.

In light of these challenges, future research will prioritize the integration of multi‐omics strategies and the exploration of highly specific novel markers using systems biology approaches. This will foster innovation in liquid biopsy and artificial intelligence‐assisted diagnostic technologies, enabling minimally invasive and dynamic disease monitoring. Furthermore, it aims to establish a precise treatment system based on molecular typing to facilitate marker‐guided individualized treatment. Through interdisciplinary collaboration, this research will expedite the transition of biomarkers from discovery to clinical application, ultimately constructing a comprehensive biomarker system that encompasses screening, diagnosis, treatment, and prognosis assessment.

### Gastric cancer

Gastric cancer remains a major global health threat with persistently high incidence and mortality. Currently, gastroscopy combined with biopsy remains the carcinoembryonic antigengold standard for diagnosing gastric cancer. However, this method is invasive, subject to sampling bias, and unsuitable for large‐scale screening, leading to low patient acceptance [327]. In serological testing, traditional tumor markers such as CEA and carbohydrate antigen 19‐9 (CA19‐9) exhibit limited sensitivity and specificity, rendering their diagnostic value insufficient when used individually [328]. Consequently, the development of non‐invasive, highly specific, and highly sensitive biomarkers holds significant importance for the early screening, diagnosis, prognostic assessment, and individualized treatment of gastric cancer (Table S25).

#### The development process of biomarkers

Research on biomarkers for gastric cancer has undergone continuous evolution in tandem with a deepening understanding of the disease and technological innovation. This research can be broadly categorized into three stages. In the initial stage, spanning from the early 21st century to 2010, the focus was primarily on the discovery and validation of traditional protein‐based tumor markers. Classic markers such as CEA, CA19‐9, and CA72‐4 were progressively incorporated into clinical auxiliary diagnosis and therapeutic effect monitoring. However, these markers lack disease specificity and can also elevate in many benign gastrointestinal diseases. Furthermore, they exhibit low sensitivity for early gastric cancer, failing to meet the criteria for independent diagnosis. During this period, *Helicobacter pylori* (*H. pylori*) was identified as a significant pathogenic factor for gastric cancer, and its detection became a crucial component of early screening. The second stage witnessed an expansion of biomarker research to the molecular level, driven by a deeper understanding of the pathological mechanisms of gastric cancer. The rise of targeted and immunotherapy has driven the clinical application of markers such as HER2, PD‐L1, microsatellite instability/mismatch repair deficiency (MSI‐H/dMMR), and Epstein‐Barr virus (EBV). HER2 has become a central biomarker for targeted therapy, while PD‐L1, MSI‐H/dMMR, and EBV provide a key basis for immunotherapy screening [327]. Novel markers, including non‐coding RNAs (microRNAs, lncRNAs), circulating tumor DNA (ctDNA), and circulating tumor cells (CTCs), have begun to emerge in the research field. Techniques such as lipidomics and mass spectrometry analysis have further facilitated the screening and validation of these markers. In the third stage, biomarker research has entered a phase of multi‐dimensional integration and clinical translation. The combination of multi‐omics technologies with new detection techniques, such as machine learning algorithms, liquid biopsy, and extracellular vesicle analysis, has driven the development of composite marker panels and novel, specific markers [329].

#### The main types of biomarkers and typical cases

Protein markers are the most mature and widely used type in clinical practice. CEA is frequently overexpressed in gastric cancer, and its levels are associated with tumor adhesion, invasion, and angiogenesis. Its level is positively correlated with tumor burden and metastasis risk, and can be used for therapeutic effect monitoring and early warning of recurrence. However, the sensitivity of early diagnosis is only 4.3−30%, and combined detection is often required. The positive rate of CA19‐9 in advanced gastric cancer (18−44%) is higher than that in early stage, and it has a relatively high sensitivity in monitoring liver metastases. Its specificity (85−95%) is better than that of CEA, but about 5−10% of Lewis antigen negative patients have false negatives. The diagnostic specificity of CA72‐4 exceeds 90%, and it is closely related to tumor staging and lymph node metastasis. Its sensitivity alone is low (18−30%), but when combined with CEA and CA19‐9, it can be increased to 60−70%. α‐Fetoprotein is re‐expressed in hepatoid adenocarcinoma subtype of gastric cancer due to cellular dedifferentiation, which can predict the risk of liver metastases [328]. Patients with high expression have poor response to chemotherapy and short survival time. About 10−20% of gastric cancers present with gene amplification or protein overexpression of HER2. It promotes tumor proliferation by activating MAPK and PI3K/AKT pathways, and is the core target of anti‐HER2 targeted therapy. The VEGF/VEGFR2 pathway promotes tumor angiogenesis, and patients with high VEGFR2 expression respond better to anti‐angiogenic drugs such as ramucirumab. c‐MET is amplified or overexpressed in approximately 5–10% of gastric cancers. It promotes tumor proliferation, invasion, and drug resistance through the HGF/c‐MET pathway and represents an important target for overcoming resistance to HER2‐targeted drugs. Claudin18.2 is overexpressed in approximately 30−40% of gastric cancers and is a promising target for CAR‐T and antibody‐drug conjugate (ADC) therapies, demonstrating significant efficacy in patients with diffuse‐type gastric cancer and peritoneal metastasis. The expression level of PD‐L1 is positively correlated with the efficacy of ICIs.

Genomic characteristic markers, MSI‐H/dMMR, are caused by mismatch repair gene mutations or methylation. They have a high tumor mutation burden and an ICI ORR of 30−50%, making them a population with immunotherapy advantages. TMB‐H patients have abundant neoantigens, good immunotherapy responses, and long survival times, partially overlapping with MSI‐H/dMMR. *TP53* is the most common mutated gene in gastric cancer, with approximately 50% of patients having mutations. This leads to loss of DNA damage repair and cell cycle regulation functions, promotes proliferation, invasion, and chemotherapy resistance, and is positively correlated with recurrence risk [328]. The *APC* gene is commonly methylated or mutated in intestinal‐type gastric cancer, promoting abnormal cell proliferation through Wnt pathway activation.

miR‐21 is highly expressed in gastric cancer tissues and serum. It inhibits proteins such as *PTEN* and *PDCD4*, thereby activating the PI3K/AKT pathway and promoting tumor proliferation, invasion, and chemotherapy resistance. The diagnostic sensitivity is approximately 75%. miR‐375 is a tumor suppressor microRNA that exhibits low expression in gastric cancer and is associated with gastric mucosal atrophy and intestinal metaplasia [327]. It demonstrates significant diagnostic value for early gastric cancer, boasting a sensitivity of approximately 70% and a specificity of around 85%. The *hsa_circ_0001789* is also observed to be underexpressed in gastric cancer. This molecule modulates the FOXO3a pathway by sequestering miR‐182‐5p, thereby inhibiting tumor proliferation and invasion. Its diminished expression correlates positively with advanced disease stages and lymph node metastasis. Additionally, lncRNA *HOTAIR* has been found to impede the expression of tumor suppressor genes by enlisting the PRC2 complex, subsequently fostering tumor growth and chemotherapy resistance. There exists a positive correlation between its presence and the likelihood of recurrence. PiR‐823, identified as a tumor suppressor piRNA, shows reduced expression levels that are directly linked to increased tumor staging and distant metastases. Regarding circulating nucleic acid markers, ctDNA harbors tumor‐specific mutations, such as those in the *TP53* and *KRAS* genes, which makes it highly valuable for early diagnosis and MRD monitoring. In terms of timeliness, ctDNA outperforms imaging techniques [329].

As a Group 1 carcinogen, *H. pylori* infection is associated with approximately 70−90% of gastric cancers. Its virulence factors, CagA and VacA, activate the NF‐κB and Wnt pathways, leading to chronic inflammation, gastric mucosal atrophy, intestinal metaplasia, and ultimately progression to gastric cancer. Infection with CAGA‐positive strains significantly elevates the risk of gastric cancer. Eradication treatment can reduce this risk by 30−40%. The combined detection of serum pepsinogen (PG) and gastrin‐17 (G‐17) enhances the accuracy of screening for precancerous lesions. Approximately 10% of gastric cancers are related to EBV [327]. The virus activates the NF‐κB pathway through the expression of proteins such as EBER and LMP1, promoting inflammation and immunosuppression, and exhibits a relatively high response rate to ICIs. EBER in situ hybridization serves as the gold standard for diagnosis. Patients with this subtype demonstrate high expression rates of PD‐L1 and TMB, making them an advantageous population for immunotherapy. *Fusobacterium nucleatum*, an oral‐derived bacterium, is enriched in gastric cancer tissues. It promotes inflammation and inhibits T cell function by activating the TLR4/NF‐κB pathway, showing a positive correlation with the progression of gastric cancer and chemotherapy resistance. Patients with high abundance of this bacterium have a poor prognosis [329]. Its detection in saliva is convenient and minimally invasive, serving as an auxiliary indicator for prognosis and response to immunotherapy. The abundance of beneficial bacteria (such as *Lachnospira* and *Faecalibacterium*) in the feces of patients with gastric cancer decreased, while that of pathogenic bacteria (such as *Enterobacteriaceae*) increased. The accuracy of *Desulfovibrio* and *Escherichia* as predictive markers was above 0.9043. The levels of *Neisseria* and *Prevotella* in salivary microbiota decreased, while *Streptococcus* increased. These changes were related to the occurrence of gastric cancer and could be used for non‐invasive screening [329].

Beyond classical molecular and microbial markers, emerging evidence highlights the role of ferroptosis‑related molecules in gastric cancer. For instance, GPX4 is highly expressed in gastric cancer and resists ferroptosis by inhibiting lipid peroxidation [330]. Its expression is negatively correlated with lymph node metastasis, invasion, and prognosis. As a product of lipid peroxidation, 4‐hydroxynonenal was associated with the TNM stage of gastric cancer. Its level increased in patients with postoperative recurrence and could be used as a dynamic monitoring indicator for disease progression. USP7 inhibited ferroptosis by stabilizing SCD protein and promoted the growth and metastasis of gastric cancer, making it a potential prognostic marker and therapeutic target.

#### Current challenges and future development trends

Research on biomarkers for gastric cancer still faces many bottlenecks. First, the sensitivity and specificity of markers are insufficient. Most existing markers have poor performance in differentiating subtypes of gastric cancer, early diagnosis, and differential diagnosis from other digestive diseases. For example, CEA and CA19‐9 have low sensitivity for early‐stage gastric cancer. Second, tumor heterogeneity limits the applicability of markers [328]. The expression of markers varies among genetic backgrounds, metabolic states, and ethnicities. HER2 detection requires multi‐site biopsy to overcome the influence of heterogeneity. Third, there is a lack of standardization of detection methods. Different techniques (such as IHC, NGS, ELISA) and judgment thresholds make it difficult to compare and promote results. For example, the antibodies and cutoff values used for PD‐L1 detection are not uniform. Fourth, the efficiency of clinical transformation is low. Most new markers (such as non‐coding RNA and ferroptosis‐related markers) are still in basic or early clinical research stages and lack large‐scale multicenter validation [329]. Fifth, the value of markers in prognostic evaluation and treatment response monitoring has not been fully confirmed. Although dynamic monitoring of CTC and ctDNA has potential, unified clinical application guidelines have yet to be established. In addition, some detection technologies are costly and complex to perform, limiting their popularization in primary medical institutions [328].

Looking forward, research on gastric cancer biomarkers is set to usher in a new paradigm characterized by coordinated evolution, multi‐dimensional integration, and precise application. The profound integration of multi‐omics technology with artificial intelligence algorithms facilitates the construction of composite marker models boasting high predictive performance. This approach allows for precise stratification based on etiology and molecular typing, fostering the development of individualized marker panels. Furthermore, advancements in non‐invasive detection methods, such as liquid biopsy and microfluidics, are poised to significantly improve both the accessibility and dynamic monitoring capabilities of these markers. Consequently, the range of application scenarios has broadened, encompassing everything from diagnosis to prognosis assessment, treatment response monitoring, and early warnings of drug resistance. The establishment of standardized testing systems, coupled with enhanced international multi‐center collaboration, promises to expedite the transition of biomarkers from foundational discovery to clinical implementation. This will ultimately culminate in the creation of a comprehensive biomarker system that spans the entirety of gastric cancer management.

### Colorectal cancer

Colorectal cancer (CRC) ranks third in incidence and second in cancer‐related deaths globally. The prognosis of colorectal cancer is closely correlated with the stage at diagnosis. The 5‐year survival rate for patients diagnosed at early stages can reach approximately 80%, whereas for those with advanced‐stage disease, it is less than 15%. However, due to the insidious nature of early symptoms, most patients are diagnosed at intermediate or advanced stages [331]. Although colonoscopy is the gold standard for diagnosis, its invasiveness results in low patient compliance. Serum markers such as CEA and CA19‐9 exhibit limited sensitivity and are therefore inadequate for early screening. Consequently, the development of highly specific and sensitive biomarkers is of critical importance for the early detection, precise staging, prognostic evaluation, and individualized treatment of colorectal cancer (Table S26) [332].

#### The development process of biomarkers

Research on biomarkers for colorectal cancer has evolved in parallel with advances in disease understanding and technological innovation, and can be broadly categorized into three stages. In the initial stage, research primarily focused on traditional serum protein markers (such as CEA, CA19‐9, CA50, CA72.4) and conventional clinical parameters (such as blood glucose, blood lipids, and liver function indicators). During this period, the detection of gene mutations such as *KRAS* and *BRAF* was initially applied to guide targeted therapy. However, these markers generally lacked specificity, making it difficult to effectively distinguish colorectal cancer from other digestive tract diseases or metabolic abnormalities. Furthermore, their low detection rate for early lesions limited their widespread application in screening [331]. In the second stage, a deepening understanding of the pathological mechanisms of colorectal cancer and the emergence of omics technologies shifted the research focus toward specific molecules associated with pathophysiological processes. Inflammatory indicators, lipid metabolism‐related molecules, and microbial markers garnered significant attention [332, 333, 334]. Liquid biopsy technology was also preliminarily applied to the investigation of CTCs and ctDNA, the latter of which demonstrated potential for detecting minimal residual disease. Additionally, microsatellite instability and mismatch repair defects were confirmed to be associated with immunotherapy efficacy, establishing them as important prognostic and predictive markers [333, 334]. In the third stage, biomarker research has entered a multi‐dimensional integration and precision phase [335]. The integration of multi‐omics technologies with machine learning algorithms has facilitated the development of composite marker panels. Liquid biopsy technology is maturing, with ctDNA, CTCs, exosomes, and their cargo miRNAs emerging as focal points for dynamic disease monitoring and treatment response evaluation. There is an increased emphasis on subtype differentiation and personalized applications, including the development of specific markers for different molecular subtypes and metastatic sites.

#### The main types of biomarkers and typical cases

An imbalance in the gut microbiota is pivotal in the onset and progression of colorectal cancer. Among the bacteria, *Fusobacterium nucleatum* exhibits significant enrichment in tumor tissues. It adheres to the colonic epithelium via proteins like FadA and Fap2, subsequently activating oncogenic pathways such as β‐catenin, thereby fostering tumor proliferation, invasion, and immune evasion [332, 333]. Its presence in the feces of colorectal cancer patients markedly increases. When detected alongside other pathogenic bacteria, notably *Colibactin*‐producing *Escherichia coli*, it yields a combined sensitivity of 0.72 and specificity of 0.76, suggesting its potential as a non‐invasive screening marker. Furthermore, this bacterium has been linked with resistance to both chemotherapy and immunotherapy. Notably, its metabolite, succinic acid, can suppress the CGAS‐STing‐IFN‐β pathway, leading to reduced CD8^+^ T cell infiltration [332, 333]. Other microorganisms also contribute to colorectal carcinogenesis. For instance, *Enterotoxigenic bacteroides* fragilis compromises the epithelial barrier by secreting bacterial toxins and activates the β‐catenin and c‐Myc pathways [332]. Conversely, *Enterococcus faecalis* bolsters anti‐tumor immunity by activating the NOD2 pathway through SagA peptidoglycan. Commendably, *Beneficial bacteria* like *Akkermansia muciniphila* and *Faecalibacterium prausnitzii* impede tumor advancement by modulating barrier function and mounting anti‐inflammatory responses.

Circulating tumor DNA is free fragmented DNA shed by tumors into the bloodstream, which carries tumor‐specific mutations and reflects tumor burden and molecular characteristics. Its detection rate in stages I–IV colorectal cancer patients has increased from 40% to over 90%. A positive result after surgery indicates an elevated risk of recurrence and can predict recurrence approximately 8 months earlier than imaging studies. Microsatellite high instability/mismatch repair deficiency is a crucial molecular feature that occurs in 6−13% of colorectal cancer patients. These patients have a high tumor mutation burden and respond well to ICI therapy [335]. MicroRNAs are involved in tumorigenesis and development through regulating target genes. miR‐155 is highly expressed in colorectal cancer tissues. It activates PI3K/AKT and NF‐κB pathways via targeting tumor suppressor genes such as *SOCS1*, *FOXO3a*, and *PTEN*, thereby promoting proliferation, invasion, and chemoresistance [336]. Its high expression is associated with advanced stages, lymph node metastasis, and poor prognosis. Others such as miR‐21 and miR‐34 also have significant values. Novel nucleic acid markers such as tRNA‐derived small RNAs show application potential.

CEA is the most widely used biomarker for colorectal cancer in clinical practice, but its sensitivity is limited and it is more suitable for monitoring treatment efficacy and early warning of recurrence. CA19‐9, CA50, CA72.4 etc. can be used as auxiliary diagnostic markers. Combined with CEA, they can improve the accuracy of diagnosis. Cytokines and chemokines play a key role in immune regulation and angiogenesis. CXCL8 (IL‐8) was elevated in both tissue and serum and associated with tumor stage, metastasis and poor prognosis [331]. The CXCL12/CXCR4/CXCR7 axis promoted metastasis by activating pathways such as PI3K/AKT and MAPK. High expression of CXCR4 was closely related to liver metastasis. CXCL13 promoted tumor progression and resistance to 5‐fluorouracil through the PI3K/AKT pathway. Other protein markers were also significant. The circulating level of FGF‐21 was significantly increased in patients with colorectal cancer. PIIINP, hyaluronic acid, TIMP1, etc. were involved in liver fibrosis and could be used to assess the risk of liver metastasis. S100 family proteins promoted tumor progression and metastasis by activating RAGE signaling [335].

Metabolic reprogramming is a hallmark of colorectal cancer [337]. SCFAs, derived from the fermentation of dietary fiber by gut microbiota, exhibit anti‐inflammatory and antitumor properties. Notably, their concentrations in the feces of colorectal cancer patients are markedly diminished. Specifically, butyrate modulates gene expression by inhibiting histone deacetylase and augments CD8^+^ T cell activity. Alterations in bile acid metabolism are also implicated in colorectal carcinogenesis. Deoxycholic acid can induce epithelial‐mesenchymal transition and angiogenic mimicry, thereby facilitating tumor progression, whereas ursodeoxycholic acid offers protection. Additionally, certain metabolites hold diagnostic potential. For instance, branched‐chain amino acids, synthesized by bacteria, bolster tumor proliferation and stemness through activation of the mTORC1 pathway. Tryptophan derivatives influence immune responses, enhancing antitumor efficacy. Trans‐3‐indoleacrylic acid has been shown to foster tumor survival via inhibition of ferroptosis. Moreover, glycolytic and fatty acid metabolites, such as lactic acid and succinic acid, are abundant in the tumor milieu and correlate with tumor invasion and drug resistance [333].

Circulating tumor cells are tumor cells that shed from the tumor tissue and enter the bloodstream. Their positive rate is related to the stage of colorectal cancer, with a detection rate of 60.7% in stage IV patients, while they are not detected in healthy individuals. They can be used as markers for tumor burden assessment and prognostic prediction [334]. Exosomes are nano‐scale vesicles secreted by cells, carrying proteins, microRNAs and other molecules. The quantity and contents of exosomes in the serum of patients with colorectal cancer change. For example, exosomal miR‐21 and miR‐92a can be used for diagnosis and prognostic evaluation. Fecal markers also have important application value. In addition to microorganisms, the combination of fecal DNA methylation detection and fecal immunochemical test combined with microbial markers can increase the early detection rate of colorectal cancer [332]. Multi‐omics fecal testing integrating microbial, genetic and epigenetic markers has become an important direction of non‐invasive screening.

In terms of long non‐coding RNA markers, the application of spatial transcriptomics technology provides a brand‐new perspective for the discovery of prognostic markers for colorectal cancer. A study utilized the 10× Genomics Visium spatial gene expression platform to conduct spatial transcriptomic analysis on tumor tissues, paired metastases and normal tissues of patients with colorectal cancer, and achieving precise identification of lncRNA expression profiles in malignant epithelial cell regions while preserving the spatial structure of the tissues [338]. By comparing the transcriptome characteristics of malignant regions with those of adjacent normal tissues, a total of 301 lncRNAs specifically upregulated in malignant regions were identified in the study. Cross‐validation was conducted using the public single‐cell and spatial transcriptome datasets (Shanghai cohort) and the TCGA‐COAD cohort. Further verification was conducted in an independent patient cohort by hybridization chain reaction RNA fluorescence in situ hybridization (HCR‐FISH) technology. It was found that *LINC01978*, *PLAC4* and *LINC01303* were almost not expressed in normal colonic epithelial tissues, but could be detected in stage II colorectal cancer tissues. And its expression level was significantly increased in the transferred tissues. It is worth noting that in the TCGA‐COAD cohort, the combined high expression characteristics of the three were significantly associated with a poorer overall survival, suggesting their potential as early risk assessment markers. Among them, the expression level of *LINC01303* in the microsatellite instability and CpG island methylation phenotypic subtypes was significantly higher than that in the chromosomal instability subtypes, demonstrating an association with molecular typing. From the perspective of functional mechanisms, *LINC01978* is related to glycolytic metabolic reprogramming and may be involved in the regulation of tumor energy metabolism. *PLAC4* is highly expressed in embryonic development‐related tissues, suggesting that it may be involved in the dedifferentiation process of tumor cells. *LINC01303* can participate in tumor progression by adsorbing microRNAs through sponges. These lncRNAs are located in the cytoplasm, suggesting that they may be packaged into extracellular vesicles and released into the blood, providing a potential possibility for liquid biopsy. This study demonstrated the unique advantages of spatial transcriptomics in precisely identifying tumor‐specific lncRNA markers, providing new candidate molecules for the early risk stratification and individualized treatment of colorectal cancer.

#### Current challenges and future development trends

Research on biomarkers for colorectal cancer is still faced with many challenges. The specificity and sensitivity of markers need to be improved. Most existing markers are insufficient in differentiating subtypes and tumors caused by different etiologies, and are easily interfered by intestinal inflammation, diet and lifestyle [331, 332, 333]. Genetic background, metabolic status and racial differences affect the expression and clinical value of markers, making it difficult to develop universal markers applicable to all patients [331]. Lack of standardized detection methods and thresholds leads to differences in detection techniques, sample processing methods and judgment criteria adopted by different studies, which makes it difficult to compare and promote results [331, 332]. Clinical transformation efficiency is relatively low. Most markers remain at the research stage and lack large‐scale, multicenter clinical validation [334]. Marker‐based Individualized treatment guidelines have not yet been formed [335].

Future research will advance in the directions of multi‐dimensional integration, precise application, technological innovation and clinical transformation. The first is multi‐omics integration and the development of composite markers. By combining multi‐dimensional data with machine learning algorithms, a composite marker panel can be constructed to enhance diagnostic and prognostic accuracy [333]. Research should emphasize subtype‐specific and individualized markers, developing specific markers for different molecular subtypes, metastasis sites and causes to meet the needs of precision medicine. Technological innovation and standardization in non‐invasive detection should be promoted, including optimizing liquid biopsy technology, developing convenient detection methods, and establishing standardized detection processes and thresholds. The application scenarios of markers should be expanded by developing markers for prognosis assessment, treatment response monitoring and drug resistance early warning to guide clinical decision‐making. International multi‐center cooperation and interdisciplinary integration should be strengthened to verify the clinical value of markers through large‐scale cohort studies, construct predictive models, and promote the implementation of new strategies for the precise secondary prevention and treatment of colorectal cancer.

### Hepatocellular cancer

Hepatocellular cancer (HCC), the predominant subtype of primary liver cancer, constitutes over 90% of all liver malignancies. Current screening combining ultrasound and alpha‐fetoprotein has a detection sensitivity of about 63% for early HCC, below clinical needs [339]. Biomarkers have emerged as crucial instruments for early detection, precise classification, therapeutic efficacy evaluation, and prognostication [340]. Recent advancements in multi‐omics technology, liquid biopsy, and artificial intelligence have paved new avenues in HCC biomarker research, propelling the diagnosis and management of liver cancer into a new era of precision medicine (Table S27) [341].

#### The development process of HCC biomarkers

The first stage is the traditional marker and imaging dominated period. In this phase, serum α‐fetoprotein and its derivatives are used as core markers, and diagnosis is made in combination with imaging techniques. As the first plasma marker applied to clinical practice, AFP has played a role in assisting HCC diagnosis, but its sensitivity is limited (47−64%) and specificity is insufficient [342]. The subsequent discovery of AFP heterogeneity and abnormal prothrombin somewhat compensated for the diagnostic needs of patients who were negative or low expression of AFP. In terms of imaging, CT and MRI can provide a morphological basis for tumor staging, but their ability to identify early microlesions is insufficient [341]. Imaging biomarkers derived from CT, PET, and MRI enable quantitative assessment of tumor morphology, metabolism, and heterogeneity. Radiomics extracts high‐dimensional features from medical images that correlate with tumor grade, lymph node metastasis, and molecular phenotypes such as PD‐L1 expression, offering a non‐invasive approach for tumor characterization and treatment response monitoring. Most biomarkers at this stage are based on single indicators, and their ability to distinguish benign liver diseases is limited [343]. There is an urgent need to improve diagnostic efficiency. With the breakthrough of omics technology, research on HCC biomarkers has entered a multidimensional and systematic exploration stage [344]. Proteomics identified tumor‐related proteins such as GPC3 and GP73. Among them, GPC3 promotes tumor proliferation by activating the Wnt pathway, and its exosome‐derived mRNA shows promise for early diagnosis [342]. Transcriptomics revealed that circulating microRNAs such as miR‐21, miR‐122, and miR‐1246 could form diagnostic panels with an AUC of 0.824–0.977 for detecting early HCC [340]. Epigenetic studies focused on ctDNA methylation biomarkers with a detection sensitivity of up to 87% [339]. Metabolomics and microbiomics further reveal the value of gut microbiota and their metabolites in predicting immunotherapy response. The integrated application of multi‐omics technologies has laid a solid foundation for constructing biomarker systems with high specificity and sensitivity [345]. Current research is dedicated to integrative analysis of multisource data and promoting clinical translation by integrating clinical features, imaging manifestations, and molecules [346].

#### The main types and typical cases of HCC biomarkers

Among protein biomarkers, AFP remains a central indicator for clinical screening. Changes in its levels can reflect the response to treatment. AFP‐L3 is associated with tumor malignancy and is a key component of the GALAD model [339]. DCP is involved in the process of tumor invasion, and its combination with AFP improves diagnostic accuracy [341]. GPC3 is highly expressed in tissues and serum, and its exosomal mRNA shows better diagnostic potential [342]. Among nucleic acid markers, ctDNA methylation has a sensitivity of 87% for early HCC detection [344]. The circulating miRNA panel effectively distinguishes HCC from healthy individuals, among which miR‐1246 predicts early postoperative recurrence. LncRNAs are involved in tumor progression by regulating the EMT process, and their combination with AFP enhances diagnostic specificity. Among cellular and composite markers, EpCAM + CTCs ≥ 2 indicate an increased risk of postoperative recurrence, and PD‐L1 + CTCs are associated with immunotherapy response [339]. The GALAD model has demonstrated excellent performance in multiple independent cohorts and is currently undergoing multicenter clinical validation.

Among the circulating markers, matrix metalloproteinases (MMPs)/tissue inhibitors of metalloproteinases (TIMPs) reflect extracellular matrix remodeling status and TIMP‐1 has potential prognostic value in HCC. Endothelial function indicators such as endothelin‐1 and vWF are related to cardiovascular complications. In terms of immune‐related markers, PD‐L1 expression level in tumor tissues is closely related to immunotherapy response, and PD‐L1 + CTCs can dynamically monitor therapeutic efficacy [341]. The density of CD8 + T cells in TILs was positively correlated with prognosis, and *hsa_circ_0064428* could be used as a potential predictor. Among the treatment response prediction markers, patients with high ABRS expression had significantly prolonged PFS after combined atezolizumab and bevacizumab treatment [347]. Enrichment of *Lachnospiraceae bacterium*‐GAM79 in gut microbiota indicated that patients benefited from immunotherapy, while enrichment of *Veillonellaceae* predicted poor outcomes. Exosomal proteins CCT8 and CFL1 were associated with venous invasion, and exosomal miR‐21‐5p and miR‐144‐3p showed better diagnostic performance than free miRNA [339].

#### Current challenges and future development trends

Research on HCC biomarkers is fraught with challenges. The marked heterogeneity of the disease, attributable to diverse etiologies and molecular subtypes, precludes the identification of a singular marker with comprehensive coverage. Moreover, traditional markers such as AFP exhibit inadequate specificity, as they may also elevate in benign liver diseases [348]. The clinical translation of novel markers remains challenging, as most are still undergoing validation and lack standardized protocols and large‐scale sample verification. Additionally, the sensitivity for early diagnosis is suboptimal, particularly for detecting very early HCC tumors <2 cm [344]. Variations across detection platforms further compromise result comparability due to low standardization levels [346].

Future research will focus on the following directions. Promote refined typing and construct etiology‐ and molecular feature‐based specific marker panels; Strengthen technology‐driven approaches to discover novel markers through new technologies such as multiomics integration, AI algorithm optimization, and spatial transcriptome [349]. Improve integrated surveillance systems, build multidimensional models by combining clinical, imaging, and molecular data, and achieve dynamic monitoring through liquid biopsy. Accelerate the coordinated development of targeted drugs and markers; Expand the application of early detection technologies in multiple tumor types [345]. Meanwhile, efforts should be made to establish standardized testing procedures, promote multicenter validation, optimize cost‐effectiveness, and ultimately realize the comprehensive transformation of biomarkers from basic research to clinical practice, improving the survival outcome of HCC patients [350].

### Pancreatic cancer

Pancreatic ductal adenocarcinoma (PDAC), which accounts for over 90% of pancreatic cancer cases, is characterized by its high aggressiveness and extremely poor prognosis, with a 5‐year survival rate of less than 10% [351]. Although the current primary serum marker, CA19‐9, is widely used in clinical monitoring, its early diagnostic sensitivity and specificity are suboptimal due to the Lewis antigen‐negative phenotype (approximately 10%) and interference from benign diseases [349]. Consequently, the development of high‐precision biomarkers has emerged as a critical challenge for achieving early screening, accurate diagnosis, effective treatment, and improved prognosis of pancreatic cancer (Table S28) [352].

#### The development process of biomarkers

Research on pancreatic cancer biomarkers has evolved through three pivotal stages, mirroring advancements in disease understanding and technology [351]. During the initial phase, there was a predominant emphasis on validating protein markers. CA19‐9, recognized as the first serum marker endorsed by the FDA, remains extensively utilized for auxiliary diagnosis and assessing treatment efficacy. Nonetheless, its inherent performance constraints have spurred the quest for novel markers [353]. Concurrently, *KRAS* mutations emerged as a principal driving factor, boasting a mutation rate surpassing 90%, thereby establishing a molecular basis for targeted therapeutic investigations [352]. The subsequent expansion phase witnessed a diversification propelled by mass spectrometry and sequencing innovations [351]. This era saw the discovery of new protein markers, including CA242 and C4BPA, while non‐coding RNAs, such as Pra‐162 and 725, facilitated early diagnosis via liquid biopsy. The research paradigm transitioned from individual molecules to multi‐indicator combinations. Notably, the combined panel of LRG1 + TIMP1 + CA19‐9 markedly amplified diagnostic accuracy [353]. The current integration and transformation phase is distinguished by multi‐omics consolidation and clinical application. composite marker panels, aided by machine learning techniques like *APOE* + *ITIH3* + *APOA1* + *APOL1* + CA19‐9, exhibit a diagnostic sensitivity of 95% and a specificity of 94.1% [351]. Furthermore, Exosome‐derived markers, including GPC1 and EPHA2, hold promise for early detection and predicting drug resistance due to their tissue‐specificity and minimally invasive nature [352].

#### The main types of biomarkers and typical cases

CA19‐9, a tumor burden‐associated carbohydrate antigen, is used for diagnosis, therapeutic effect monitoring and recurrence warning. However, its sensitivity and specificity are limited and it has no diagnostic value in Lewis antigen negative individuals. CEA and CA125, as auxiliary markers related to tumor invasion and abdominal metastasis respectively, are often combined with CA19‐9 to improve the risk assessment ability. Among new protein markers, C4BPA shows abnormal expression in early pancreatic cancer and has tissue differentiation ability. Urinary NGAL has unique value in early screening of non‐jaundiced patients [353]. CEACAM1 has been found abnormally expressed in PanIN, suggesting that it may be useful in monitoring precancerous lesions. In terms of treatment guidance, high OPN expression is associated with poor survival of patients, and its cutoff value (>102 ng/mL) is relatively high in differentiating pancreatic cancer from chronic pancreatitis. SPARC, a key protein for matrix remodeling, whose expression level can predict the efficacy of albumin‐bound paclitaxel [352].

*KRAS* mutation, the most common genetic alteration in PDAC, mediates activation of signaling pathways such as MAPK and promotes tumor proliferation and metabolic reprogramming. Its specific mutant subtypes have become a basis for screening targeted drugs [354]. Deletions of *SMAD4* and *CDKN2A*/p16 are respectively involved in tumor metastasis and cell cycle dysregulation, serving as important molecules for prognostic stratification and subtype identification. Among non‐coding RNAs, NIR‐162,725, NIR‐366,845, etc. influence tumor behavior by modulating signaling pathways such as MAPK and Hippo [317]. Among them, NIR‐017061 can inhibit the expression of EFNA5 and has potential therapeutic value. LncRNAs such as *HOTAIR* and *GAS5* participate in tumor progression by regulating processes like EMT and autophagy [355].

To overcome the performance limitations of a single marker, multiple composite panels have been developed and validated. The combined model of LRG1 + TIMP1 + CA19‐9 has enhanced the ability to identify early lesions. The urine panel of LYVE1 + REG1A + TFF1 possesses non‐invasive and easy‐to‐operate advantages, making it suitable for population screening. In terms of exosomal markers, GPC1 protein exhibits a diagnostic performance close to 100%, EPHA2 can predict gemcitabine resistance, and exosomal lncrnas such as *LINC01133* promote tumor invasion by regulating the Wnt/β‐catenin pathway [356].

Pancreatic intraepithelial neoplasia, intraductal papillary mucinous neoplasm and mucinous cystic neoplasm are important precursor lesions of pancreatic cancer [357]. The abnormal expressions of ANXA10 and TFF2 respectively indicate the risk of malignant transformation in PanIN and IPMN. In MCN, a combination of MUC5AC + MUC2 + MUC1 can distinguish invasive subtypes, while the expression of progesterone receptor is diagnostically specific. *KRAS* and *GNAS* mutations are also common in IPMN and can be used as auxiliary indicators for assessing the risk of malignancy [352].

#### Current challenges and future development trends

Research on biomarkers for pancreatic cancer continues to face numerous challenges. First, the specificity and sensitivity of these markers are inadequate, complicating the differentiation between benign lesions like pancreatic cancer and chronic pancreatitis. Second, due to high tumor heterogeneity, marker expression varies considerably among patients with differing molecular subtypes, races, and stages [352]. Third, there is a notable absence of standardization in detection techniques; methods such as exosome isolation and mutation detection remain non‐uniform. Fourth, clinical translation is delayed, with most markers yet to undergo large‐sample multi‐center validation. Fifth, an application system for precancerous lesion markers has not been established, hindering early and precise intervention [352].

Future research on biomarkers for pancreatic cancer is expected to exhibit a trend toward multi‐dimensional and coordinated development. By integrating multi‐omics data, including proteomics, genomics, and metabolomics, and leveraging artificial intelligence and machine learning algorithms, composite diagnostic models with enhanced accuracy can be constructed [358]. In line with the principles of precision medicine, this approach will facilitate the development of individualized monitoring markers for specific gene mutations, such as *KRAS* ^G12C^, and the establishment of dynamic risk assessment systems for the transformation of precancerous lesions, thereby providing a foundation for early intervention [352]. Technological innovations will drive the evolution of detection methods toward convenient, low‐cost formats, such as microfluidic chips and biosensors, while also promoting the standardization of detection processes. Consequently, the application of these biomarkers will expand from diagnosis to encompass the entire disease management process, including efficacy evaluation, drug resistance prediction, and immunotherapy screening. Ultimately, through international multi‐center collaboration and large‐scale clinical validation, the translation of biomarker discoveries from basic research into clinical practice will be accelerated, providing systematic support for the early screening, precise treatment, and improved prognosis of pancreatic cancer [353].

### Cholangiocarcinoma

Cholangiocarcinoma (CCA) is a highly heterogeneous malignant tumor originating from the biliary epithelium. Current imaging and serological tests have limited early detection ability, while treatment remains constrained by the efficacy bottlenecks of radiotherapy and chemotherapy [359]. Therefore, developing biomarkers with high sensitivity and specificity is crucial for achieving early screening, precise diagnosis, prognostic assessment, and targeted therapy (Table S29) [360].

#### The development process of biomarkers for cholangiocarcinoma

The era of traditional markers and imaging detection. Early studies focused on serum protein markers and imaging techniques. CA19‐9 and CEA, as classic serum markers, are widely used for auxiliary diagnosis [360]. However, CA19‐9 can also be elevated in benign biliary diseases, and false negatives are prone to occur in Lewis antigen‐negative individuals. CEA may increase in various diseases, so its value is limited when used alone. It is often combined with CA19‐9 to improve the discrimination ability. Although imaging techniques such as CT and MRCP can evaluate the degree of obstruction and tumor extent, it is difficult to effectively distinguish early malignant lesions from benign stenosis [359, 361]. This stage lays a clinical foundation for biomarkers but is limited by insufficient sensitivity and specificity, making early diagnosis impossible.

Multi‐omics technology‐driven exploration phase [362]. With the development of multi‐omics technologies such as proteomics, transcriptomics, and metabolomics, CCA marker research has entered a systematic exploration stage. Proteomics reveals that CYFRA21‐1 has a diagnostic sensitivity of 75.6% and a specificity of 96.2% for iCCA, which is superior to CA19‐9 [363]. MMP‐7 promotes invasion by degrading extracellular matrix, and its level is related to postoperative recurrence. In terms of nucleic acids omics, miR‐21 and miR‐155 are highly expressed in serum and bile. Among them, miR‐21 regulates *PTEN* and *PDCD4* to promote tumor proliferation and chemotherapy resistance. ctDNA can detect driver mutations such as *KRAS*, *IDH1/2*, and *FGFR2* [360]. Its detection rate in advanced patients is approximately 90%, and the frequency of variant alleles positively correlates with tumor burden. The metabolomic study identified *IDH1/2* mutation‐related metabolite 2‐hydroxyglutaric acid (2‐HG), which can be used as a biomarker for both mutation status and therapeutic response [360].

#### The main types and typical cases of biomarkers for cholangiocarcinoma

Such biomarkers are designed to identify high‐risk groups at an early stage and distinguish benign from malignant disease. In addition to CA19‐9 and CEA, CYFRA21‐1, a fragment of cytokeratin 19, was significantly increased in the serum of patients with iCCA but not in benign lesions [364]. Its diagnostic performance is superior to that of CA19‐9. MUC4 and MUC5AC also showed good diagnostic potential. The specificity of biliary detection of MUC4 for differentiating malignant stenosis reached 93%, and the serum level of MUC5AC was associated with poor prognosis [363, 364]. Among nucleic acid markers, miR‐200c‐3p was highly expressed in bile from CCA patients, with a diagnostic specificity of 87%. miR‐155 promoted tumor invasion through the STAT3 pathway and was a specific marker of inflammation‐related iCCA [362]. ctDNA could detect mutations such as *KRAS*, *IDH1/2*, and *FGFR2*. Its detection rate in plasma increased with the stage. Although it was difficult to detect in early cases, combined methylation analysis could significantly improve sensitivity, and the specificity of differential diagnosis between benign and malignant lesions was close to 100% [363, 365]. Regarding metabolite markers, 2‐HG, as a characteristic metabolite of IDH‐mutated CCA, had a sensitivity of 83% and a specificity of 90% when diagnosing IDH‐mutated CCA based on its serum level, and it could also monitor the response to targeted therapy in real time [359]. The AUC of lipid metabolism panels for differentiating iCCA from hepatocellular carcinoma exceeded 0.90, which was superior to traditional AFP and CA19‐9 [365].

Such biomarkers are used to assess disease progression and treatment response. Osteopontin is involved in cell adhesion and migration, its serum level is significantly elevated in CCA patients, and the preoperative level can predict recurrence risk. IL‐6, as a key inflammatory factor in the tumor microenvironment, has a diagnostic sensitivity of 73% and specificity of 92% [359]. A high level indicates poor prognosis. At the nucleic acid level, *FGFR2* fusions were observed in 10−15% of iCCA patients [363]. The carriers respond well to FGFR inhibitors such as pemigatinib, with an ORR of 40−42%, and their median survival time was significantly prolonged [362, 363]. *IDH1* mutation occurs in 13−20% of iCCA patients. Treatment with ivosidenib prolonged the median PFS from 1.4 months to 2.7 months, and long‐term responders (treatment ≥ 1 year) had a 2‐year OS rate of 92.1% [364]. The frequency of variant alleles in ctDNA (VAF) has significant prognostic value. The median OS of those with VAF > 3.9% was only 4.9 months. As cellular markers of tumor aggressiveness, CTC counts of ≥2 indicate poor prognosis [365]. Combining CTC counts with ctDNA analysis could enhance the efficacy of minimal residual disease monitoring (AUC = 0.92) [364]. In addition, serum MMP‐7 levels were associated with acute exacerbation frequency and all‐cause mortality. Patients with *BRAF* ^V600E^ mutations treated with dabrafenib plus trametinib achieved an ORR of 47%, providing a new therapeutic option for this subgroup [364].

#### Current challenges and future development trends

Research on CCA biomarkers still faces multiple challenges. First, tumor heterogeneity is strong. iCCA is mainly characterized by *IDH1* mutation and *FGFR2* fusion, while pCCA/dCCA are more often seen with HER2 amplification and *KRAS* mutation [364]. A single marker has difficulty in comprehensively characterizing the nature of the disease. Second, the specificity of markers is insufficient. The false positive rate of CA19‐9 in bile duct obstruction exceeded 50%, and other markers such as miR‐21 can also be seen in various digestive tract tumors [366]. Third, clinical transformation is difficult. Most candidate substances are still in the validation stage. Only CA19‐9, IDH1 and FGFR2 related tests have entered routine application [363, 364]. Insufficient standardization and lack of multicenter data limit their promotion. Fourth, there is a lack of early diagnostic markers. existing indicators have low sensitivity to early lesions and partially rely on invasive samples, limiting their screening applications [364].

Future research on cholangiocarcinoma biomarkers will likely emphasize precision, technology‐driven approaches, and integrated dynamic monitoring [367]. This includes promoting individualized diagnosis and treatment through molecular typing, deepening the clinical application of existing markers such as IDH1 and FGFR2, and expanding rare operable targets like RET and NTRK [368]. By leveraging multi‐omics integration and artificial intelligence modeling, the efficiency of marker discovery can be enhanced. Additionally, combining non‐invasive technologies such as exhaled volatile organic compound analysis and microfluidic chips can lower the clinical threshold [363, 367]; Concurrently, a dynamic monitoring system based on liquid biopsy should be developed. regular detection of markers like ctDNA and CTCs enables real‐time assessment of tumor burden and treatment response, establishing a clinical closed loop of “screening‐diagnosis‐treatment‐monitoring” [368]. The overarching objective is to devise a highly sensitive screening strategy for high‐risk groups, develop disease progression prediction models for early intervention, and bolster treatment success rates through biomarker‐guided targeted drug research and development, thereby systematically improving patient prognoses in cholangiocarcinoma cases (Figure S3) [367, 368].

### Multilevel network integration of biomarkers for digestive system diseases

**FIGURE 2:**
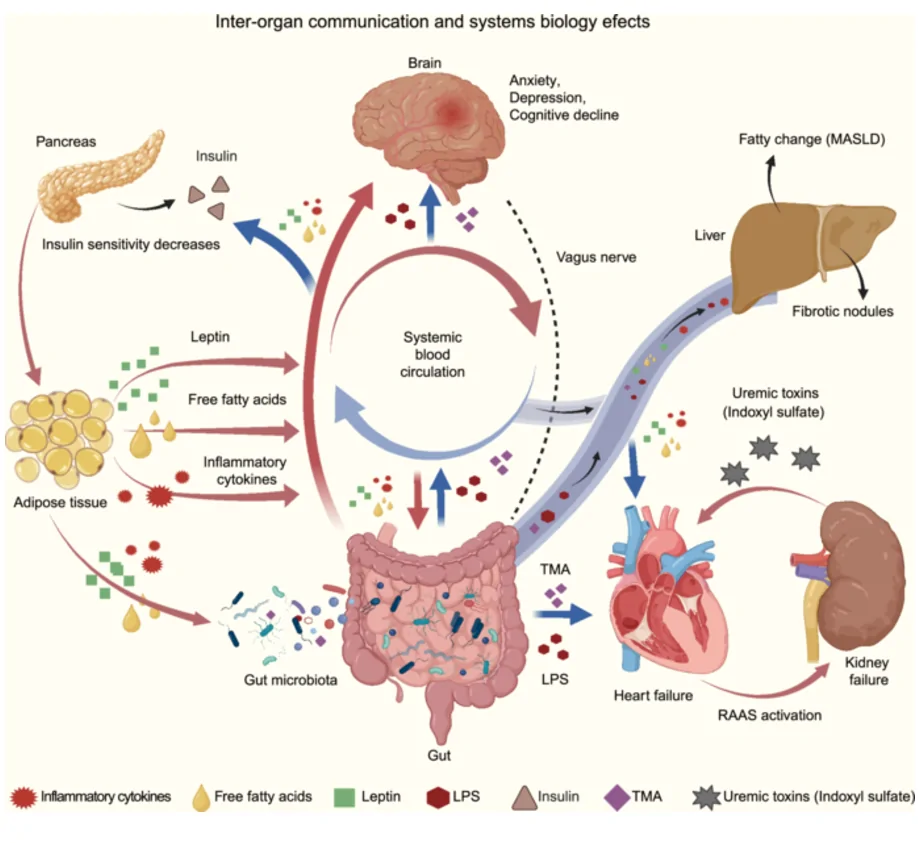
Inter‐organ communication and systems biology effects. This figure explains the systemic biological mechanism by which local organ lesions affect distant organs and lead to “comorbidity” through the blood circulation system as the core medium. In the figure, four classic dialog axes are taken as examples to intuitively show the systematic interaction: Gut–liver axis: metabolites of gut microbiota TMA and endotoxin LPS enter the liver through the portal vein, driving or aggravating MASLD and liver fibrosis. Gut–brain axis: Gut microbiota and its metabolites (TMA, LPS) affect brain function through circulatory system and neural pathways, and are associated with anxiety, depression and cognitive decline. Fat–organ axis: Adipose tissue affects systemic insulin sensitivity, cardiovascular function and multiple organ metabolism by releasing signal molecules such as leptin, free fatty acids and inflammatory factors to the circulatory system. In addition, insulin sensitivity can also reversely affect the function and metabolic state of adipose tissue. Heart–kidney axis: heart failure leads to insufficient renal perfusion and activates the RAAS; on the contrary, water and sodium retention and uremic toxins accumulation caused by renal failure will aggravate cardiac load and form a vicious circle.

As the largest endocrine and immune organ in the human body, the digestive system is characterized by a biomarker network characterized by unique spatiotemporal heterogeneity and dynamic evolution. This section describes the establishment of a multilevel integration framework from molecules to systems through the systematic analysis of marker lineages within key digestive organs, including the liver, gallbladder, pancreas, and intestine (Table S30 and Figure 2) [369].

#### Molecular cascade reaction network of the liver injury process

Hepatocyte injury triggers a complex and orderly series of molecular cascades. Various stages of injury are accompanied by dynamic changes in specific biomarkers, providing a crucial basis for disease assessment [370]. During the initial stage of injury, alterations in hepatocyte membrane permeability lead to a substantial release of cytoplasmic alanine aminotransferase (ALT) and aspartate aminotransferase (AST) into the bloodstream, serving as sensitive indicators of early mild hepatocellular damage. As injury intensifies, mitochondrial architecture becomes disrupted, resulting in a significant increase in the release ratio of mitochondrial‐derived AST (m‐AST), signifying more severe energy metabolism disturbances within hepatocytes and involvement at the organelle level. During the progression of chronic injury, cytokeratin 18 (CK‐18) fragmentation occurs, indicating that CK‐18 is a characteristic marker for initiating hepatocyte apoptosis. The ratio between CK‐18 fragments and ALT levels effectively distinguishes simple steatosis from nonalcoholic steatohepatitis, offering an important reference for pathological classification [371]. Notably, the expression of miR‐122, a liver‐specific microRNA, significantly increased during the early phase of drug‐induced liver injury. Its dynamic alterations precede traditional enzymatic markers such as ALT and AST, enabling earlier detection of ultra‐early hepatocyte damage signals. This provides a valuable time window for implementing precise clinical interventions and reducing the risk of progressive liver injury [372].

#### The mechanism underlying the reprogramming of the fibrosis microenvironment

Hepatic fibrosis is a dynamic pathological process characterized by excessive extracellular matrix deposition during the chronic progression of hepatocyte injury and involves the coordinated regulation of multiple cell types. The core mechanisms include the activation of hepatic stellate cells from their resting state, the capillarization of hepatic sinusoidal endothelial cells, and the polarization of Kupffer cells with subsequent secretion of profibrotic factors. The interactions among these three processes collectively promote the fibrotic cascade. Common clinical markers are classified into two categories: direct and indirect. Direct markers, such as hyaluronic acid, are primarily synthesized and released by activated hepatic stellate cells, directly reflecting the imbalance between extracellular matrix synthesis and degradation. In contrast, indirect biomarkers, including thrombocytopenia and an elevated APRI score, reflect secondary pathological changes such as portal hypertension and hypersplenism, thus assisting in the assessment of disease severity. Recent research has revealed that exosomal miR‐21 and miR‐29b can target and regulate the activation and proliferation of hepatic stellate cells via paracrine signaling, offering a novel perspective and potential targets for noninvasive monitoring and targeted intervention in liver fibrosis [373].

#### Multiomics evolution characteristics during the carcinogenesis process

The occurrence and development of hepatocellular carcinoma are accompanied by the dynamic evolution of multiomics characteristics, including the genome, transcriptome, and epigenome, and are closely associated with precancerous lesions such as liver fibrosis [374]. At the precancerous stage, tumor‐specific molecular changes begin to emerge. An abnormally elevated proportion of the alpha‐fetoprotein variant L3 reflects aberrant glycosylations in hepatocytes and serves as a significant molecular marker for early screening. Concurrently, the detection rate of *TERT* promoter mutations in circulating tumor DNA progressively increases as the lesion advances from a precancerous state to malignancy, establishing it as a key genomic marker that reflects the clonal evolution of the tumor [375]. In the advanced stage, epigenetic dysregulation in liver cancer becomes more pronounced. Aberrant METTL3‐mediated m6A methylation is detectable in the plasma of patients and is positively correlated with tumor invasiveness, metastatic capacity, and poor prognosis through its regulation of oncogene expression [376]. The combined detection of such epigenetic markers with specific protein markers, such as GP73, can lead to the establishment of a multidimensional and highly sensitive liver cancer risk prediction model, providing precise clinical references for early diagnosis, progression monitoring, and prognostic assessment of the disease.

### The digestive system serves as the central hub of metabolism–immune–microbiota

The digestive system engages in bidirectional communication with various bodily systems via a complex axial network, thus functioning as a central hub for the integration of cross‐system biomarkers (Table S31).

#### The precise regulatory network of the gut–liver–immune axis

The liver and intestine establish a mutually regulated feedback system, termed the liver–intestinal axis, through portal vein access and bile acid circulation, thus maintaining metabolic homeostasis. The intestinal microbiota, as a key regulatory factor, metabolizes dietary fiber to produce SCFAs. Among these, butyric acid plays a particularly prominent role. It serves as the preferred energy source for colonic epithelial cells, promotes mucosal repair, enhances barrier integrity, and enters the liver via the portal vein. It regulates hepatocyte function by modulating the expression of lipid and glucose metabolism‐related genes in liver cells while inhibiting excessive activation of Kupffer cells in the liver and alleviating the underlying inflammatory response [377]. When the intestinal barrier is compromised because of factors such as dietary imbalance and flora disorders, lipopolysaccharide (LPS) can penetrate the mucosal barrier and enter the bloodstream. Upon reaching the liver via the portal vein, it activates the TLR4 signaling pathway, triggering a downstream inflammatory cascade. This not only induces insulin resistance in hepatocytes but also aggravates the inflammatory response of the liver parenchyma. In the long term, it can develop into nonalcoholic fatty liver disease and other lesions [378]. Elevated serum catenin levels can indicate increased intestinal permeability at an early stage. Fatty acid‐binding protein 2 (FABP2) can reflect intestinal mucosal damage and abnormal lipid absorption and is an early warning indicator of abnormal intestinal barrier function [379]. The disorder of bile acid spectrum not only reflects an imbalance in hepatoenteral circulation but also has reverse effects on the composition of the intestinal flora, forming a vicious cycle and further aggravating hepatointestinal function disorders.

#### Bidirectional dialog mechanism of the gut–brain–microbiota axis

The dorsal nucleus of the vagus nerve and the nucleus tractus solitarius serve as central integration nodes within the gut–brain axis. These structures receive mechanical stimuli from the intestine and chemical signals, including metabolic products and nutrients derived from the microbiota. Through neural pathways, they facilitate bidirectional regulation between the gut and the brain [199]. The intestine, recognized as the largest peripheral neuroendocrine organ, contains endocrine cells responsible for synthesizing approximately 95% of the body's serotonin. This neurotransmitter plays a pivotal role in regulating gastrointestinal motility, secretion, and mucosal barrier function. Additionally, it projects to the central nervous system via vagus nerve afferent fibers, influencing mood, appetite, and cognitive functions, thus establishing a crucial molecular bridge for gut–brain signal transmission [380]. Clinical research has substantiated the pathophysiological relevance of the microbiota–gut–brain axis. Specifically, a significant positive correlation exists between the proportion of beneficial bacteria such as *Lactobacillus* and *Bifidobacterium* in the feces of patients with irritable bowel syndrome and the plasma level of BDNF, which is essential for neuronal survival and synaptic plasticity. Abnormal BDNF levels are frequently associated with emotional disorders and gastrointestinal dysfunction [381]. Consequently, the combination of high‐throughput sequencing of the fecal microbiota and the detection of serum brain–gut peptides can be used to accurately identify the association between microbiota imbalance and neuroendocrine disorders. This approach paves the way for the precise classification and individualized treatment of functional gastrointestinal diseases.

#### System integration effect of the digestive–metabolic–vascular axis

Nonalcoholic fatty liver disease, a typical manifestation of metabolic syndrome in the liver, involves the formation of an intricately interconnected pathological network with metabolic disorders in multiple organs, such as adipose tissue and blood vessels, during development [382]. Adiponectin, which is secreted by adipose tissue, serves as a key metabolic regulatory factor that effectively inhibits hepatic gluconeogenesis and de novo fatty acid synthesis through activation of AMPK signaling, thus maintaining lipid homeostasis in the liver. Conversely, FGF‐21, which is secreted by the liver, targets and regulates adipose tissue metabolism, promotes the conversion of white fat to thermogenic brown fat, and accelerates energy expenditure. When the adiponectin‐FGF‐21 metabolic axis becomes imbalanced, impaired lipid clearance in the liver leads to substantial accumulation, inducing hepatocellular steatosis [383]. Damaged hepatocytes secrete increased amounts of VEGF and platelet‐derived growth factor (PDGF). The former induces the capillarization of hepatic sinusoidal endothelial cells, thus disrupting liver microcirculation. Conversely, the latter promotes the activation of hepatic stellate cells, intensifying liver fibrosis and portal hypertension. Serum FGF‐21 levels and carotid IMT are positively correlated in clinical research. These findings directly illustrate the systemic integration effect of the digestive–metabolic–vascular axis, providing theoretical support for collaborative intervention strategies in cross‐organ metabolic diseases.

#### The biomarker value of the microbial–host co‐metabolic network

**FIGURE 3:**
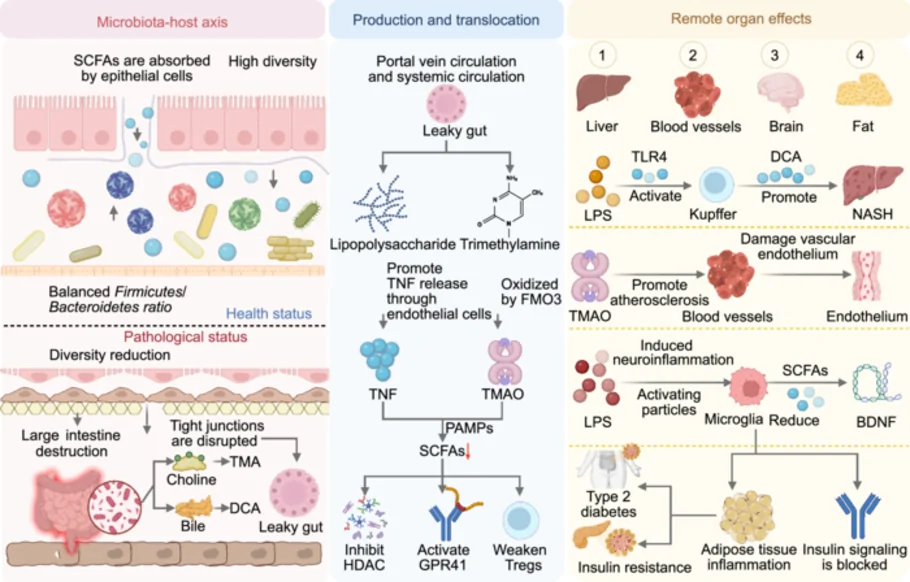
Schematic of the integrated effects of the gut microbiota on host metabolism and distant organ effects. This figure illustrates how gut microbiota composition, microbial metabolites and intestinal barrier integrity regulate host metabolism and systemic organ function. Under healthy conditions, high microbial diversity and a balanced *Firmicutes/Bacteroidetes* ratio promote SCFA production and absorption by intestinal epithelial cells, supporting intestinal homeostasis. Under pathological conditions, reduced microbial diversity, disruption of tight junctions and intestinal injury increase gut permeability, leading to a “leaky gut” state. Dysbiosis alters the metabolism of choline and bile acids, TMA and DCA, while facilitating the translocation of LPS, PAMPs and other microbial products into the portal and systemic circulation. TMA is oxidized by FMO3 to TMAO, whereas reduced SCFA levels affect immune regulation through HDAC inhibition, GPR41 activation and regulatory T‐cell modulation. Gut‐derived metabolites and inflammatory signals further influence distant organs: LPS activates hepatic Kupffer cells via TLR4 and promotes NASH; TMAO contributes to atherosclerosis and endothelial injury; LPS‐associated inflammatory particles activate microglia and induce neuroinflammation; and impaired SCFA signaling promotes adipose inflammation, insulin resistance, blocked insulin signaling and type 2 diabetes.

A highly synergistic co‐metabolic network exists between the human body and its intestinal microbiota. The metabolic byproducts of this microbiota serve as crucial links connecting the digestive system with systemic diseases [384]. Specifically, trimethylamine, produced from the metabolism of choline, carnitine, and other substances by these microorganisms, enters the liver via the portal vein. It is oxidized by flavin monooxygenase 3 (FMO3) to form trimethylamine‐n‐oxide (TMAO). This metabolite promotes the onset and progression of atherosclerosis by inhibiting reverse cholesterol transport and inducing excessive platelet activation [385]. In a similar fashion, secondary bile acids such as deoxycholic acid, whose production is mediated by the intestinal microbiota, exhibit dual functionality. They facilitate the emulsification and absorption of lipids in the intestine and serve as endogenous ligands for the farnesoid X receptor (FXR), thus activating downstream signaling pathways that regulate glucose homeostasis, lipid metabolism, and intestinal mucosal immunity with high precision. These significant microbe–host co‐metabolic markers elucidate the intrinsic molecular connections between the digestive system and cardiovascular and metabolic diseases, offering novel molecular targets and a theoretical foundation for the early detection and targeted treatment of cross‐system diseases (Figure 3) [386].

## ENDOCRINE AND METABOLIC SYSTEM

Literature searches for the endocrine and metabolic systems were carried out in PubMed and Web of Science using Boolean logic with field‐restricted search strings. The specific Boolean queries were constructed as follows: for diabetes mellitus, (“diabetes mellitus”) AND (“biomarker” OR “biomarkers” OR “biological marker” OR “biological markers”); for thyroid dysfunction, (“thyroid dysfunction” OR “thyroid function disorder”) AND (“biomarker” OR “biomarkers” OR “biological marker” OR “biological markers”); for metabolic syndrome, (“metabolic syndrome”) AND (“biomarker” OR “biomarkers” OR “biological marker” OR “biological markers”); for thyroid carcinoma, (“thyroid cancer” OR “thyroid carcinoma”) AND (“biomarker” OR “biomarkers” OR “biological marker” OR “biological markers”); for pituitary adenoma, (“pituitary tumor” OR “pituitary adenoma”) AND (“biomarker” OR “biomarkers” OR “biological marker” OR “biological markers”); for adrenocortical carcinoma, (“adrenocortical carcinoma”) AND (“biomarker” OR “biomarkers” OR “biological marker” OR “biological markers”); and for pancreatic neuroendocrine neoplasms, (“pancreatic neuroendocrine tumor” OR “pancreatic neuroendocrine neoplasm” OR “pNET”) AND (“biomarker” OR “biomarkers” OR “biological marker” OR “biological markers”). The retrieval period was defined as January 1, 2020 through December 31, 2025. Only articles published in English were considered. Both original research articles and systematic or narrative reviews were included, with systematic reviews and meta‐analyses prioritized to enhance the evidence level and clinical reliability of the included biomarkers. Retrieved records were screened by titles and abstracts, and articles deemed irrelevant to endocrine and metabolic disease biomarkers or lacking complete content were excluded, with the remaining full texts subjected to thorough evaluation. The included biomarkers were required to correspond to at least one of three clinical application scenarios, namely diagnosis, prognostic evaluation, or therapeutic efficacy monitoring, with explicit detection methods, cutoff values, or definitive clinical significance. The selected disease spectrum encompasses diabetes mellitus, thyroid dysfunction, metabolic syndrome, thyroid carcinoma, pituitary adenoma, adrenocortical carcinoma, and pancreatic neuroendocrine neoplasms, all representing conditions with high prevalence, substantial disease burden, well‐established biomarker frameworks, and strong clinical translational relevance.

### Diabetes mellitus

DM is a group of metabolic diseases characterized by chronic hyperglycemia. The condition results from insulin secretory deficiency or insulin resistance. Clinical subtypes include T1D and T2D and LADA [387]. Disorders of glucose metabolism lead to microvascular and macrovascular complications including diabetic nephropathy and retinopathy and cardiovascular events. These pathologies represent leading causes of end‐stage renal disease and visual loss and non‐traumatic amputation [388, 389]. LADA is prone to be misdiagnosed as T2D due to its heterogeneous clinical manifestations, and its accurate identification and management strongly depend on specific biomarkers (Table S32) [387, 390].

#### The development process of DM biomarkers

The development of DM biomarkers is closely linked to the deepening understanding of diseases and advancements in detection technologies. Early diagnosis primarily relied on fundamental indicators such as blood glucose, urine glucose, glycated hemoglobin (HbA1c), and urinary albumin. HbA1c, reflective of average blood glucose levels over a span of 2–3 months, serves as the gold standard for both diagnosis and disease monitoring [389, 391]. As immunological techniques evolved, pancreatic islet autoantibodies like glutamic acid decarboxylase antibody (GADA) and tyrosine phosphatase IA‐2 antibody emerged as crucial tools for distinguishing autoimmune diabetes, thereby significantly enhancing the diagnostic precision of subtypes such as LADA [387, 390].

Recently, the advent of omics technologies has propelled biomarker research into the realm of precision medicine. The urine peptide classifier CKD‐273, developed through proteomics, amalgamates 273 distinct urine peptides and is capable of predicting diabetic nephropathy risk 5 years prior to any clinical indicator abnormalities [388]. In the field of metabolomics, serum metabolites such as leucine and dihydrosphingosine have been identified, which can effectively differentiate between diabetic patients and healthy individuals. Furthermore, metabolites like 3‐hydroxyisobutyric acid offer a more precise indication of early renal function decline trends [388]. Additionally, non‐coding RNAs sourced from exosomes are emerging as significant tools for delving into the mechanisms of DM and its complications, as well as for early diagnosis, given their potential role in modulating pancreatic β‐cell function and insulin resistance [392, 393].

#### The main types and typical cases of DM biomarkers

Among the diagnostic biomarkers, HbA1c is a key diagnostic indicator. The HbA1c assay was first approved by the U.S. FDA in 2013 for monitoring glycemic control [394]. A value of more than 6.5% has been adopted by the WHO as the diagnostic threshold for DM, and every 1% reduction can reduce the risk of microvascular complications by 33% [389, 391]. In the differential diagnosis of DM subtypes, GADA is the key to identifying autoimmune diabetes, with a positive rate of up to 70−90% in LADA. High titers often indicate more severe insulin secretion deficiency. Combined with tyrosine phosphatase IA‐2 antibody detection, it can further improve diagnostic specificity [387, 390]. Exosomal non‐coding RNAs provide new approaches for early diagnosis. Serum exosomal miR‐204 was highly expressed before clinical onset of T1D, reflecting early beta cell damage [392]. Urinary exosomal miR‐192 was significantly increased in patients with T2D and early kidney disease and could identify individuals at high risk of nephropathy during negative urinary albumin stage [392, 393]. The urinary peptide classifier CKD‐273 had good performance in predicting the occurrence of albuminuria in patients with T2D and was valuable in stratifying the risk of kidney disease in patients with normal urinary albumin [291].

For prognosis evaluation, C‐peptide, as the core indicator for evaluating pancreatic β cell function, is closely related to disease progression. Patients with lower levels have a significantly increased risk of insulin dependence within 10 years [390, 395]. Among inflammation‐related markers, soluble tumor necrosis factor receptor 1/2 was significantly associated with diabetic nephropathy prognosis. Patients with elevated receptor levels had an increased risk of progressing to end‐stage renal disease [291]. Metabolomics markers also showed good prognostic value. The level of 3‐hydroxyisobutyric acid was negatively correlated with the rate of decline in glomerular filtration [291]. Regarding cardiovascular complications, exosomal miR‐320 aggravated myocardial microvascular disorders by inhibiting insulin‐like growth factor 1 and heat shock protein 20. Patients with high expression had a significantly increased risk of heart failure within 5 years [392, 393].

In terms of biomarkers for therapeutic effect monitoring, HbA1c remains the core monitoring indicator for blood glucose control. Maintaining an appropriate level during treatment can effectively reduce the risk of complications [389, 391]. In evaluating the therapeutic effect on diabetic nephropathy, urinary NGAL is highly valuable. A decrease in this indicator after SGLT2 inhibitors treatment indicates improvement in renal tubular injury and a significant reduction in the risk of end‐stage renal disease [291]. Exosomal markers have advantages in monitoring local complications. After the treatment of diabetic foot ulcers, increased exosomal miR‐126 in wound exudate suggests a shorter healing time, while no significant change leads to delayed healing [392, 393]. For cardiovascular complications, increased serum exosomal miR‐126 after GLP‐1 receptor agonists treatment suggests improved vascular endothelial function and reduced risk of cardiovascular events [389, 392, 393].

Spatial omics provides high‐resolution insights into locally resident biomarkers in diabetic complications. Spatial transcriptomics reveals the spatial colocalization of miR‐126 with vascular endothelial cells and inflammatory cells in diabetic foot ulcers, supporting its local function in angiogenesis. For diabetic nephropathy, this approach also defines the tubular‐specific spatial expression of miR‐192 and its link to regional renal interstitial injury [396].

#### Current challenges and future development trends

There are significant ethnic disparities in the application of biomarkers for endocrine and metabolic diseases, and their diagnostic thresholds, reference intervals, and clinical evaluation efficacy are affected by racial background. Taking T2D as an example, the World Health Organization defines HbA1c > 6.5% as the universal diagnostic criterion for diabetes [397], while East Asian populations have a significantly increased risk of diabetic microvascular complications at the subcritical HbA1c range of 6.0−6.5%. Therefore, the Asia‐Pacific diabetes guidelines have lowered the diagnostic threshold of HbA1c to 6.0% for Asian populations [398]. African Americans have significantly higher baseline UACR levels than Caucasians, and the diagnostic cutoff for diabetic nephropathy needs to be appropriately increased to avoid overdiagnosis [399]. The positivity rate of GADA), the core biomarker of latent autoimmune diabetes in adults (LADA), is 70−90% in Northern European Caucasian patients, but only 40−50% in Latino and Southeast Asian patients, showing obvious ethnic differences in diagnostic efficacy [400]. The above research evidence covers multi‐ethnic cohorts including Caucasians, East Asians, Africans, and Latinos, suggesting that racial characteristics should be fully considered in the clinical application of biomarkers for endocrine and metabolic diseases.

Currently, DM biomarkers still face multiple challenges. Some markers lack specificity; for example, GADA exhibits a certain false positive rate in the high‐risk T2D population [390]. The standardization of detection techniques is poor; miRNA in exosomes varies widely due to different separation and detection methods across laboratories [307]. Many potential markers, such as urinary exosomal miR‐192 and *CKD‐273*, have not yet been included in clinical guidelines despite sufficient evidence [291].

Future directions will focus on the construction of multi‐marker integration models that combine metabolomics, proteomics and exosomal markers to improve typing and prediction accuracy [393]. The sources of novel biomarkers will be expanded, such as circRNA, lncRNA and other noncoding RNA. For example, urinary exosome *circ_0000907* expression is positively correlated with healing rate in diabetic foot ulcers, which has the potential to become a new indicator for therapeutic effect monitoring [393, 401]. Promoting standardization of detection technology and its clinical translation is essential. With technological advances, biomarkers are expected to play a more central role in DM precision prevention, control and individualized treatment.

### Thyroid dysfunction

Thyroid dysfunction is an endocrine disorder resulting from abnormal thyroid hormone secretion or action. It primarily encompasses hyperthyroidism, hypothyroidism, and autoimmune thyroid diseases such as Hashimoto's thyroiditis and Graves' disease.The condition is influenced by genetic susceptibility, environmental disruptors, and immune imbalance [402, 403]. Globally, its incidence is 3−21%, with women and the elderly more susceptible; notably, hypothyroidism is far more common than hyperthyroidism. As key regulators of energy metabolism and nervous system development, thyroid hormone disorders can cause fatigue, abnormal weight, and metabolic disorders, increasing the risk of cardiovascular diseases and neurocognitive impairment [404]. Some autoimmune thyroid diseases are also linked with comorbidities like migraines [405]. Hashimoto's thyroiditis is characterized by thyroid lymphocyte infiltration and elevated TPO‐Ab, while Graves' disease is caused by abnormal TRAb leading to hyperthyroidism [406]. Accurate diagnosis of both conditions relies on specific biomarkers (Table S33) [407, 408].

#### The development process of biomarkers for thyroid dysfunction

Early on, hormone indicators such as thyroid‐stimulating hormone (TSH), free triiodothyronine (FT3), and free thyroxine (FT4) established the foundation for functional assessment [404]. TSH, which sensitively reflects the state of the thyroid gland, is a core indicator for screening hyperthyroidism and hypothyroidism. With advancements in immunological techniques, thyroid autoantibodies have become crucial for differentiating autoimmune thyroid diseases. TPO‐Ab and thyroglobulin antibody (Tg‐Ab) are employed to diagnose Hashimoto's thyroiditis, while TRAb serves as a specific marker for Graves' disease [407, 408], effectively distinguishing between autoimmune and non‐autoimmune thyroid disorders has led biomarker research into a more precise stage. Transcriptomic studies have found that miRNAs such as miR‐146a, miR‐142 and miR‐301 are differentially expressed in thyroid tissue and peripheral blood of patients with Hashimoto's thyroiditis and involved in the disease process by regulating immune and inflammatory pathways [406]. Proteomics revealed that abnormal expression of selenoprotein P and glutathione peroxidase 1 were associated with thyroid dysfunction and cancer risk [409]. In recent years, environmental exposure‐related markers have become a research hotspot [402, 403]. The *in vivo* concentration of environmental endocrine disruptors such as heavy metals and organic pollutants can be used as potential early warning indicators for thyroid dysfunction. At the same time, genomic studies confirmed that gene variations in *THADA* and *ITPK1* were closely related to thyroid function indexes and disease susceptibility, providing a molecular basis for the early identification of genetically related thyroid disorders [404].

#### The main types and typical cases of biomarkers for thyroid dysfunction

Among the diagnostic biomarkers, TSH is the most classic indicator, which decreases significantly in hyperthyroidism and increases significantly in hypothyroidism. Combined with FT3 and FT4, the type of functioresponse in real time and guide clinicalnal abnormality can be identified [404]. In differential diagnosis of autoimmune thyroid diseases, TPO‐Ab is a sensitive marker for Hashimoto's thyroiditis, with positive rate of 70−90%. High titer indicates increased risk of thyroid tissue damage [407]. The diagnostic specificity of TRAb for Graves' disease exceeds 95%, and its level is positively correlated with the severity of hyperthyroidism [406]. Environmental exposure‐related markers provide new dimensions for etiological diagnosis. Elevated serum cadmium concentration is associated with thyroid hormone synthesis disorders. Cadmium/selenium ratio can help to distinguish benign from malignant thyroid lesions [403]. Urinary phthalate metabolite concentrations are positively correlated with the risk of thyroid dysfunction and can be used as screening indicators for environmentally exposed thyroid diseases [402]. For molecular markers, miR‐146a was upregulated by 2−3 times in thyroid tissues of patients with Hashimoto's thyroiditis and was positively correlated with TPO‐Ab levels. Combined detection could improve diagnostic accuracy [406].

In Hashimoto's thyroiditis, patients with high expression of miR‐301a‐5p have an increased risk of developing clinical hypothyroidism within 10 years, which is related to the regulation of Th17 cell differentiation and enhanced inflammatory response [406]. Patients with serum selenium levels lower than 45 μg/L had a significantly increased risk of thyroid dysfunction deterioration, which was positively correlated with the risk of thyroid cancer [409]. In Graves' disease, persistently elevated titers of TRAb indicated an increased risk of relapse; patients whose TSH levels did not return to normal after treatment had a recurrence rate exceeding 40% at 1 year [406]. Genetic markers provide a basis for prognosticating genetically associated thyroid disorders. Carriers of the rs12712881 variant in the *THADA* gene were at increased risk of abnormal TSH fluctuations and more likely to progress to persistent thyroid dysfunction [404]. The *ITPK1* rs6575306 variant was associated with the risk of cardiovascular complications in hypothyroid patients. Among environmental exposure markers, patients with thyroid dysfunction and blood lead concentrations higher than 10 μg/dL had a 2.3‐fold increased risk of neurocognitive impairment, which may serve as a potential predictor of long‐term complications [403].

In terms of biomarkers for therapeutic effect monitoring, TSH remains the gold standard for monitoring treatment. After levothyroxine replacement therapy, hypothyroid patients need to maintain TSH at 0.5−4.5 μUI/mL. A continuous increase indicates insufficient dosage and a decrease suggests excessive dosage [404]. In Graves' disease patients after anti‐thyroid drug treatment, normalization of FT3 and FT4 and more than 50% reduction of TRAb titer indicate effective treatment and reduced risk of relapse [408]. Autoantibodies and molecular markers show unique values [407, 408]. More than 30% reduction of TPO‐Ab and Tg‐Ab levels in Hashimoto's thyroiditis patients after treatment indicated controlled thyroid inflammation and improved functional stability [408]. The expression of miR‐142‐3p was downregulated in effectively treated patients. Two‐year progression‐free survival rate reached 85% in patients whose level returned to healthy status after 3 months of treatment [406]. For environmental exposure related thyroid disorders, decreased phthalate metabolites and heavy metals levels were synchronized with improvement of thyroid function and could be used to monitor the effect of environment intervention [402, 403].

#### Current challenges and future development trends

At present, biomarkers for thyroid dysfunction are confronted with challenges including inadequate specificity, suboptimal standardization of detection methods, and an absence of early warning indicators [406, 407]. For example, the false positive rate of TPO‐Ab within the general population approximates 5%, which may result in overdiagnosis in regions characterized by excessive iodine consumption. Furthermore, miRNA detection employs a variety of platforms such as qRT‐PCR and NGS, necessitating enhanced standardization. Additionally, most markers exhibit significant alterations only when there is evident functional abnormality, thereby complicating ultra‐early intervention efforts.

Future research will focus on the integrated application of multiple biomarkers. The combination of hormone indicators, autoantibodies, environmental exposure markers and genetic variants to construct a prediction model will improve the accuracy of early diagnosis of Hashimoto's thyroiditis [404, 407, 408]. In terms of new biomarkers, non‐coding RNA such as circRNA and lncRNA are research hotspots. For example, the expression of *circ_0000907* in thyroid tissue is related to Graves' disease activity and is expected to become a new evaluation index [401]. In terms of detection technology innovation, microfluidic chip technology can simultaneously detect TSH, TPO‐Ab and miR‐146a, using only 5 μL of sample and shortening the detection time to 30 min, which can realize rapid bedside diagnosis. With the advancement of technology, biomarkers will play a more central role in precision screening, individualized treatment and complication prevention for thyroid dysfunction.

### Metabolic syndrome

MetS is a cluster of metabolic disorders characterized by central obesity, insulin resistance, dyslipidemia, and hypertension, often accompanied by non‐alcoholic fatty liver disease and chronic inflammation [410]. Its pathogenesis involves genetic predisposition, environmental factors, and an imbalance in the gut microbiota [411]. The global prevalence among adults has reached 30%, with a notable trend towards younger age groups [412]. As a multifactorial metabolic disorder, MetS disrupts metabolic homeostasis and significantly elevates the risk of T2D, cardiovascular diseases, and various cancers, posing a major global public health challenge (Table S34).

#### The development process of mets biomarkers

Research on MetS biomarkers has evolved from traditional clinical indicators to multi‐omics integration. Early diagnosis relied on waist circumference (WC), blood pressure (BP), fasting blood glucose, triglycerides, and high‐density lipoprotein cholesterol (HDL‐C). WC and BP are convenient detection methods that serve as core tools for population screening [412]. With a deeper understanding of inflammatory mechanisms, markers such as CRP and IL‐6 have been confirmed to be closely related to insulin resistance; hs‐CRP ≥ 3 mg/L serves as an important basis for MetS risk stratification [412]. Mendelian randomization studies based on CRP gene haplotypes further clarify that the observed association between serum CRP and insulin resistance/MetS is non‐causal, and the inflammatory response reflected by CRP may be the downstream manifestation of metabolic disorders, while the upstream inflammatory effectors may be the key to the causal link between inflammation and MetS [413]. More recently, omics technologies have advanced biomarker research into the precision stage. Metabolomics studies have found that branched‐chain amino acids (BCAAs) and aromatic amino acids are significantly elevated in the serum of MetS patients, demonstrating strong differential diagnostic value. Lipidomics has revealed that ceramides and sphingomyelins are closely associated with dyslipidemia and cardiovascular risk. Bidirectional mendelian randomization studies have confirmed a bidirectional causal link between circulating BCAAs and MetS‐related dyslipidemia: elevated BCAAs can directly cause increased triglycerides and decreased HDL‐C, while obesity and T2D, as core phenotypes of MetS, can also lead to the accumulation of BCAAs in the circulation [414]. Significant progress has also been made in gut microbiota research; decreased abundance of beneficial bacteria such as *Akkermansia muciniphila* and *Faecalibacterium prausnitzii* is associated with the occurrence and development of MetS, while reduced levels of their metabolites, SCFAs, reflect intestinal metabolic disorders [411]. For gut microbiota‐derived metabolites such as trimethylamine N‐oxide (TMAO) mentioned in mechanistic studies, mendelian randomization studies have verified its causal association with the increased risk of MetS and its cardiovascular complications [415]. Concurrently, oxidative stress markers and epigenetic markers offer new directions for early disease warning [416].

#### The main types and typical cases of mets biomarkers

Among routine clinical indicators, WC directly reflects central obesity; combining it with BMI improves the accuracy of risk assessment [412]. The simultaneous presence of abnormalities in three or more metabolic parameters, namely fasting blood glucose, triglycerides, HDL‐C, and elevated blood pressure (BP), is diagnostic for MetS. Among metabolic markers, BCAAs contribute to pathogenesis by promoting insulin resistance and lipid synthesis; their elevated serum levels enhance diagnostic accuracy. Elevated homocysteine is positively correlated with both MetS and cardiovascular complication risk, serving as a valuable auxiliary diagnostic marker. Regarding gut microbiota markers, *Akkermansia muciniphila* abundance demonstrates high diagnostic specificity [411]; combined detection with SCFAs can effectively distinguish different MetS subtypes. For inflammatory markers, combined measurement of CRP and IL‐6 accurately reflects systemic low‐grade inflammation [416].

Among oxidative stress markers, MetS patients with increased serum malondialdehyde had a significantly higher risk of developing T2D within 5 years [412]. The level of Urinary 8‐hydroxy‐2′‐deoxyguanosine was positively associated with the risk of cardiovascular events and was an independent predictor for long‐term complications [416]. Among metabolism and flora markers, decreased levels of adiponectin in patients indicated an increased risk of cardiovascular disease, reflecting dysfunction of adipose tissue. Decreased abundance of *Faecalibacterium prausnitzii* was related to the progression of nonalcoholic fatty liver disease [411]. For genetic markers, carriers of the *THADA* gene rs12712881 variant showed accelerated insulin resistance and were more likely to develop persistent MetS [404].

In terms of biomarkers for therapeutic effect monitoring, HbA1c is the gold standard for monitoring blood glucose control efficacy [412]. Reduced WC and controlled BP indicate effective intervention. Among metabolic and inflammatory markers, a reduced leptin/adiponectin ratio indicates improved dyslipidemia in tandem with enhanced insulin sensitivity. A decreased IL‐6 level indicates inflammation resolution and predicts stability of metabolic indicators [416]. Among gut microbiota biomarkers, increased SCFA levels after dietary fiber intervention indicate effective regulation of gut microbiota [411]. The 2‐year progression‐free survival rate was 80% in patients whose *Akkermansia muciniphila* abundance returned to normal.

#### Current challenges and future development trends

Current applications of MetS biomarkers encounter several challenges, including insufficient specificity, a lack of standardization in detection methods, and the heterogeneity of the disease, all of which compromise their applicability [395, 411, 416]. For instance, CRP levels are also elevated in infections and autoimmune diseases; methodologies for microbiota detection and SCFA analysis exhibit significant variation between laboratories; moreover, marker expression profiles differ considerably across MetS subtypes, thereby affecting their universality.

Future research will focus on constructing multi‐marker joint models. The integration of clinical indicators, metabolomics, gut microbiota and inflammatory markers can significantly improve the accuracy of early diagnosis [411, 412, 416]. For new markers, lipofuscin is a cross marker of oxidative stress and metabolic disorders; its tissue accumulation is related to MetS progression [416]. In terms of personalized intervention orientation, accurate programs should be developed based on metabolism typing and gut microbiota typing, such as improving SCFA production by dietary fiber or regulating *Akkermansia abundance* by probiotics [411]. The development of rapid detection technologies such as microfluidic chips will enable the simultaneous detection of multiple markers, providing technical support for point‐of‐care diagnosis and real‐time monitoring, and promoting the precise prevention, control and management of MetS.

### Thyroid carcinoma

TC is the most common malignant tumor of the endocrine system, derived from abnormal proliferation of thyroid follicular epithelium or parafollicular cells. Its major pathological subtypes include papillary, follicular, medullary, and highly malignant undifferentiated carcinoma [417]. The global incidence of TC has increased more than threefold over the past three decades and is about three times higher in women than in men. Pathogenesis involves genetic susceptibility, environmental endocrine disruptors, and autoimmune thyroid diseases [404]. During progression, TC metastasizes via immune escape and vascular invasion, commonly leading to cervical lymph node and distant organ metastasis; some subtypes are closely linked to immune‐related adverse events (Table S35) [407, 418].

#### The development process of biomarkers for thyroid cancer

Research on TC biomarkers has transitioned from traditional serum markers to the integration of multi‐omics. Foundational markers such as thyroglobulin (Tg), calcitonin (Ct), and CEA established the basis for diagnosis and therapeutic oversight; notably, Tg serves as the primary indicator for monitoring postoperative recurrence in differentiated thyroid cancer [419]. Advancements in molecular biology have unveiled driver gene mutations including *BRAF* ^V600E^, *RET*, and *RAS*. Specifically, *BRAF* ^V600E^ is a hallmark genetic marker for papillary carcinoma, while the *RET* mutation aids in differentiating hereditary from sporadic medullary carcinoma [417].

Recently, the integration of immunology and multi‐omics has advanced TC marker research towards precision medicine. PD‐L1 mediates tumor immune evasion by suppressing T cell activity; its tissue expression level and serum soluble form are crucial for diagnostic and prognostic evaluation [407]. Significant progress in Non‐coding RNA research has revealed that circRNAs and miRNAs contribute to tumorigenesis through the regulation of pathways such as Wnt/β‐catenin and MAPK, thereby emerging as potential targets for liquid biopsy [401, 419, 420]. Furthermore, epigenetic markers, ctDNA, and exosome‐derived miRNAs continue to emerge, offering novel avenues for the early diagnosis and dynamic monitoring of TC [417].

#### The main types and typical cases of thyroid cancer biomarkers

Diagnostic biomarkers are key tools for TC classification and early screening. Among the traditional markers, elevated serum Ct combined with fine‐needle aspiration can diagnose medullary carcinoma [419]. Calcitonin (Ct), a specific serum biomarker for medullary thyroid carcinoma (MTC), was cleared by the FDA through the 510 (k) pathway in the 1970s for the diagnosis, preoperative assessment, and postoperative recurrence monitoring of MTC. As a core regulatory‐approved marker for MTC, it exhibits high specificity and sensitivity, serving as a cornerstone for the clinical identification and surveillance of this subtype of thyroid cancer. Tg is widely used to evaluate postoperative residual lesions in differentiated thyroid cancer, but interference from anti‐Tg antibodies must be considered. Among molecular markers, the *BRAF* ^V600E^ mutation is important for distinguishing papillary carcinoma from benign nodules; combining it with the *TERT* promoter mutation improves diagnostic specificity [407, 417]. The *RET* mutation rate is high in hereditary medullary carcinoma; the M918T subtype is the most aggressive [419]. *PAX8/PPARG* gene rearrangement is a specific marker for differential diagnosis between follicular carcinoma and follicular adenoma. For immune and noncoding RNA markers, soluble PD‐L1 is specifically increased in the serum of patients with papillary carcinoma, and miR‐375 is highly expressed in fine‐needle aspiration samples from medullary carcinoma [407]. Combination detection improves the accuracy of uncertain nodule diagnosis.

Prognostic assessment biomarkers evaluate tumor aggressiveness, recurrence risk and long‐term patient outcomes. The Ki67 proliferative index is a core indicator; its increase indicates high risk and increased recurrence risk, which becomes an important reference for WHO classification [417]. *TERT* promoter mutation was associated with shorter survival in patients with sporadic medullary carcinoma [419]. Carriers of *RET* M918T mutation had lower 10‐year survival rates. *CDKN2A* gene methylation positively correlated with aggressiveness in nonfunctioning thyroid tumors. Among immune and molecular markers, high PD‐L1 expression indicated poor prognosis in undifferentiated and poorly differentiated cancers; its positive rate increased with the degree of dedifferentiation of the tumor [407]. High miR‐375 expression in medullary carcinoma was associated with distant metastasis and mortality risk; circulating miR‐375 levels could predict overall survival [419]. *RAS* mutation carriers usually have less aggressive tumors than *RET* mutation carriers and relatively better prognoses [419].

Efficacy monitoring markers reflect treatment response in real time and guide clinical strategy adjustment. Among the traditional markers, a decrease in Tg level to <1 ng/mL after surgery in patients with differentiated thyroid cancer indicates effective treatment; continuous increase suggests the possibility of recurrence [419]. Ct and CEA doubling time <6 months after treatment in patients with medullary carcinoma indicates disease progression [419]. For molecular and immune markers, changes in PD‐L1 expression can reflect the efficacy of *BRAF* inhibitors in patients with *BRAF* ^V600E^ mutation [407]. In combination with ICIs, progression‐free survival was significantly prolonged in patients with high PD‐L1 expression [407]. circEIF6 may enhance cisplatin resistance by regulating the miR‐144‐3p/TGF‐α axis; its expression level may have the potential to evaluate chemotherapy response [401, 420]. Silencing this gene can reverse drug resistance. Exosomal miR‐1261 is downregulated after treatment in papillary carcinoma and is associated with reduced tumor recurrence risk. In addition, the occurrence of immune‐related thyroid adverse events is positively correlated with the efficacy of ICIs, which can serve as an indirect monitoring indicator [418].

#### Current challenges and future development trends

The clinical application of TC biomarkers currently faces several challenges. For instance, some markers lack specificity; for example, PD‐L1 can be elevated in autoimmune thyroid diseases, leading to a propensity for false positives [407]. Furthermore, standardized detection systems are flawed; results for PD‐L1 expression vary when different antibodies are used, and platforms for non‐coding RNA detection are not standardized [417]. In addition, there is a scarcity of highly sensitive markers suitable for ultra‐early screening, as most existing indicators only exhibit significant abnormalities during the intermediate or advanced stages of tumor development.

Future directions are focused on the construction of multi‐marker integrated prediction models. The combination of gene mutations, immune markers, non‐coding RNAs, and epigenetic indicators improves the overall diagnostic and prognostic evaluation efficiency [407, 417, 419]. Actively developing new markers, such as using ctDNA for dynamic tumor mutation burden monitoring or evaluating invasiveness through exosomal proteins, further expands the dimensions of liquid biopsy applications [419]. Enhancing the guidance of biomarkers in precision treatment; for example, selecting a combination therapy of *BRAF* inhibitor and ICI based on *BRAF* ^V600E^ mutation status, adjusting chemotherapy based on circEIF6 expression levels, or screening suitable populations for immunotherapy based on PD‐L1 expression [407]. With continuous technological advancements, biomarkers are expected to play an increasingly central role in the precise classification, individualized treatment, and recurrence monitoring of TC.

### Pituitary adenoma

Pituitary Adenoma (PA), also known as pituitary neuroendocrine tumors (PitNETs), is a common intracranial tumor originating from the anterior lobe cells of the pituitary gland, accounting for about 17.2% of all intracranial tumors [421]. According to hormone secretion status, it can be divided into functional subtypes (such as prolactinoma, growth hormone‐secreting tumor, and adenocorticotropic hormone‐secreting tumor) and nonfunctional subtypes [422, 423]. Its occurrence is closely related to gene mutations such as *AIP* and *USP8*, epigenetic abnormalities such as *MEG3* methylation, and transcription factor expression disorders such as PIT1 and TPIT [423]. Although most pituitary tumors are benign in course, invasive tumors can invade the cavernous sinus and visual pathway structures. Functional tumors can cause metabolic disorders and dysfunction of multiple organ systems due to excessive hormone secretion. In very few cases, distant metastasis may even occur (Table S36) [422, 423].

#### The development process of PA biomarkers

Initial studies focused on hormonal markers such as prolactin (PRL), growth hormone (GH), and adrenocorticotropic hormone (ACTH), laying the groundwork for diagnosing functional PAs. For example, elevated serum PRL levels serve as the gold standard in diagnosing prolactinomas, boasting a sensitivity exceeding 95% [424]. The advent of immunohistochemical techniques introduced transcription factors like PIT1, TPIT, and SF1 as pivotal instruments for subtype classification, markedly enhancing diagnostic precision [425].

Recent advances in multi‐omics technologies, such as transcriptomics, have further propelled marker research in precision medicine. Various non‐coding RNAs have been confirmed to play roles in tumor progression. For example, miR‐let‐7 and miR‐423‐5p influence proliferation by regulating HMGA2 and PTTG1 [423, 424]. while LncRNA *H19* inhibits tumor growth through the mTORC1 pathway [421]. The circRNA *hsa_circ_0001368* is highly expressed in GH tumors and associated with invasiveness [424]. In addition, markers related to exosomes are also a research hotspot. For instance, serum exosome miR‐1180‐3p can serve as an early diagnostic indicator for nonfunctioning PAs, and exosome proteins such as MMP1 and N‐cadherin aid in assessing tumor invasiveness [421].

#### The main types and typical cases of PA biomarkers

Hormonal markers are central to the diagnosis of functional PAs. Prolactinomas are diagnosed based on serum PRL levels combined with imaging; GH‐secreting tumors require random GH and IGF‐1 elevation confirmed by a glucose suppression test; ACTH‐secreting tumors rely on ACTH, cortisol rhythms, and the dexamethasone suppression test [423, 424]. Transcription factors such as PIT1, TPIT, and SF1 are important for subtype identification [425]. *USP8* gene mutations are frequently detected in ACTH adenomas and are subtype‐specific [425]. Serum exosomal miR‐1180‐3p has diagnostic value for nonfunctioning PAs [421].

Among the prognostic assessment biomarkers, the Ki‐67 proliferation index is essential for assessing tumor proliferative activity, with higher values indicating high‐risk features and increased recurrence risk [422]. Among genetic markers, *AIP* gene mutations are linked to increased aggressiveness and drug resistance in GH tumors [425]. *CDKN2A* methylation shows a relatively high prevalence in nonfunctioning PAs and is positively associated with aggressiveness and recurrence risk. Epigenetic markers such as *MEG3* are silenced in nonfunctioning PAs due to promoter methylation [421]. Elevated levels of exosomal MMP1 and RASSF10 are also linked to increased aggressiveness, while *USP8* mutations in ACTH adenomas indicate a higher postoperative remission rate [425].

In terms of biomarkers for therapeutic effect monitoring, hormone levels are the basis for evaluating treatment response. For example, normalization of PRL and reduction in tumor volume after prolactinoma treatment indicate efficacy [423, 424]. Normalization of GH and IGF‐1 after surgery for GH tumors indicates biochemical remission. Molecular markers provide additional information. For instance, increased lncRNA *H19* in exosomes from patients with prolactinomas indicated sensitivity to cabergoline treatment [421]. Normalization of miR‐423‐5p expression was associated with a reduced risk of recurrence in patients with GH tumors [424]. The rate of recurrence decreased significantly in patients who were negative for *USP8* mutations following surgery for ACTH tumors. In addition, urinary NGAL can be used to monitor therapeutic responses in PA‐related kidney injury [426].

#### Current challenges and future development trends

Currently, the study of PA biomarkers is confronted with several challengesic diagnostic markers for non‐functional subtypes, which complicates early identification. Additionally, there is insufficient standardization of detection methods, impacting consistency (for instance, in the case of non‐coding RNA). Furthermore, marker specificity is limited (as evidenced by the fact that the critical value for Ki‐67 is not unified and some miRNAs are also abnormally expressed in other tumors) [424].

Future trends are centered on the construction of multi‐marker integrated models to enhance diagnostic and prognostic assessments by combining hormones, transcription factors, non‐coding RNAs, and imaging features [421, 423, 424]. Further research into new biomarkers is encouraged, particularly exploring the application of exosomal circRNA and ctDNA in monitoring recurrence [421]. The promotion of technological innovation and clinical translation is also vital, with a focus on utilizing artificial intelligence to integrate radiomics and molecular marker data to improve the accuracy of invasiveness prediction [427]. As technology continues to advance, it is anticipated that biomarkers will play an increasingly central role in the precise diagnosis and treatment system for PAs.

### Adrenocortical carcinoma

ACC is a rare malignant tumor that originates from the adrenal cortical cells, with an annual incidence of approximately 0.5−2.0 per million individuals, predominantly affecting those aged between 40 and 60 years [428, 429]. This disease can be categorized into functional and non‐functional types. The former type results from abnormal secretions of cortisol and androgen, leading to conditions such as Cushing's syndrome or abnormalities in sexual characteristics; the latter primarily arises due to the mass effect of the tumor. There is a strong association between the occurrence of ACC and mutations in the *TP53*, *CTNNB1*, and *TERT* genes [430, 431]. The pathological mechanism encompasses uncontrolled cell proliferation, aggressive invasion, and hormonal secretion disorders. Regrettably, the 5‐year survival rate for this disease stands at a mere 15–40%, with a high propensity for distant metastasis (in areas such as the liver, lungs, and bones). Patients with the functional variant often suffer severe metabolic and cardiovascular complications (Table S37) [428, 429].

#### The development process of ACC biomarkers

Early diagnostic approaches relied on hormonal markers, such as markedly elevated serum dehydroepiandrosterone sulfate (DHEAS), which demonstrated high sensitivity in differentiating benign from malignant lesions [431, 432]. With the widespread adoption of immunohistochemical techniques, biomarkers including Ki‐67 and p53 emerged as central indicators for risk stratification; notably, the Ki‐67 index has been incorporated into the WHO classification system [430, 433]. Advances in molecular genetics have identified *TP53* and *CTNNB1* mutations as key driving events [336]. Furthermore, non‐coding RNAs such as miR‐125b are downregulated in ACC tissues, exhibiting significant diagnostic potential, while their exosomal form offers promise for non‐invasive detection [338]. Additionally, *TERT* promoter mutations or amplifications contribute to tumor immortalization through a telomere maintenance mechanism, establishing them as independent prognostic factors [336]. Emerging technologies, including exosome protein analysis and ctDNA assessment, are increasingly being applied in clinical settings, offering novel modalities for dynamic disease monitoring [434].

#### The main types and typical cases of ACC biomarkers

Diagnostic biomarkers are primarily used for ACC classification, benign‐malignant differentiation and early screening. Among the hormonal markers, elevated serum cortisol and a negative dexamethasone suppression test suggest functional ACC [431, 432]. An increased DHEAS with gender specificity has high specificity in diagnosing ACC. Among protein markers, immunohistochemical positivity of steroidogenic factor 1 (SF1) helps to confirm an adrenal cortical origin [430, 433]. Nuclear β‐catenin localization is consistent with *CTNNB1* mutation and further supports the diagnosis of ACC. For molecular markers, *TP53* gene mutations are important in genetically related ACCs [336]. The combination of serum miR‐125b and DHEAS improves diagnostic sensitivity [338].

Prognostic assessment biomarkers evaluate tumor invasiveness and recurrence risk. The Ki‐67 index is central; its elevation signifies a high‐risk profile and increased likelihood of recurrence [430, 433]. Among genetic indicators, *TERT* promoter mutations are significantly linked to reduced patient survival, whereas *CDKN2A* methylation positively correlates with tumor aggressiveness [336]. Diminished expression of non‐coding RNAs such as miR‐125b is tied to shortened overall survival, implicating mechanisms like tumor suppressor gene inactivation and metastasis facilitation [338]. Patients with *TP53* mutations exhibit elevated postoperative recurrence rates and often display resistance to chemotherapy [336]. Additionally, lowered serum selenium levels correlate with an increased risk of disease progression, offering supplementary prognostic insights [434].

In terms of biomarkers for therapeutic effect monitoring, biomarkers reflecting treatment response in real‐time and assisting recurrence monitoring are effective for efficacy monitoring. Hormonal indicators remain central, such as postoperative cortisol level decrease indicating successful surgery; dynamic DHEAS decrease reflecting reduced tumor burden [431, 432]. Protein and molecular markers provide further evidence: negative nuclear β‐catenin expression indicates complete resection [336]; decreased cfDNA mutation abundance predicts good treatment response in patients with *TP53* mutation [434]; increased exosomal miR‐125b level is positively correlated with progression‐free survival [431, 432]. In addition, the reduction of Ki‐67 index to a low level after treatment suggests controlled proliferation and improved prognosis [434].

#### Current challenges and future development trends

Currently, the research and application of ACC biomarkers face numerous challenges. Some hormonal markers may exhibit abnormalities in benign lesions, thereby limiting their specificity [431, 432]. The detection methods for protein markers, such as Ki‐67, lack complete standardization [430, 433]. Furthermore, early diagnostic systems are suboptimal, particularly for non‐functional ACC, due to the absence of effective screening tools.

Future directions include the construction of multi‐marker integrated models combining hormones, proteins, genes and non‐coding RNA to improve diagnostic and prognostic accuracy [428, 430]. Expansion of exosome proteins and new markers such as ctDNA will facilitate clinical translation of liquid biopsy [434]. Marker‐based precision treatment strategies can be established, for example, selection of Wnt pathway inhibitors based on *CTNNB1* mutation, adjustment of chemotherapy according to *TP53* status, formulation of individualized surveillance plan according to Ki‐67 index [428, 430]. With continuous technological advances, biomarkers are expected to play a central role in early identification, individualized treatment and whole‐course management of ACC.

### Pancreatic neuroendocrine neoplasms

pNENs are heterogeneous tumors originating from pancreatic neuroendocrine cells, constituting approximately 3−5% of all pancreatic neoplasms [434]. They are classified by hormone secretion into functional and non‐functional types, and histopathologically into well‐differentiated and poorly differentiated subtypes [435]. Pathogenesis is closely associated with genetic predisposition syndromes and molecular alterations [411, 434]. Prognosis varies significantly: well‐differentiated tumors have a 5‐year survival rate of 85.4%, while poorly differentiated ones have a median survival of ~7.5 months [434, 436]. Common metastatic sites are the liver and lymph nodes; functional subtypes may cause metabolic complications due to excessive hormone secretion (Table S38) [411, 435].

#### The development process of pNENs biomarkers

As biomarker research advances, the diagnosis and treatment of pNEN are becoming increasingly precise. Initial studies focused on traditional markers such as chromogranin A (CgA), neuron‐specific enolase (NSE), and specific hormones, establishing a foundation for diagnosis and monitoring [411, 435]. Among these, CgA emerged as a universal serum marker; the detection of insulin in conjunction with Whipple's triad constitutes the diagnostic criterion for insulinoma. With advancements in molecular biology, gene mutations including *MEN1*, *DAXX*/*ATRX*, and *TP53*/*RB1* have been identified, facilitating the differentiation between hereditary and sporadic cases, as well as between well and poorly differentiated tumors [411, 434, 435].

Recently, transcriptomics and epigenetics have further expanded the scope of markers. Biomarkers such as miRNAs (miR‐196a, miR‐21) and *TERT* promoter methylation are increasingly being applied in liquid biopsy for dynamic monitoring [411, 435]. In imaging, the combination of somatostatin receptor 2A (SSTR2A) with 68Ga‐DOTATATE PET/CT has significantly improved diagnostic accuracy and treatment guidance [434, 436]. Emerging detection tools, including NETest, exosomal miRNAs, and tumor microenvironment markers, are progressively entering clinical practice, offering new possibilities for early diagnosis and prognostic stratification [411, 435].

#### The main types and typical cases of pNENs biomarkers

Diagnostic biomarkers form the foundation for pNEN classification, differentiation between benign and malignant tumors, and early screening. Among traditional markers, serum CgA demonstrates a diagnostic sensitivity of 60−83%; when combined with NSE, it enhances specificity, though interfering factors such as renal insufficiency must be excluded [411]. For functional subtypes, a serum insulin level of ≥43 pmol/L, in conjunction with Whipple's triad, confirms an insulinoma diagnosis, while gastrin levels ≥300 pg/mL indicate gastrinoma [411]. Regarding molecular markers, *MEN1* mutations are specific to genetically linked cases [436]; *DAXX*/*ATRX* mutations, associated with telomere alternative lengthening, facilitate differentiation between WD‐PNET and PanNEC [435]; and *TP53/RB1* double mutations serve as critical indicators for distinguishing PanNEC from WD‐PNET G3 [436]. In terms of immune and imaging markers, SSTR2A immunohistochemical positivity reaches 80% in WD‐PNET, guiding selection for peptide receptor radionuclide therapy, while high PD‐L1 expression in PanNEC provides a molecular basis for immunotherapy [435].

Prognostic assessment biomarkers predict tumor aggressiveness, risk of recurrence and long‐term outcome. The Ki67 proliferative index is central to the WHO classification; a Ki67 ≥ 20% (G3) indicates high risk with a sevenfold higher risk of recurrence than G1/G2 [434]. Among genetic and molecular markers, patients with *DAXX*/*ATRX* mutations have a 40% rate of recurrence at 5 years [435]; overall survival is significantly reduced in PanNECs with *TP53/RB1* alterations [436]; miR‐196a overexpression is independently associated with early recurrence after surgery for WD‐PNET [411].

For immune and imaging markers, high PD‐L1 expression in PanNEC is related to immune escape and poor prognosis [435]; a total tumor uptake of >37.8 on 68Ga‐DOTATATE PET/CT is associated with an increased risk of progression in WD‐PNET; increased infiltration by tumor‐associated macrophages (CD68 + ) indicates an elevated risk of recurrence in nonfunctional pNENs.

Biomarkers for therapeutic effect monitoring enable real‐time evaluation of therapeutic efficacy and guide adjustments to clinical management. Among traditional biomarkers, a postoperative decrease in CgA levels exceeding 50% in patients with WD‐PNET indicates surgical effectiveness; conversely, a continuous increase suggests the potential for recurrence [411]. In functional subtypes, normalization of insulin and gastrin levels corresponds with the alleviation of clinical symptoms.

For molecular and novel markers, a NETest (51‐gene transcript detection) score of ≤40% indicates effective treatment; a dynamic increase of ≥40% can signal disease progression with 98% consistency with imaging assessments [411]. In WD‐PNET patients undergoing peptide receptor radionuclide therapy, sustained high SSTR2A expression suggests a favorable therapeutic outcome. A decrease in *DAXX*/*ATRX* mutation abundance by >50% in ctDNA is positively correlated with chemotherapy response [435]. An elevation of ≥30% in exosomal miR‐125b levels following effective treatment can predict 2‐year progression‐free survival [411].

#### Current challenges and future development trends

Currently, the application of biomarkers in pNENs faces numerous challenges. Tumor heterogeneity results in inadequate marker specificity; for instance, CgA can be elevated in benign gastrointestinal disorders and certain WD‐PNETs lack definitive molecular markers [411, 434]. There is a lack of standardization in detection methods, with significant inter‐observer variability in Ki‐67 counts and non‐unified approaches for non‐coding RNA detection. Furthermore, there is a dearth of reliable early diagnostic markers, particularly for non‐functional tumors <2 cm, which limits detection capabilities. The sensitivity of liquid biopsy in early‐stage patients remains suboptimal, with a detection rate of only 30–40% [435].

Future research directions will focus on the construction of multi‐marker combined models to improve diagnostic and stratified accuracy by integrating clinical, molecular, imaging, and liquid biopsy data; novel markers such as lncRNAs, circRNAs, and tumor microenvironment‐related indicators are research hotspots [411]; technically, the popularization of liquid biopsy and AI‐assisted radiomics are expected to achieve non‐invasive, dynamic, and efficient disease monitoring [437]; individualized treatment will rely more heavily on molecular marker‐guided selection of targeted therapy, chemotherapy, and radionuclide therapy [434]. Predictably, with continuous technological advancements, biomarkers will play an increasingly central role in precision medicine for pNENs (Figure S4).

### Hierarchical network integration of biomarkers in the endocrine and metabolic systems

The endocrine and metabolic systems maintain bodily homeostasis through a sophisticated feedback regulatory network. The biomarkers associated with these systems exhibit unique dynamic characteristics and hierarchical structures. This study presents a multilevel integrated framework, spanning from core hormone regulation to tissue effect response, derived from a systematic analysis of major endocrine and metabolic diseases, including diabetes, thyroid diseases, obesity, and metabolic syndrome (Table S39).

#### Core regulatory markers of the central‐target gland axis system

The hypothalamic–pituitary–target gland axes constitute the core regulatory hierarchy of the endocrine system. The pulsatile secretory characteristics and circadian rhythms of thyroid‐stimulating hormone (TSH), a key messenger of the pituitary–thyroid axis, provide dynamic indicators for assessing thyroid function. Similarly, the negative feedback regulation between adrenocorticotropic hormone (ACTH) and cortisol can reveal the functional status of the hypothalamic–pituitary–adrenal (HPA) axis through dexamethasone suppression tests. Notably, these axial markers not only reflect target gland functions but also demonstrate the precision of central regulation. For example, the early increase in TSH in subclinical hypothyroidism is a significant early warning signal for progression to overt hypothyroidism. Damage to the central regulatory axis can be caused by various factors. Traumatic brain injury is a common cause of hypopituitarism and growth hormone deficiency [438], whereas thickening of the pituitary stalk may indicate multiple pathological processes, such as tumors, inflammation or infection [439, 440]. In addition, although isolated hypopituitarism is rare in adults, its diagnosis requires a detailed assessment of axis function [441].

#### Dynamic balance markers of the energy metabolism network

Markers of glucose and lipid metabolism form the core monitoring network for energy homeostasis. Glycated hemoglobin (HbA1c), a “memory” indicator reflecting average blood glucose levels over 2–3 months, complements transient blood glucose indicators such as fasting plasma glucose (FPG) and 2−h postprandial blood glucose (2hPG). Recent advancements in continuous glucose monitoring (CGM) technology offer dynamic and comprehensive insights into daily blood glucose management for patients with T2D, markedly enhancing control outcomes [442, 443]. In terms of lipid metabolism, both low‐density lipoprotein cholesterol (LDL‐C) and non‐high‐density lipoprotein cholesterol (non‐HDL‐C) are used to gauge atherosclerosis risk. The apolipoprotein A1/B100 ratio offers a more precise evaluation of lipoprotein particle function. The present evidence and clinical practices indicate that compared with LDL‐C, apolipoprotein B (ApoB) is a superior marker for assessing atherosclerosis risk because of its ability to accurately represent the total number of atherosclerotic lipoprotein particles [444]. Moreover, the “cumulative exposure to LDL” hypothesis highlights the pivotal role of lifelong LDL‐C burden in cardiovascular risk, emphasizing its significance for early preventative measures [445]. The use of these markers collectively provides a holistic view of metabolic imbalances. Their judicious use is crucial for the early diagnosis and management of prediabetes [391] and hyperglycemia in hospitalized patients [446].

#### Systemic regulatory factors of the fat endocrine network

Adipose tissue, which is the largest endocrine organ, secretes adipokines such as adiponectin, leptin, and resistin, which constitute a crucial metabolic regulatory network. Adiponectin augments insulin sensitivity via activation of the AMPK pathway, and decreased adiponectin levels are strongly associated with metabolic syndrome. Leptin influences the hypothalamic feeding center by traversing the blood–brain barrier, and resistance to leptin is a significant factor potentially associated with obesity pathogenesis. In addition to its central role in energy homeostasis, abnormal leptin levels are correlated with increased risks of obesity and cardiovascular diseases [447]. The integration of these adipokines with conventional metabolic markers offers a novel classification framework for obesity‐associated metabolic disorders [448].

#### Interactive markers of bone metabolism and mineral homeostasis

Bone metabolism markers represent a unique aspect of endocrine regulation. The equilibrium between parathyroid hormone (PTH) and 25‐hydroxyvitamin D plays a crucial role in the regulation of calcium and phosphorus metabolism. Concurrently, osteocalcin (OC), an osteoblast‐specific protein, has recently been shown to influence energy metabolism. By monitoring serum levels of calcium, phosphorus, and alkaline phosphatase (ALP), detecting metabolic bone diseases at their nascent stages is possible. Additionally, bone turnover markers such as PINP and β‐CTX provide a dynamic means to assess treatment responses in osteoporosis. These markers are integral to both the diagnosis and evaluation of treatment response in osteoporosis. Notably, effective anti‐osteoporosis interventions can markedly reduce fracture risk [449, 450]. Special attention should be given to specific groups, such as men, during diagnosis and treatment [451]. A recent review offered a comprehensive overview of the pathophysiology, diagnostic methods, and therapeutic advancements in osteoporosis [452].

### The endocrine and metabolic systems as systemic regulatory hubs

**Figure 4:**
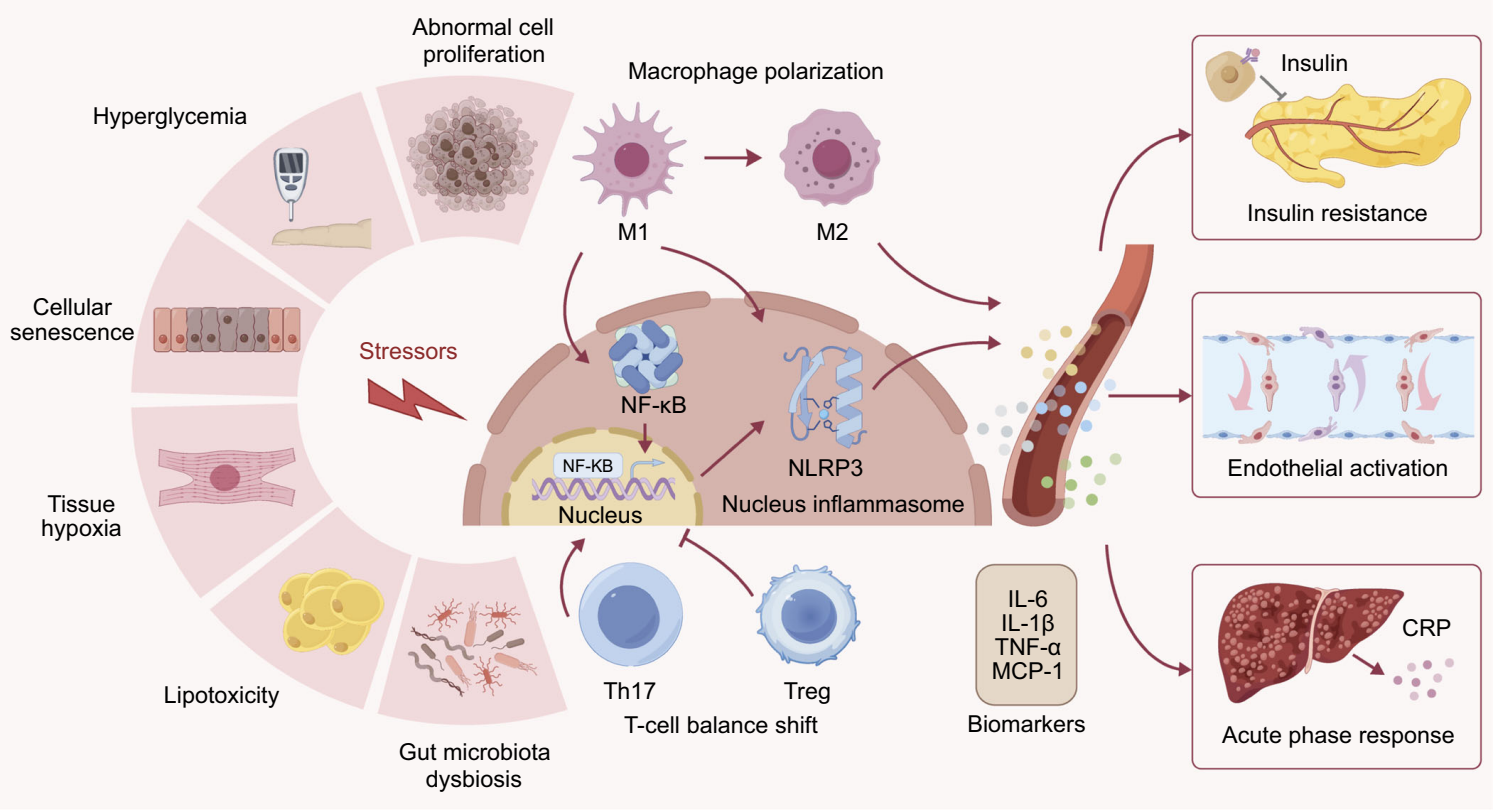
Schematic of chronic low‐grade inflammation and immunometabolic imbalance as a shared “soil” for chronic diseases. This figure illustrates how diverse chronic stressors converge on inflammatory signaling hubs to drive systemic, low‐grade inflammation and downstream organ dysfunction across multiple chronic diseases (e.g., neurodegeneration, metabolic disorders, cardiovascular diseases, and cancer). The mechanism diagram summarizes key triggers, including gut microbiota dysbiosis with increased translocation of LPS, lipotoxicity induced by saturated fatty acids, hyperglycemia, cellular senescence with release of SASP, and tissue hypoxia. In the center, these stressors promote immune cell infiltration and immunophenotypic shifts within the microenvironment: macrophages polarize toward a pro‐inflammatoand exhibits significant spatialry M1 state (from anti‐inflammatory m2), and the Th17/Treg balance is disrupted toward Th17 dominance. These immune alterations activate core inflammatory pathways, highlighted by NF‐κB signaling and the NLRP3 inflammasome, which act as key “engines” for the production and amplification of pro‐inflammatory mediators. The resulting cytokines and chemokines, including IL‐1β, IL‐6, TNF‐α, and MCP‐1, enter the circulation and exert systemic effects on multiple organs, leading to insulin resistance, endothelial activation, and hepatic acute‐phase responses (e.g., CRP production).

The endocrine and metabolic systems engage in intricate communication with various organs throughout the body via hormone signals, functioning as central regulators in the cross‐system integration of biomarkers (Table S40 and Figure 4).

#### Systemic regulatory network of the hypothalamic–pituitary–target gland axis

The hypothalamic–pituitary axes serve as neuroendocrine transducers and regulate systemic functions through multiple downstream axonal systems. The growth hormone–insulin‐like growth factor 1 (GH–IGF‐1) axis not only regulates linear growth but also affects protein synthesis and glycolipid metabolism. The thyrotropin‐releasing hormone (TRH)–TSH–thyroid hormone axis affects cardiovascular function and central nervous system development by regulating the basal metabolic rate. A combination of these axial markers enables integrated assessment of multisystem comorbidities through associations such as those between the IGF‐1 level and bone mineral density (BMD) and the FT3 level and lipid profile.

#### Energy metabolism–cardiovascular–renal axis integration

Insulin resistance, the core link of metabolic syndrome, affects cardiovascular and renal functions through multiple mechanisms. The hyperinsulinemic–euglycemic clamp technique serves as the gold standard for assessing insulin sensitivity, whereas the HOMA‐IR offers a clinically practical alternative indicator. Combining these markers of metabolic abnormalities with BNP and UACR can provide early warning of cardiovascular and renal complications in patients with diabetes. Notably, the therapeutic efficacy of SGLT2 inhibitors underscores the clinical significance of the metabolic–cardiorenal axis. From a preventive standpoint, primary prevention of subclinical atherosclerosis in young individuals necessitates the integration of metabolic markers such as blood lipids and blood glucose for risk assessment [453]. Among these, dietary intervention forms the cornerstone of dyslipidemia management [454].

#### Cross‐dialog between the stress axis and immune metabolism

The HPA axis is activated in response to stress, with cortisol acting as the key effector hormone that regulates immune and metabolic functions via glucocorticoid receptors. Excessive cortisol in patients with Cushing's syndrome results in central obesity and diabetes, whereas Addison's disease presents opposite metabolic changes. The diagnosis and management of Cushing's syndrome are rather complicated, especially with respect to the diagnostic challenges of periodic Cushing's syndrome [455, 456]. The latest overview of the disease summarized its pathology, diagnosis and treatment [457, 458]. Moreover, inflammatory factors can induce insulin resistance, forming a vicious cycle between metabolism and immunity. Combining serum cortisol rhythm detection and inflammatory markers provides new methods for evaluating metabolic inflammation. Cushing's syndrome itself may also affect the thyroid axis [459].

#### Emerging role of the gut–pancreas–brain axis in metabolic regulation

Together, intestinal hormones and neural signals form the gut–pancreas–brain regulatory axis. Glucagon‐like peptide‐1 (GLP‐1) is secreted by intestinal L cells after food intake, promoting insulin release and suppressing appetite. Ghrelin levels increase during fasting periods, stimulating eating behavior. GLP‐1 and ghrelin have antagonistic effects on food reward and eating behavior and are central to the regulation of the brain–gut axis [460]. The function of the brain–gut axis is closely related to the human reward system [461]. The combination of these enteroendocrine hormones with central nervous system imaging offers a novel biomarker panel for studying obesity and abnormal eating behaviors. Sleep deprivation disrupts central appetite regulation by impacting key peptide substances in the hypothalamic appetite‐regulating center, such as ghrelin and leptin [462].

#### Cross‐system early warning value of endocrine and metabolic markers

Endocrine and metabolic markers serve as early warning indicators for systemic diseases. For example, abnormal thyroid function, characterized by elevated TSH levels, is significantly linked to dyslipidemia and nonalcoholic fatty liver disease. Similarly, a low serum vitamin D level not only disrupts bone metabolism but is also correlated with an increased risk of autoimmune diseases and tumors. These interconnected associations underscore the sentinel role of the endocrine–metabolic system in comprehensive health monitoring.

## URINARY SYSTEM

Literature retrieval for the urinary system was systematically performed in PubMed and Web of Science using Boolean logic with field‐restricted search strings. The comprehensive search strategies were tailored for each disease as follows: for chronic kidney disease, (“chronic kidney disease” OR “renal insufficiency, chronic”) AND (“biomarker”) AND (“disease”); for acute kidney injury, (“acute kidney injury”) AND (“biomarker”) AND (“disease”); for glomerulonephritis, (“glomerulonephritis” OR “glomerulonephritis”) AND (“biomarker”) AND (“disease”); for renal cell carcinoma, (“renal cell carcinoma” OR “carcinoma, renal cell”) AND (“biomarker”) AND (“disease”); and for bladder cancer, (“bladder cancer” OR “urinary bladder neoplasms”) AND (“biomarker”) AND (“disease”). The retrieval period was defined as January 1, 2020 through December 31, 2025. Only articles published in English were considered. Both original research articles and systematic or narrative reviews were included. Retrieved records were screened by titles and abstracts; articles deemed irrelevant to urinary disease biomarkers, lacking full‐text availability, or containing incomplete data were excluded, and the remaining full texts were thoroughly evaluated. The selected disease spectrum encompasses CKD, acute kidney injury (AKI), glomerulonephritis, renal cell carcinoma (RCC), and bladder cancer, all representing major urinary system disorders with high prevalence, substantial disease burden, well‐established biomarker frameworks, and strong clinical translational relevance.

### Chronic kidney disease

CCKD is a major chronic condition characterized by progressive and irreversible loss of renal function. Patients with end‐stage renal disease (ESRD) have significantly reduced long‐term quality of life. As there are no overt early symptoms, most patients are diagnosed only after a significant decline in glomerular filtration rate (GFR) or the occurrence of structural kidney damage. Therefore, it is crucial to develop effective biomarkers for early diagnosis, accurate prognosis assessment, and therapeutic decision making (Table S41) [463].

#### Development process of biomarkers

Historically, clinical evaluation has relied on conventional markers such as serum creatinine, cystatin C, and urinary albumin. Serum creatinine is widely used due to its ease of measurement; however, it only increases significantly after approximately 50% of renal function has been lost, rendering it insensitive to early‐stage pathology [464]. Cystatin C, which is less affected by muscle mass, offers improved sensitivity for detecting early declines in renal function [464]. Urinary albumin remains a key indicator of glomerular injury, yet approximately 30% of patients with diabetic kidney disease (DKD) exhibit normal levels despite existing functional impairment, indicating limited utility as a universal early biomarker [465].

In terms of the omics technology‐driven stage, advances in omics technologies have facilitated the discovery of novel biomarkers. Proteomics has identified KIM‐1 and NGAL as markers of tubular injury, which can detect ischemic or nephrotoxic insults before changes in serum creatinine [466]. Metabolomics has uncovered metabolites such as 5‐methoxytryptophan (5‐MTP), whose levels correlate with advancing CKD stages and may reflect alterations in pathways regulating inflammation and antioxidant defense [467]. Gut microbiome profiling has demonstrated microbial dysbiosis in patients with CKD. Specifically, certain genera, such as Ruminococcus and Roseburia, hold promise for early discrimination [468]. MicroRNA studies have pinpointed circulating noncoding RNAs, including miR‐451 and miR‐181a, that closely correlate with estimated GFR (eGFR) and the extent of renal fibrosis [469].

Current studies highlight the importance of integrating multi‐omics data to improve diagnostic accuracy. For example, the CKD273 urinary peptide panel showed predictive value for microalbuminuria development in T2D patients [470]. Multimodal models combining KIM‐1, 5‐MTP, and gut microbiota metabolites with machine learning algorithms significantly enhance early detection capabilities. Additionally, liquid biopsy approaches, such as urinary exosome analysis and plasma cfDNA assessment, offer dynamic monitoring tools for tracking disease progression and therapeutic response.

#### Main types and core characteristics of biomarkers

Podocyte‐related biomarkers show great potential for the early diagnosis and assessment of glomerular diseases. Dendrin moves from the cytoplasm to the nucleus when podocytes are injured, a process associated with disease progression by promoting apoptosis, making it a possible marker for early glomerular damage [471]. Podocin and podocalyxin, which are crucial for maintaining the structure and function of podocytes, have urinary concentrations that positively correlate with disease severity, thus reflecting the degree of glomerular pathology [472, 473]. The abnormal expression of immunoglobulin G (IgG) may indicate a disruption of the glomerular mechanical barrier and serves as a useful adjunct in evaluating disease activity [474]. c‐Myb, which regulates epithelial‐mesenchymal transition, is associated with renal fibrosis and represents a new early injury biomarker, offering fresh insights into disease mechanisms and opportunities for early intervention [475]. Together, these biomarkers shed light on various aspects of the pathophysiology of glomerular injury and provide multifaceted references for clinical diagnosis, disease monitoring, and treatment evaluation.

Biomarkers of renal tubulointerstitial injury also play a key role in the early detection, monitoring, and prognosis evaluation of kidney diseases. KIM‐1 and NGAL are highly sensitive indicators of tubular damage that can be rapidly released into urine after injury. The rise is usually 2−4 weeks earlier than that of serum creatinine, providing an important time window for intervention [466]. The CosMx single‐cell spatial transcriptomics technique enables precise localization of KIM‐1 (HAVCR1) expression in renal biopsy tissues. Studies have revealed that in patients with DKD, KIM‐1 is specifically expressed in injured proximal tubular cells (LTL+/KIM1+) and colocalizes with the lipid droplet marker PLIN2 [476]. Liver‐type fatty acid‐binding protein (l‐FABP) reflects abnormal lipid metabolism and oxidative stress in renal tubules, and has good performance in complex clinical scenarios with little interference [477]. IL‐18, an inflammation‐related cytokine, and uromodulin, which plays an important role in regulating tubular function, are significantly correlated with eGFR and clinical outcomes, and can accurately reflect disease severity and trends [478].

Advanced imaging technologies have become essential non‐invasive assessment tools in the clinical diagnosis of CKD. Ultrasound radiomics enables quantitative evaluation of fibrosis severity by extracting texture and filtering features from renal parenchyma, with preliminary clinical studies demonstrating its capability to non‐invasively distinguish membranous nephropathy from IgA nephropathy [479]. Shear wave elastography (SWE) and acoustic radiation force impulse imaging allow non‐invasive assessment of renal stiffness, which correlates with the degree of renal interstitial fibrosis [480]. ASL magnetic resonance imaging can quantify renal blood flow without the use of contrast agents, making it suitable for CKD patients at risk of contrast‐induced nephropathy [481]. Furthermore, artificial intelligence‐based imaging analysis models combined with clinical biomarkers can significantly improve the predictive performance of CKD risk assessment models [482].

Prognostic and predictive biomarkers are essential for assessing disease progression, predicting treatment responses, and stratifying patient risk. Serum uromodulin levels reflect tubular function; decreased expression is associated with impaired tubular integrity and increased risk of progression to ESRD [483]. MicroRNA‐181a (miR‐181a) downregulation correlates with accelerated loss of renal function and progressive fibrosis, providing a molecular marker for monitoring disease advancement [484]. 5‐MTP and asymmetric dimethylarginine (ADMA) have demonstrated value in evaluating responses to antioxidant therapy and statin interventions, respectively, enabling therapeutic optimization [467]. Emerging biomarkers, including β‐trace protein (BTP), β2‐microglobulin (β2M), and fetuin‐A, require further validation to establish their clinical utility [485].

These biomarkers collectively enable a holistic approach to kidney disease management through risk prediction, therapeutic guidance, and longitudinal monitoring across multiple domains.

#### Current challenges and future development trends

Despite these advances, several challenges remain in the clinical translation of kidney disease biomarkers. For early detection, both conventional and many novel biomarkers have suboptimal sensitivity (often < 60%) for identifying stage I/II CKD, limiting their utility in screening and secondary prevention strategies. Technologically, while multi‐omics platforms hold transformative potential, high costs and lack of standardized analytical protocols limit widespread implementation in routine clinical practice. Furthermore, novel biomarkers are not yet integrated into standard diagnostic pathways. Clinician awareness regarding their clinical validity, interpretation frameworks, and appropriate contexts of use remains limited, restricting optimal utilization.

In addition, traditional markers such as serum creatinine and eGFR equations exhibit systematic discrepancies across racial groups. For example, Black individuals typically present higher serum creatinine concentrations at comparable renal function levels compared with White populations. Such differences necessitate race‐adjusted algorithms in certain clinical equations and highlight that uniform reference intervals may lead to misclassification in non‐White populations.

The Future directions of biomarker research are becoming increasingly evident. Technologically, the integration of emerging methodologies, including spatial transcriptomics, exosome surface proteomics, and microfluidic chip systems, is expected to drive biomarker detection towards greater precision, speed, and miniaturization, thus reducing costs and enhancing scalability. Clinically, the combination of liquid biopsy with organoid‐based drug sensitivity testing holds promise for real‐time monitoring of therapeutic responses, facilitating individualized treatment strategies and advancing precision medicine [486]. Crucially, multi‐dimensional integration emerges as a central trajectory. By synthesizing multi‐omics data, artificial intelligence algorithms, and comprehensive clinical information, integrated biomarker systems can be developed to support full‐cycle care, ranging from disease screening and early diagnosis to ongoing monitoring and prognostic management. Ultimately, this enables intelligent, precise, and personalized management of kidney diseases.

### Acute kidney injury

AKI is a common and serious complication in patients with trauma and critical illness, which is associated with high morbidity and increased mortality risk as well as long‐term renal dysfunction. Limitations of conventional diagnostic criteria are well documented, prompting a shift in biomarker research from single analyte detection to integrated multidimensional approaches (Table S42) [487].

#### Development process of biomarkers

For decades, AKI diagnosis has relied primarily on functional markers such as serum creatinine, which reflects glomerular filtration status. However, serum creatinine levels do not increase until more than 50% of renal function is impaired and are confounded by factors including age, sex, and muscle mass [488].

With a more in‐depth understanding of AKI pathophysiology, biomarkers specific to tubular injury, inflammation, and microvascular dysfunction have been identified. NGAL and KIM‐1 increase within hours of renal insult allowing detection of subclinical injury prior to functional deterioration [489].

Current research is aimed at developing integrated models combining structural biomarkers, functional indices, and clinical risk scores. By integrating biomarkers with different pathophysiological mechanisms and using tools such as the renal angina index to stratify patients, clinicians can achieve earlier diagnosis, better risk stratification, and targeted therapeutic guidance. In 2014, the FDA approved a test based on the combination of tissue inhibitor of metalloproteinase 2 in urine and insulin‐like growth factor binding protein 7 ([TIMP‐2] × [IGFBP7]) to determine whether certain critically ill patients are at risk of developing moderate to severe AKI [490]. The [TIMP‐2] × [IGFBP7] panel represents a transition in AKI management, from reactive identification toward proactive secondary prevention [491].

#### Major types and representative biomarkers

Traditional functional markers remain clinically relevant, with serum creatinine retaining its position as the diagnostic gold standard despite requiring contextual interpretation within clinical scenarios. Cystatin C, being independent of muscle mass, demonstrates superior early predictive capability; in trauma ICU patients, admission levels exceeding 0.78 mg/L predict AKI development within 1 week with greater sensitivity than creatinine [492]. Injury‐specific biomarkers have emerged as critical diagnostic tools due to their high sensitivity and specificity profiles. NGAL demonstrates rapid release following tubular injury and can be detected in both plasma and urine, exhibiting robust performance in identifying ischemic and nephrotoxic AKI [489]. KIM‐1 shows significant upregulation in proximal tubular cells post‐injury and displays high specificity for ischemia‐reperfusion injury [493]. The combined measurement of [TIMP‐2] × [IGFBP7], reflecting renal tubular cell cycle arrest, achieves an area under the receiver operating characteristic curve (AUC) of 0.792, making it valuable for early risk stratification [491]. Urinary IL‐18 participates in inflammatory cascades and demonstrates particular diagnostic utility in sepsis‐associated AKI. In prerenal AKI, urinary sodium concentrations decline 1‐2 days prior to AKI onset, with persistently low values indicating microcirculatory stress [494]. Multimarker panels enhance diagnostic precision: combining NGAL and KIM‐1 yields an AUC > 0.85 for early AKI detection, while integrating [TIMP‐2] × [IGFBP7] with the renal angina index enables precise identification of high‐risk patients requiring urgent intervention.

In the clinical management of AKI bedside ultrasound has become an important real‐time assessment tool. Contrast‐enhanced ultrasound (CEUS) enables quantitative evaluation of microperfusion in the renal cortex and medulla. Its corticomedullary perfusion index ratio (PI ratio) is significantly correlated with the severity of chronic tubulointerstitial injury and can predict renal function recovery [495]. Shear wave velocity (SWV) measured by SWE decreases progressively with the severity of AKI, and its combination with the PI ratio yields a diagnostic specificity for AKI of up to 90.9% [496]. The renal artery resistive index (RI) is significantly elevated in critically ill patients with AKI and is positively correlated with active tubulointerstitial injury [497]. These imaging markers can detect microvascular dysfunction prior to the elevation of serum creatinine, providing a critical time window for early intervention.

Prognostic and predictive biomarkers are essential for forecasting disease trajectories and tailoring therapeutic interventions. Among progression markers, urinary chemokine ligand 14 (CCL14) at concentrations ≥1.3 ng/mL effectively predicts progression to stages 2–3 AKI [498]. Dickkopf‐related protein 3 (DKK3), implicated in fibrogenesis, indicates an increased risk of transition from AKI to CKD when elevated [499]. Chitinase‐3‐like protein 1 (CHI3L1/YKL‐40), reflecting systemic inflammatory burden, demonstrates enhanced prognostic accuracy when combined with soluble urokinase plasminogen activator receptor [500, 501]. Peptidomic panels, including CKD273 and AKI204, enable prediction of AKI incidence and mortality through urinary peptide profiling [502]. For treatment response prediction, l‐FABP reflects renal tubular hypoxia, with serial measurements facilitating monitoring of therapeutic efficacy. Elevated mitochondrial DNA (mtDNA) levels predict responsiveness to renoprotective therapies, providing a rationale for treatment optimization.

#### Current challenges and future development trends

Despite these advances, biomarkers still face persistent challenges. Diagnostic sensitivity for KDIGO stage 1 AKI remains suboptimal and insufficient to meet the need for ultra‐early detection. Furthermore, many biomarkers are affected by extrarenal factors such as systemic inflammation and sepsis, which may reduce specificity and accuracy [503]. Technological barriers to clinical implementation include a lack of standardized assays, variable cutoff values between populations, and underpowered validation in special cohorts.

Future directions include the development of multimodal integration models that combine liquid biopsy data, physiological parameters, and artificial intelligence for precise disease phenotyping. Targeted discovery of injury‐specific biomarkers, particularly those associated with unique etiologies such as rhabdomyolysis, will improve specificity. Clinically, dynamic monitoring protocols using serial biomarker measurements will enable a transition from static assessment to continuous adaptive management, ultimately supporting personalized precision care throughout the disease trajectory.

### Glomerulonephritis

Glomerulonephritis is a heterogeneous group of diseases characterized by primary glomerular injury. Certain subtypes may rapidly progress to end‐stage renal disease. Given the marked variability in clinical outcomes, the development of biomarkers for early diagnosis, prognostic stratification, and treatment guidance has significant clinical value (Table S43) [504].

#### Disease classification system

Glomerulonephritis is primarily classified based on etiology and the anatomical extent of renal involvement. Clinically, it is divided into two main categories: primary and secondary glomerulonephritis. Primary glomerulonephritis accounts for over 70% of cases and includes several distinct pathological subtypes. IgA nephropathy is characterized by predominant IgA deposition in the mesangial region and displays significant clinical heterogeneity. Membranous nephropathy, caused by subepithelial immune complex deposition, is a leading cause of nephrotic syndrome in adults. Mesangial proliferative glomerulonephritis is characterized by mesangial cell hyperplasia and is often associated with preceding infectious triggers. Other primary subtypes, such as minimal change disease and focal segmental glomerulosclerosis, are not discussed in detail due to limited biomarker evidence. Secondary glomerulonephritis occurs in the setting of systemic diseases. Lupus nephritis is the most common renal manifestation of systemic lupus erythematosus (SLE), characterized by diffuse deposition of immune complexes. Other secondary forms, including ANCA‑associated vasculitis, Henoch‑Schönlein purpura nephritis, and anti‑GBM disease, are beyond the scope of this section. In addition, chronic glomerulonephritis represents the progressive phase of various glomerular disorders and is an important cause of end‑stage renal disease [505].

#### Development history of biomarkers

Historically, renal function assessment has been heavily reliant on conventional serum markers such as creatinine and BUN. However, these indicators only reflect established structural damage and functional decline, lacking sensitivity for the detection of early glomerular injury. Moreover, they lack discriminatory power among different subtypes of glomerular disease, thus limiting their utility in early diagnosis and precise classification [506].

With the advancement of technology, biomarker discovery has entered an omics‐driven era. Transcriptomic studies have revealed that the expression level of lncRNA NONRATG001910.2 is positively correlated with the degree of glomerulosclerosis, providing a potential molecular target for understanding disease progression pathways [507]. Proteomic analyses, utilizing high‐throughput screening, have identified urinary soluble CD163 (sCD163) as a specific biomarker for monitoring disease activity in IgA nephropathy [508]. MicroRNA profiling has facilitated the creation of a combined detection model using miR‐30a‐5p and miR‐106a‐5p, achieving a diagnostic accuracy of up to 89% for certain glomerular diseases, thereby significantly improving diagnostic performance [509].

Current research has progressed to the stage of multi‐omics integration. High‐throughput proteomic platforms such as SOMAscan enable the rapid identification of numerous candidate biomarkers [510]. Metabolomic approaches have revealed distinct patterns of metabolic dysregulation in membranous nephropathy, offering insights into subtype‐specific pathophysiology at the metabolic level [511]. Studies on the Gut microbiome have identified *Shigella* as a characteristic microbial signature in patients with IgA nephropathy, expanding the scope of biomarker discovery and laying the foundation for constructing integrated, multidimensional diagnostic frameworks [512].

GeoMx digital spatial profiling (DSP) has enabled transcriptome‐wide analysis of single glomeruli, revealing that *TGFBR3*, is specifically enriched in podocytes (WT1 + NPHS1 + cells) and exhibits significant spatial correlation with other podocyte‐specific genes such as *PODXL* and *CLIC5*. This spatial localization confirms *TGFBR3* as a locally retained biomarker of podocyte injury rather than a passive filtration product [513].

#### Main biomarker types

In the realm of diagnostic and screening methodologies, urinary N‐acetyl‐β‐d‐glucosaminidase serves as a reliable indicator of renal tubular damage and can predict the remission status of IgA nephropathy [514]. The inflammation‐related biomarker sCD163 holds significant reference value in evaluating disease activity in both IgA nephropathy and lupus nephritis. Within the domain of circulating microRNAs, a combined detection model of miR‐30a‐5p/miR‐106a‐5p has been successfully employed in the diagnosis of mesangial proliferative glomerulonephritis, thereby providing a molecular basis for the differentiation of pathological subtypes [509]. The identification of novel protein biomarkers further enhances the diagnostic landscape. Cathepsin S is highly expressed in the serum and renal tissue of patients with IgA nephropathy, offering a new target for disease mechanism research and diagnosis [515]. Additionally, transforming growth factor β receptor 3 (TGFBR3) can specifically identify a unique type of membranous nephropathy, suggesting that this subtype may have an autoimmune‐related pathogenesis basis [516].

Magnetic resonance diffusion tensor imaging (DTI) provides novel imaging biomarkers for the noninvasive assessment of glomerulonephritis [517]. By measuring parameters such as fractional anisotropy (FA) and mean diffusivity (MD), DTI allows sensitive detection of microstructural alterations in renal tissue, and can identify differences in corticomedullary FA values in the early stages of glomerulonephritis [518]. Diffusion kurtosis imaging (DKI) further enhances sensitivity to non‐Gaussian water diffusion, enabling detection of reduced cortical FA as early as Day 2 of the disease course, with characteristic time‐dependent and region‐dependent changes as the disease progresses [519]. These advanced MRI techniques offer noninvasive imaging biomarkers for the early diagnosis, pathological monitoring, and therapeutic evaluation of glomerulonephritis.

In membranous nephropathy, the IgG4 subtype of phospholipase A2 receptor (PLA2R) antibody is more effective in evaluating therapeutic outcomes and disease prognosis than other subtypes [520]. The expression level of serum soluble interleukin‐2 receptor α (sIL‐2Rα) correlates closely with histological damage severity in patients with IgA nephropathy, serving as a significant predictor for disease progression risk [521]. Immunoglobulin heavy chain profiling can accurately predict treatment responsiveness in membranous nephropathy patients, thereby informing adjustments to treatment plans [522]. Regarding metabolite biomarkers, urinary glycine has been identified as a protective indicator for IgA nephropathy, with its level changes reflecting disease prognosis trends [511].

#### Current challenges and development trends

Despite significant advancements in the research of biomarkers for glomerular diseases, there remain numerous challenges in both clinical application and technological development. Diagnostically, current biomarkers lack sufficient sensitivity to detect early stages of the disease, thereby failing to meet the requirements for early screening and intervention. Furthermore, glomerular diseases exhibit considerable heterogeneity, with distinct pathological mechanisms and clinical phenotypes across different subtypes [523]. This diversity hampers the creation and implementation of universal biomarkers. From a technology transfer perspective, the detection costs for most novel biomarkers are prohibitively high. Additionally, there is an absence of standardized detection methods and procedures, as well as large‐scale multicenter clinical validation data. These factors severely limit their transition from laboratory research to routine clinical use. In response to these challenges, the field of biomarkers for glomerular diseases has a clear developmental trajectory. Multimodal integration is emerging as a central trend, exemplified by the construction of a “miRNA‐protein‐metabolite‐microbiota” multi‐dimensional combined detection panel. This approach facilitates a comprehensive assessment of the disease, thereby enhancing the accuracy of both diagnosis and prognostic evaluations. technological innovation is a key driver in this field; spatial proteomics technology allows for precise analysis of molecular expression and spatial distribution within the renal microenvironment, while extracellular vesicle proteomics offers new avenues for biomarker discovery. These advancements collectively propel biomarker research towards greater precision and depth. The implementation of precision medicine concepts is a significant objective, with the widespread adoption of liquid biopsy technology enabling dynamic monitoring of disease progression. The integration of organoid models and drug sensitivity tests provides a direct foundation for individualized treatment strategies, and biomarker‐guided targeted therapy holds the promise of achieving “precise targeting, on‐demand treatment,” thereby significantly improving patient outcomes.

### Renal cell carcinoma

RCC is a highly heterogeneous malignancy within the urinary system, encompassing a broad pathological spectrum from indolent lesions to aggressive subtypes. Given that most patients are diagnosed at metastatic stages and prognosis varies significantly across subtypes, there is an urgent clinical need to develop a biomarker system capable of enabling early diagnosis, precise classification, and individualized treatment guidance (Table S44) [524].

#### Disease classification and pathological features

RCC is categorized into several subtypes based on tissue morphology, immunophenotype, and molecular characteristics.

In terms of common primary subtypes, clear cell RCC (ccRCC) accounts for over 70%, with *VHL* gene inactivation and abnormal activation of the hypoxia‐inducible factor pathway as its molecular core [525]; papillary RCC (pRCC) is subdivided into type I, driven by the *MET* gene, and type II, associated with *SETD2/NRF2* mutations [526]; clear cell papillary RCC exhibits a unique immunophenotype of CK7^+^/CA‐IX + and has a favorable prognosis [527].

In terms of special types, RCC encompasses translocation‐related variants associated with *TFE3/TFEB* gene fusion, as well as subtypes linked to genetic syndromes such as *VHL* syndrome and *HLRCC*, each characterized by a distinct molecular basis and clinical phenotype [528].

### Development of biomarkers

In terms of traditional biomarker stage, relying on non‐specific indicators such as serum LDH and VHL gene detection provides only limited information for disease monitoring, lacking the capacity for early diagnosis and classification [529, 530].

In terms of omics technology‐driven stage, multimodal omics technologies have ushered in a new era of systematic exploration in biomarker research [531]. Imaging omics enables the quantification of tumor metabolic and perfusion characteristics, while immunohistochemical markers (PAX8, CA‐IX) significantly enhance classification accuracy. Furthermore, liquid biopsy technologies (ctDNA, urine exosomes) provide novel non‐invasive diagnostic approaches, and inflammatory indicators (NLR, CRP) refine the pre‐clinical evaluation system.

Current research is centered on the integration of multi‐dimensional data, achieved through the development of composite biomarker models (such as the CBS score) and the application of artificial intelligence algorithms. This approach aims to accurately predict treatment response [532]. The advent of New molecular imaging probes, such as 18F‐VM4‐037, further propels the advancement of non‐invasive classification technology [533].

#### Main biomarker types and clinical applications

Among the diagnostic and screening biomarkers, PAX8, as a biomarker of renal origin tracing, has an irreplaceable value in the differential diagnosis of metastatic foci [534]. CA‐IX has a diagnostic specificity of over 90% for ccRCC, and markers such as *CD10*, *CK7*, and *AMACR* together constitute an immunohistochemical panel for subtype differentiation [535]. In terms of molecular testing, *MET* mutation guides pRCC classification, and *TFE3/TFEB* fusion gene is the basis for the diagnosis of translocation‐related RCC [527]. Serum sCA‐IX provides a reference for ccRCC screening, and combined detection of AQP‐1/PLIN2 in urinary exosomes has a sensitivity of 82% for early diagnosis [525, 536]. ctDNA can capture driver mutations such as *VHL* and *BAP1*, and its dynamic changes can more accurately reflect treatment response and drug resistance evolution [537]. The new CA‐IX targeted probe 18F‐VM4‐037 has a sensitivity of 92% for detecting metastatic foci of ccRCC, promoting imaging diagnosis from morphological evaluation to molecular typing [533].

Radiomics and radiogenomics have emerged as core digital biomarkers for the precise diagnosis of RCC. CT‐based radiomic models can differentiate clear ccRCC from non‐clear cell subtypes, with a logistic regression model achieving an AUC of 0.906, a sensitivity of 95.6%, and a specificity of 69.2%, outperforming conventional diagnosis by radiologists [538]. MRI radiomics combine d with machine learning enables accurate differentiation among ccRCC, papillary RCC, and chromophobe RCC, with a linear discriminant analysis model yielding an AUC of 0.959 [539]. Habitat imaging, by identifying subregions with distinct enhancement patterns within tumors, can predict the risk of distant metastasis postoperatively, with a specific subregion (Habitat 3) strongly associated with metastasis [540]. PET/CT tracers such as 18F‐FDG, 124I‐cG250, and radiolabeled PSMA further improve histological characterization, metastasis detection, and treatment response assessment [541]. Artificial intelligence‐assisted imaging analysis has achieved higher accuracy than radiologist interpretation in distinguishing benign from malignant renal masses.

Among the prognosis and prediction biomarkers, prognostic evaluation is fundamentally based on inflammatory indicators (NLR, CRP) and metabolic indicators (LDH) [542, 543]. Serum cytokines (IL‐6, IL‐8) [544, 545] and tissue molecular markers (*BAP1*, *TP53*) further refine risk stratification. Regarding therapeutic prediction [546], VEGF levels inform anti‐angiogenic therapy decisions [537], the CBS score specifically predicts mTOR inhibitor efficacy, and dynamic monitoring of ctDNA provides early response indicators for immunotherapy [532].

#### Challenges and prospects

Current research on biomarkers for RCC is confronted with challenges such as inadequate sensitivity for early diagnosis, significant disease heterogeneity, and high technical transformation barriers. Future directions encompass the construction of a multimodal integrated biomarker system through the integration of imaging omics, liquid biopsy, and clinical parameters. The promotion of frontier technologies like spatial omics and microbial metabolomics is also crucial. Additionally, establishing a dynamic monitoring network based on biomarkers to enable real‐time optimization of treatment strategies is vital. Through the profound integration of multi‐omics data and artificial intelligence, the diagnosis and treatment of RCC are transitioning towards an authentic era of precision medicine.

### Bladder cancer

Bladder cancer, the most common urinary malignancy, presents significant clinical challenges, including dependence on invasive cystoscopy for early detection, imprecise disease stratification, and inadequate treatment response prediction. Systematic integration of non‐subtype‐specific biomarkers offers a promising approach to precision diagnosis and therapy (Table S45) [547].

#### Development process of biomarkers

In terms of traditional body fluid biomarker stage, this stage is represented by urinary exfoliated cytology and nuclear matrix protein 22 (NMP22) has a specificity of nearly 100%, but it exhibits low sensitivity for detecting low‐grade tumors and its accuracy depends on the expertise of the pathologist [548]. NMP22 demonstrates higher sensitivity than cytology, yet its performance is compromised by benign conditions such as cystitis [549]. NMP22 was approved by the FDA in 1996 for the screening of bladder cancer recurrence [550]. Overall, the biomarkers in this category are predominantly single indicators that have not yet achieved widespread clinical adoption.

In terms of the exploration stage driven by omics technologies, the advent of omics technologies has propelled biomarker research into a new era of systematic screening. Transcriptomics has identified RNA biomarkers such as circHIPK3 [551], *PVT1* [552], and SKA3 [553], whose expression is strongly correlated with the onset and progression of bladder cancer; proteomics has discovered proteins in urine or serum, including PKCα [537] and LGALS3BP [554], some of which have been experimentally validated for their diagnostic value; at the epigenetic level, alterations such as *ECRG4* and *ITIH5* [555] gene promoter methylation and *TERT* promoter mutations represent promising avenues for early non‐invasive diagnosis [556]. Furthermore, circulating tumor cells and the urinary microbiome offer novel perspectives for prognostic assessment and therapeutic prediction.

Current research focuses on the integration of multi‐dimensional data, including nucleic acids, proteins, and microorganisms. This involves constructing composite biomarker panels and utilizing artificial intelligence algorithms to enhance diagnostic and prognostic efficacy. From a technical standpoint, the establishment of standardized detection protocols and the development of cost‐effective kits have propelled the transition of biomarkers towards clinical application. Furthermore, the use of liquid biopsy technology has facilitated dynamic monitoring of postoperative recurrence and treatment response.

#### Main biomarker types and clinical applications

Among the diagnostic and screening biomarkers, CircRNAs, owing to their stable structure and enrichment in urine, have emerged as ideal non‐invasive biomarkers. circHIPK3 [551], *hsa_circ_0137439* [557], circSTAG2 [558], etc., all showed good diagnostic performance, with some of which having an AUC greater than 0.85 and were associated with tumor stage and recurrence risk. lncRNAs [559] and mRNAs [560] also showed tissue or body fluid‐specific expression. In addition, *TERT* promoter mutations had a high detection rate in urinary ctDNA making them suitable for screening high‐risk populations [556]. Urinary proteins such as PKCα [553], LGALS3BP [554], etc. showed high diagnostic specificity and correlation with grade; the ratio of CUBN/MPO and proteins such as HOXA9 could effectively distinguish malignant from benign disease [561]. Serum proteins such as Epiplakin [562], PTX3 [563], sPD‐L1 [564] etc. were significantly present in advanced patients and demonstrated auxiliary diagnostic utility. DNA methylation markers including *ECRG4* and *ITIH5* [555] detected by quantitative methylation‐specific PCR (qMSP) in urine exhibited high specificity. Alterations in abundance of specific bacterial taxa within the urinary microbiome can be incorporated into a diagnostic classifier achieving an area under receiver operating characteristic curve (AUC) of 0.83 [555].

Artificial intelligence‐based image analysis has demonstrated significant potential in the diagnosis of bladder cancer. Deep learning models grounded in convolutional neural networks (CNN) conduct an analysis of blue‐light cystoscopy images, attaining a sensitivity of 95.77% and a specificity of 87.84% for tumor detection [565]. Specifically, the study utilized four pre‐trained CNN architectures, namely InceptionV3, MobileNetV2, ResNet50, and VGG16, and fine‐tuned them through transfer learning on a multicenter dataset consisting of 216 blue‐light images. Each network was augmented with additional layers, which incorporated batch normalization, a global average pooling layer, dropout with a rate of 50%, and a dense layer followed by Softmax activation. A leave‐10‐patients‐out cross‐validation strategy was adopted to assess model performance. The MobileNetV2 network achieved the highest mean sensitivity of 91.81% for malignancy detection. These results illustrate that transfer learning allows for the effective classification of bladder cancer from blue‐light cystoscopy images, even with a relatively small sample size. Weakly supervised deep learning models on whole‐slide images enable pathological grading of non‐muscle invasive bladder cancer, with an F1‐score of approximately 0.85 [566]. Hybrid deep learning models combining radiomics with CT/MRI achieve an accuracy of about 85% for tumor staging [567]. Computational pathology can also detect prognosis‐related morphological features such as tumor budding and predict overall survival [568]. Multimodal artificial intelligence, integrating imaging, pathological, and molecular data, provides comprehensive digital biomarker support for risk stratification and precise treatment decision‐making in bladder cancer [569].

Among the prognostic and predictive biomarkers, elevated expression of multiple mRNA biomarkers, such as *IGF2BP3* [570], is significantly associated with an increased risk of tumor recurrence and reduced overall survival. Long non‐coding RNAs including *PVT1*, as well as circular RNAs such as circRIMS1 and circPSMA7, promote tumor progression by regulating specific molecular pathways, and their expression levels hold prognostic value in predicting clinical outcomes [571]. Overexpression of proteins including GPR123 [572] and *CD276* [573] has been identified as an independent prognostic risk factor, while expression of VASH1 [574] and FTO [575] may help predict response to chemotherapy. Additionally, activation of signaling pathways such as SH3YL1‐NOX4 [576] is closely linked to treatment response and disease prognosis. Composite biomarker panels, including the three‐gene signature (*CDKN2A*, *CTSV*, *FOXM1*) [577], the 13‐mRNA prognostic signature [578], and the CUBN/MPO ratio [561], have demonstrated improved performance in risk stratification and therapy prediction relative to conventional staging systems, supporting more individualized treatment decisions.

#### Current challenges and future directions

Current biomarker research is confronted with several major challenges, including suboptimal sensitivity for early detection, confounding effects of tumor heterogeneity, lack of standardized assay protocols and insufficient integration into routine clinical workflow. Most candidate biomarkers remain restricted to preclinical or exploratory phases without successful translation into clinical practice. Future efforts will be directed toward multimodal integration to develop comprehensive biomarker systems that combine nucleic acids, proteins, microbiome profiles and imaging data, augmented by artificial intelligence to enhance analytical accuracy and predictive power. Emerging technologies such as spatial transcriptomics and exosome‐based analyses offer promising avenues for identifying highly specific and biologically relevant biomarkers. To advance precision oncology, individualized treatment approaches should incorporate organoid‐based drug sensitivity testing and dynamic biomarker monitoring. Moreover, the development of cost‐effective, standardized and scalab,le diagnostic platforms will be crucial to enable widespread implementation of biomarkers in primary and community healthcare settings.

### Spatiotemporal dynamic integration network of urinary system disease biomarkers

The urinary system, which functions as a pivotal excretory and endocrine organ, presents biomarkers with unique spatiotemporal dynamics that are distinct in their disease‐stage specificity, cellular compartmentalization, and responsiveness to pathophysiological disturbances. By comprehensively examining major urinary tract disorders such as AKI, CKD, RCC, and bladder cancer, we developed an integrated framework that is both multidimensional and temporally resolved. This framework encompasses early injury detection, dynamic risk assessment, functional trajectory observation, and longitudinal prognosis (Table S46).

#### Cascade‐activated biomarker network in acute kidney injury

KIM‐1, a type I transmembrane glycoprotein, is expressed at the lowest level in proximal tubular epithelial cells under normal physiological conditions. Following ischemia–reperfusion injury or nephrotoxic insult, KIM‐1 mRNA and protein levels increase 10‐ to 100‐fold within 2–6 h. Prospective cohort studies have established that each 1 ng/mg increase in creatinine in the urinary KIM‐1 concentration is associated with a 1.7‐fold increased hazard for progression to KDIGO stages 2–3 AKI, providing a predictive window of 24–48 h prior to serum creatinine elevation [579]. NGAL, which is rapidly synthesized and secreted by injured distal tubular and collecting duct cells, displays robust early diagnostic kinetics: urinary NGAL concentrations increase significantly within 2 h after cardiac surgery, peak at 4 h after surgery, and strongly inversely correlate with the GFR decline during the first 6 h after surgery (*r* = −0.66; *p* < 0.001) [580]. A comprehensive meta‐analysis involving 2526 patients undergoing cardiopulmonary bypass revealed that urinary NGAL concentrations ≥150 ng/mL yielded a pooled sensitivity of 0.78, specificity of 0.82, and area under the curve (AUC) of 0.88, surpassing the predictive performance of contemporaneous measurements of cystatin C and interleukin‐18 (IL‐18) for AKI prediction [581]. The urinary product of tissue inhibitor of metalloproteinases‐2 (TIMP‐2) and insulin‐like growth factor‐binding protein 7 (IGFBP7), deployed commercially as the NephroCheck® assay, provides biologically grounded risk stratification for impending moderate‐to‐severe AKI. In a prospective, multicenter Sapphire trial (*n* = 420), a TIMP‐2•IGFBP7 value > 0.3 (ng/mL)^2^/1000 measured within 4 h after surgery conferred a sevenfold increased risk of developing KDIGO stage 2–3 AKI within 12 h, with a negative predictive value of 94%. This test has received FDA clearance for use in critical care and perioperative settings. Importantly, the sustained upregulation of KIM‐1 is associated with maladaptive repair and correlated with NF‐κB and TGF‐β/Smad3 signaling. This, in turn, amplifies the production of IL‐6 and TNF‐α and establishes a self‐perpetuating “inflammation–fibrosis” axis, which is a mechanistic link implicated in the progression from AKI to CKD [582].

#### Fibrosis‐associated biomarker panel in chronic kidney disease progression

TGF‐β is a key regulator of renal fibrogenesis. Ligand binding activates Smad2/3 phosphorylation, which is associated with nuclear translocation of the Smad2/3–Smad4 complex and transcriptional reprogramming toward myofibroblast differentiation, as indicated by de novo α‑smooth muscle actin (α‑SMA) expression and the excessive synthesis of collagen I/III and fibronectin [583]. Urinary type III procollagen N‐terminal propeptide (PIIINP) reflects actual collagen biosynthesis rates; clinical validation confirmed its independent positive association with histopathological interstitial fibrosis scores, supporting its use as a noninvasive surrogate for ongoing matrix turnover in CKD [584]. Renal tissue α‐SMA expression is not only quantitatively associated with the fibrotic burden but also predicts a subsequent decrease in the eGFR and thus serves as a functional marker of myofibroblast activation and progressive parenchymal loss [585]. Together, these markers form a complementary pathophysiologically anchored panel that allows early identification of patients at high risk for rapid CKD progression and informs timely therapeutic escalation.

#### Integrated biomarker strategy for precision renal function assessment

Serum creatinine and cystatin C have complementary physiological determinants. Creatinine reflects muscle mass and tubular secretion, whereas cystatin C indicates glomerular filtration independent of lean body mass. Using both in validated equations such as the CKD‐EPI CysC‐Cr improves the accuracy of GFR estimation, particularly in populations with altered muscle mass, such as elderly, cachectic, or obese individuals. Large‐scale cross‐sectional analyses have shown that dual‐marker models reduce the median absolute error to less than or equal to 8.5% and reclassify 16.9% of individuals previously misclassified as having CKD solely on the basis of creatinine levels [586]. Both the urine protein‐to‐creatinine ratio and the UACR offer standardized quantitative assessments of global and selective glomerular permeability defects, respectively, which are strongly associated with podocyte injury and structural disruption of the basement membrane [587]. Furthermore, tubular injury biomarkers such as N‐acetyl‐β‐d‐glucosaminidase (NAG), a lysosomal enzyme released during proximal tubular stress, and retinol‐binding protein, whose urinary excretion indicates impaired megalin‐mediated reabsorption, provide early detection of nephrotoxicity before changes in creatinine levels occur, allowing for preemptive intervention [588, 589].

#### Evolutionary biomarker landscape in urologic malignancies

Bladder tumor antigen (BTA) and nuclear matrix protein 22 (NMP22) are clinically established urinary biomarkers for bladder cancer surveillance. BTA demonstrates an overall sensitivity of 67% across all tumor grades, substantially exceeding that of conventional cytology (43%) while maintaining acceptable specificity [590]. In a prospective study of 1331 high‐risk patients who presented with hematuria or prior malignancy, point‐of‐care NMP22 testing achieved 55.7% sensitivity and 85.7% specificity, supporting its role in reducing unnecessary cystoscopies during outpatient follow‐up [591]. For clear‐cell RCC (ccRCC), carbonic anhydrase IX (CAIX) functions as a hypoxia‐inducible oncomarker that is dependent on the VHL pathway. More than 90% of ccRCC tumors exhibit strong membranous CAIX expression, whereas normal renal parenchyma and nonclear‐cell subtypes show minimal or absent staining, establishing its diagnostic and theranostic relevance per international guidelines [592]. Emerging epigenetic biomarkers such as 5‐hydroxymethylcytosine (5‐hmC) further expand detection capabilities. In a cohort of 246 renal cancer patients, peripheral blood 5‐hmC concentrations at or above 0.15 ng/mL enabled the detection of early‐stage ccRCC with 85.4% sensitivity and 87.8% specificity; moreover, an inverse correlation with tumor diameter suggests its utility in identifying small renal masses and monitoring postresection recurrence [593].

### The urinary system as a central integrative hub in systemic physiology

Beyond excretion, the kidney functions as a master regulator of fluid–electrolyte homeostasis, metabolic clearance, and endocrine signaling, thereby serving as a pivotal node in multisystem crosstalk. Its biomarkers thus have cross‐organ prognostic and mechanistic significance (Table S47).

#### Cardiorenal axis

In heart failure, reduced cardiac output and venous congestion impair renal perfusion pressure, triggering compensatory activation of the renin–angiotensin–aldosterone system (RAAS) and the SNS. Angiotensin II and aldosterone promote sodium retention, whereas norepinephrine induces renal vasoconstriction, establishing a self‐amplifying cycle of volume overload, worsening congestion, and progressive renal hypoperfusion that culminates in cardiorenal syndrome (CRS) [594]. Concurrently, acute hemodynamic stress elevates both circulating B‐type natriuretic peptide (BNP) and tubular‐derived NGAL. Retrospective analyses revealed that the BNP/NGAL ratio is an independent predictor of 90‐day mortality or dialysis initiation in patients with CRS, with each unit increase conferring an 18% higher hazard, extending beyond that of conventional risk models [595]. Furthermore, a declining GFR impairs the systemic clearance of proinflammatory cytokines such as IL‐6 and TNF‐α, promoting a low‐grade inflammatory milieu that accelerates endothelial dysfunction and atherosclerosis, which are key drivers of adverse cardiovascular outcomes in patients with CRS [596].

#### Renal–bone–mineral axis

In CKD, impaired renal 1α‐hydroxylation of vitamin D and phosphate retention initiate a cascade of mineral dysregulation. As early as CKD stage 2, osteocytes and osteoblasts markedly upregulate fibroblast growth factor 23 (FGF‐23), which is associated with reduced renal 1α‐hydroxylase (CYP27B1) and increased 24‐hydroxylase (CYP24A1), thereby exacerbating active vitamin D deficiency and stimulating parathyroid hormone (PTH) secretion. This interaction forms a tripartite feedback loop that is central to CKD‐mineral and bone disorder (CKD‐MBD) [597, 598]. This dysregulation promotes cortical demineralization and marrow fibrosis, whereas hyperphosphatemia and an elevated calcium–phosphate product drive vascular smooth muscle cell osteogenic transdifferentiation and medial calcification, directly contributing to arterial stiffness, left ventricular hypertrophy, and increased cardiovascular mortality [599].

#### Immune–kidney axis

The kidney plays a key role in immune homeostasis, clearing immune complexes and cytokines while also producing context‐dependent immunomodulatory signals. In antibody‐mediated glomerulopathies, complement activation fragments such as C3a and the terminal membrane attack complex (C5b‐9) are elevated. Urinary C5b‐9 levels correlate with the severity of proteinuria in membranous nephropathy [600], and serum C3a concentrations reflect anti‐nucleosome antibody titers and help predict flare risk in patients with lupus nephritis [601]. Additionally, the urinary CD4^+^/CD8^+^ T‐cell ratio serves as a functional indicator of tubulointerstitial inflammation. A ratio greater than 2 signifies dense lymphocytic infiltration, which is associated with a better response to mycophenolate mofetil and a more rapid decrease in the eGFR [602, 603]. Together, these findings underscore the dual functions of the kidney as both a target and modulator of systemic immunity.

#### Renal–metabolic–endocrine axis

The kidney plays a major role in systemic glucose regulation through three key mechanisms. The first is gluconeogenesis, which accounts for approximately 20% of total endogenous glucose production during fasting and is mediated by proximal tubular phosphoenolpyruvate carboxykinase (PEPCK). Another is insulin clearance, mediated by renal insulinase, which reduces the half‐life of plasma insulin to approximately 4 min. The third is glucagon degradation, which is facilitated by proximal tubular glucagon receptors [604]. The urinary C‐peptide‐to‐creatinine ratio (UCPCR) serves as a noninvasive surrogate marker of the β‐cell secretory capacity. Cross‐sectional data have shown that UCPCR decreases progressively with increasing albuminuria and increases with increasing eGFR, often before changes in serum C‐peptide become detectable [605]. Sodium–glucose cotransporter 2 (SGLT2), located primarily on the S1 segment of the proximal tubule, is upregulated under hyperglycemic conditions. This upregulation promotes increased reabsorption of filtered glucose and helps perpetuate hyperglycemia. By interrupting this cycle, SGLT2 inhibitors lower blood glucose while also reducing intraglomerular pressure, providing a mechanistic explanation for their proven renoprotective effects in DKD [606].

#### Cross‐system prognostic value of urinary biomarkers

Microalbuminuria extends beyond its role as a marker of glomerular injury; it is a well‐validated indicator of systemic endothelial dysfunction and can independently predict the occurrence of cardiovascular events across diverse populations [607]. Similarly, serum uric acid functions as a pleiotropic metabolic sentinel. Elevated serum uric acid levels are associated with the development of hypertension, metabolic syndrome, and the progression of CKD, reflecting underlying oxidative stress, mitochondrial dysfunction, and dysregulated purine metabolism (Figure S5) [608].

## RESPIRATORY SYSTEM

A systematic literature search was conducted in PubMed and Web of Science using Boolean logic with field‐restricted search strings targeting the title/abstract fields. The detailed Boolean queries were constructed for each respiratory disease as follows: for chronic obstructive pulmonary disease (COPD), (“chronic obstructive pulmonary disease” OR “pulmonary disease, chronic obstructive”) AND (“biomarker”) AND (“disease”); for asthma, (“asthma”) AND (“biomarker”) AND (“disease”); for pneumonia, (“pneumonia”) AND (“biomarker”) AND (“disease”); for tuberculosis, (“tuberculosis”) AND (“biomarker”) AND (“disease”); for non‐small cell lung cancer (NSCLC), (“non‐small cell lung cancer” OR “carcinoma, non‐small‐cell lung”) AND (“biomarker”) AND (“disease”); for small cell lung cancer (SCLC), (“small cell lung cancer” OR “small cell lung carcinoma”) AND (“biomarker”) AND (“disease”); and for pleural mesothelioma, (“pleural mesothelioma” OR “mesothelioma, malignant”) AND (“biomarker”) AND (“disease”). The retrieval period was defined as January 1, 2020 through December 31, 2025. Only articles published in English were considered. Both original research articles and systematic or narrative reviews were eligible for inclusion. Retrieved records were screened by titles and abstracts; articles deemed irrelevant to respiratory disease biomarkers, lacking full‐text availability, or containing incomplete data were excluded, and the remaining full texts were thoroughly evaluated. The selected disease spectrum encompasses COPD, asthma, pneumonia, tuberculosis, NSCLC, SCLC, and pleural mesothelioma, all representing conditions with high incidence, substantial disease burden, well‐established biomarker frameworks, and strong clinical translational relevance.

### Pneumonia

Pneumonia, an inflammatory disease of the lungs, is caused by pathogen infection or damage from physicochemical and immune factors. Its primary clinical manifestations include fever, cough, sputum production, and dyspnea. The severity of the condition is closely tied to the type of pathogen, the patient's age, and their immune status (Table S48) [609].

#### Development process of pneumonia biomarkers

Among the traditional biomarkers, C‐reactive protein (CRP), an acute phase protein, increases rapidly following infection. Its concentration is positively correlated with the severity of inflammation and is widely utilized for pneumonia screening and disease assessment. the development of ultrasensitive detection technology has enabled the measurement of high‐sensitivity CRP (hs‐CRP), which improves the ability to identify low‐grade inflammation. Procalcitonin (PCT) levels increase significantly during bacterial infections but do not change appreciably in viral infections; consequently, PCT is frequently used as an adjunct in the diagnosis, prognostication, and antibiotic stewardship of bacterial pneumonia.

Among the molecular biomarkers, advancements in molecular biology have shifted the focus of research towards molecular markers such as genes, miRNAs, and lncRNAs. These markers play a crucial role in the onset and progression of pneumonia, thereby offering novel avenues for early diagnosis, prognosis evaluation, and personalized treatment strategies.

Current research is centered on the integration of multi‐omics and its clinical translation, utilizing organoid models, spatial omics, and artificial intelligence to construct a comprehensive marker system. This system can be employed for early diagnosis, prognosis prediction, and treatment guidance.

#### Main types and typical cases of pneumonia biomarkers

Among the diagnostic and screening biomarkers, CRP, as a classic inflammatory indicator, increases rapidly after the onset of pneumonia and participates in anti‐infection immunity by activating complement and promoting phagocytosis [610]. PCT is elevated in bacterial infection, which has great value in differentiating between bacterial and viral pneumonia [611]. In 2017, the FDA approved PCT as a diagnostic aid to guide antibiotic treatment decisions for acute LRTIs [612]. Serum amyloid A (SAA) is an acute phase protein with high sensitivity, which is helpful for early identification and preliminary determination of pneumonia [613]. IL‐6, as a key cytokine in the early stage of inflammation, is released earlier than CRP and PCT, which can reflect the intensity of initial inflammation [614]. PTX3, as a long pentraxin protein, has high tissue specificity and helps to distinguish infectious from non‐infectious lung diseases [615].

Among the prognosis and surveillance biomarkers, Pro‐ADM levels are positively correlated with the severity of pneumonia and systemic inflammatory response, and persistently elevated levels indicate a poor prognosis [616]. As a marker associated with cardiac function, NT‐proBNP was significantly increased in pneumonia complicated by increased cardiac load, and its dynamic changes could assess the risk of disease progression and death [617]. FGF‐21 is highly expressed after lung tissue injury and involved in anti‐inflammatory and repair processes, and persistently high post‐treatment levels indicate poor tissue repair and complication risk [617].

Among the image markers and digital markers, chest CT can assist in differentiating bacterial, viral, and fungal pneumonia by lesion distribution, morphology (consolidation, ground‐glass shadow, nodules), and concomitant manifestations (pleural effusion, cavity), and assess the extent of lesions and complications [618]. AI‐based image‐assisted diagnostic systems can quantitatively assess the inflammatory burden of the lungs and improve early identification capabilities. Clinical risk scores constructed by combining vital signs, laboratory parameters, and imaging features in electronic medical records have been widely used for disease stratification [619].

#### Current challenges and future development trends

Research on pneumonia biomarkers continues to face challenges, including insufficient sensitivity for early diagnosis, histological heterogeneity, and the absence of a standardized verification system [620]. Future studies should prioritize the development of an integrated multi‐omics diagnostic model that combines multi‐dimensional data, such as the proteome, transcriptome, and exosomes, to establish a high‐precision prediction system. The combined use of organoids and drug susceptibility testing platforms will facilitate the advancement of individualized treatment strategies and the precise secondary prevention and control of pneumonia.

### Tuberculosis

Tuberculosis, a chronic respiratory infectious disease caused by *Mycobacterium tuberculosis*, is primarily transmitted through airborne droplets. Despite the effectiveness of modern multi‐drug combination chemotherapy in curing most patients, significant challenges persist globally. These include the spread of drug‐resistant tuberculosis and HIV co‐infection, necessitating more advanced diagnostic methods and prevention and control strategies (Table S49) [621].

#### Development process of tuberculosis biomarkers

In terms of early exploration stage, early diagnosis of tuberculosis (TB) primarily depends on sputum smear microscopy and TB culture, both of which are time‐consuming and necessitate high laboratory standards. As our understanding of the immune mechanisms of TB deepens, researchers have begun to investigate the use of specific molecules in blood, urine, and other samples as supplementary diagnostic tools. This marks the advent of TB biomarker research.

In terms of discovery and application of pathogen biomarkers, the advancement of molecular diagnostic techniques has facilitated the use of pathogen‐specific markers [622]. For instance, the GeneXpert MTB/RIF system allows for concurrent detection of *Mycobacterium tuberculosis* and rifampicin resistance within approximately 2 h. This system exhibits high sensitivity in smear‐positive samples but demonstrates reduced efficacy in samples with low bacterial counts.

In terms of omics integration and clinical translation, the current research has entered the stage of multi‐omics integration and clinical translation. By integrating transcriptome, proteome, and metabolome data with organoid models and artificial intelligence methods, this approach aims to construct a new marker system for early diagnosis, prognostic evaluation, and therapeutic guidance. For example, spatial metabolomics has revealed unique metabolic signatures within tuberculosis granulomas. Lipid metabolites are specifically enriched in the necrotic core, providing survival factors for *Mycobacterium tuberculosis* and contributing to local immune evasion. This spatial insight offers a new perspective for understanding bacterial persistence and identifying therapeutic targets [623].

#### Main types and typical cases of tuberculosis biomarkers

Among the diagnostic and screening biomarkers, *mycobacterium tuberculosis* DNA is an important basis for etiological diagnosis. Nucleic acid amplification systems such as GeneXpert have proven their detection efficacy in a variety of samples, including tissue, urine and cerebrospinal fluid, enabling direct identification of the disease at the pathogen level [624]. Whole blood transcriptome studies identified gene expression signatures centered on neutrophil and type I interferon responses. *FCGR1B* and other genes were significantly upregulated in active tuberculosis, and combined with other immune‐related genes could effectively distinguish between active and latent infection [625]. MicroRNA expression profiling revealed abnormal expression of molecules such as hsa‐miR‐196b in patients with tuberculosis, showing their potential as diagnostic markers [626].

As a systemic inflammatory marker, CRP can be quantitatively analyzed using rapid detection technology. The WHO recommends its use for screening of TB in HIV‐infected individuals with cutoff value set at >5 mg/L [627]. Neutrophil to lymphocyte ratio can be used as an indicator of inflammation but has limited specificity in the diagnosis of tuberculosis. Monocyte to lymphocyte ratio is more specific because myeloid cells are primarily involved in TB infection and have shown good diagnostic potential in both adult and pediatric patients [628]. Using NMR and mass spectrometry, researchers have identified several metabolites associated with tuberculosis among which ketone bodies, lactate and pyruvate show high values in differentiating tuberculosis from other inflammatory and metabolic diseases [629].

Among the prognostic and surveillance biomarkers, owing to the short half‐life of mycobacterial mRNA and its ability to reflect bacterial activity through concentration changes, mRNA‑based detection methods hold unique value for dynamically monitoring treatment response [630].

Among the image markers and digital markers, chest X‐ray can reveal typical changes such as lung apical infiltrates, cavities, nodules, and mediastinal lymphadenopathy. CT can more clearly show the “bud signs” of micronodules, the thickness of the cavity wall and the range of bronchial dissemination, which is valuable in judging activity and identifying drug‐resistant tuberculosis [631]. AI‐assisted image interpretation systems have been used for large‐scale screening in resource‐limited areas, significantly improving diagnostic consistency. Based on the medication video (video‐supervised treatment) and treatment adherence data collected by mobile terminals, the treatment implementation can be effectively monitored [632].

#### Current challenges and future development trends

In terms of existing shortcomings and challenges, the primary challenge remains the inadequate sensitivity of early diagnosis. Significant heterogeneity was observed in the expression of markers across different pathological types. The majority of novel markers lack validation through large‐scale, multicenter studies and standardized detection protocols.

In terms of future development direction, the future study should construct a multi‐omics integrated diagnostic model by fusing multi‐dimensional data, including the proteome, transcriptome, and exosomes. Furthermore, it sought to promote the application of liquid biopsy technology for early screening and efficacy monitoring. An organoid model of tuberculosis was also established to screen for chemotherapy‐sensitive populations in combination with functional experiments.

### Chronic obstructive pulmonary disease

COPD is a prevalent respiratory disorder characterized by persistent airflow limitation, clinically manifesting as chronic cough, sputum production, and dyspnea on exertion. According to the WHO, COPD is projected to become the third leading cause of death worldwide by 2030 (Table S50) [633].

#### Development process of COPD biomarkers

Imaging biomarkers offer a non‐invasive approach to COPD assessment. CT can accurately measure the extent of emphysema, airway wall thickness, and pulmonary vascular remodeling, thereby aiding in disease staging and phenotypic characterization.

In terms of multi‐omics technology‐driven exploration, the advent of multi‐omics technology has ushered in a new era of systematic screening in COPD biomarker research. The utilization of multi‐dimensional analysis methods, including proteome, transcriptome, and metabolome, has facilitated the identification of novel markers. For instance, protein markers with high expression in lung tissue have been discovered, offering fresh insights into disease mechanism elucidation and early diagnosis. For example, spatial proteomics has enabled the mapping of immune cell distribution within lung tissue of COPD patients. Studies reveal that the spatial aggregation patterns and cell‑cell contact frequencies of specific immune cells correlate more strongly with emphysema severity and lung function decline than single blood inflammatory markers do. This spatial information provides new insights into disease heterogeneity and offers potential targets for precise phenotyping and local intervention [634].

Current research is focused on developing a multi‐omics‐driven precision medicine model. The integration of spatial omics, organoid models, and artificial intelligence algorithms is facilitating the establishment of a composite marker system with value for early diagnosis, prognostic assessment, and therapeutic guidance.

#### Main types and typical cases of COPD biomarkers

Among the diagnostic and screening biomarkers, CRP is often elevated in patients with COPD, particularly during acute exacerbations, and its levels are associated with cardiovascular event risk and mortality, but it lacks disease specificity due to its extensive involvement in systemic inflammatory responses. Notably, a Mendelian randomization study found that CRP mediated only 3.93% of the total effect of certain risk factors on COPD, suggesting that CRP is more likely to be a downstream inflammatory marker rather than a causal driver of disease pathogenesis [635]. Plasma fibrinogen levels were significantly associated with the prevalence of COPD, frequency of exacerbations, and all‐cause mortality. Plasma fibrinogen was certified by the FDA in 2015 as a prognostic or enrichment biomarker for all‐cause mortality and COPD exacerbations. It is the first biomarker drug development tool eligible for COPD use under the FDA's *Drug Development Tool Qualification Program* [636]. In addition, MCP‐4 and eotaxin‐3 were upregulated in airway samples from patients with COPD, showing potential for differentiating between patients and healthy individuals and discriminating disease activity status, which has value as auxiliary diagnostic markers.

In the field of circulating nucleic acids and epigenetic markers, microRNAs, such as miR‐155, miR‐17, and miR‐181, display distinct expression profiles in patients with COPD. Among these, miR‐181 demonstrates high sensitivity, while downregulation of miR‐17 may be linked to tissue remodeling processes. Integrated analysis of multiple miRNAs holds promise for enhancing the accuracy of COPD diagnosis and disease stratification [637].

Among the prognostic and predictive biomarkers, molecules associated with extracellular matrix remodeling, including MMPs and TIMPs, as well as markers of endothelial dysfunction, such as endothelin‐1 and von Willebrand factor, may provide valuable prognostic information for assessing the progression of COPD and the risk of cardiovascular complications [638].

Among sputum biomarkers, an increased eosinophil count indicates a higher risk of acute exacerbation and good response to glucocorticoid therapy, which has been used as a predictive marker to guide treatment decisions. Sputum proteomic and metabolomic analysis showed potential in identifying disease phenotypes and features associated with treatment responses. Lung function parameters such as the FEV_1_/FVC are core objective measures for assessing airflow limitation and aid in the diagnosis and grading of COPD [639].

Among the image markers and digital markers, high‐resolution CT is an important means of COPD phenotypic assessment. Quantitative CT can measure the percentage of areas of low attenuation (%LAA) to assess emphysema severity and quantify airway remodeling by parameters such as airway wall thickness and lumen area. These imaging markers not only help distinguish between emphysema‐dominant and airway‐dominant COPD, but also predict disease progression and risk of acute exacerbation [640]**.** Based on the daily activity level, heart rate variability, and nocturnal blood oxygen saturation monitored by wearable devices, the patient's functional status and disease stability can be reflected in real time. The digital lung function monitoring system built in combination with mobile medical technology can realize remote dynamic assessment of key indicators such as FEV_1_, providing a quantitative basis for the management of COPD chronic diseases.

#### Current challenges and future development trends

In terms of current challenges, COPD biomarker research still faces multiple challenges. The disease is highly heterogeneous and it is difficult for a single marker to fully reflect the complex clinical phenotype. Most of the existing candidate markers lack disease specificity. The translational efficiency from basic discovery to routine clinical application is low. And there is a lack of sensitive panel of markers that can identify early patients before significant decline in lung function.

Future research will likely focus on several key trends: promoting precision medicine, such as guiding the use of inhaled glucocorticoids based on blood eosinophil levels; strengthening technology‐driven approaches by integrating multi‐omics, artificial intelligence, and breath volatile organic compound analysis to construct high‐precision composite marker models; emphasizing multi‐dimensional integration and dynamic monitoring by combining clinical, imaging, and molecular data to develop a marker system that can reflect disease activity and treatment response in real time; and prospectively focusing on the research and development of biomarkers for early screening, risk prediction of acute exacerbations, and targeted therapy, thereby providing support for the comprehensive management of COPD.

### Asthma

Asthma, a chronic airway disease affecting over 300 million patients globally, exhibits significant clinical heterogeneity. Its pathological features include variable airflow obstruction and airway hyperresponsiveness. These are often triggered by environmental factors such as allergens, resulting in episodic and reversible bronchoconstriction (Table S51) [639].

#### Development process of asthma biomarkers

In terms of exploration of inflammatory cells and mediators, early studies focused on inflammatory cells and related mediators. As the core effector cells, increased proportion of sputum eosinophils (>3%) was significantly associated with the degree of airway inflammation, frequency of acute exacerbations, and poor disease control [641]. This stage established the relationship between the level of airway inflammation and clinical phenotype, providing a cytological basis for phenotypic division.

In terms of multi‐omics technology‐driven exploration, the advent of multi‐omics technologies, including proteome, transcriptome, and metabolome applications, has propelled asthma biomarker research into a phase of systematic screening. This shift in research focus has moved from singular inflammatory indices to comprehensive molecular characterization analysis. The objective is to uncover novel markers with significant diagnostic potential and elucidate their pathological mechanisms.

Current research efforts are dedicated to the development of a multi‐omics integrated precision medicine system. Utilizing organoid models, spatial omics, and artificial intelligence algorithms, researchers are advancing the discovery and application of novel marker combinations for early detection, prognostic evaluation, and therapeutic decision‐making.

#### Main types and typical cases of asthma biomarkers

Among the diagnostic and screening biomarkers, peripheral blood eosinophil count is a non‐invasive indicator of eosinophilic airway inflammation, and its elevation indicates a Th2 inflammatory state and is associated with the risk of acute asthma exacerbation. However, this indicator also appears in other allergic diseases with limited specificity [642]. As a key mediator of type I hypersensitivity reactions, serum total IgE levels are significantly elevated in allergic asthma and negatively correlated with lung function parameters, which has long been considered an auxiliary basis for assessing disease activity [643].

Fractional exhaled nitric oxide (FeNO) is a gaseous molecule produced by airway epithelium under the induction of Th2 cytokines such as IL‐13, which can reflect the level of eosinophilic inflammation more specifically. FeNO > 50 ppb (children > 35 ppb) suggests a Th2 hyperresponsive phenotype and is helpful in evaluating the response to inhaled glucocorticoid therapy. FeNO is a core marker of eosinophilic airway inflammation, but there are significant racial differences in its normal reference range and diagnostic cut‐off. Several studies have shown that non‐Hispanic black children have significantly lower levels of FeNO than white children after adjusting for age, height, and allergy status, while Hispanic people fall somewhere in between. If ≥35 ppb (children) is used uniformly as the criterion for Th2 hyperresponsiveness phenotypes, it may lead to black children with asthma being incorrectly classified as non‐eosinophilic phenotypes, thus missing out on ICS treatment opportunities. Similarly, the distribution of serum total IgE levels varies greatly among different ethnicities, and their weight in asthma diagnosis needs to be corrected for population context [644]. Although it is disturbed by factors such as smoking and diet, it still has great value in asthma phenotyping and management. Cytokines such as IL‐4, IL‐5, IL‐13 and alarmins derived from epithelium (TSLP, IL‐33, IL‐25) are elevated in sputum or bronchoalveolar lavage fluid, which are direct evidence of Th2 inflammation. A Mendelian randomization study based on a European population of 522,681 mimicked IL‐6 receptor (IL‐6R) blockade effects through 26 genetic variants and found that downregulation of the IL‐6R signaling pathway significantly reduced the risk of asthma (OR = 0.82, 95% CI: 0.74–0.90) and was also protective against COPD (OR = 0.71) [645].

Among the prognostic and predictive biomarkers, periostin, an extracellular matrix protein expressed downstream of IL‐4 and IL‐13, is significantly elevated in the serum of patients with eosinophilic phenotype asthma [646]. Research indicates that periostin serves as an effective predictor for airway eosinophil enhancement. Furthermore, its discriminant performance within multiple logistic regression models surpasses other inflammatory parameters.

Among the image markers and digital markers, functional imaging techniques such as CT can assess airway remodeling indicators such as airway wall thickening (WA%), air retention (gas trap index), and mucus plug score to predict disease progression and risk of acute exacerbation [647]. Peak expiratory flow rate variability can be assessed at home, and the rate of variability can be automatically recorded and calculated to assist in asthma diagnosis and monitoring [648].

#### Current challenges and future development trends

Most candidate markers are systemic inflammatory markers, which makes it difficult to distinguish asthma from other inflammatory or allergic diseases [649]. The efficiency of translational research from basic to clinical is low and standardized testing systems and validation platforms are lacking. There is a lack of sensitive marker panels that can identify high‐risk or early stage patients before significant decline in lung function.

In terms of future development trend, the paradigm of asthma management is shifting from the traditional “unified diagnosis and treatment” approach to endotyping based on biomarkers. Successful examples include inhaled glucocorticoid therapy strategies guided by blood eosinophil levels. By integrating technologies such as multi‐omics, artificial intelligence, and exhaled volatile organic compound analysis, high‐precision composite marker models can be constructed. A multi‐dimensional evaluation system should be established by integrating clinical, imaging, and molecular data. Furthermore, dynamic monitoring markers must be developed to reflect disease activity and treatment response in real time. This research should focus on early screening, providing early warnings for acute exacerbation risk, and predicting the efficacy of targeted biologics, with the ultimate goal of promoting the precision and individualization of the entire asthma management process.

### Non‐small cell lung cancer

NSCLC is the predominant pathological type of lung cancer, constituting approximately 80%–85% of all cases. Owing to its insidious onset and absence of effective early screening modalities, most patients are diagnosed at intermediate to advanced stages, resulting in suboptimal treatment efficacy and a dismal 5‐year survival rate of less than 5% for those with advanced disease (Table S52) [650].

#### Biomarker development process

In terms of application of traditional tumor markers, serum markers, such as CEA, squamous cell carcinoma antigen, and cytokeratin 19 fragments, are widely employed in auxiliary diagnosis and disease monitoring [651]. However, these markers can also be elevated in benign lung diseases, indicating a lack of specificity. Therefore, they must be comprehensively evaluated in conjunction with imaging and pathological examinations.

In terms of exploration of targeted driver genes, advancements in molecular biology technology have paved the way for precision targeted therapy for NSCLC, marked by the discovery of driver genes such as *EGFR* and *ALK* [652]. Researchers are now concentrating on identifying molecular markers that can predict the efficacy of targeted treatments, thereby establishing the groundwork for personalized treatment strategies.

Current research has advanced to the stage of multi‐omics technology integration, wherein a multimodal marker system is constructed by integrating genomic, transcriptomic, proteomic, and imaging features. This approach aims to facilitate early screening, dynamic monitoring of treatment efficacy, and optimization of therapeutic strategies.

#### Biomarker types and typical cases

Among traditional serum markers, CEA exhibits heightened sensitivity in lung adenocarcinoma and is frequently employed for recurrence monitoring [653]. Cytokeratin 19 fragment levels can assist in evaluating disease progression. MicroRNA markers, which promote tumor development through the regulation of tumor suppressor pathways, show serum levels significantly correlated with the risk of NSCLC [654]. Circulating tumor DNA analysis offers a non‐invasive diagnostic alternative for patients from whom tissue samples are unavailable, by detecting mutations in genes such as *EGFR* and *KRAS* in the blood [655].

Among the targeted therapy‐related markers, EGFR mutations occur in approximately 50% of Asian lung adenocarcinoma, with exon 19 deletion and exon 21 L858R point mutation being sensitive to tyrosine kinase inhibitor therapy [656]. *ALK* fusion is more common in young non‐smoking patients with adenocarcinoma, with an incidence of 3−7%, and inhibitors such as crizotinib are highly effective [657]. The incidence of *ROS1* fusion is about 1−2%, and its inhibitor treatment is also highly effective [658]. *BRAF* ^V600E^ mutations are mainly observed in lung adenocarcinoma, and the combination of *BRAF* inhibitors with MEK inhibitors can improve patient outcomes [659].

Among the immunotherapy‐related markers, PD‐L1 is currently the most commonly used predictor of immune efficacy, and patients with high expression have a higher response rate to PD‐1/PD‐L1 inhibitor monotherapy. Tumor mutational burden reflects tumor antigenicity by quantifying the number of genomic mutations and can be used as a complementary predictor for PD‐L1 negative patients. Although mismatch repair deficiency/microsatellite instability only occurs in 1−3% of cases, it is an advantageous group for immunotherapy [660].

Among the prognostic assessment markers, high expression of VEGF is closely related to tumor angiogenesis and invasion and metastasis, indicating a poor prognosis and serving as a reference for the efficacy of anti‐angiogenic therapy [661]. The Ki‐67 proliferation index is positively correlated with the risk of tumor recurrence. The number and pattern of tumor infiltrating lymphocytes can predict the effectiveness of immunotherapy, and patients with high infiltration have more significant survival benefits [662]. Serum levels of CYFRA21‐1 and CEA are important factors affecting the survival of advanced NSCLC patients and may be reliable biomarkers for predicting the effectiveness of immunotherapy in NSCLC patients [663].

Among the image markers and digital markers, low‐dose spiral CT is the gold standard for lung cancer screening and can significantly reduce mortality in high‐risk populations [664]. The metabolic parameters of PET‐CT can reflect tumor burden and biological aggressiveness, which can be used for accurate staging and treatment response assessment [665]. The pathological image analysis system based on deep learning can automatically identify the expression level of PD‐L1 and the density of tumor‐infiltrating lymphocytes. At the same time, the multimodal artificial intelligence model integrating imaging, pathology and clinical data demonstrates the core value of digital markers in predicting recurrence risk and individualized adjuvant treatment decisions [666].

#### Existing challenges and development trends

The sensitivity of early diagnostic markers is inadequate, hindering effective early screening. Tumor heterogeneity compromises the stability and reliability of marker expression. There is a lack of standardization in detection methods, particularly regarding unified thresholds for emerging biomarkers such as tumor mutation burden. The complexities and variabilities of drug resistance mechanisms make it challenging to accurately predict treatment response using current markers.

In terms of future development direction, the integration of multi‐omics is poised to become a mainstream strategy, enhancing diagnostic accuracy through the construction of multi‐dimensional marker models. Refinements in liquid biopsy technology will facilitate the widespread application of circulating tumor DNA, circulating tumor cells, and exosomes for dynamic monitoring. Organoid models are anticipated to replicate the heterogeneity of the tumor microenvironment, thereby informing individualized therapeutic strategies. Emerging technologies, including single‐cell sequencing and spatial omics, are expected to expedite the discovery and validation of novel biomarkers.

### Small cell lung cancer

SCLC is a highly malignant neuroendocrine tumor originating from bronchial mucosal silver cells, accounting for 15–20% of all lung cancers and closely associated with smoking (Table S53) [650].

#### Development process of SCLC biomarkers

In terms of era of traditional clinical markers, serum markers, notably neuron‐specific enolase (NSE) and gastrin‐releasing peptide precursor (ProGRP), are utilized in clinical practice due to their association with the neuroendocrine properties of SCLC [667]. These markers primarily aid in auxiliary diagnosis and monitoring treatment efficacy. However, their specificity is limited as they can also be elevated in certain benign lesions or other tumors. Therefore, their interpretation necessitates correlation with imaging studies and clinical manifestations.

With the advent of molecular biology, research has focused on driver gene and signaling pathway aberrations such as *MYC* family amplification, *FGFR1* alterations, and NOTCH pathway dysregulation. These findings not only shed light on the molecular underpinnings of SCLC but also provide a basis for developing targeted agents and clinical translation of relevant biomarkers.

Current research in SCLC has advanced to a stage of multi‐omics integration and precision medicine, wherein the integration of multi‐dimensional data from the genome, transcriptome, and proteome, combined with liquid biopsy and artificial intelligence technologies, facilitates the construction of a biomarker panel with high discriminative efficiency for early identification, prognostic stratification, and individualized treatment.

#### Main types and typical cases of SCLC biomarkers

Among the diagnostic and screening biomarkers, neuron‐specific enolase (NSE), a key glycolytic enzyme, is highly expressed in SCLC and has a diagnostic sensitivity of approximately 60−80% and specificity of 70−85%, making it commonly used for differentiating between SCLC and NSCLC as well as for initial screening in high‐risk populations. However, caution should be exercised as elevated levels can also be observed in other neuroendocrine tumors and benign lung diseases. The ProGRP shows significant elevation in the serum of SCLC patients, achieving a diagnostic sensitivity of 70−90% and specificity of 85−95%. Notably, its increase often precedes imaging abnormalities, offering potential for early screening and serving as an indicator for monitoring disease progression and recurrence [668]. Additionally, CTCs in the peripheral blood serve as a non‐invasive, real‐time marker of tumor burden in SCLC patients. Their numbers are significantly increased, presenting a diagnostic sensitivity of about 65−80% and specificity of 90−95%, thereby providing valuable reference for early diagnosis and staging [669].

Among the prognostic and predictive biomarkers, amplification of the *MYC* family, observed in 20−40% of SCLC cases, correlates with highly aggressive tumors, early metastasis, and chemotherapy resistance. It is an important indicator of poor prognosis and prediction of chemotherapy efficacy. The expression of PD‐L1 is heterogeneous, with a positive rate of approximately 10−30%. Patients with PD‐L1 positivity respond better to ICIs, and PD‐L1 positivity can be used as a predictive marker of immunotherapy efficacy. However, current detection standards have not been unified. The multidrug resistance gene (*MDR1*) encodes P‑glycoprotein, which can efflux chemotherapeutic drugs, leading to multidrug resistance. High expression of MDR1 suggests a high risk of resistance to etoposide, paclitaxel, etc., which helps individualize the selection of chemotherapy regimens.

#### Current challenges and future development trends

In terms of existing deficiencies and challenges, existing markers lack sufficient sensitivity for early SCLC detection, thereby failing to meet screening requirements. Given the high molecular heterogeneity of SCLC, no single marker can adequately capture the disease's characteristics. Most novel markers suffer from the absence of standardized detection protocols and large‐scale validation, hindering their clinical adoption. There remains a paucity of markers suitable for dynamically monitoring treatment efficacy and predicting recurrence.

Future research should focus on the development of a multi‐omics integrated diagnostic panel that incorporates genomic, transcriptomic, proteomic, and radiomic data to enhance the accuracy of early diagnosis and disease typing. Further efforts are required to advance the application of liquid biopsy technology using ctDNA, exosomes, and CTCs for non‐invasive and dynamic disease monitoring and efficacy evaluation. The development of organoid models and drug susceptibility detection systems is also necessary to guide the precise selection of chemotherapy, targeted therapy, and immunotherapy. Finally, optimizing relevant immunotherapeutic markers by combining TMB, tumor‐infiltrating lymphocytes, and microbiome data will be crucial for establishing a comprehensive model for predicting immune efficacy.

### Pleural mesothelioma

Pleural mesothelioma is a rare and highly aggressive malignancy that originates from pleural mesothelial cells [670]. Its onset has a well‐established causal relationship with asbestos exposure, and the incubation period can extend to several decades. Despite numerous countries imposing restrictions on asbestos use, its incidence is anticipated to remain elevated for some time due to the disease's long latency period (Table S54) [671].

#### Biomarker development process

In terms of era of traditional serum markers, this stage is characterized by mesothelin (SMRP) and its associated soluble fragments, which have become the most widely used diagnostic and monitoring tool in clinical practice [672]. However, these markers can also be elevated in other malignancies such as ovarian and pancreatic cancers, as well as in certain benign pleural lesions. Therefore, a comprehensive evaluation based on clinical manifestations, imaging, and pathological results is required.

In terms of omics integration and clinical translation, the Current research endeavors to construct a precision medicine model, driven by multi‐omics. This model integrates organoid models, spatial omics, and artificial intelligence algorithms with the objective of developing a multi‐marker combination system. This system is intended for use in early diagnosis, prognosis stratification, and treatment guidance.

#### Main types and typical cases of biomarkers

In terms of multi‐omics technology‐driven exploration, the advent of multi‐omics technologies, including proteome, transcriptome, and metabolome, has ushered in an era of systematic screening. This stage is dedicated to the discovery of novel markers with enhanced diagnostic specificity and the elucidation of disease molecular foundations from multiple perspectives.

Among the diagnostic and screening biomarkers, mesothelin, a cell adhesion‐related protein, is highly expressed in pleural mesothelioma and also observed in other malignancies such as pancreatic and ovarian cancers. It is one of the most extensively studied serum markers, suitable for screening and monitoring disease progression in high‐risk populations. Its soluble form, found in serum and pleural effusion, holds dual potential as both a diagnostic tool and therapeutic target [673].

Fibulin‐3 has demonstrated excellent diagnostic performance in early studies, effectively differentiating patients with pleural mesothelioma from healthy asbestos‐exposed individuals, with an area under the curve of up to 0.99. However, several subsequent independent studies have failed to fully replicate its outstanding performance and its diagnostic accuracy remains controversial. Nevertheless, it is still considered to be superior to most plasma markers in terms of overall diagnostic efficacy and has been proposed as a potential intervention target for delaying disease progression [674].

Hyaluronic acid, a polymeric polysaccharide, has been shown to promote various cancers and its concentration is significantly elevated in pleural effusion associated with pleural mesothelioma. Research indicates that its diagnostic efficacy is comparable to that of mesothelin, suggesting that a combined diagnostic model utilizing both could potentially enhance identification accuracy [675].

Among circulating nucleic acids and epigenetic markers, circulating tumor DNA offers the potential for non‐invasive diagnosis and tracking clonal evolution through the detection of tumor‐related gene mutations or methylation alterations. Long non‐coding RNAs, such as *ATG5* and *GAS5*, have also been suggested as prospective diagnostic markers; however, associated research remains in its nascent stages [676, 677].

Among the prognostic and predictive biomarkers, VEGF has been suggested to have some potential in prognostication and disease monitoring [678]. Osteopontin (OPN) showed high diagnostic accuracy for discriminating pleural mesothelioma from healthy individuals, but its specificity was limited, making it difficult to effectively differentiate benign pleural effusion or other pleural diseases. However, several studies consistently demonstrated that elevated osteopontin levels were significantly associated with poor patient outcomes, suggesting that it may be a potential prognostic predictor [678].

Among the image markers and digital markers, CT can show the morphology (nodular or annular) of pleural thickening, its extent, and whether it invades the mediastinum, chest wall, diaphragm, and other structures, and is used for staging and surgical resectability assessment. The metabolic activity of PET‐CT is related to tumor aggressiveness, which helps to differentiate between benign and malignant pleuropathy and guide the biopsy site. MRI is advantageous in assessing chest wall, diaphragm, and spinal invasions [679].

#### Current challenges and future development trends

In terms of current challenges, early diagnosis remains challenged by inadequate sensitivity, making it difficult to reliably identify markers present during the early stages of disease. The heterogeneity inherent in tumor histology results in significant variations in marker expression across different subtypes, thereby impacting detection consistency. Most emerging markers lack standardized testing protocols and large‐scale multicenter validation, which hinders their clinical translation. Furthermore, current markers are not effective in monitoring treatment response, complicating the accurate reflection of the relationship between imaging changes and therapeutic efficacy.

Future research should focus on the development of a multi‐omics integrated diagnostic panel that combines multidimensional data, including proteomic, transcriptomic, and exosome profiles, to establish high‐precision multimodal models. The application of liquid biopsy techniques, such as circulating tumor DNA and exosome analysis, will facilitate early screening and the dynamic monitoring of treatment response. Furthermore, drug susceptibility testing systems that utilize organoid models and proteomic methods are expected to enable the individualized screening of therapeutic strategies, offering a novel approach to improving patient prognosis.

### Dynamic surveillance network for biomarkers of respiratory diseases

**FIGURE 5:**
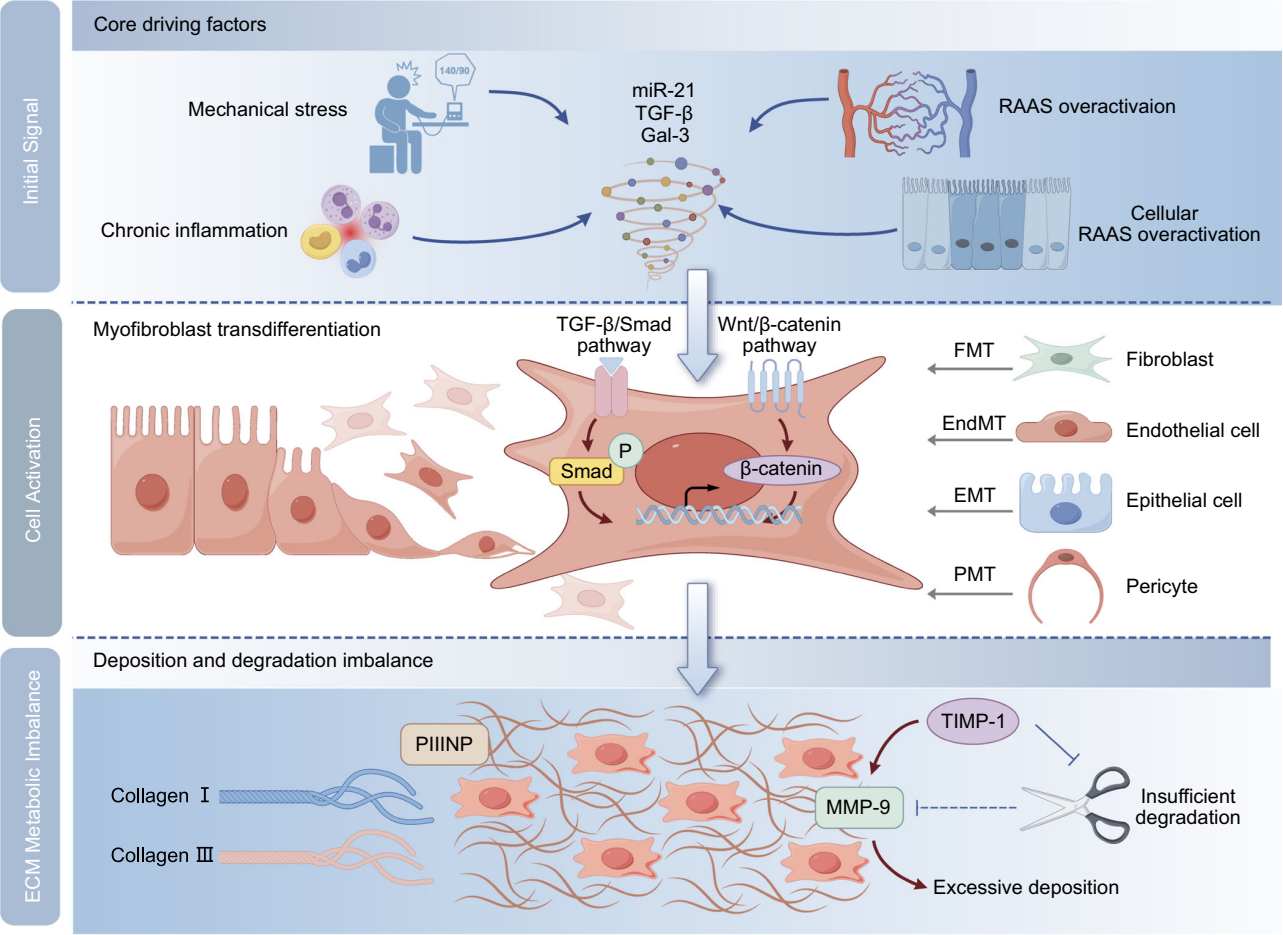
Schematic of tissue fibrosis as a shared terminal pathway of organ failure. This figure summarizes the core mechanisms of tissue fibrosis, a common end‐stage process linking functional decline across multiple organs (e.g., heart, liver, kidney, lung) and systemic autoimmune diseases. The upper panel highlights initiating signals, in which chronic inflammation acts as a central driving force, together with mechanical stress (e.g., hypertension‐associated load), overactivation of the RAAS, and cellular senescence. These upstream cues converge on profibrotic mediators and regulators, including TGF‐β, Gal‐3, and miR‐21, which collectively amplify fibrogenic signaling and microenvironmental remodeling. In the middle panel, the key cellular event is myofibroblast activation and expansion. Activated myofibroblasts arise from multiple sources, including resident fibroblasts as well as transdifferentiation programs such as FMT, EMT, EndMT, and PMT, thereby increasing the pool of ECM‐producing effector cells. Central signaling axes are emphasized, with the TGF‐β/Smad pathway serving as the dominant profibrotic driver, and the Wnt/β‐catenin pathway acting as a complementary pathway that sustains fibroblast activation and matrix gene transcription. The lower panel illustrates ECM metabolic imbalance, where disrupted equilibrium between MMPs and their tissue inhibitors (TIMPs) leads to excessive ECM deposition and insufficient degradation. This imbalance promotes accumulation of collagen and progressive tissue stiffening and scarring.

The respiratory system, a primary interface for gas exchange and material interaction between the human body and its external environment, is continuously exposed to diverse biological, physical, and chemical stimuli. Its biomarker network, comprising the mucosal epithelium, immune cells, and metabolites, exhibits unique spatiotemporal dynamics. Spatially, the biomarker network encompasses local inflammatory factors in the airway, the sputum microbiome, and pathological markers within lung tissue, extending to include peripheral blood, urine, and other indicators in systemic circulation. Temporally, it demonstrates a dynamic evolution that can be phased, reversible, or irreversible, corresponding with disease stage, the ongoing influence of external stimuli, and therapeutic interventions. Accordingly, in this study, major respiratory diseases with high clinical incidence, including COPD, asthma, lung cancer, and lung infection, and the initiation mechanisms and signaling pathways governing local inflammatory responses across different disease states, as well as the systemic effects resulting from the diffusion of inflammatory factors, activation of the immune response, and metabolic disturbances, were systematically investigated. This framework not only integrates a comprehensive biomarker system spanning early warning, progression monitoring, and prognosis assessment but also establishes an organic association between localized pathological changes and the systemic response, thereby providing crucial theoretical support for the accurate classification of respiratory diseases, along with targeted intervention and mechanistic research strategies (Table S55 and Figure 5).

#### Cascades of airway inflammation and oxidative stress markers

FeNO serves as a specific marker for eosinophilic airway inflammation. Its levels are notably elevated in asthma patients and are positively correlated with the severity of airway hyperresponsiveness. This correlation intuitively mirrors the activity of airway eosinophil infiltration, offering a foundational basis for inflammatory classification of the disease. Concurrently, 8‐isoprostaglandins found in exhaled breath condensate act as characteristic products of lipid peroxidation reactions. These compounds serve as crucial indicators for measuring oxidative stress levels in the lungs. Their concentrations significantly increase during the acute exacerbation phase of COPD, accurately indicating the severity of airway oxidative damage and the acute progression of the disease. Notably, neutrophil elastase (NE) and myeloperoxidase (MPO) in sputum collectively represent the primary markers of neutrophilic inflammation. The synergistic fluctuations in their levels can aid in the differentiation of various inflammatory endotypes of asthma, furnishing an essential reference for devising tailored anti‐inflammatory treatment strategies in clinical settings.

#### Markers of lung tissue injury and repair

Alveolar surfactant protein D (SP‐D) and Clara cell protein 16 (CC16) are proteins specifically secreted by alveolar epithelial cells and airway Clara cells, respectively. In the event of lung tissue damage, these proteins can leak from damaged epithelial cells into the peripheral circulation at an early stage of the lesion. This makes them highly sensitive markers that reflect the functional integrity of the lung epithelial barrier. During pulmonary fibrosis, the expression levels of matrix metalloproteinase‐7 (MMP‐7) and periostin are closely and positively correlated with the extent and severity of lung fibrosis. These levels not only accurately predict the progression stage of fibrotic lesions but also effectively predict the disease progression rate in patients with idiopathic pulmonary fibrosis. This provides a reliable basis for determining the timing of clinical interventions and monitoring their efficacy.

#### Evolutionary marker lineage of respiratory tumors

In the context of early screening and precise diagnosis of lung cancer, a combined detection protocol that includes CEA, cytokeratin 19 fragment (CYFRA21‐1), and gastrin‐releasing peptide precursor (ProGRP) can compensate for deficiencies in sensitivity or specificity associated with single markers. This approach effectively differentiates between various pathological types of lung cancer and offers valuable insights for preliminary clinical classification. Notably, CYFRA21‐1 is highly specific for lung squamous cell carcinoma, with elevated levels often correlating closely with the onset and progression of this carcinoma type. ProGRP serves as a highly sensitive marker for small cell lung cancer, showing significant positivity even in the early stages of the disease, thereby serving as a crucial indicator for both screening and differential diagnosis. Concurrently, identifying key driver gene mutations, such as those in *EGFR* and *ALK*, in circulating tumor DNA (ctDNA) enables accurate capture of the molecular characteristics of tumor cells. These findings provide an essential foundation for devising targeted therapy regimens for patients with advanced NSCLC, markedly enhancing treatment relevance and efficacy.

#### Lung infection and immune response marker network

Procalcitonin (PCT) serves as a highly specific marker for bacterial infection, with its expression level fluctuating in correlation with the progression of the infection. In the early differential diagnosis of community‐acquired pneumonia, PCT effectively differentiates between bacterial and viral or noninfectious inflammation. This marker provides valuable scientific guidance for determining the appropriate timing for antibiotic initiation, duration of medication course, and discontinuation indications, thereby holding significant clinical application value. Concurrently, soluble myeloid cell trigger receptor‐1 (sTREM‐1), a key indicator of the local pulmonary inflammatory response, is notably elevated in the alveolar lavage fluid of patients with infectious lung injury. This elevation can directly indicate the activity of bacterial infection within the lungs. In cases of viral pneumonia, the expression level of interferon‐inducible protein‐10 (IP‐10) is closely correlated with disease severity. Its value intuitively reflects the intensity of the antiviral immune response of the body, offering a reliable reference for disease assessment and prognosis.

### The respiratory system serves as the core hub of environment–body interactions

The respiratory system serves as a crucial conduit for interactions between the body and its external environment by not only facilitating gas exchange by supplying oxygen to the entire body and expelling carbon dioxide but also maintaining internal homeostasis. This system employs immune defense mechanisms such as mucosal filtration and ciliary clearance to fend off harmful exogenous substances. Given its dual role in material transport and barrier protection, the respiratory system functions as an environmental sensor in the cross‐system integration of biomarkers. This allows for the conversion of external stimuli into biological indicators and enables the transmission and interaction of these indicators across multiple systems via the circulatory and lymphatic systems. This provides a foundation for disease diagnosis and treatment (Table S56).

#### Hemodynamic coupling of the lung–mandrel axis

Chronic hypoxic lung disease persistently stimulates pulmonary blood vessels, inducing sustained vasoconstriction and promoting cellular proliferation and matrix deposition within vessel walls. These pathological changes culminate in irreversible pulmonary vascular remodeling. Consequently, pulmonary circulatory resistance markedly increases, directly elevating the afterload on the right ventricle. Prolonged overloading of the right ventricle may result in hypertrophy and dilation, potentially progressing to pulmonary heart disease. In response to this stress, cardiomyocytes in the right ventricular wall secrete elevated levels of B‐type natriuretic peptide (BNP) and its N‐terminal precursor (NT‐proBNP), making them significant biomarkers for assessing impaired right ventricular function. Concurrently, chronic hypoxia disrupts the coagulation–fibrinolytic equilibrium of the body, enhancing platelet activation and coagulation factor release and thereby establishing a hypercoagulable state. This condition elevates d‐dimer levels and heightens the risk of pulmonary thromboembolism, exacerbating pulmonary circulation disturbances and right heart failure and perpetuating a detrimental cycle.

#### Two‐way dialog of the lung–gut–microbial axis

The gut microbiota precisely regulates the pulmonary immune response via the gut–lung axis, a cross‐organ regulatory pathway. SCFAs, key metabolites produced by the intestinal microbiota from dietary fiber, can reach the lungs via systemic circulation. They not only inhibit the excessive secretion of proinflammatory factors and reduce inflammatory damage to the airway mucosa but also increase the balance and regulatory capacity of immune cells within lung tissue. Consequently, SCFAs play a significant protective role in mitigating asthmatic airway hyperresponsiveness and delaying the progression of inflammation in patients with COPD. Conversely, during persistent pulmonary inflammation, a large number of inflammatory mediators enter the bloodstream and disseminate to the intestines, disrupting the integrity of the intestinal mucosal barrier and altering the intestinal microecological environment. This leads to a decreased abundance of beneficial bacteria and excessive proliferation of harmful bacteria. Such dysbiosis further impairs the synthesis and secretion of SCFAs, thereby diminishing their inhibitory effect on pulmonary inflammation and ultimately establishing a vicious cycle that exacerbates interconnected lung‐intestinal inflammation. Clinical studies have demonstrated a strong correlation between fecal microbiota diversity and lung disease activity; higher microbial diversity is associated with better control of pulmonary inflammation, providing theoretical support for interventions for lung diseases through modulation of the intestinal flora.

#### Stress regulation of the respiratory–neuro–endocrine axis

Chronic respiratory diseases, such as COPD and interstitial lung disease, induce persistent hypoxia and chronic inflammation that synergistically stimulate and activate the HPA axis. Hypoxia directly influences oxygen‐sensitive neurons in the hypothalamus, while inflammatory factors can cross the blood–brain barrier to alter central regulation. This disruption leads to dysregulation of the rhythm of cortisol secreted by the adrenal cortex, thereby disrupting its normal circadian fluctuation. Consequently, this not only impairs the anti‐inflammatory and stress‐regulating capacities of the body but also may exacerbate immune imbalances and metabolic disorders. In patients with obstructive sleep apnea, nocturnal episodes of intermittent hypoxia frequently trigger stress responses, directly activating the SNS and promoting the release of catecholamines, including adrenaline and norepinephrine, from the adrenal medulla. The sustained elevation of these hormones induces vasoconstriction, tachycardia, and blood pressure fluctuations. In the long term, this significantly increases the risk of cardiovascular complications, including hypertension, arrhythmia, and myocardial infarction, establishing a vicious cycle of “hypoxia–sympathetic activation–cardiovascular injury.”

#### Systemic effects of the environmental–lung–systemic axis

Owing to its small particle size and large specific surface area, atmospheric fine particulate matter (PM_25_) readily adsorbs toxic substances such as heavy metals and polycyclic aromatic hydrocarbons. These particles can directly penetrate the respiratory mucosal barrier, infiltrate deep into the alveoli, and even enter systemic circulation, leading to multitarget damage. Upon entering the lungs, PM_25_ stimulates alveolar macrophages and epithelial cells, prompting the release of numerous reactive oxygen radicals. This action triggers oxidative stress within the lungs and activates inflammatory signaling pathways, notably NF‐κB. Consequently, increased secretion of proinflammatory factors such as TNF‐α and interleukin‐6 (IL‐6) occurs, resulting in sustained local inflammation. The resulting oxidative stress and inflammatory response in the lungs can propagate throughout the body, prompting the liver to synthesize and release CRP. This leads to markedly elevated levels of systemic inflammatory markers, including CRP and IL‐6. These inflammatory factors can impair vascular endothelial function, increase platelet activation and thrombosis, expedite atherosclerosis, and significantly increase the risk of cardiovascular events such as coronary heart disease, myocardial infarction, and stroke. Furthermore, the black carbon content in urine serves as a specific biomarker for assessing long‐term exposure to PM_25_ pollution. As the core component of PM_25_, black carbon is metabolized and excreted in urine upon entering the human body, with its urinary concentration positively correlated with the cumulative exposure dose of an individual. Numerous epidemiological studies have demonstrated that higher urinary black carbon levels are significantly associated with accelerated declines in pulmonary function and increased cardiovascular disease mortality. This association remains independent of confounders such as smoking and age, thereby providing an objective measure for evaluating the long‐term health risks of PM_25_ pollution and identifying high‐risk populations.

#### Cross‐system early warning value of respiratory markers

Forced expiratory volume in the first second (FEV_1_) serves as a pivotal metric in both the diagnosis and classification of COPD. Its value offers a direct reflection of airway obstruction severity. Concurrently, the FEV_1_ is a crucial benchmark for evaluating systemic health risks. Numerous clinical studies have confirmed that reduced FEV_1_ values are significantly positively correlated with increased risks of cardiovascular events, such as hypertension, coronary heart disease, and heart failure, as well as all‐cause mortality. Notably, this association remains robust, irrespective of confounding variables such as age and smoking history, indicating that the FEV_1_ is a dependable prognostic marker for long‐term adverse outcomes. In parallel, the arterial partial pressure of oxygen (PaO_2_), a principal gauge of the oxygenation status of the body, is strongly related to the rate at which cognitive function deteriorates. Prolonged exposure to chronic hypoxia can compromise the oxygen supply to brain tissue, precipitating pathological alterations such as neuronal damage, aberrant synaptic functionality, amplified oxidative stress, and increased expression of inflammatory mediators. These changes incrementally impair memory, attention, and executive capabilities, thus hastening cognitive decline. Such observations underscore the progressive damage caused by chronic hypoxia to the nervous system, furnishing a compelling rationale for early intervention in chronic respiratory ailments and associated cognitive deficits (Figure S6).

## LOCOMOTOR SYSTEM

Literature searches for the locomotor system were performed in PubMed and Web of Science using Boolean logic with field‐restricted search strings. The detailed Boolean queries were constructed for each disease as follows: for osteoporosis, (“osteoporosis”) AND (“biomarker”); for rheumatoid arthritis, (“rheumatoid arthritis”) AND (“biomarker”); for osteoarthritis, (“osteoarthritis” OR “ostarthritis” OR “ostearthritis”) AND (“biomarker”); for osteosarcoma, (“osteosarcoma” OR “osteogenic sarcoma”) AND (“biomarker”); and for multiple myeloma, (“multiple myeloma”) AND (“biomarker”). The retrieval period was defined as January 1, 2020 through December 31, 2025. Only articles published in English were considered. Both original research articles and systematic or narrative reviews were included. Retrieved records were screened by titles and abstracts; articles deemed irrelevant to locomotor system biomarkers, lacking full‐text availability, or containing incomplete data were excluded, and the remaining full texts were thoroughly evaluated. The selected disease spectrum encompasses osteoporosis, rheumatoid arthritis, osteoarthritis, osteosarcoma, and multiple myeloma, all representing major disorders of the locomotor system with high prevalence, substantial disease burden, well‐established biomarker frameworks, and strong clinical translational relevance.

### Osteoporosis

Osteoporosis, a metabolic bone disease characterized by diminished bone mass and microstructural deterioration, is primarily driven by an imbalance between bone resorption and formation [680]. Notably, the 1‐year mortality rate following a hip fracture stands at 20−24% [681]. Given the inconspicuous early symptoms and the limitations of traditional bone density testing in detecting early bone metabolic irregularities, there is an imperative need to develop efficient biomarkers (Table S57).

#### The development process of biomarkers

Among the traditional biomarkers, early studies predominantly depended on serological markers, such as ALP, osteocalcin (OC), type I procollagen N‐terminal propeptide (PINP), and β‐collagen cross‐linked C‐terminal peptide (β‐CTX), in conjunction with imaging indicators like BMD measured via dual‐energy X‐ray absorptiometry [682]. Although these biomarkers established the diagnostic foundation, they presented significant limitations: serum levels were vulnerable to disruptions from liver function and external factors; BMD testing was unable to detect early alterations in bone microstructure; and it had a limited capacity to distinguish between cortical and cancellous bone.

Imaging molecular biomarkers enable quantitative assessment of bone marrow fat content via MRI, thereby refining the evaluation system. Imaging biomarkers for osteoporosis have evolved from single bone density measurements to an integrated quantitative assessment system encompassing bone mass, bone microstructure, bone marrow composition, and bone strength. This advancement provides critical support for secondary prevention and precise fracture risk prediction.

In terms of exploration of molecular markers driven by omics technology, omics technologies have advanced molecular‐level research. Bone metabolism regulators (osteoprotegerin, sclerostin, Dickkopf‐1) and enzyme markers (cysteine protease K, tartrate resistant acid phosphatase) enable precise assessment of osteoblast and osteoclast activity [683]. Noncoding RNAs (miR‐133a, *circRNA_0007059*) and lipid biomarker sphingosine‐1‐phosphate (S1P) provide new dimensions for early diagnosis [684, 685, 686]. Imaging molecular biomarkers quantify bone marrow adiposity by MRI, enriching the evaluation system.

Mendelian randomization studies have further strengthened the causal evidence for certain bone metabolism regulators. For example, genetic analyses have identified sclerostin as a therapeutic target and monitoring biomarker, confirming a causal relationship between sclerostin levels and fracture risk. Additionally, a reverse negative feedback regulatory pathway exists between BMD and sclerostin, providing genetic support for targeting this molecule in osteoporosis management [687].

Current research has transitioned into the integration phase of precision medicine, utilizing AI algorithms and organoid models to holistically analyze disease characteristics through the amalgamation of serum proteins, nucleic acids, lipid biomarkers, and imaging data. Multi‐dimensional evaluation models facilitate a more precise prediction of fracture risk, while patient‐derived bone tissue organoids offer direct evidence for personalized treatment strategies. Additionally, dynamic monitoring of circulating cfDNA can provide early warnings of rapid bone loss risks.

#### The main types of biomarkers and their clinical applications

The combined detection of serum markers can significantly improve the diagnostic efficiency. The combination of PINP and β‐CTX is the core of bone metabolism assessment, and the combination of PINP, β‐CTX and hepcidin can significantly improve the sensitivity of osteoporosis diagnosis [688]. Among circulating nucleic acid markers, miR‐133a may be associated with regulating bone formation, possibly by targeting and inhibiting ATF4, a transcription factor of osteoblasts [686]; *circRNA_0007059* binds to miR‐378 to upregulate BMP‐2 expression and enhance the sensitivity of diagnosis [685]. Imaging markers such as proton density in the vertebral body marrow are negatively correlated with lumbar BMD, which can be used to noninvasively evaluate marrow fat content [689].

Serum biomarkers are pivotal in gauging the efficacy of treatments. Per the International Osteoporosis Foundation's guidelines, a rise of ≥20% in PINP signals the commencement of bone‐forming drug effects. Conversely, a reduction of ≥50% in β‐CTX denotes successful anti‐osteopenic therapy. By integrating osteopontin levels with MRI and serum markers like β‐CTX, one can precisely assess disease progression, leading to a more accurate evaluation of treatment outcomes.

Among the prognosis and predictive biomarkers, the expression levels of genes regulating bone metabolism have significant prognostic value. The elevated level of FGF‐23 indicated abnormal bone mineralization, while the expression of tissue proteinase K and sPD‐L1 was associated with response rates to corresponding inhibitors. A plasma/bone marrow ratio of S1P > 1.2 suggested a high risk of fracture [690]. Organoids derived from patient's bone tissues accurately reproduced the metabolic characteristics of bone, providing an ideal model for drug sensitivity screening. High expression of iron metabolism related gene such as *GSTP1* may be associated with iron accumulation in bone tissues [691], while patients with low expression of *circRNA_0007059* showed higher sensitivity to BMP‐2 treatment [685]. These findings provide new directions for individualized therapy.

#### Current challenges and future trends

Early diagnosis of osteoporosis remains a formidable challenge. The crux of the issue resides in the diagnostic sensitivity, as existing biomarker panels exhibit an accuracy rate below 65% for stage I lesions. Furthermore, the extensive molecular variations observed across different disease subtypes and progression stages pose significant obstacles to biomarker development. Concurrently, the prohibitive costs and absence of standardized protocols for multi‐omics testing impede clinical translation. Additionally, most emerging biomarkers necessitate large‐scale, multicenter validation.

To confront these challenges, the amalgamation of multimodal biomarkers is poised to be a pivotal direction of development. The construction of cross‐dimensional panels, in conjunction with AI algorithms, holds the potential to markedly enhance diagnostic precision. Pioneering innovations such as spatial multi‐omics and single‐cell sequencing are set to unveil interactions within the bone microenvironment and delineate key cell subpopulation characteristics. The establishment of dynamic monitoring systems, grounded in liquid biopsy and coupled with drug sensitivity testing in bone tissue organoids, will pave the way for precise, individualized treatment planning.

### Rheumatoid arthritis

RA is a chronic autoimmune disease with synovial inflammation. It has a global prevalence of about 0.46%. Its typical features are immune‐mediated synovial hyperplasia, pannus formation and progressive osteochondritis [692]. The lack of predictive power for treatment response and the risk of missing seronegative patients have limited clinical application. Therefore, developing a multidimensional biomarker system is crucial (Table S58).

#### The development process of biomarkers

Research into RA biomarkers has transitioned from single‐marker approaches to multi‐omics integration [693]. Initial diagnostic strategies predominantly relied on serological markers, such as rheumatoid factor (RF) and anti‐cyclic citrullinated peptide (anti‐CCP), in conjunction with inflammatory indicators like CRP and ESR [694, 695]. Among them, RF and anti‐CCP were included in the FDA diagnostic criteria in 1981 and 2005, respectively.

However, these conventional markers demonstrated limitations, including insufficient specificity and suboptimal sensitivity for early detection, particularly exhibiting limited diagnostic efficacy in seronegative RA cases.

With the development of proteomics and flow cytometry, research has shifted to explore molecular markers. The discovery of novel autoantibodies such as anti‐mutated citrullinated vimentin antibody (anti‐MCV) and anti‐Peptidylarginine Deiminase 4 (anti‐PD4) antibodies filled the diagnostic gap in seronegative RA [696, 697]. Meanwhile, detection of immune cell markers like plasmacytoid dendritic cells and follicular helper T cell ratio provides new evidence for predicting treatment response [698]. Studies on molecular markers such as microRNA have further elucidated the pathogenesis of RA [699]. The combined analysis of spatial proteomics and single‐cell RNA sequencing can further elucidate cell type enrichment characteristics in various spatial regions, clarifying the localization and functions of T cells, myeloid cells, and specific fibroblast subpopulations in different pathological microenvironments of RA [700]. This strategy elucidates the pathogenesis of RA from a spatial perspective, providing reliable evidence for screening region‐specific biomarkers and identifying precise therapeutic targets.

Current research has reached a stage of multidimensional integration and clinical translation. The electrochemical immunoassay platform facilitates the simultaneous detection of multiple antibodies, while AI algorithms have developed predictive models that significantly enhance diagnostic accuracy. Patient‐derived synovial organoids serve as an ideal platform for biomarker validation and drug sensitivity testing, thereby propelling the progress of personalized medicine.

#### The main types of biomarkers and their clinical applications

Autoantibody markers form the cornerstone of RA diagnosis. Combining RF and anti‐CCP increases the sensitivity of early diagnosis to 82%. Anti‐CCP titers demonstrate a significant correlation with the risk of joint erosion. The detection rates for mutated citrullinated vimentin antibody and anti‐PD4 are higher than those of anti‐CCP. Immunological cell markers, detected by flow cytometry, possess important prognostic value. The proportion of plasmacytoid dendritic cells is closely associated with the response to TNF‐α inhibitors, while follicle T helper cell levels can predict the efficacy of abatacept. These biomarkers provide a basis for the individualized selection of biological agents. Molecular markers offer a new perspective on early diagnosis. For instance, abnormal expression of microRNAs such as miR‐155 can be detected prior to the manifestation of clinical symptoms [701]. Serum amyloid A exhibits greater sensitivity to early inflammation than traditional inflammatory markers, creating opportunities for ultra‐early intervention [702].

Among the disease monitoring biomarkers, dynamic changes in biomarkers during treatment provide an objective basis for evaluating therapeutic efficacy. A decrease in autoantibody titer is significantly correlated with relapse risk, while the sustained normalization of inflammatory markers indicates disease remission. Changes in immune cell subsets can predict therapeutic response at an early stage; for instance, a reduction in the proportion of follicular T helper cells is closely associated with the efficacy of tocilizumab [703]. This multidimensional monitoring strategy significantly improves the accuracy of efficacy evaluation. When multiple criteria are met simultaneously, including decreased antibody titers, alterations in specific immune cell proportions, and normalized inflammatory markers, the long‐term prognosis of patients shows marked improvement. Therefore, Regular multidimensional monitoring is recommended for patients receiving biologic therapy to facilitate timely adjustments to treatment regimens.

Among the prognosis and predictive markers, different biological agents are associated with corresponding predictive biomarkers. For instance, baseline IL‐6 levels influence response rates to tocilizumab [704], guiding precise treatment selection. Biomarkers for predicting bone fracture are significant for evaluating disease progression: anti‑MCV and anti‑PD4 antibodies show strong associations with bone erosion risk, while matrix metalloproteinase‑3 (MMP‑3) levels reflect cartilage degradation [696, 697, 705]. These markers aid in identifying high‑risk patients who may require intensive therapeutic intervention. The integration of organoid models with multi‑omics technologies has established a novel platform for investigating drug‑resistance mechanisms. Patient‑derived synovial organoids can simulate in vivo pathological conditions, enabling validation of biomarker‐drug sensitivity correlations. Furthermore, emerging biomarkers identified via proteomic screening such as heat shock protein 90 (HSP90) offer new therapeutic targets for refractory rheumatoid arthritis [706].

#### Current challenges and future trends

Early diagnosis remains a critical challenge in RA, and the diagnostic sensitivity during the preclinical stage needs to be improved. Due to the significant heterogeneity of the disease, no single biomarker can cover all subtypes of the disease. Therefore, different combinations of biomarkers are needed for different clinical manifestations. The application of these findings in clinical practice also faces many challenges, such as the standardization and popularization of advanced detection methods like flow cytometry. In addition, most new biomarkers lack large‐scale clinical validation data, which limits their practical application.

Future research will focus on the integration of multimodal biomarkers to establish a multidimensional evaluation system that combines autoantibodies, immune cells, molecular markers and imaging features. This approach is expected to significantly improve diagnostic sensitivity and predictive accuracy by leveraging the powerful analytical capabilities of AI algorithms. Technologies such as spatial multi‐omics, microfluidic chips and single cell sequencing are opening new avenues for identifying novel biomarkers and therapeutic targets. A comprehensive management system based on liquid biopsy and organoid drug sensitivity testing enables precise treatment selection and timely adjustments. The development of specific biomarkers for seronegative RA will further refine the diagnostic and classification system for this condition.

### Osteoarthritis

Osteoarthritis, the most common chronic joint disease worldwide, involves an interplay of genetic factors, mechanical stress, and inflammatory response, and clinically manifests as progressive joint pain and functional impairment [707]. Currently, there is a lack of effective early diagnostic and disease‐modifying treatments. The development of robust biomarker systems has become a critical breakthrough for precision medicine in osteoarthritis management (Table S59).

#### The development process of biomarkers

Among the traditional biomarkers, serum CRP and ESR levels, while serving as inflammatory markers, lack specificity, rendering them inadequate for non‐inflammatory conditions. Although joint fluid analysis can provide valuable reference data, it is an invasive procedure. Traditional biomarkers exhibit insufficient overall sensitivity, thereby limiting their effectiveness for early diagnosis.

In terms of exploration of molecular markers driven by omics technology, omics technologies have advanced osteoarthritis research into the molecular domain. Cartilage metabolic markers, such as urinary C‐terminal Telopeptide of Type Ⅱ Collagen (CTX‐Ⅱ) and serum Cartilage Oligomeric Matrix Protein (COMP), serve as key indicators of cartilage degradation. Inflammatory factors, including IL‐6 and TNF‐α, elucidate the mechanisms of synovial inflammation [708], while microRNAs and epigenetic markers offer novel avenues for early diagnosis [709, 710]. This phase signifies a shift from structural evaluation to the investigation of molecular mechanisms.

Current research is centered on the integration of multimodal data and its clinical translation. This involves combining proteomic, metabolomic, imaging, and clinical data to develop predictive models that improve diagnostic accuracy. The use of organoid models and liquid biopsy technology offers a new platform for pharmacodynamic evaluation and personalized treatment, propelling the diagnosis and treatment of osteoarthritis towards precision and dynamic methodologies.

#### The main types of biomarkers and their clinical applications

Multiple biomarkers exhibit significant clinical relevance. In protein biomarker analysis, the combined detection of urinary CTX‐Ⅱ and serum COMP markedly enhances early diagnostic accuracy [711]. Serum hyaluronic acid and MMP‐13 specifically indicate the metabolic balance and degradation activity of the cartilage matrix, respectively [712].

Regarding nucleic acid biomarkers, the downregulation of miRNA‐140‐5p is closely linked to disorders in chondrocyte proliferation and differentiation [713], while the methylation status of the *SOX9* gene serves as a crucial indicator for assessing cartilage tissue repair potential. In the realm of inflammatory markers, IL‐6 and TNF‐α levels in synovial fluid positively correlate with the severity of clinical symptoms such as joint swelling and pain, effectively mirroring the intensity of local inflammatory response and disease activity.

During the monitoring process, a ≥30% decrease in urinary CTX‐Ⅱ levels is indicative of treatment efficacy. Serum HA levels exhibit dynamic changes that reflect the status of cartilage repair, while inflammatory markers such as CRP and IL‐6 levels aid in evaluating the outcomes of anti‐inflammatory treatments. The integration of imaging and molecular approaches, for instance, the use of MRI for cartilage volume measurement combined with CTX‐Ⅱ detection, facilitates a precise assessment of disease progression.

Among the prognosis and predictive biomarkers, genetic markers, specifically *ADAMTS‐5* and *MMP‐13* gene polymorphisms, are significantly associated with the risk of disease progression [714]. Within the immune microenvironment, there is a positive correlation between the infiltration ratio of synovial CD68^+^ macrophages and the severity of joint damage [715]. Levels of sPD‐L1 effectively predict response to immune modulation therapy, while drug sensitivity testing on chondroblasts provides accurate guidance for individualized treatment planning.

#### Current challenges and future trends

Despite the phased progress achieved in relevant research, several challenges remain: inadequate sensitivity for early diagnosis, pronounced disease heterogeneity, significant obstacles to technology translation, and a scarcity of clinical validation data. These factors have emerged as major bottlenecks in the field. Current biomarker panels exhibit limited efficacy in detecting early lesions, and the lack of standardization in multi‐omics testing further impedes its clinical application.

To address these challenges, future research should focus on the following key directions: Developing multimodal biomarker panels by integrating proteomic, nucleomic and imaging features to overcome the limitations of unidimensional detection and improve early diagnosis and prognostic accuracy; Deeply applying cutting‐edge technologies such as spatial multi‐omics and single‐cell sequencing to decipher molecular mechanisms of disease heterogeneity at microscopic levels, thereby establishing liquid biopsy‐based dynamic monitoring systems for real‐time tracking of disease progression and evaluation of treatment efficacy; Developing organoid technology to guide the development of personalized therapeutic strategies.

### Osteosarcoma

Osteosarcoma, a primary malignant bone tumor originating from osteoblast‐derived mesenchymal stem cells. The disease demonstrates significant molecular heterogeneity and complex pathogenesis, leading to substantial variability in prognosis and inconsistent responses to treatment [716]. Most patients remain at risk for recurrence and drug resistance. Consequently, the identification of highly sensitive biomarkers for early diagnosis, risk stratification, therapeutic monitoring, and prediction of treatment resistance represents a critical unmet need (Table S60).

#### The development process of biomarkers

Among the traditional biomarkers, this phase mainly relies on serological and morphological markers. For example, serum ALP and lactate dehydrogenase (LDH) are core indicators for assessing tumor burden and prognosis, which are used in clinical staging and risk stratification. Bone marrow biopsy combined with immunohistochemistry is the gold standard to confirm osteoblastic differentiation of tumor cells. However, traditional biomarkers have obvious limitations. ALP is easily interfered by benign bone diseases, LDH lacks specificity, and bone marrow biopsy is an invasive procedure that makes it difficult to achieve dynamic monitoring.

In terms of exploration of molecular markers driven by omics technology, advances in genomics, transcriptomics, and proteomics have enabled research at the molecular precision level. *TP53* mutation, *RB1* deletion, and other genetic abnormalities have been established as high‐risk prognostic markers and can be detected using techniques such as fluorescence in situ hybridization and next‐generation sequencing; moreover, the discovery of novel molecules, including SAA, circulating non‐coding RNA, and heat shock proteins, offers new avenues for diagnostic and prognostic evaluation [717].

Current research has entered the integration phase of precision medicine, utilizing multi‐omics data and liquid biopsy analysis in conjunction with artificial intelligence and organoid models to comprehensively decipher osteosarcoma's molecular characteristics. Dynamic monitoring of circulating tumor DNA allows for tracking clonal evolution and provides early warnings of drug resistance. Prognostic models developed through multi‐omics integration facilitate more accurate survival outcome predictions. Patient‐derived organoids can mimic primary tumor features, providing direct evidence for personalized treatment strategies.

#### The main types of biomarkers and their clinical applications

Among the diagnosis and screening biomarkers, traditional serum markers ALP and LDH form the basis for prognostic stratification, which can be further refined by combining them with SAA to improve risk stratification accuracy. SAA levels are significantly elevated in osteosarcoma patients, and their dynamic changes closely correlate with treatment responses [718]. Emerging biomarkers such as *TIM‐3* and *WNT6* mRNA address limitations of conventional markers, *WNT6* may have potential as a marker for differentiating osteosarcoma from benign bone lesions (AUC 0.854) [719]. Ultra‐deep sequencing enables detection of mutations in *TP53*, *RB1*, and other genes within circulating tumor DNA, with mutation‐positive patients exhibiting a significantly increased risk of recurrence. Among non‐coding RNAs, miR‐124 is significantly downregulated in the serum of osteosarcoma patients, yielding a diagnostic AUC of 0.846 [720]; circRNAs such as *hsa_circ_0005721* show sustained upregulation in patient blood, effectively distinguishing healthy individuals from those with benign bone tumors [721]. Overexpression of the heat shock protein HSP90 indicates chemotherapy resistance and poor prognosis [722], and its inhibitor is currently in preclinical development.

Among the disease surveillance biomarker, the most frequently used efficacy monitoring tools are ALP, LDH and SAA. The normalization of ALP and LDH and a decrease in SAA by ≥60% after treatment indicated the effectiveness of the treatment and extended progression‐free survival. Survivin‐positive CTCs served as an independent prognostic marker [723]. MRD is an important indicator for evaluating therapeutic efficacy [724]. Exosomal encapsulated ncRNAs emerged as novel biomarkers, especially high expression of *hsa_circ_103801* [725], which was strongly associated with cisplatin resistance. Metabolic markers such as abnormal glycine and proline metabolism showed associations with tumor proliferation activity, while posttreatment normalization indicated suppressed tumor growth.

Among the prognosis and predictive biomarkers, genetic aberrations such as *TP53* mutation, *RB1* deletion and *NOTCH1* mutation are classified as high‐risk variants with a 5‐year overall survival rate of less than 40% in patients carrying any of these aberrations [726]. *MDM2* amplification is associated with favorable prognosis in low grade osteosarcoma [727]. gene‐expression‐profile‐based prognostic signature can accurately identify the high risk population and guide the duration of maintenance therapy.

Abnormal expression of proteins and immune markers can also indicate disease status. EPHA2 is highly expressed on the surface of osteosarcoma cells, and patients who are positive for this protein have significantly reduced survival rates [728]. The immune checkpoint molecules PD‐L1 and TIM‐3 mediate immunosuppression, where elevated serum levels of TIM‐3 correlate with poor prognosis [729]. Patient‐derived organoids of osteosarcoma faithfully recapitulate primary tumor characteristics, providing an ideal model for drug sensitivity screening. Dynamic monitoring of ctDNA allows tracking of clonal evolution and early detection of resistant mutations [730].

#### Current challenges and future trends

In the realm of early diagnosis, existing biomarker panels exhibit a diagnostic sensitivity that falls below 65% for stage I osteosarcoma, thereby rendering ultra‐early screening impractical. The inherent heterogeneity of tumors precludes the use of single markers to accurately represent disease status. Furthermore, technological challenges arise from the prohibitive costs and absence of standardized protocols associated with multi‐omics testing, which in turn obstruct its integration into primary care settings. It is also noteworthy that the majority of these novel biomarkers have been validated within the confines of small‐scale studies, thus lacking robust support from large‐scale clinical data.

To address these challenges, future research should focus on overcoming development bottlenecks by prioritizing multidimensional technological integration and precise alignment with clinical needs. This includes the development of a comprehensive biomarker panel that integrates serum proteins, circulating nucleic acids, minimal residual disease status, and imaging characteristics. Utilizing AI algorithms for cross‐dimensional data analysis can surpass the limitations of single biomarkers or testing methods, thereby significantly improving early diagnostic sensitivity and disease prognosis accuracy. Additionally, the extensive application of spatial multi‐omics and single‐cell sequencing technologies will facilitate targeted therapy upgrades at the microscopic level. Based on this foundation, the establishment of a full‐cycle dynamic monitoring system, combined with organoid drug sensitivity testing for clinical translation, will enable personalized treatment plans tailored to individual patients.

### Multiple myeloma

Multiple myeloma is a malignant hematologic tumor characterized by clonal proliferation of malignant plasma cells in the bone marrow and associated organ dysfunction [731]. The early symptoms of this disease are nonspecific and highly heterogeneous, with many patients being diagnosed only when extensive bone injuries or renal insufficiency occur. Therefore, developing highly sensitive biomarkers is a critical challenge for precision medicine in multiple myeloma (Table S61).

#### The development process of biomarkers

Among the traditional biomarkers, the staging system is based on serum β2‐microglobulin and albumin, which are used to evaluate tumor burden and prognosis [732], and the ratio of serum free light chain (FLC), which is used for diagnosis and monitoring [733]. The proportion of clonal plasma cells in bone marrow ≥10% is the gold standard for diagnosis, and the clonal characteristics can be further confirmed by *CD138* immunohistochemistry [734]. However, there are obvious limitations to these markers: β2‐microglobulin is easily affected by renal function, albumin is affected by nutritional status, and bone marrow biopsy is an invasive procedure that makes dynamic monitoring difficult.

With the development of genomics, transcriptomics and proteomics, research has entered the molecular level. Cytogenetic abnormalities such as del(17p) and t(4;14) have been established as high‐risk prognostic markers [735]. The expression of GEP70 and SKY92 can be used to achieve accurate risk stratification [736]. In 2024, the FDA officially recognized MRD as a key indicator for evaluating the therapeutic efficacy of multiple myeloma (MM) treatment. In addition, the discovery of new biomarkers such as serum BCMA, metabolomics markers, and non‐coding RNA provides a new dimension for the diagnosis and evaluation of prognosis in MM.

Current research has entered the integrated phase of precision medicine, using integrated genomics, transcriptomics, proteomics and liquid biopsy data combined with artificial intelligence and organoid models to comprehensively analyze the molecular characteristics of MM. The dynamic monitoring of ctDNA can track clonal evolution and provide early warnings of drug resistance, while multi‐omics integrated prognostic models can more accurately predict survival outcomes. Organoid models provide an ideal platform for drug sensitivity testing and drug resistance mechanism studies.

#### The main types of biomarkers and their clinical applications

Regarding serum markers, the combination of β2‐microglobulin and albumin is the basis for ISS staging, and the addition of LDH can further improve the accuracy of stratification. The ratio of serum free light chain combined with immunofixation electrophoresis can significantly improve the diagnostic rate of nonsecretory MM. Serum BCMA level was significantly increased in MM patients, and its diagnostic sensitivity reached 75%, which could effectively distinguish monoclonal gammopathy of undetermined significance (MGUS) from early stage MM [737]. Among circulating nucleic acids and epigenetic markers, ctDNA enables noninvasive monitoring of gene mutations such as *TP53* and *KRAS* [738]. Its detection sensitivity is consistent with that of MRD in bone marrow, and it can more accurately predict the risk of recurrence. The expression levels of noncoding RNAs such as miR‐203 were downregulated in MM patients, so they might be used as potential biomarkers [739]. Low miR‑30d expression may be associated with poor prognosis [740]. The methylation status of *RASSF1A* and other genes was related to disease progression and drug resistance, and the progression‐free survival of methylated positive patients was significantly shortened. Free DNA methylation profiles such as *RASSF1A* can serve as noninvasive biomarkers, providing a new perspective for multiomic diagnosis in clinical practice of MM [741].

Among the disease surveillance biomarker, the serological dynamic index serves as the primary tool for monitoring treatment efficacy [742]. A partial response is indicated by either normalization or a reduction of at least 50% in the serum free light chain ratio, coupled with a minimum 50% decrease in M protein levels. Full remission is confirmed through complete normalization of both biomarkers. Therapeutic effectiveness is demonstrated by ≥60% reduction in serum BCMA concentrations, which correlates with extended progression‐free survival durations. Lactate dehydrogenase (LDH) levels reflect tumor metabolic activity, where post‐treatment normalization suggests inhibited proliferative activity. Elevated ALP concentrations may indicate improvement in osseous lesions.

Among the prognosis and predictive biomarkers, cytogenetic abnormalities serve as the cornerstone of prognostic stratification. Del(17p) and t(4;14) are high‐risk abnormalities, whereas t(11;14) indicates sensitivity to BCL‐2 inhibitors [743]. Gene expression profiles such as GEP70 gene signature can identify high‐risk patients [736]. The number of circulating tumor cells and survivin‑positive circulating tumor cells have been established as prognostic markers in other malignancies; however, their significance in multiple myeloma requires further validation.

#### Current challenges and future trends

Early diagnosis remains a bottleneck, with the sensitivity of current biomarker combinations being less than 65% for stage I MM diagnosis. However, multi‐omics testing is expensive and complex, making it difficult to implement in primary hospitals. Liquid biopsy has limited sensitivity in patients with low tumor burden. Most novel biomarkers lack large‐scale clinical validation, preventing their inclusion in clinical guidelines.

In the future, research should focus on integrating multimodal biomarkers to develop a comprehensive diagnostic panel that combines serum proteins, circulating nucleic acids, minimal residual lesions, and imaging features. This panel will be enhanced by AI algorithms to improve diagnostic accuracy. Spatial multi‐omics technologies will elucidate tumor‐microenvironment interaction patterns, while single‐cell sequencing will decode tumor stem cell characteristics. Microfluidic chips will refine exosome detection. We will establish a dynamic monitoring system based on liquid biopsy, which will be combined with organoid drug sensitivity testing to guide personalized treatment.

MM exhibits significant ethnic variations, directly leading to marked deviations in biomarker expression, distribution, and prognostic evaluation criteria across different populations, which substantially impacts precision diagnosis and risk stratification. Compared to white individuals, black individuals exhibit a significantly higher incidence rate of MM and show characteristic differences in key molecular markers. At the cytogenetic level, black patients exhibit a higher incidence of t(11;14) translocation, while lower proportions of high‐risk markers such as t(4;14),17p deletion, and *TP53* mutations are observed. In addition, there are ethnic differences in immunoglobulin subtype distribution, with a lower proportion of IgM subtype in black patients with MGUS, suggesting that the interpretation of conventional markers such as serum M protein and FLC ratio requires correction for ethnic background [744]. This race‐driven biomarker heterogeneity highlights the limitations of a single universal evaluation criterion, underscoring the necessity of establishing race‐specific reference ranges and risk models to improve diagnostic accuracy.

### Dynamic monitoring network for biomarkers of musculoskeletal disorders

The musculoskeletal system, which serves as the primary mechanical support structure and facilitates movement within the human body, exhibits unique biomechanical and metabolic properties. This study presents a comprehensive framework that encompasses bone metabolism through muscle function, achieved by synthesizing analyses of major musculoskeletal disorders such as osteoporosis, arthritis, sarcopenia, and sports injuries (Table S62).

#### Biphasic regulatory network of bone metabolic balance biomarkers

The equilibrium of bone metabolism is upheld by both bone formation and resorption markers, with their coordinated changes indicating shifts in the state of bone turnover. Among the bone formation markers, bone‐specific alkaline phosphatase (BALP) is synthesized predominantly by osteoblasts; it plays a crucial role in bone mineralization by catalyzing phosphoester hydrolysis, serving as a specific indicator of osteoblast activity. In some individuals with osteoporosis, BALP levels may be lower than those in normal controls but can rebound following anti‐osteoporosis treatment, establishing BALP level as a vital metric for gauging therapeutic effectiveness [745]. The precursor fragment known as type I procollagen N‐terminal propeptide (PINP) is released during the synthesis of type I collagen, directly mirroring the rate of bone collagen synthesis. Serum concentrations of PINP remain relatively consistent with minimal diurnal variations, making it more appropriate than other bone formation markers for extended evaluations of bone turnover status and for tracking the efficacy of anti‐osteoclastic treatments [746]. Conversely, bone resorption markers such as type I collagen C‐terminal cross‐linking end peptide (β‐CTX) and anti‐tartrate‑resistant acid phosphatase 5b (TRAP5b) primarily indicate osteoclast activity; elevated levels often indicate rapid bone loss [747, 748]. Notably, osteocalcin (OC) is not only a bone formation marker but also has endocrine functions. OC modulates insulin secretion through its binding to receptors on pancreatic β‐cells and concurrently promotes lipolysis and energy metabolism in adipocytes, thus intricately intertwining bone metabolism with systemic glucose metabolism [749].

#### Series of markers for articular cartilage metabolism and degradation

In joint diseases such as osteoarthritis and rheumatoid arthritis, cartilage metabolic markers exhibit characteristic abnormal changes. The carboxy‐terminal propeptide of type II collagen (PIINP) reflects the synthesis activity and repair capacity of cartilage: In the early stages of osteoarthritis, when cartilage tissue initiates repair mechanisms in response to injury, PIINP levels are often significantly elevated, whereas in the late stages of the disease, PIINP levels gradually decrease as chondrocyte function declines [750]. Urinary C‐telopeptide of type II collagen (CTX‐II) is a specific biomarker for cartilage degradation. Elevated levels of this peptide indicate cartilage destruction and can be used to assess the extent of joint damage and predict outcomes [751]. Moreover, serum levels of COMP, a component of the extracellular matrix of chondrocytes, are positively correlated with the degree of articular cartilage destruction, making it suitable for evaluating joint load and monitoring sports injuries at an early stage [752]. Furthermore, in inflammatory joint diseases, markedly elevated levels of MMP‐3, which is produced primarily by synovial fibroblasts and inflammatory cells, serve as critical biomarkers for assessing synovitis activity and predicting joint destruction risk [753].

#### Combination of biomarkers for muscle mass and function assessment

In the evaluation of muscle‐related diseases such as sarcopenia, multidimensional biomarkers are often needed. The serum creatinine production rate is positively correlated with muscle mass and can indirectly indicate total muscle volume, but its specificity is limited and susceptible to interference from factors such as renal function and dietary meat intake [754]. In contrast, growth differentiation factor‐8 (GDF‐8) levels are negatively correlated with muscle mass and serve as a characteristic marker of impaired muscle regeneration [755]. In muscular dystrophy and other diseases, the disruption of muscle cell membrane integrity leads to the release of intracellular enzymes, resulting in significantly elevated levels of creatine kinase (CK) and aldolase (ALD). As a sensitive biomarker for muscle cell injury, the magnitude of CK elevation is positively correlated with the degree of injury. ALD, as a key glycolytic enzyme, is localized primarily in the cytoplasm of muscle cells. ALD levels increase significantly during the inflammatory phase of muscle activity and gradually decrease after effective treatment [756]. Recent studies have also shown that serum myoglobin (Mb) levels increase rapidly in the early stages of muscle cell injury, demonstrating high sensitivity and thus being useful for the early diagnosis of acute muscle injury [757]. Additionally, as an exercise‐induced myokine, irisin not only promotes the browning of white adipose tissue and increases energy expenditure but also improves insulin sensitivity through the AMPK pathway, thereby directly linking muscle exercise to systemic metabolic health; it has become an important biomarker for assessing the benefits of exercise and muscle functional status [758].

#### Tumors and injury repair markers in the musculoskeletal system

In the diagnosis and treatment of bone tumors, ALP levels are often significantly elevated in patients with osteosarcoma, correlating with the proliferative activity of tumor cells and their capacity for bone formation [759]. Consequently, ALP can be utilized to monitor disease progression and evaluate therapeutic efficacy. The N‐terminal cross‐linking peptide (NTX) of type I collagen found in urine results from the degradation of type I collagen during bone resorption. In patients with bone metastases, NTX levels are typically markedly elevated because of increased osteoclast activity induced by tumor cells, making it a biomarker for bone metastasis [760]. With respect to sports injuries, serum concentrations of collagen degradation products, such as C1M and C3M, are positively correlated with injury severity, indicating the degree of connective tissue damage [761]. Concurrently, alterations in inflammatory markers such as IL‐6 and TNF‐α are intimately linked to the injury repair process and serve as indicators to gauge the progression of recovery [762]. Furthermore, the expression of MMP‐9, which is a member of the MMP family, is frequently abnormally elevated following sports injury, potentially indicating the occurrence of aberrant tissue repair.

### The musculoskeletal system as an integrated hub of mechanics–metabolism–endocrinology

The musculoskeletal system interacts with other bodily systems in a multidimensional network through mechanical stimulation, metabolic regulation, and endocrine functions. It acts as a mechanical sensor and signal transduction hub, integrating biomarkers across different systems. By secreting bioactive molecules such as actin and osteocalcin, the musculoskeletal system helps regulate systemic energy metabolism, immune function, neural modulation, and endocrine balance. Moreover, the functional states of various systemic components influence the structure and function of the musculoskeletal system through feedback signals, creating a dynamic, bidirectional regulatory loop (Table S63).

#### Energy metabolism coupling of the bone–muscle–fat axis

As a primary organ for glucose metabolism in the human body, skeletal muscle plays a crucial role in maintaining systemic glucose homeostasis by consuming significant amounts of glucose during contraction. Exercise can stimulate the secretion of irisin, which not only facilitates the browning of white adipose tissue and increases energy expenditure but also regulates bone metabolism and muscle growth [763]. Concurrently, adiponectin and leptin secreted by adipose tissue provide feedback regulation to bone metabolism, collectively forming an energy balance network within the bone–muscle–fat axis. Adiponectin promotes osteoblast proliferation and bone formation while suppressing osteoclast activity [764]; leptin contributes to the regulation of bone metabolism via the central nervous system and promotes muscle protein synthesis [765]. In patients with sarcopenic obesity, characterized by low muscle mass and increased visceral fat accumulation, there is a reduction in irisin secretion and decreased levels of adiponectin and resistance to leptin. This combination significantly increases the risk of metabolic syndrome, cardiovascular diseases, and osteoporosis [764].

#### Mechanobiological transduction of systemic effects

The skeleton, as the body's primary mechano‐sensor, can sense mechanical stress and convert it into biochemical signals to regulate local bone metabolism and systemic physiological functions. This process is completed mainly through mechanosensitive ion channels and integrin signaling pathways. Mechanical stress activates mechanosensitive receptors on the surface of osteoblasts, triggering intracellular Ca^2+^ signal transduction, thereby promoting osteoblast proliferation and differentiation and stimulating the secretion of factors such as osteocalcin and bone bridging protein. Osteocalcin and osteopontin produced by osteoblasts not only participate in the regulation of local bone metabolism but also influence distal organ function through the circulatory system: osteocalcin regulates insulin secretion and glucose metabolism, whereas osteopontin is involved in processes such as immunomodulation and vascular remodeling [766]. Insufficient mechanical stimulation environments, such as bed rest or space microgravity, inhibit osteoblast activity while activating osteoclasts, leading to bone loss and muscle atrophy, further confirming the systemic regulatory role of mechanical stimulation on the musculoskeletal system and systemic metabolism [767, 768].

#### Inflammatory regulatory network of the motor–immune axis

Moderate exercise can establish a complex inflammatory regulatory network, that is, the exercise–immune axis, by regulating myokine secretion [769, 770]. During exercise, skeletal muscle secretes anti‐inflammatory myokines such as IL‐6, IL‐15 and IL‐10. These factors not only exert anti‐inflammatory effects but also participate in muscle repair and energy metabolism regulation, thereby enhancing immune function [771]. In contrast, chronic overtraining suppresses immune function and increases susceptibility to infection [772]. In autoimmune diseases such as rheumatoid arthritis and ankylosing spondylitis, regular exercise helps reduce joint inflammation and pain while improving joint function. This mechanism involves exercise‐induced anti‐inflammatory action factor secretion and the inhibition of abnormal immune cell activation, demonstrating the bidirectional role of the exercise–immune axis in regulating inflammation and immune function.

#### Synergistic regulatory mechanism of the neuromuscular axis

The neuromuscular axis regulates muscle contraction, growth and neural function through bidirectional crosstalk between motor neurons and muscle tissue. Motor neurons control the contraction of muscle cells by releasing neurotransmitters via neuromuscular junctions. In turn, muscle tissue secretes BDNF and glial cell line‐derived neurotrophic factor (GDNF), which promote neuronal survival, differentiation and synaptic plasticity to maintain the functional integrity of neuromuscular junctions [773, 774]. In diseases such as ALS, degenerative changes in motor neurons lead to neuromuscular junction dysfunction and subsequent muscle atrophy; furthermore, serum neurofilament light chain (NfL), a specific biomarker of neuronal axonal injury, is elevated, reflecting the degree of axonal damage, and is closely related to the disease progression rate and prognosis [775].

#### The value of cross‐system early warning of motor system markers

Biomarkers of the musculoskeletal system not only reflect its functional status but also serve as significant early warning indicators across different systems. For example, BMD is a key diagnostic indicator for osteoporosis and has been identified as an independent predictor of cardiovascular death and all‐cause mortality [776]. Similarly, grip strength, a simple measure of muscle function, is closely linked to cognitive decline and disability risk, making it a valuable tool for assessing the overall health of the elderly population [777]. The levels of certain traditional cardiovascular markers, such as serum troponin and BNP, can increase following excessive exercise, suggesting potential exercise‐induced myocardial injury [778]. Conversely, renal function markers such as urinary protein and creatinine are associated with exercise intensity, and vigorous exercise can potentially trigger rhabdomyolysis, which may lead to AKI [779]. These findings underscore the crucial role of musculoskeletal biomarkers in systemic health assessment, offering new perspectives and strategies for the early detection and comprehensive treatment of multisystemic diseases (Figure S7).

## REPRODUCTIVE SYSTEM

Literature searches for the reproductive system were performed in PubMed and Web of Science using Boolean logic with field‐restricted search strings. For male reproductive diseases, the following Boolean queries were constructed: for benign prostatic hyperplasia, (“prostatic hyperplasia” OR (prostat* AND (hyperplas* OR hypertroph* OR enlarg* OR adenoma*)) OR “BPH”) AND (“biomarker”) AND (“disease”); for prostate cancer, (“prostatic neoplasms” OR “prostate neoplasm” OR “prostatic neoplasm” OR “cancer of the prostate” OR “prostatic cancer” OR “prostate cancer” OR “prostate gland cancer” OR “prostate tumor” OR “prostatic tumor”) AND (“biomarker”) AND (“disease”); and for testicular cancer, (“testicular neoplasms” OR “testicular neoplasm” OR “testis neoplasm” OR “cancer of the testis” OR “testicular cancer” OR “testis cancer” OR “testicular tumor” OR “testicular carcinoma”) AND (“biomarker”) AND (“disease”). For female reproductive diseases, the search strategies were as follows: for polycystic ovary syndrome, (“polycystic ovarian syndrome” OR “PCOS” OR “polycystic ovary syndrome” OR “Polycystic Ovary Syndrome”) AND (“biomarker”) AND (“disease”); for cervical cancer, (“cervical cancer” OR “cervical carcinoma” OR “Uterine Cervical Neoplasms”) AND (“biomarker”) AND (“disease”); for ovarian cancer, (“ovarian cancer” OR “oophoroma” OR “Ovary cancer” OR “Ovarian Neoplasms”) AND (“biomarker”) AND (“disease”); for breast cancer, (“breast cancer” OR “breast carcinoma” OR “breast tumor” OR “Breast Neoplasms”) AND (“biomarker”) AND (“disease”); for endometrial cancer, (“endometrial cancer” OR “endometrial adenocarcinoma” OR “endometrial carcinoma” OR “Endometrial Neoplasms”) AND (“biomarker”) AND (“disease”); and for endometriosis, (“endometriosis” OR “Endometriosis”) AND (“biomarker”) AND (“disease”). The retrieval period was defined as January 1, 2020 through December 31, 2025. Only articles published in English were considered. Both original research articles and systematic or narrative reviews were included. Retrieved records were screened by titles and abstracts; articles deemed irrelevant to reproductive system biomarkers, lacking full‐text availability, or containing incomplete data were excluded, and the remaining full texts were thoroughly evaluated. The selected disease spectrum encompasses polycystic ovary syndrome, endometriosis, benign prostatic hyperplasia (BPH), ovarian cancer, cervical cancer, endometrial cancer, breast cancer, prostate cancer, and testicular germ cell tumors, all representing major reproductive disorders with high prevalence, substantial disease burden, well‐established biomarker frameworks, and strong clinical translational relevance.

### Polycystic ovary syndrome

Polycystic ovary syndrome (PCOS) is the most common endocrine and metabolic disorder in women of reproductive age, characterized by ovulatory dysfunction, hyperandrogenism, and polycystic ovarian morphology [780]. Developing reliable biomarkers is essential to facilitate early diagnosis, phenotype stratification, and targeted therapeutic interventions (Table S64).

#### The development process of biomarkers

Early studies mainly relied on hormonal markers such as testosterone, luteinizing hormone to follicle‐stimulating hormone (LH/FSH) ratio and anti‐Müllerian hormone (AMH) [781]. Although these markers constitute the basis of clinical diagnosis, their sensitivity is limited in patients with early or atypical presentations and they are susceptible to confounding factors such as menstrual cycle phase, age and body weight. These limitations reduce their effectiveness as standalone diagnostic indicators [782]. With the development of genomics, metabolomics and proteomics, an increasing number of novel biomarkers have been identified. Mendelian randomization studies have shown that circulating microRNAs, metabolites, and inflammatory factors are closely related to the pathogenesis of PCOS [783], advancing biomarker research into the era of systematic screening. The current study focuses on establishing a multi‐omics‐driven precision classification system. By integrating genomic, metabolomic, proteomic and clinical data, supplemented by artificial intelligence algorithms, it aims to develop a comprehensive biomarker panel for early identification, risk assessment of complications and prediction of treatment response [784].

#### Main types and typical cases of biomarkers

In the clinical diagnosis and exploration of pathological mechanisms of PCOS, multiple biomarkers have revealed the complexity of this disease from different perspectives. The hormonal biomarker is one of the core elements for diagnosis, among which AMH is a reliable indicator reflecting ovarian reserve and antral follicle count, and it increases significantly in patients with PCOS. However, there may be substantial differences among various racial groups. A study reveals that the AMH threshold for Caucasians is 4.7–5.0 ng/mL, whereas for East Asian populations (such as Japan and Korea) it is higher, reaching 10 ng/mL [785] **.** In order to improve the accuracy of differential diagnosis, studies are focusing on integrating AMH with testosterone, insulin resistance index and other indicators to construct diagnostic models to effectively distinguish PCOS from premature ovarian insufficiency, functional hypothalamic amenorrhea and other diseases [786]. Inflammatory biomarkers reveal the chronic low‐grade inflammation state often associated with PCOS. Classic inflammatory factors such as CRP, IL‐6 and TNF‐α increase continuously [784], while MCP‐1, MIP‐1α and IL‐18 also increased significantly. These factors not only help assess the severity of the disease but are also closely related to the risk prediction of metabolic and other comorbidities. In addition, proteomics and new molecular biomarker research provide a deeper perspective. Sex hormone‐binding globulin, as a key transport protein, its decreased plasma level is closely related to hyperandrogenism in PCOS patients [787]. Meta‐analysis also confirmed that the decrease in its concentration was significantly correlated with an increased risk of PCOS. In the field of non‐coding RNA, abnormal expression of miR‐18b and miR‐146a in follicular fluid affects steroid hormone synthesis; meanwhile, long non‐coding RNA Taurine Upregulated Gene 1 (*TUG1*) is significantly upregulated and shows great potential as a novel diagnostic biomarker [783]. Pelvic ultrasound is a core imaging detection method in clinical practice. Polycystic‐like morphology of the ovaries is a classic imaging marker. The quantitative diagnostic criteria are that the number of follicles with a diameter of 2–9 mm in one or both ovaries is ≥12, or the ovarian volume is ≥10 mL. This is an important basis for the clinical diagnosis of PCOS. Meanwhile, the key digital markers based on ultrasound, such as the count of ovarian follicles and the quantified value of ovarian volume, combined with the detection indicators of sex hormones, can be used to construct a multi‐dimensional digital scoring model. This model can accurately distinguish PCOS from other ovulatory disorders and also provide a quantitative basis for stratifying the severity of the disease.

In the systematic study of PCOS, metabolomics and gut microbiomics [788, 789] have provided new insights into its long‐term complications and pathological mechanisms. Metabolomic markers can identify individuals at an early stage who are at increased risk for long‐term metabolic complications. Studies have found that kisspeptin is significantly elevated in the serum of patients with PCOS and positively correlated with LH and testosterone levels, suggesting that it may serve as a dual‐function marker for both diagnosis and metabolic assessment [790]. Meanwhile, glutamine transporter SLC1A5 has been identified to play a key role in PCOS [791], and pharmacological blockade of this protein showed reproductive benefits in PCOS‐like mouse models, indicating its potential as a therapeutic target. On the other hand, studies on gut microbial markers have revealed the important role of gut microbiota in the pathogenesis of PCOS, with *Bacteroides* being significantly enriched in the patient's gut and regarded as a key microbial marker. Antibiotic treatment could improve the PCOS phenotype and prevent insulin resistance by activating ileal FXR, and further treatment with FXR agonist chenodeoxycholic acid also successfully improved glucose metabolism in PCOS mice, suggesting that targeting the *Bacteroides*‐FXR axis might become a novel potential therapeutic strategy [792].

#### Current challenges and future trends

Currently, the research and application of biomarkers for PCOS still face multiple challenges [793]. Firstly, most biomarkers such as CRP and insulin have insufficient specificity. They are not unique to PCOS but also commonly found in obesity and metabolic syndrome, thus being susceptible to interference during differential diagnosis. Additionally, PCOS exhibits significant phenotypic heterogeneity, encompassing various molecular subtypes such as reproductive and metabolic types. This makes it challenging for a single biomarker to comprehensively reflect the complex pathological background of the disease. Notably, although multi‐omics technologies offer potential for systemic analysis of PCOS, their high detection costs and standardization difficulties present significant barriers to technological transformation, severely limiting their clinical application and promotion.

To address the aforementioned challenges, PCOS research is trending toward multi‐modal integration and high‐tech innovation. On one hand, by integrating multi‐omics data such as genomics, metabolomics, proteomics, and microbiomics, combined with AI algorithms to optimize marker combinations, it is possible to construct diagnostic models that transcend single indicators, thereby significantly enhancing disease diagnosis and prediction efficacy [784]. On the other hand, in‐depth applications of high technologies provide strong support for this goal. Spatial transcriptomics can be used to characterize cell–molecule interaction mechanisms in the ovarian microenvironment at high spatial resolution, while microbiomics can be employed to explore specific bacterial community characteristics in areas such as the gut. Ultimately, all these efforts are aimed at clinical translation. By establishing standardized detection systems and promoting efficient and low‐cost detection platforms, these research achievements will translate into individualized treatment plans that guide clinical practice.

### Endometriosis

Endometriosis (EMT) is a chronic gynecological disease characterized by the growth of endometrial tissue outside the uterine cavity. It is often accompanied by pain, infertility and decreased quality of life. Due to the lack of specificity of its symptoms, clinical diagnosis is generally delayed [794]. Therefore, the development of reliable biomarkers for early identification, classification and treatment guidance is of great clinical significance (Table S65).

#### The development process of biomarkers

Early biomarker efforts included Cancer Antigen 125 (CA‐125), particularly for ovarian endometriotic cysts, but sensitivity is low in early‐stage or peritoneal lesions. Advances in genomics, proteomics, and metabolomics have introduced circulating microRNAs, inflammatory cytokines, and epigenetic markers such as DNA methylation. Current efforts focus on integrating multi‐omics data with AI and organoid models to support early screening and disease stratification.

#### Main types and typical cases of biomarkers

In terms of diagnosis and screening of biomarkers, biomarkers such as CA‐125 have served as auxiliary diagnostic tools since the 1980s, particularly in patients with ovarian endometriotic cysts, where elevated levels are often observed. However, their sensitivity remains relatively low in early‐stage or peritoneal lesions (approximately 30−50%), and false‐positive results may occur in conditions such as pelvic inflammatory disease or pregnancy, limiting their utility as a standalone screening tool [795]. Advancements in genomics, proteomics, and metabolomics have ushered research into a new phase. Circulating microRNAs (miR‐199a and miR‐122) [796], inflammatory cytokines (IL‐6 and IL‐8) [797], and epigenetic markers such as DNA methylation offer promising avenues for non‐invasive diagnosis. Current efforts are focused on integrating transcriptomic, epigenomic, proteomic, and metabolomic data, combined with artificial intelligence and organoid models, to construct a comprehensive biomarker system capable of supporting early screening, disease stratification, and prediction of treatment response. Pelvic MRI is the core imaging biomarker for the clinical diagnosis of deep endometriosis. T2‐weighted imaging can clearly display the morphology, size and adjacent relationship of deep lesions such as ovarian chocolate cysts, uterosacral ligaments and rectovaginal septum, providing precise imaging evidence for the formulation of surgical plans. The deep pelvic endometriosis index (dPEI) is a commonly used digital biomarker in clinical practice. By quantitatively scoring the distribution of lesions in 9 pelvic regions, it realizes the grading of disease severity. The scoring results can linearly predict the duration of surgery and the risk of postoperative complications, and is an important digital indicator for preoperative assessment and postoperative follow‐up.

In the realm of precision therapy for EMT, proteomic markers and genomics are collaboratively propelling the evolution of novel targeted approaches. The neurotrophic factor (NGF) has emerged as a prospective biomarker [798]. Corresponding targeted interventions, such as the pan‐Trk inhibitor entrectinib and anti‐NGF treatments, have demonstrated efficacy in alleviating pain and reducing lesion size in studies [799]. Concurrently, genomic investigations have broadened therapeutic horizons by uncovering detrimental coding mutations in the Neuropeptide S Receptor 1 (*NPSR1*) *TGCT* gene among patients. Notably, this gene exhibits specific expression in glandular epithelium. In animal models, the administration of NPSR1 inhibitors markedly mitigates peritoneal inflammation and pelvic discomfort, suggesting its potential as a pivotal therapeutic target [800]. Collectively, these insights delineate a translational medical trajectory from biomarker identification to targeted modulation, offering fresh avenues for both mechanistic exploration and clinical management of EMT.

#### Current challenges and future trends

EMT is currently confronted with multiple challenges, the most prominent of which is delayed diagnosis. The absence of non‐invasive early diagnostic tools possessing both high sensitivity and specificity often results in untimely patient identification. Furthermore, this disease displays considerable heterogeneity, with varying molecular characteristics across different clinical types, thereby complicating the diagnostic and treatment procedures. Concurrently, despite the discovery of numerous potential biomarkers, the majority have not been thoroughly validated through large‐sample prospective studies. This lack of validation impedes their integration into clinical guidelines and consequently the effective translation of relevant research findings into clinical practice.

To address the limitations of traditional EMT diagnostics, current research is pivoting towards innovative non‐invasive strategies. Central to this shift is the concept of multimodal integrated diagnosis. This approach entails creating a composite biomarker panel comprising “serum proteins (CA‐125 and Human Epididymis Protein 4), plasma miRNAs, and methylated cfDNA.” By employing artificial intelligence algorithms for comprehensive scoring and risk stratification, there is an aim to markedly enhance diagnostic accuracy. Building on this foundation, attention has also been directed towards exosome surfaceomics, specifically examining exosomes secreted by lesion cells [801]. Through screening for distinct proteins and microRNAs they carry, there is potential to substantially boost the sensitivity and clinical relevance of liquid biopsies. The overarching objective of these advancements is to achieve a genuine leap in non‐invasive diagnostic capabilities. In the future, it is expected that diagnoses can be carried out solely based on biomarkers present in blood, urine, or saliva, such as specific microRNAs and metabolites. This will streamline the diagnostic process and substantially increase the early disease detection rates.

### Benign prostatic hyperplasia

BPH is a prevalent benign urological disorder among middle‐aged and elderly men that significantly impairs the quality of life [802]. Current clinical biomarkers lack adequate specificity, frequently resulting in false positives that can be misconstrued as prostate cancer (PCa). Consequently, the development of efficient and reliable biomarkers for early screening, prognosis evaluation, and treatment guidance for BPH is of significant clinical importance (Table S66) [803].

#### The development of biomarkers

In terms of PSA era and histological confirmation, since the 1980s, prostate‐specific antigen (PSA) has been approved by the FDA for the screening, diagnosis, and monitoring of PCa and BPH. However, PSA levels may also increase in PCa and prostatitis, resulting in limited diagnostic specificity and potentially leading to unnecessary biopsies. Furthermore, PSA cannot predict patient response to medications such as 5α‐reductase inhibitors, limiting its value in guiding personalized treatment [804].

With the development of genomics and proteomics, research on BPH biomarkers has transitioned from single‐indicator dependence to multidimensional exploration. Inflammatory and oxidative stress markers have been confirmed to participate in the pathogenesis and progression of BPH, exhibiting diagnostic and prognostic potential [805]. Meanwhile, microRNAs in serum or urine show high stability, suggesting broad prospects as biomarkers for differential diagnosis and prognosis assessment of BPH [806].

Current research endeavors to integrate genomic, transcriptomic, proteomic, and metabolomic data in order to systematically map the molecular landscape of BPH, thereby identifying characteristic molecular fingerprints. By amalgamating biomarkers such as PSA, inflammatory factors, and microRNA, researchers are able to develop diagnostic or predictive models. Additionally, they are creating biomarkers to predict the efficacy of α‐blockers or 5‐α reductase inhibitors, thereby advancing precision medicine and personalized treatment for BPH.

#### Main types and typical cases of biomarkers

Among the diagnosis and screening biomarkers, PSA remains the cornerstone biomarker for BPH diagnosis, albeit with limited specificity. To improve diagnostic accuracy, clinicians often combine it with the Prostate Health Index (PHI) and Prostate Cancer Antigen 3 (PCA3), which were approved by the FDA in 2012 [807]. This combination not only improves the detection rate of BPH but also provides additional value for differentiating BPH from PCa [808].

Among the inflammation and oxidative stress markers, 8‐iso‐prostaglandin and pro‐inflammatory cytokines are abnormally elevated in BPH due to bladder wall ischemia‐reperfusion injury induced by bladder outlet obstruction [805]. The levels of these factors are significantly negatively correlated with urinary flow rate. They not only have diagnostic potential but also provide noninvasive monitoring evidence for assessing disease severity and adjusting treatment regimens.

Among the new protein biomarkers, several novel proteins have shown promise in the diagnosis and risk stratification of BPH and related prostatic disorders. Low *CD44* expression is associated with high Gleason grades and disease progression in PCa, suggesting its value as a tissue‐specific biomarker [809]. Serpin A outperformed PSA in differentiating BPH from PCa, especially within the PSA gray zone [810]. *CDK5RAP2* was specifically elevated in the serum of patients with BPH, facilitating differentiation between benign lesions and malignant tumors [811]. Prostate exosome proteins correlated with the severity of tissue inflammation and could predict surgical outcomes and inflammatory grading [812]. Furthermore, plasma urotensin‐2 was significantly elevated in metastatic PCa, independent of Gleason grade and PSA, indicating its potential role as a biomarker for assessing systemic disease burden and metastatic spread [813].

Among the lipid metabolism‐related markers, high‐density lipoprotein cholesterol (HDL‐C) and apolipoprotein A (ApoA) may have prognostic value in BPH, with higher levels associated with lower risk of BPH [803]. However, due to the lack of a clear cutoff value, they cannot be used as standalone diagnostic tests. They are more useful for understanding pathogenesis and identifying targets for metabolic intervention.

Among the imaging and digital biomarkers, correlative quantitative parameters of transrectal ultrasound (TRUS) and multiparametric magnetic resonance imaging (mpMRI) serve as commonly used imaging markers for clinical diagnosis and disease assessment of BPH. TRUS enables precise measurement of prostate volume and residual urine volume, facilitating evaluation of the degree of prostatic hyperplasia [814]. mpMRI provides apparent diffusion coefficient (ADC) and Prostate Imaging Reporting and Data System (PI‐RADS) score, which strongly suggest BPH lesions and effectively differentiate BPH from PCa presenting with low ADC values and high PI‐RADS scores [815].

#### Current challenges and future development trends

In terms of existing shortcomings and challenges, current PSA testing lacks adequate specificity and is subject to interference from prostatitis, PCa, etc. Meanwhile, the pathogenesis of BPH is complex, involving multiple factors such as inflammation, hormones, metabolism, etc. A single biomarker cannot comprehensively reflect the whole picture of the disease. Although new biomarkers are constantly being discovered, most of them remain in the research stage and lack multicenter prospective validation. Some biomarkers rely on tissue samples, limiting their clinical application. In addition, there is still a lack of biomarkers that can predict disease progression or drug response. The lack of standardization between different testing platforms also affects the comparability of results and their clinical application.

Future research will focus on cross‐omics integration to systematically elucidate the molecular network mechanisms underlying BPH. The discovery of novel molecular biomarkers is expected to improve differential diagnosis accuracy [806]. Epigenetic biomarkers such as steroid 5α‐reductase 2 (*SRD5A2*) promoter methylation may facilitate drug efficacy prediction, as expression silencing caused by *SRD5A2* methylation accounts for approximately 30% of natural finasteride resistance. Detection of this methylation could enable pretreatment screening of susceptible populations and guide combination therapy strategies [816]. Metabolomic applications will advance the identification of “metabolic fingerprints” associated with inflammation and oxidative stress while promoting the development of new biomarker panels based on noninvasive samples like urine, thereby improving screening convenience and accessibility.

### Ovarian cancer

Ovarian cancer (OC) is the most lethal gynecologic malignancy, among which high‐grade serous carcinoma is the most common and highly invasive [817]. Due to a lack of obvious early symptoms, more than 70% of patients are diagnosed at an advanced stage. The 5‐year survival rate has not improved significantly for a long time [818]. In this context, efficient and accurate biomarkers are crucial for early screening, prognostic evaluation, and individualized treatment (Table S67).

#### The development process of biomarkers

CA‐125, the most extensively characterized biomarker for OC, has long been utilized for monitoring treatment efficacy and detecting recurrence. However, its limitations in sensitivity and specificity have restricted its utility in population screening [819]. Advances in genomics, proteomics, and metabolomics have led to the systematic discovery of novel biomarkers, including ctDNA, microRNAs, and exosomes, propelling research into a multi‐dimensional exploration phase [820]. Current research is dedicated to integrating genomic, transcriptomic, proteomic, and metabolomic data with artificial intelligence and organoid models to construct a high‐sensitivity and high‐specificity multiplex biomarker system, thereby facilitating early diagnosis and precision therapeutic strategies [821].

#### Main types and typical cases of biomarkers

Within the precision diagnosis and treatment framework for ovarian cancer, the application of biomarkers has evolved from single diagnostic indicators to a multidimensional network encompassing diagnosis, classification, and targeted therapy. Among these, HE4 stands as the most valuable auxiliary diagnostic marker, demonstrating significantly higher specificity than CA‐125. It is FDA‐approved for use in monitoring disease progression or recurrence in ovarian cancer [634]. Combining both markers substantially improves diagnostic accuracy and has been formally incorporated into ovarian malignancy risk assessment algorithms [822]. In the field of liquid biopsy, ctDNA can detect *BRCA1/2* mutations in approximately 20% of patients with high‐grade serous carcinoma. These mutations not only indicate homologous recombination repair deficiency and carry diagnostic significance but also directly inform targeted therapies such as PARP inhibitors. Additionally, serum‐based microRNAs, such as the members of the miR‐200 family and miR‐21, which are highly expressed in patients, demonstrate promising diagnostic potential [823]. Methylation changes in promoter regions of genes such as *RASSF1A* and *BRCA1*, as early molecular events, further provide a new epigenetic direction for liquid biopsy. Proteomics has facilitated the discovery of novel biomarkers with dual therapeutic value. Mesothelin (MSLN) is overexpressed in 80% of epithelial ovarian cancers and has emerged as a key target for cell‐based therapies such as CAR‐NK [824]. Likewise, folate receptor α is highly expressed in tumor tissues and holds both diagnostic and therapeutic relevance [825].

Among the prognosis and predictive biomarkers, *BRCA1/2* mutation and homologous recombination repair deficiency status are key markers for predicting the efficacy of PARP inhibitors [826]. Patients with positive homologous recombination repair deficiency have a better response to platinum‐based chemotherapy and PARP inhibitors. By integrating proteomics and phosphorylation data, it was found that activation of the oxidative phosphorylation pathway is closely related to platinum resistance, and mitochondrial inhibitor IACS‐010759 has been successfully used to reverse drug resistance in preclinical models [826]. Organoid models provide a new platform for studying the mechanism of drug resistance. Recent study found that aurora A was significantly upregulated in cisplatin‐resistant patient‐derived organoids and promoted the formation of drug resistance by regulating cellular senescence and glucose metabolism [827]. Mechanistic studies showed that aurora A can directly phosphorylate transcription factor SOX8, thereby activating FOXK1 and affecting glucose metabolism and chemosensitivity. Spatial omics technology offers a novel perspective for the research on local resident biomarkers in OC. By means of spatial proteomics, it elucidates the potential biological mechanism underlying the malignant transformation from serous borderline tumors (SBT) to low‐grade serous ovarian tumors (LGSC) within serous ovarian tumors. It comprehensively characterizes the molecular features of SBT and LGSC and identifies new therapeutic targets. The combined treatment with milciclib, which targets CDK4/6, and mirvetuximab, which targets FOLR1, achieves significant tumor shrinkage in vivo. This not only provides in‐depth resources for subsequent disease research but also generates valuable discoveries with transformative value50.

#### Current challenges and future trends

Current early diagnosis and precise treatment of OC still face many challenges. First, the effectiveness of multi‐mode early diagnosis is insufficient [828]. The sensitivity of existing biomarkers for stage I OC is generally lower than 70%, and the positive predictive value in population screening is not high, limiting its clinical application value and dynamic evolution characteristics of tumors further increase the difficulty of diagnosis and treatment. There are significant differences in molecular characteristics among different pathological subtypes, and the spectrum of biomarkers also changes dynamically during the treatment process, making it challenging to accurately assess the condition [829]. At the same time, the high cost of multi‐omic detection and the lack of standardization of the process lead to low sensitivity and stability of ctDNA detection. Finally, the complexity of drug resistance mechanisms cannot be ignored, involving metabolic reprogramming, tumor microenvironment remodeling, abnormal DNA repair, and other biological processes, making it difficult for a single biomarker to comprehensively predict treatment response, thereby affecting the formulation of individualized treatment strategies.

Future research aims to establish a comprehensive technical system spanning from precise diagnosis to individualized treatment. Initially, the integration of ctDNA, protein biomarkers, and metabolites will facilitate the construction of a multimodal biomarker panel. By employing artificial intelligence for multi‐dimensional analysis, this approach can surmount the limitations inherent to single biomarkers. Building On this foundation, spatial omics technology will be utilized to elucidate the spatial interactions between tumor cells and their microenvironment at the site of origin, leading to the identification of more specific novel biomarkers. Ultimately, by leveraging liquid biopsy technology for real‐time monitoring of treatment response and incorporating patient‐derived organoid drug sensitivity assays, an “assessment‐treatment‐monitoring‐adjustment” loop for individualized treatment is envisioned [830]. This strategy seeks to translate pioneering discoveries into tangible clinical advantages.

### Cervical cancer

Cervical cancer (CCA) is associated with persistent high‐risk HPV infection [831]. Despite screening advances, advanced‐stage survival remains low [832]. Specific and sensitive biomarkers are needed for early diagnosis and personalized treatment (Table S68).

#### The development process of biomarkers

Squamous cell carcinoma antigen (SCCA) [833], cytokeratin 19 fragment (CYFRA21‐1) [834] and CEA [835] are the main indicators. SCCA is a classic marker for cervical squamous cell carcinoma, mainly used to monitor efficacy and provide early warning of recurrence; However, its sensitivity in early diagnosis is only 30−40%, and it is prone to false positives due to interference from benign diseases. CYFRA21‐1 and CEA have insufficient diagnostic specificity and are mostly used as auxiliary indicators in combination testing. With the elucidation of the pathogenic mechanism of HPV and the development of omics technology, research has shifted toward detection of HPV DNA/RNA and exploration of multi‐omic biomarkers. The discovery of circulating microRNA, gene methylation markers, and new indicators from proteomics provides a new approach to overcome the limitations of traditional biomarkers. Current studies have entered a stage of multidimensional integration. By integrating genomics, transcriptomics, proteomics, and epigenetic data combined with organoid models and artificial intelligence algorithms, a biomarker panel system with diagnostic and predictive value can be constructed to promote the transformation of biomarkers from basic research to clinical application.

#### Main types and typical cases of biomarkers

In terms of diagnosis and screening of biomarkers, high‐risk HPV *E6/E7* mRNA can directly indicate the active replication status of the virus. Its detection sensitivity and specificity surpass those of HPV DNA detection, effectively differentiating between transient and persistent infections. Circulating HPV DNA is a significant indicator for liquid biopsy and holds clinical value in HPV‐related tumors [836]. Further investigation into epigenetic and microRNA regulatory levels shows that promoter hypermethylation events in genes such as *MEG3*, *MGMT*, and *RASSF1A* are closely linked to tumor occurrence, development, and invasiveness. specific microRNAs, like miR‐146a, which promote cancer by regulating the NF‐κB pathway, and miR‐16, whose low expression positively correlates with tumor stage, not only contribute to pathogenesis but also enhance diagnostic efficacy when detected in combination [837, 838]. At the protein function level, novel protein markers like osteopontin show abnormally high expression in various malignant tumors. their levels significantly correlate with lymph node metastasis and poor prognosis in cervical cancer, suggesting their potential as valuable prognostic assessment indicators. These multi‐level markers, spanning genetics, epigenetics, and functional proteins, collectively provide a robust scientific basis for early diagnosis, risk stratification, and individualized treatment of diseases [839].

Among the prognosis and predictive biomarkers, immune therapy‐related markers, such as PD‐L1 expression levels, tumor mutational burden, and the composition of tumor‐infiltrating lymphocytes have become important criteria for evaluating whether patients can benefit from ICIs [840]. At the same time, the exploration and discovery of genomic instability markers have revealed that some CCAs exhibit homologous recombination repair defects, suggesting that it may be used as a potential predictor of PARP inhibitor sensitivity, opening up new directions for targeted therapy. In terms of treatment decision‐making at the preclinical research level, the newly established CCA organoid model has a unique role [841]. This model can not only directly evaluate drug response through *in vitro* drug sensitivity assays but also systematically screen abnormal pathways related to chemotherapy resistance through multi‐omics analysis, providing key experimental evidence for drug repositioning and combination regimen selection. These advances together constitute a complete research system from biomarker guidance to the verification of individualized treatment plans.

#### Current challenges and future trends

The early and accurate diagnosis and treatment of gynecological tumors continue to present numerous challenges. current technologies significantly fall short in distinguishing precancerous lesions during the initial diagnostic stage, thereby complicating precise risk stratification within high‐risk populations. Furthermore, due to the substantial heterogeneity of tumors, their molecular characteristics vary considerably across different pathological types and HPV infection subtypes [842]. This variation undermines the predictive efficacy of any single biomarker and impedes the construction of a universally applicable diagnostic model. Additionally, while multi‐omics detection technologies offer a wealth of information, their complex procedures, prohibitive costs, and the absence of standardized industry protocols severely limit their widespread adoption and use in grassroots medical institutions. Addressing these issues systematically is crucial for realizing the full potential of precision diagnosis and treatment for the majority of patients.

Future research aims to construct a comprehensive technical system spanning from precise diagnosis to individualized treatment. Initially, a multimodal integrated diagnostic system will be developed, incorporating HPV typing, DNA methylation detection, protein markers, and radiomics features. This approach forms a multi‐dimensional information network that encompasses molecular to imaging data, thereby significantly enhancing the accuracy of early diagnosis and classification. Subsequently, spatial omics technology will be employed to meticulously analyze the cellular structure and spatial interaction network within the tumor microenvironment. This analysis provides crucial evidence for identifying novel therapeutic targets and combinations for immune therapy. During the treatment phase, liquid biopsy technology will be utilized to dynamically monitor changes in therapeutic efficacy and to identify drug resistance mutations at an early stage, thus enabling real‐time tracking of treatment responses. Ultimately, through patient‐specific organoid drug sensitivity tests and multi‐dimensional biomarker analysis, the clinical implementation of truly individualized precision treatment can be achieved. This marks a significant shift from the “general model” to a “customized as needed” approach in tumor treatment [843].

### Endometrial cancer

Endometrial cancer (EC) is the most common gynecologic malignancy in developed countries. While many patients are diagnosed early, advanced‐stage and high‐risk subtypes pose survival challenges [844]. Biomarkers are transforming early detection and personalized therapy (Table S69).

#### The development process of biomarkers

In the early stage, CA125 and HE4 are the main markers. The specificity of HE4 is better than that of CA125. Combined detection of these two markers has become a common clinical monitoring method, but its sensitivity for early diagnosis is insufficient, and there are false positive problems in pelvic inflammatory diseases and benign ovarian diseases [845]. With the development of multi‐omics technology, researchers have begun to explore circulating microRNA (such as miR‐15a‐5p) [846], ctDNA mutation profiles (including *PTEN*, *PIK3CA*, *CTNNB1*, *POLE*, etc.) [847, 848] and proteomics techniques in biomarker discovery, opening up new directions for EC biomarker research. The current research focus has shifted toward developing a multi‐omics precision medicine model [849]. By establishing organoid libraries, applying spatial omics and artificial intelligence algorithms, systematic exploration is conducted to identify novel biomarker combinations with clinical application value for early diagnosis, prognostic assessment, and medication guidance.

#### Main types and typical cases of biomarkers

Among traditional biomarkers, the combined detection of CA125 and HE4 is currently a commonly used clinical method, and its diagnostic efficacy increases with age [850]. Among circulating nucleic acid markers, *PTEN* inactivation is the main driver of EC [851], while *TP53* inactivation is more common in serous carcinoma, high‐grade EC, and uterine carcinosarcoma [852]. The detection of these mutations by plasma ctDNA has the potential for early screening. Among microRNA markers, miR‐31 promotes tumorigenesis by inhibiting the Hippo pathway and can serve as a novel molecular marker to predict recurrence risk and prognosis [853]. Epigenetic markers such as *MGMT* gene methylation are abnormal at the pre‐cancerous stage and provide new targets for liquid biopsy [854]. A proteomic study also found that the *SIGLEC10* Q144K mutation affects the expression of the SIGLEC‐10 protein and interacts with estrogen receptor α to cause progesterone resistance, providing a new strategy for the prevention and early detection of early‐onset EC [855].

Among the prognosis and predictive biomarkers, POLE‐mutated patients have a good prognosis, with more than 90% surviving for 5 years; they usually do not require postoperative adjuvant chemotherapy [856]; while *TP53*‐mutated patients are mostly high‐risk subtypes and need intensive postoperative treatment [857]. This classification has been included in clinical practice guidelines. The establishment of organoid models makes it possible to test drug sensitivity in vitro. Studies have found that *ARID1A* mutation is associated with sensitivity to EZH2 inhibitors [858], and *PIK3CA*‐mutated patients may respond to PI3K inhibitors [859].

#### Current challenges and future trends

There are still multiple challenges in the field of early diagnosis and accurate detection of EC. First, the sensitivity of early diagnosis is significantly insufficient. Taking stage I EC as an example, the sensitivity of single biomarker‐based detection methods is generally lower than 60% [845], which makes it difficult to meet clinical needs for early screening. Second, tumor heterogeneity is another major obstacle. There are significant differences in gene mutations and expression profiles among different molecular subtypes, resulting in unstable predictive efficacy of a single biomarker and limiting its clinical application. In addition, there are prominent technical transformation barriers: multi‐omics detection processes are complex and analytical steps are numerous, leading to high overall costs [849]. At the same time, there is a lack of unified industry standards for sample processing, data interpretation, and quality control, and standardized systems need to be improved urgently. Only by systematically overcoming these bottlenecks can we truly promote the clinical transformation of early diagnosis and individualized treatment of EC.

A combined panel of ‘ctDNA (focusing on driver mutations such as *PTEN/PIK3CA* etc.) + protein biomarkers (CA125/HE4)’ can be constructed through multimodal integration, and AI algorithms can be introduced for in‐depth analysis to significantly improve the accuracy of early diagnosis and classification. In addition, using spatial omics technology to finely analyze the characteristics of spatial interaction between tumor cells and immune microenvironment can provide new insights into drug resistance and recurrence mechanisms. At the same time, developing exosome surface proteomics biomarkers aims to complement the shortcomings of existing liquid biopsy. On this basis, we can establish a dynamic monitoring system based on liquid biopsy, through continuous monitoring and combined drug sensitivity testing of organoids and molecular biomarkers, ultimately achieving dynamic evaluation and real‐time adjustment of individualized treatment plans.

### Breast cancer

Breast cancer (BCa) is the most prevalent malignancy in women worldwide [860]. Subtype heterogeneity and poor outcomes in advanced cases necessitate effective biomarkers for early detection and treatment guidance (Table S70) [861].

#### The development process of biomarkers

Biomarkers such as estrogen receptor (ER), progesterone receptor (PR) and human epidermal growth factor receptor 2 (HER2) have become the basis for molecular classification and treatment decision‐making in BCa [862]. However, these markers lack sufficient sensitivity for early diagnosis and are also expressed in benign breast lesions and other cancer types, limiting their screening value. Notably, approximately 15−20% of TNBC patients lack expression of all three receptors, resulting in limited treatment options [863]. With the maturity of omics technologies, research on biomarkers for BCa has entered a new phase. Researchers have begun to explore clinically valuable markers from multiple dimensions, including mutation profiles of ctDNA (such as *TP53* and *PIK3CA*), microRNAs (including miR‐21 and miR‐155), and proteomic alterations [863]. Current studies have entered the era of multi‐omics. By integrating genomic, transcriptomic, proteomic, and immunological data, this approach aims to deeply elucidate tumor heterogeneity and resistance mechanisms and discover novel therapeutic targets (PIK3CA and FGFR) [857]. Mammography, breast ultrasound and MRI are the core imaging markers in clinical practice.

#### Main types and typical cases of biomarkers

In terms of diagnosis and screening of biomarkers, when used alone, the traditional serum markers CA15‐3 and CEA have a sensitivity of less than 40% for early‐stage BCa. However, their combined detection can improve diagnostic efficiency in advanced BCa [864]. The detection of serum *HER2* extracellular domain (*HER2 ECD*) can help monitor disease status in patients with HER2 positivity [865]. In terms of circulating nucleic acid markers, *BRCA1/2* mutations are not only associated with hereditary risk of BCa but also guide targeted therapy using PARP inhibitors [866]. miR‐155 and miR‐21 are highly expressed in patient plasma and had screening potential [867]. The Mendelian randomization study, based on the analysis of genetic variations in the promoter region of the miR‐21 gene, found that the risk allele at this locus was associated with an increased risk of BCa, and the association was most significant for TNBC. DNA methylation markers show abnormalities in precancerous lesions and may provide early warning [868].

In the clinical diagnosis and research of BCa, molecular typing and biomarkers application is the core framework of precision medicine. Among them, ER, PR and HER2, as the “gold standard” for classification, directly determine whether patients can benefit from endocrine therapy and anti‐HER2 targeted therapy [869]; while in pathological morphology differentiation, E‐cadherin loss is a key indicator to distinguish lobular BCa from ductal BCa [870]. In order to further achieve individualized treatment, multi‐gene detection tools such as 21 gene test and 70 gene test are widely used to assess recurrence risk and guide adjuvant chemotherapy decision‐making. With the deepening of research, new biomarkers continue to emerge: for example, proteomics has found that high expression of PHGDH is related to chemotherapy resistance in TNBC, while studies on BCa organoids models have confirmed that patients with abnormal FGFR pathway are sensitive to corresponding inhibitors [871]. In terms of treatment, PARP inhibitors have become the standard treatment for advanced TNBC carrying *BRCA1/2* germline mutation [872]; at the same time, the application of liquid biopsy technology makes it possible to dynamically monitor the mutation status of genes such as *BRCA1/2*, providing a powerful tool for the whole management process [873]. Through spatial proteomics analysis, it has been discovered that the spatial distribution of immune cell markers within the breast cancer tumor microenvironment exhibits significant specificity. *CD8*‐positive T cells predominantly cluster at the tumor infiltration edge, thereby forming an anti‐tumor immune hotspot region. The extent of spatial co‐localization between CD8^+^ T cells and PD‐L1‐positive tumor cells in this region can serve as a local residence indicator for predicting the therapeutic efficacy of ICIs [874].

#### Current challenges and future trends

The current state of liquid biopsy technology presents several challenges for clinical application [875]. First, the concentrations of circulating nucleic acids and protein markers released by early‐stage tumors are exceedingly low. This results in a lack of sensitivity for early diagnosis and fails to meet screening requirements. Second, due to the high heterogeneity of tumors, there are significant differences in molecular characteristics among different lesions. This variability compromises the predictive efficacy of single or limited markers, thereby affecting the accuracy of clinical judgment. Furthermore, liquid biopsy lacks standardized protocols for sample processing, detection procedures, and data analysis, indicating a need for improved technical standardization. Additionally, the high cost of testing impedes its large‐scale application.

In order to facilitate a comprehensive enhancement in the precision of tumor diagnosis and treatment, we are dedicated to constructing a multi‐tiered and dynamic technical system [875]. Initially, we incorporate a multi‐dimensional liquid biopsy panel comprising “ctDNA mutations + protein markers” and employ advanced AI algorithms for thorough analysis, thereby significantly enhancing the accuracy of early tumor diagnosis and prognosis prediction. Furthermore, we utilize spatial omics technology to examine the heterogeneous spatial structure of the tumor microenvironment, yielding crucial insights into immune escape and drug resistance mechanisms. Concurrently, we are actively identifying surface protein and nucleic acid markers on exosomes to expand the detection parameters of liquid biopsy. Building On this foundation, we establish a dynamic monitoring system predicated on liquid biopsy to monitor the evolutionary trajectory of tumors in real time. This dynamic data is then integrated with results from organoid drug sensitivity tests and specific biomarkers to ultimately devise truly individualized, dynamic treatment strategies for each patient, thereby maximizing therapeutic efficacy.

### Prostate cancer

Prostate cancer (PCa) is a malignant neoplasm characterized by marked molecular heterogeneity [876]. Approximately 20% of patients have a 5‑year survival rate below 30% at the time of diagnosis. Therefore, identification and development of robust biomarkers for early detection, prognostic stratification and therapeutic guidance is of great clinical importance (Table S71).

#### The development of biomarkers

In terms of the era of traditional protein markers, since the 1980s, PSA has been progressively utilized in clinical screening and disease monitoring as a crucial biomarker [877]. However, despite its high sensitivity, PSA's specificity is comparatively low. This leads to an elevated rate of false positives and consequently, unnecessary tissue biopsies. These limitations have curtailed its extensive application in population screening.

As genomics, transcriptomics and proteomics technologies have matured, research on PCa biomarkers has entered a phase of multi‐dimensional exploration. The focus of this research has shifted from single molecules to system level analysis, leading to the discovery of novel markers such as microRNAs (such as miR‐375 and miR‐155) and proteomic changes. This expansion in molecular understanding enhances diagnostic and prognostic assessment capabilities [878].

Current research has entered the stage of multi‐omics integration. By integrating genomic, transcriptomic, proteomic and immunomic data and supplemented by intelligent algorithms, the heterogeneity and drug resistance mechanism of PCa are systematically revealed, which promotes the discovery and translational research of new targets such as melanoma cell adhesion molecule (METCAM) and Insulin‐like growth factor 1/Insulin‐like growth factor 1 receptor (IGF‐1/IGF‐1R) [879, 880].

#### Main types and typical cases of biomarkers

Among the diagnosis and screening biomarkers, PSA remains the fundamental indicator for clinical diagnosis and staging. Its combined detection with markers such as PHI and PCA3 can increase the detection rate and optimize risk stratification, demonstrating particular complementary value in differentiating BPH from PCa.

As carcinogenic factors, microRNAs (miR‐375 and miR‐141) promote tumor proliferation and invasion, and their stable presence in body fluids demonstrates excellent diagnostic properties and minimally invasive detection potential. Epigenetic markers such as methylation alterations of genes including Glutathione S‐transferase P1 (*GSTP1*) and *RAS* association domain family 1 (*RASSF1*), histone modifications (Histone H3 lysine 4 monomethylation (*H3K4me1*) and Enhancer of zeste homolog 2 (EZH2)), are abnormally expressed in early stage PCa, providing a new direction for the development of non‐invasive diagnostic tools.

In terms of proteomics and discovery of new biomarkers, studies have shown that METCAM is highly expressed in cancer tissues and promotes epithelial‐mesenchymal transition and tumor metastasis. The IGF‐1/IGF‐1R signaling pathway enhances cell survival and treatment resistance by activating the phosphatidylinositol 3‐kinase/protein kinase B/mammalian target of rapamycin (PI3K/AKT/mTOR) pathways [880]. As a cell surface receptor, receptor for advanced glycation end products (RAGE) is involved in chronic inflammatory responses and drives tumor progression [881]. It has great potential to differentiate benign from malignant lesions as well as advanced cancers, serving as a complementary tool to tissue biopsy.

Among the prognosis and predictive biomarkers, elevated chromogranin A (CgA) levels are strongly associated with neuroendocrine differentiation and disease aggressiveness, facilitating the identification of high‐risk patients and guiding chemotherapy selection [882]. Inhibitors targeting epigenetic regulators (including EZH2, Lysine‐specific demethylase 1 (LSD1) and Histone Deacetylase (HDAC)) are currently in clinical trials, offering novel therapeutic strategies for advanced PCa.

#### Current challenges and future development trends

In terms of existing shortcomings and challenges, although conventional biomarkers such as PSA are extensively employed in PCa clinical screening and disease monitoring, their limited specificity presents a significant obstacle to early diagnosis. This often results in an elevated number of false positives, leading to unwarranted tissue biopsies and causing undue psychological stress to patients. The substantial molecular heterogeneity inherent in PCa, coupled with the intricate nature of the tumor microenvironment, introduces formidable challenges in the quest for new biomarkers and in the development of targeted therapeutic agents. Furthermore, despite the promising potential demonstrated by numerous novel molecular markers, including microRNAs (miR‐375, miR‐141) and epigenetic markers like *GSTP1* methylation, their widespread clinical adoption remains hampered by several factors. These include insufficient sample sizes, the absence of standardized detection methodologies, and a lack of comprehensive multicenter validation studies. Consequently, the majority of these candidate biomarkers remain in preclinical or preliminary clinical trial phases, necessitating rigorous systematic validation and dedicated translational research before they can be integrated as standard clinical tools.

In terms of future research trends, the future development of PCa biomarkers will emphasize multi‐dimensional and systematic research strategies [883]. On the one hand, multi‐marker composite models based on multi‐omics integration (such as *GSTP1* methylation combined with miRNA expression profile) are expected to significantly improve diagnostic accuracy and prognostic ability, promoting personalized medicine. On the other hand, non‐invasive or minimally invasive detection technologies will gradually become mainstream, such as liquid biopsy technology using urine, plasma or exosomes, which may replace or complement existing PSA testing and biopsy procedures for early screening, dynamic monitoring and evaluation of treatment efficacy. Spatial transcriptomics has enabled precise mapping of biomarker expression in situ, revealing that key prognostic molecules such as SFRP4 are stroma‐specific and closely linked to extracellular matrix remodeling, which cannot be reflected by circulating liquid biomarkers alone [884]. In terms of treatment, rapid progress has been made in developing inhibitors targeting epigenetic regulators (EZH2, LSD1, and HDAC), some of which have entered clinical trials, providing new therapeutic hope for advanced patients, especially those with castration‐resistant PCa. In addition, with the deepening application of artificial intelligence and machine learning in medical data analysis, the discovery of biomarkers and clinical decision‐making process will become more efficient and accurate in the future, further promoting the early detection and precise treatment of PCa.

### Testicular germ cell tumors

Testicular germ cell tumors (TGCT) represent the most common malignant tumors of the male reproductive system in individuals aged 15–35. The identification of robust biomarkers for early diagnosis, risk stratification, and therapeutic guidance is of paramount clinical significance (Table S72) [885].

#### The development of biomarkers

The study of TGCT biomarkers has progressed from conventional serum markers to the integration of multi‐omics.

In terms of the era of classic serum markers, since the 1970s, alpha‐fetoprotein (AFP), β‐human chorionic gonadotropin (β‐hCG) and lactate dehydrogenase (LDH) have been established as cornerstone markers in clinical management of TGCT, especially for diagnosis, staging and efficacy monitoring of non‐seminoma [886]. However, these biomarkers lack sufficient sensitivity in early‐stage cases or cases of pure seminoma. Only approximately 15% of patients with pure seminoma and 50% of patients with non‐seminoma exhibit elevated levels, which restricts their application in comprehensive screening and accurate stratification.

With the development of genomics and proteomics, research has increasingly focused on detecting mutations in genes such as *KIT* and *KRAS* within ctDNA, as well as more specific molecular markers like microRNAs (miR‐371a‐3p), offering a novel approach to overcome the limitations of traditional serum biomarkers [887].

Current research is dedicated to the development of a precision medicine model, driven by multi‐omics. This involves the integration of genomic, transcriptomic, proteomic, and metabolomic data with AI algorithms. The ultimate goal is to devise innovative biomarker combinations for early diagnosis, prognosis evaluation, and personalized treatment strategies.

#### Main types and typical cases of biomarkers

Among the diagnosis and screening biomarkers, AFP, β‐hCG and LDH remain the cornerstone of clinical diagnosis and staging. The combined test significantly improves the detection rate of non‐seminomatous cell tumors, especially in mixed germ cell tumors, showing a complementary value. Recent studies have found that miR‐371a‐3p is significantly elevated in early stage TGCT, which has predictive potential for disease progression and recurrence/metastasis. A prospective study in 2019 showed that its AUC for diagnosing clinical stage I TGCT reached 0.958, indicating good early identification ability [888].

Epigenetic markers such as *RFPL3S* are significantly downregulated in TGCT and may serve as potential predictors of immune therapy response [889]. Proteomic studies have identified Sprouty homolog 4 (*SPRY4*) and its transcript, *SPRY4* intronic transcript 1 (*SPRY4‐IT1*), to be highly expressed in tumor tissues, potentially contributing to oncogenesis through the activation of the PI3K/AKT signaling pathway [890].

Among the prognosis and predictive biomarkers, genomic markers such as *KIT* mutations are associated with spermatogonial cell tumor development, while *TP53* mutations are frequently observed in patients resistant to chemotherapy [889]. These markers aid in identifying high‐risk populations and directing targeted therapeutic approaches. In the realm of immunotherapy, studies have demonstrated significantly elevated PD‐L1 expression in TGCT relative to normal tissues, indicating that PD‐1/PD‐L1 inhibitors could emerge as a novel therapeutic option for those resistant to chemotherapy [891].

#### Current challenges and future development trends

Current research and application of TGCT biomarkers face multiple challenges. In early diagnosis, traditional serum markers (AFP, β‐hCG, LDH) exhibit limited sensitivity for pure seminoma, resulting in approximately 85% of such patients being difficult to accurately identify through serological testing at early stages. Furthermore, the high heterogeneity of TGCT and the complex mechanisms of chemotherapy resistance, such as treatment resistance mediated by gene mutations like *TP53*, present difficulties for prognostic assessment and personalized treatment. Although novel molecular markers such as miR‐371a‐3p have demonstrated excellent diagnostic performance (with an AUC reaching 0.958) in studies, their detection methods remain unstandardized, restricting their application in large‐scale screening and clinical promotion [886]. Additionally, differences in molecular characteristics among various pathological subtypes pose challenges to the development of universal markers.

Future directions in TGCT biomarkers will focus on multimodal integration and AI‐assisted prediction [892]. For example, contrast‐enhanced CT imaging of postchemotherapy non‐seminoma patients combined with blood miR‐371a‐3p expression levels have been used to develop machine learning models that accurately predict the pathological outcomes of residual retroperitoneal lesions (necrosis/fibrosis vs. active tumor), which significantly improves the accuracy of treatment efficacy evaluation and helps some patients avoid unnecessary surgery. Meanwhile, the combination of patient‐derived organoid models and biomarkers (such as *KIT* mutation and miR‐371a‐3p) will promote the optimization of individualized therapeutic strategies and clinical transformation. In addition, with the continuous maturity of multi‐omics data integrated analysis and liquid biopsy technology, future efforts will be devoted to establishing multidimensional prediction systems based on genomics, transcriptome, and imaging features, so as to achieve comprehensive precision medicine for early diagnosis, risk stratification, and treatment monitoring.

### Dynamic monitoring network for biomarkers of reproductive system diseases

The reproductive system plays a pivotal role in human reproduction and endocrine regulation. The expression of biomarkers, including hormones, proteins, and metabolites, often exhibits periodicity and stage‐specific characteristics. For example, hormonal fluctuations during the menstrual cycle in women and the circadian rhythm of testosterone in men are well‐documented phenomena [893, 894]. Building on this foundation, our review systematically examines key diseases such as polycystic ovary syndrome, endometriosis, prostate cancer, and premature ovarian failure. We integrate biomarker data from various dimensions, including endocrine regulation, the local microenvironment, immune inflammation, and genetic epigenetics, to construct a comprehensive framework spanning “reproductive endocrinology–reproductive function” [895, 896, 897, 898]. This approach aims to enhance our understanding of the mechanisms underlying reproductive system diseases and to advance the development of precision medical strategies (Table S73).

#### Hierarchical network of markers for the hypothalamic–pituitary–gonadal axis

The foundation of reproductive endocrine regulation is established by the pulsatile secretion of gonadotropin‐releasing hormone (GnRH) from the hypothalamus. This secretion varies in frequency and amplitude, selectively modulating the synthesis and release of FSH and LH by the pituitary gland [899]. In the female reproductive cycle, FSH stimulates early follicular development. A surge in LH, prompted by estradiol (E2) secreted by the dominant follicle, is crucial for ovulation. Postovulation, progesterone (P) from the corpus luteum and E2 collaboratively maintain cycle equilibrium and periodic endometrial transformation through feedback mechanisms [900]. AMH from the granulosa cells of small antral follicles remains consistently stable throughout the cycle, making it a more reliable early quantitative indicator than basal FSH for assessing ovarian reserve function [901]. In male reproduction, inhibin B from testicular support cells provides specific negative feedback to suppress FSH secretion. Serum inhibin B concentrations accurately mirror the functional state of the spermatogenic epithelium, serving as a vital biomarker for evaluating male spermatogenic capability [902].

#### Series of markers for reproductive cell development and function

In the context of female reproduction, the precise prediction of the ovarian response to ovulation‐inducing drugs is essential for tailoring individualized treatment strategies. The follicular output rate (FORT) and ovarian sensitivity index (OSI) serve as dynamic functional metrics to gauge ovarian responsiveness. These indicators offer more direct insight into the actual reaction of the ovaries to gonadotropin stimulation than basic markers that merely indicate ovarian reserve, such as the AMH level and antral follicle count (AFC). Consequently, the FORT and OSI hold significant value in forecasting the number of retrievable oocytes and in detecting potential over‐response or under‐response [903]. With respect to optimal embryo selection, preimplantation genetic testing (PGT), which can be employed to examine the chromosome ploidy of blastocyst trophoblast cells, can increase the embryo implantation rate. The results of numerous studies have validated that PGT notably increases the embryo implantation rate and clinical pregnancy rate per transfer, especially in specific cohorts such as older individuals, individuals with repeated implantation failures, and individuals with recurrent miscarriages, while simultaneously mitigating miscarriage risk [904, 905]. In male infertility evaluations, the sperm DNA fragmentation index (DFI) and nuclear protein maturity are vital indicators that mirror the integrity and functionality of the sperm genome. Elevated DFI levels and nuclear protein packaging abnormalities may introduce compromised paternal DNA into the embryo, correlating with recurrent miscarriages, developmental anomalies in assisted reproductive technology embryos, and adverse pregnancy outcomes [904].

#### Reproductive system tumor marker spectrum

In the preoperative evaluation of ovarian tumors, the combined detection of CA125 and HE4, along with the integration calculation using the risk of ovarian malignancy algorithm (ROMA), effectively addresses the limitations of single markers. This approach significantly enhances the ability to differentiate between benign and malignant tumors and is especially beneficial for identifying early‐stage cancers [906]. In prostate cancer screening and diagnosis, to address the low specificity of prostate‐specific antigen (PSA), derived indicators such as PSA density (PSAD), PSA velocity (PSAV), and the ratio of free PSA to total PSA (f/t PSA) are typically employed in clinical settings. By considering factors such as prostate volume, dynamic changes in PSA levels, and PSA molecular forms, there has been a collective improvement in diagnostic specificity, leading to a reduction in unnecessary prostate biopsies [907]. Notably, the newly introduced Prostate Health Index (PHI) amalgamates data from the [−2]proPSA subtype, fPSA, and tPSA through a mathematical formula. This index has shown superior diagnostic performance compared with conventional indicators, enabling the more precise identification of clinically significant prostate cancer and thus refining the early diagnostic process [908].

#### Temporal and sequential dynamic changes in pregnancy‐related markers

During early pregnancy, human chorionic gonadotropin (hCG) is secreted exclusively by trophoblast cells and serves as the earliest detectable marker of pregnancy, observable 8–10 days postfertilization. The doubling time of the hCG serum level acts as a crucial dynamic indicator for assessing normal pregnancy progression and differentiating ectopic pregnancies [909]. For fetal chromosomal abnormality screening in early gestation, the combined assessment of maternal serum pregnancy‐associated plasma protein A (*PAPP‐A*), free β‐hCG, and ultrasound measurement of nuchal translucency (NT) thickness forms the primary biochemical component of the screening protocol, offering a high detection rate for Down syndrome [910]. In predicting obstetric complications, the placental pathogenesis of preeclampsia can be indicated by an imbalance in angiogenic factors. Notably, the ratio of soluble fms‐like tyrosine kinase‐1 (sFlt‐1) to placental growth factor (PlGF) has emerged as a sensitive integrated marker reflecting placental dysfunction. This ratio holds significant clinical value in disease prediction, auxiliary diagnosis, and severity evaluation [911].

### The reproductive system serves as the central hub for regulating the life cycle and metabolism

The reproductive system modulates reproductive functions via the secretion of sex hormones, thereby establishing a complex network of interactions with various bodily systems. This cross‐system integration of biomarkers underscores its dual critical role in developmental programming and metabolic regulation (Table S74).

#### Integration of the hypothalamus–pituitary–gonadal–metabolic axis

Sex hormones exert their effects through both genomic and nongenomic mechanisms to regulate the function of metabolic organs such as adipose tissue, muscle, and liver; they have a profound effect on systemic metabolic homeostasis [912]. Specifically, estrogen effectively enhances insulin sensitivity and promotes fatty acid oxidation by activating key signaling molecules such as AMPK and PPARα, thereby exerting metabolically protective effects [823, 913], whereas abnormal levels of androgen (either excess or deficiency) are often associated with insulin resistance and an increased risk of metabolic syndrome [914]. This association is particularly typical in PCOS, where hyperandrogenemia exacerbates insulin resistance, jointly driving a vicious cycle of reproductive disorders and metabolic aberrations [915]. Moreover, adipose tissue is not merely an energy storage organ but also actively participates in regulation via secreted adipokines. For example, adiponectin regulates systemic glucose and lipid metabolism and can directly inhibit androgen synthesis in the ovary [916]. A low adiponectin level serves as a critical link connecting metabolic disorders and reproductive endocrine disorders in patients with PCOS, highlighting the central role of the adiposity–reproductive axis in integrating systemic physiology.

#### Bidirectional regulation of the reproductive–immune–microenvironment axis

Sex hormones exert a significant regulatory influence on the immune system, providing a biological foundation for sex‐based differences in immune responses. Estrogen typically enhances humoral immune responses by acting on its corresponding receptors in immune cells. Conversely, androgens and progestogens predominantly exhibit immunosuppressive properties. Dynamic equilibrium between these effects is essential for maintaining immune homeostasis [917, 918]. This intricate regulatory network is most pronounced during pregnancy: At the maternal–fetal interface, key immune cells such as regulatory T cells, dendritic cells, and uterine natural killer cells are precisely modulated in terms of their phenotypes and functions. This modulation creates a distinctive microenvironment of immune tolerance, safeguarding the fetus from maternal immune rejection while also defending against potential infections [919]. Nonetheless, under pathological conditions, this regulation can become disrupted. For example, in endometriosis, the abnormal amplification of the proinflammatory effect of estrogen perpetuates the inflammatory response at the lesion site. Moreover, resistance to progesterone impedes its anti‐inflammatory and differentiation‐promoting actions, leading to persistent inflammation and establishing a detrimental cycle of chronic inflammation and disease progression [920].

#### The interaction of the bone–reproductive–endocrine axis

Estrogen is a pivotal hormone involved in maintaining bone homeostasis; it curtails osteoclast activity by upregulating the expression of osteoprotegerin (OPG) and suppressing the expression of the receptor activator of nuclear factor κB ligand (RANKL). Moreover, estrogen promotes osteoblast differentiation through the activation of the Wnt/β‐catenin signaling pathway [921]. Following menopause, a precipitous decrease in estrogen levels lifts the inhibitory constraints on osteoclasts, resulting in a marked increase in bone resorption. This phenomenon constitutes a critical mechanism in the pathogenesis and progression of osteoporosis among postmenopausal women [922]. Notably, in the context of male bone health, locally produced estrogen, which is derived from the conversion of androgens via aromatase, is equally indispensable, and estrogen deficiency can precipitate significant bone loss [923]. Recent research has revealed that the skeleton functions as an endocrine organ capable of modulating testicular testosterone synthesis through the secretion of “bone‐derived hormones” such as osteocalcin and adiponectin. This establishes a comprehensive bidirectional regulatory loop termed the “bone–reproductive axis,” underscoring the intricate interplay between the skeletal and reproductive systems [924].

#### Emerging field of the gut microbiota–reproductive axis

The gut microbiota, often referred to as a “virtual endocrine organ” of the human body, plays a pivotal role in modulating the intestinal–portal circulation of steroid hormones. This modulation significantly influences overall estrogen concentrations within the body. Central to this mechanism is the presence of a β‐glucuronidase‐rich microbiota that hydrolyzes conjugated estrogen, which is excreted by the liver into the intestinal lumen, where its converted into its active form, facilitating its reabsorption [925]. A bacterial community structure characterized by high enzyme activity can result in consistently elevated levels of active estrogen, thereby markedly influencing the risk of diseases associated with estrogen, such as breast cancer and endometriosis [926]. This “microbiota–endocrine” interaction holds particular significance in the context of PCOS. Research has indicated that disturbances in the intestinal microbiota of patients with PCOS precede metabolic irregularities such as insulin resistance. Moreover, when transplanted into animals, the microbiota from these patients can directly induce a disease phenotype [927]. These findings suggest that an imbalance in intestinal microecology might be instrumental in triggering PCOS via the “microbiota–reproductive axis” rather than merely representing a secondary alteration.

#### Developmental programming effect of the environmental–reproductive axis

Environmental endocrine disruptors, such as ubiquitous bisphenol A and phthalates, directly interfere with developmental programs and physiological functions of the reproductive system by mimicking or antagonizing endogenous sex hormones [893]. The adverse effects of environmental endocrine disruptors have been widely documented in human populations, including alterations in pubertal onset timing in children, diminished ovarian reserve function in women, and progressive deterioration of sperm quality in men [928]. Of greater concern is the potential for these chemicals to have transgenerational effects. The underlying mechanism involves the ability of environmental endocrine disruptors to induce stable epigenetic modifications in reproductive cells, such as alterations in DNA methylation and histone modifications. This misprogrammed epigenetic information can be transmitted to subsequent generations via gametes, constituting a key molecular basis through which environmental factors can induce transgenerational or even intergenerational health effects. This phenomenon provides a plausible explanation for the observed association between environmental exposure in ancestors and the reproductive disease risk of their grandchildren [926].

#### Neuro–reproductive axis integration

The central nervous system precisely regulates the pulsatile secretion of GnRH in the hypothalamus through neurotransmitters and neurohormones, thereby integrating neural signals with reproductive endocrine functions. Dopamine and GABA inhibit the activity of GnRH neurons, and glutamate and norepinephrine exert excitatory effects, collectively maintaining its secretory rhythm [927]. Glucocorticoids released by the HPA axis in response to stress can suppress GnRH transcription and secretion and diminish pituitary responsiveness, leading to a temporary inhibition of reproductive function, which is particularly evident in conditions such as chronic stress or exercise‐induced amenorrhea [929]. The frequency and amplitude of GnRH pulses are core biomarkers reflecting the function of the neuro‐reproductive axis, and abnormalities in these parameters are associated with disorders such as precocious puberty and hypogonadotropic hypogonadism [930]. Furthermore, the neuropeptide kisspeptin serves as an upstream integration factor that receives metabolic and stress signals and directly activates GnRH neurons, forming a critical link between environmental cues, nutritional status, and reproduction initiation [928]. Therefore, the neuro–reproductive axis maintains reproductive homeostasis via multilevel signal convergence, and its dysfunction constitutes the fundamental basis for various reproductive endocrine disorders (Figure S8).

## BLOOD AND THE IMMUNE SYSTEM

Comprehensive literature searches were undertaken in PubMed and Web of Science using Boolean logic with field‐restricted search strings. The specific Boolean queries were constructed for each hematological and immune disease as follows: for anemia, (“anemia” OR “Anemia”) AND (“biomarker”) AND (“disease”); for autoimmune diseases, (“autoimmune diseases” OR “autoimmune Diseases”) AND (“biomarker”) AND (“disease”); for acute myeloid leukemia, (“acute myeloid leukemia” OR “acute myelogenous leukemia” OR “leukemia, myeloid, acute”) AND (“biomarker”) AND (“disease”); for chronic myeloid leukemia, (“chronic myeloid leukemia” OR “chronic myelogenous leukemia” OR “leukemia, myeloid, chronic‐phase”) AND (“biomarker”) AND (“disease”); for Hodgkin lymphoma, (“hodgkin's lymphoma” OR “hodgkin lymphoma”) AND (“biomarker”) AND (“disease”); and for non‐hodgkin lymphoma, (“non‐hodgkin lymphoma” OR “non‐hodgkin's lymphoma” OR “lymphoma, non‐hodgkin”) AND (“biomarker”) AND (“disease”). The retrieval period was defined as January 1, 2020 through December 31, 2025. Only articles published in English were considered. Both original research articles and systematic or narrative reviews were included. Retrieved records were screened by titles and abstracts; articles deemed irrelevant to blood and immune system biomarkers, lacking full‐text availability, or containing incomplete data were excluded, and the remaining full texts were thoroughly evaluated. The selected disease spectrum encompasses anemia, autoimmune diseases, acute myeloid leukemia (AML), chronic myeloid leukemia, Hodgkin lymphoma, and non‐Hodgkin lymphoma, all representing major disorders of the blood and immune system with high prevalence, substantial disease burden, well‐established biomarker frameworks, and strong clinical translational relevance.

### Anemia

Anemia is a clinical syndrome of reduced red blood cell mass, leading to insufficient oxygen supply to tissues. It encompasses various subtypes such as iron deficiency anemia (IDA), megaloblastic anemia (MGA), aplastic anemia (AA) and hemolytic anemia (HA) [931]. Its diagnosis, classification, and prognosis depend on an effective biomarker system, which has evolved from traditional parameters to multidimensional molecular markers, enabling precision medicine (Table S75).

#### The development process of biomarkers

The progression of anemia biomarkers has evolved through three distinct phases, mirroring a developmental path from fundamental hematological indicators to molecular categorization, and transitioning from single‐parameter testing to a comprehensive, integrated evaluation.

Initially, basic hematological parameters such as hemoglobin, red blood cell count, and hematocrit were the mainstay for diagnosing anemia and assessing its severity. The introduction of nutrition‐related biomarkers like ferritin, vitamin B12, and folic acid markedly improved the etiological diagnosis of iron deficiency anemia and megaloblastic anemia [932]. While these biomarkers are advantageous due to their assay accessibility and widespread clinical application, their limited specificity in intricate or overlapping pathological scenarios restricted their efficacy in precise differential diagnosis. As immunology and proteomics technologies progressed, research emphasis transitioned towards identifying disease‐specific molecular markers. For aplastic anemia, immune‐associated markers such as soluble *CD117* [933] and anti‐COX‐2 autoantibody [934] have been pinpointed; for iron deficiency anemia, indicators like glutathione hemoglobin and zinc protoporphyrin/hemoglobin (ZPP/H) [935] have been established. These markers considerably elevate the precision in subtype differentiation and complex etiology diagnosis of anemia. Contemporary research is primarily centered on prognostic predictions and therapeutic guidance. Markers like Endothelial activation and stress index (EASIX) [936], apolipoprotein A (ApoA) [937], among others, aid in stratifying patient survival risks and forecasting treatment outcomes. Merging multiple indicators has emerged as the predominant strategy. By amalgamating traditional parameters with novel molecular markers, a holistic system encompassing diagnosis, classification, and prognostic evaluation is devised, facilitating tailored treatment decisions.

#### Main types and typical cases of biomarkers

Diagnosis and etiology of differential markers are used to confirm the presence of anemia, differentiate subtypes and identify causes, which form the basis for clinical diagnosis. Regarding traditional core diagnostic markers, hemoglobin is the gold standard for diagnosing anemia, with severity classified according to population‐specific thresholds [938]. Clinically, hemoglobin levels are typically used as the primary diagnostic criterion: hemoglobin levels below the normal range but above 90 g/L are classified as mild anemia; those between 60 and 90 g/L as moderate anemia; and below 60 g/L as severe anemia. There are significant differences in hemoglobin concentrations among children of different races. A study analyzed 1718 white, 741 black, and 315 oriental (Asian) healthy and non‐poor children aged 5 to 14. The results showed that the median hemoglobin concentration of black children was approximately 0.5 g/dL lower than that of white and oriental children (*p* < 0.001). Even after excluding abnormal hemoglobin (such as Hb S, Hb C), thalassemia traits, or iron deficiency, this difference still existed (0.5 g/dL). In contrast, the hemoglobin levels of white and oriental children were almost exactly the same. Approximately 10% of healthy black children will be misjudged as anemic when using a unified hemoglobin standard for anemia screening; therefore, it is recommended that racial differences be taken into account and that the hemoglobin threshold for black children be set approximately 0.5 g/dL lower than that for white children, so as to avoid overdiagnosis [939]. Ferritin reflects body iron stores and decreases early in IDA but may be falsely increased under inflammatory conditions. It needs to be combined with CRP for comprehensive judgment. Vitamin B12 and folic acid deficiency can cause MGA, and red blood cell folate is a better indicator of long‐term reserve status than serum folic acid.

Subsequent studies have identified novel etiology‐specific hematological markers that significantly enhance the diagnostic accuracy of anemia‐related diseases. Anti‐COX‐2 autoantibodies serve as specific markers for immune‐mediated aplastic anemia, exhibiting a positive rate as high as 83% in patients over 40 years old with the HLA‐DRB1*15:01 genotype [934]. Glutathione hemoglobin is a highly specific indicator for iron‐deficiency anemia, maintaining diagnostic accuracy even in the presence of inflammation [940]. Ektacytometry technology can evaluate red blood cell membrane function in hereditary spherocytosis and monitor the efficacy of splenectomy. ZPP/H can be utilized for diagnosing inflammation combined with iron‐deficiency anemia, effectively mitigating the false negative issue associated with ferritin [935].

Prognostic and therapeutic response predictive biomarkers are used to assess the risk of disease progression and treatment response, guiding clinical decision‐making for optimization. Soluble *CD117* is a dual diagnostic and therapeutic marker in severe aplastic anemia. A baseline level less than 50 pg/mL indicates shorter survival, while elevated levels indicate good treatment response [933]. Apo A can be used as a predictor of immunosuppressive therapy response, with levels below 1.205 g/L indicating better event‐free survival [937]. EASIX is a prognostic tool calculated from conventional parameters, where scores higher than 0.72 significantly increase all‐cause mortality (HR = 1.42) and predicts 10‐year mortality with an AUC of 0.78 [936]. IL‐1β is a mechanism marker and potential therapeutic target for CKD‐related anemia, and inhibiting its activity improves the anemic state [941].

#### Current challenges and future trends

In terms of existing challenges, the existing system of anemia markers presents several limitations. traditional indicators, such as ferritin, are susceptible to interference in complex conditions like inflammation [942]. Newer markers, for instance glutathione hemoglobin, despite their high specificity, are expensive and challenging to standardize due to their dependence on mass spectrometry technology. Furthermore, specific markers for certain rare subtypes of anemia are still lacking, impeding the achievement of precise diagnosis and treatment.

Future research should focus on the integration of multiple biomarkers and precision medicine: constructing a combined panel of “traditional indicators + new molecules” (such as ferritin combined with ZPP/H and glutathione hemoglobin) to enhance the ability to distinguish complex anemia; promoting the standardization and convenience of testing technologies, and improving accessibility at the grassroots level; formulating individualized treatment plans based on biomarkers, such as implementing anti‐inflammatory targeted therapy according to IL‐1β levels; continuously discovering biomarkers for rare anemia subtypes using multi‐omics technology, and improving overall diagnostic and therapeutic capabilities [943].

#### Autoimmune disease

Autoimmune diseases (such as SLE, antiphospholipid syndrome (APS), and Sjögren's syndrome (pSS)) cause tissue damage via immune dysfunction. Their complex pathogenesis and varied manifestations make early diagnosis, monitoring, and treatment challenging [944]. Consequently, biomarkers have emerged as crucial tools to address these clinical hurdles (Table S76).

#### The development of biomarkers

The study of biomarkers for autoimmune diseases has evolved from focusing on single indicators to integrating multi‐omics data, transitioning from empirical application to mechanism‐driven precision.

In the initial phase, research primarily centered on immunological assay techniques, which established autoantibodies such as anti‐nuclear antibody (ANA), anti‐double‐stranded DNA antibody (anti‐dsDNA), and anti‐Smith antibody (anti‐Sm), along with classical markers including complement C3 and C4 [945]. While these indicators are cost‐effective, relatively simple to perform, and remain fundamental to the diagnosis of conditions such as SLE, their limited specificity frequently impedes the accurate assessment of disease activity and organ involvement. For instance, ANA can be positive across a range of autoimmune disorders. With the emergence of high‑throughput sequencing, proteomics, and epigenetics, biomarker research has entered a diversified stage. Novel nucleic acid biomarkers such as circRNAs and lncRNAs, distinct immune cell subsets [946], and metabolites like L‑pyroglutamic acid have been successively identified [947]. Omics technologies have substantially expanded the dimensionality of biomarkers, laying the groundwork for deeper understanding of disease mechanisms and precise diagnosis. Current efforts focus on integrating multi‑omics data with clinical translation. By constructing combined biomarker panels, diagnostic performance has been markedly enhanced. For example, the combination of DEAD‑box helicase 5 (DDX5) with anti‑dsDNA and anti‑Sm antibodies achieved an AUC of 0.976 for SLE diagnosis [948]. The application of AI algorithms has further refined biomarker selection and validation, driving the expansion of biomarkers from purely diagnostic tools to multifaceted utilities encompassing disease stratification, activity monitoring, prognosis assessment, and treatment guidance.

#### Main types and typical cases of biomarkers

Among the autoantibodies and complement markers associated with SLE, anti‐Sm antibody exhibits a specificity of 99%, serving as a crucial serological indicator for SLE [945]. The anti‐TCP1 antibody demonstrates a positive rate of 79% in SLE cases and possesses considerable disease specificity [949]. In SLE patients, the pre‐collagen N‐proteinase *ADAMTS‐2* is upregulated, effectively distinguishing SLE and offering both diagnostic and therapeutic potential [950]. There's a significant association between the cytokine IP‐10 levels and SLE disease activity, particularly concerning the extent of arthritis involvement [950]. PS‐IgG complexes are found to deposit in the renal tissues of lupus nephritis (LN) patients, contributing to the pathogenesis of both SLE and LN [951]. Furthermore, *CD64* expression in SLE monocytes is heightened, playing a role in modulating skin inflammation [952].

Among the nucleic acid molecular markers of SLE, circular RNA *hsa_circ_0044235* and *hsa_circ_0001947* are downregulated in expression, and their combined detection can effectively distinguish SLE from rheumatoid arthritis (RA) [953]. *LINC00667* and *DANCR* are upregulated in plasma exosomes [954], demonstrating potential for diagnosis and activity monitoring. The long non‐coding RNA NR_103776.1 not only differentiates SLE from RA but also distinguishes between active and stable stages in SLE patients [955]. Circular RNA circGARS promotes the progression of SLE via the A20/NF‐κB signaling pathway and exhibits independent diagnostic value [956]. LncRNA *H19* is upregulated in the serum and PBMC of SLE patients; it inhibits cell viability by targeting miR‐19b, thereby possessing both diagnostic value and a pathological regulatory function [957]. In terms of immune molecule markers for SLE, semaphorin 4 A (Sema4A) is highly expressed on CD4^+^CD11c^+^ dendritic cells, which aids in differentiating SLE from RA [958]. Sialic acid‐binding Ig‐like lectin 1 (SIGLEC1), as an alternative interferon type marker, has a sensitivity of 98.7% and a diagnostic AUC of 0.95 [959]. Among the metabolite markers, l‐pyroglutamic acid is significantly elevated in SLE serum, with an AUC of 0.955 at a critical value of 61.54 μM, making it suitable for differentiating atypical cases [947].

In the diagnosis of APS, a combination of anti‐cardiolipin antibodies (aCL), anti‐β2‐glycoprotein I antibodies (anti‐β(2)GPI), and lupus anticoagulant (LA) forms the primary serological criteria. Notably, the highly specific anti‐β(2)GPI antibody is instrumental in supplementary diagnosis for patients testing negative for aCL. Furthermore, aPS/PT IgG correlates with increased stroke risk, while aAnxV IgM aids in identifying APS cases with negative serum results [960]. In parallel, the classification criteria for primary Sjögren's syndrome (pSS) predominantly feature anti‐Ro/SSA and anti‐La/SSB antibodies. The presence of anti‐Ro/SSA is linked to early disease onset and extranodal manifestations [961], whereas a positive rheumatoid factor suggests potential development of extranodal complications such as arthritis and skin vasculitis.

Among blood and protein markers, AGR is negatively correlated with the SLEDAI‐2K score and is an independent risk factor for disease activity [962]; urinary soluble *CD163* (usCD163) is positively correlated with the urine protein–creatinine ratio, reflecting LN severity [963]; while urinary immunoglobulin γ−3 chain C region (IGHG3) is a preferred indicator for noninvasive assessment of LN [964]. In addition, soluble CD14 (sCD14) was significantly elevated in plasma from patients with LN, which was helpful to predict renal damage progression [965]. NINJ1 was increased in serum from patients with SLE and could be used as a marker for hematologic involvement of SLE [966]. Among cytokine and chemokine markers, IL‐10 and LIF were positively correlated with disease activity [967]; CXCL13 was significantly elevated in active neuropsychiatric lupus (NPSLE), and it was a reliable indicator for activity evaluation [968]; sST2 was significantly elevated in pediatric SLE (pSLE) patients and decreased after treatment, allowing dynamic monitoring of disease changes. Among nucleic acid molecular markers, exosomal *DANCR* was positively correlated with SLEDAI‐2K and can be used as an indicator for dynamic monitoring [969]; lncRNA *SNHG1* was closely related to inflammatory indexes and reflected the disease state in real time [970].

In pSS, positive RF is associated with hypergammaglobulinemia and hypocomplementemia, suggesting that the disease may be active; patients who are anti‐Ro/SSA positive have a higher risk of extraglandular manifestations. In APS, an elevated titer of antibodies against anti‐β(2)GPI positively correlates with the risk of recurrent thrombosis.

Among the hematological markers, decreased mean platelet volume (MPV) was negatively correlated with cumulative organ damage and served as a risk indicator for long‐term prognosis; low expression of miR‐342‐3p was significantly associated with poor prognosis [971]. In terms of immune molecules and cell markers, increased proportions of CD226^+^ B cells could predict progression of organ damage [972]; high expression of C‐type lectin domain family 4 member E (*CLEC4E*) indicated poor prognosis and combined with traditional markers improved the accuracy of risk prediction (AUC = 0.9407) [973]. Regarding protein markers, high mobility group box‐1 protein (HMGB‐1) was associated with renal involvement and high titers of ANA and was an independent predictor of organ damage; pre‐granulysin (PGRN) participated in the process of kidney injury, and its elevated level indicated the risk of disease progression [974]. Moreover, among other autoimmune disease‐related prognostic markers, decreased levels of anti‐HSP90α IgG were associated with the risk of APS recurrence; patients positive for anti‐PS/PT IgG had higher risks of long‐term cardiovascular complications [960].

Among the predictive markers of treatment response in SLE, changes in B‐cell maturation antigen (BCMA) levels can predict the efficacy of belimumab [975]. Regarding resistance mechanisms and targets, radical S‐adenosyl methionine domain containing 2 (RSAD2) is involved in immune inflammation by regulating dendritic cell activation and could serve as a potential therapeutic target [976]; Sema5A promotes Th17 differentiation via the PI3K‐Akt‐mTOR signaling pathway, with its level positively correlated with IL‐17A [977]; SH2 domain‐containing protein‐tyrosine phosphatase‐2 (SHP2) is upregulated in SLE patients, and its inhibitor significantly improves renal pathology and immune disorders in animal models [978].

#### Current challenges and future prospects

In terms of current challenges, research on biomarkers for autoimmune diseases continues to face numerous challenges. The disease's high heterogeneity means that a single biomarker is inadequate for fully representing the complex condition [979]. Most new biomarkers remain in the exploratory phase, with a lack of standardization in detection methods. Furthermore, their reliability and universality require validation through large‐scale prospective studies.

Future research will concentrate on several key areas: the construction of comprehensive biomarker models through multi‐omics data integration; the development of non‐invasive dynamic monitoring technologies for urine biomarkers and exosomal RNA, among others; fostering collaborative advancement in targeted therapy and biomarker applications; and utilizing artificial intelligence and machine learning to enhance precision in disease classification, prognosis prediction, and treatment decision‐making [980].

### Acute myeloid leukemia

AML is a highly heterogeneous hematologic malignancy with widely varying prognoses, and high‐risk patients generally face high recurrence rate and short survival [981]. Early accurate diagnosis, prognostic stratification, and individualized treatment are key to improving outcomes, necessitating the discovery and clinical translation of effective biomarkers [982] (Table S77).

#### The development of biomarkers

The research on AML biomarkers has undergone an evolution, transitioning from the detection of single molecules to the multi‐dimensional and integrated applications. This progression can be categorized into three distinct stages.

The initial stage of biomarker development was defined by the use of chromosomal karyotyping and the identification of pivotal genetic alterations. Recurrent chromosomal abnormalities, such as t(8;21) and inv(16) [983], together with mutations in genes including nucleophosmin 1 (*NPM1*) and fms‑like tyrosine kinase 3 (*FLT3)*, formed the diagnostic and prognostic foundation for acute myeloid leukemia (*AML*) [984]. Clinical decision‑making increasingly incorporated cytogenetic and molecular profiling, allowing for refined disease classification and treatment selection. Nevertheless, the scope of these early approaches remained limited, leaving a proportion of patients without clearly defined biomarkers.

The advent of high‑throughput technologies, including genomic sequencing, transcriptomics, and proteomics, has enabled the discovery of novel biomarkers across multiple molecular dimensions. These encompass non‑coding RNAs (miRNAs, lncRNAs, circRNAs) and functionally relevant proteins such as branched‑chain amino acid transaminase 1 (*BCAT1*) and stathmin 1 (*STMN1*) [985, 986]. This phase facilitated a more integrated understanding of AML pathogenesis by correlating nucleic acid alterations, epigenetic modifications, and protein‑level dysregulation.

The current stage focuses on the systematic integration of genomic, transcriptomic, and proteomic data, supported by bioinformatics, to construct robust prognostic and predictive models. Advanced methodologies such as single‑cell sequencing and organoid culture systems are further accelerating the clinical translation of biomarkers, thereby enabling precise and individualized therapeutic guidance. Advanced spatial proteomics has further revealed that response to donor lymphocyte infusion therapy in AML is associated with a high diversity of immune neighborhoods and the colocalization of immune triplets (CD8^+^ cytotoxic T lymphocytes, B cells, and NK cells) within the bone marrow microenvironment. These spatial signatures reflect coordinated immune networks and can serve as macroscopic markers for evaluating therapeutic response.

Diagnosis and screening biomarkers are designed to enable early detection of AML, accurate subtype classification, and differential diagnosis from other hematological disorders such as myelodysplastic syndromes. For instance, the *RUNX1‐RUNX1T1* fusion gene defines core‐binding factor AML, a subtype typically associated with favorable responses to conventional chemotherapy [987]. In cytogenetically normal AML, *NPM1* and *FLT3* mutations serve as key diagnostic markers [984], while *PTBP1* expression has been shown to correlate positively with the proliferation marker Ki‐67 and negatively with the apoptosis marker p53 in AML patients [988]. Furthermore, *CD109* and low‐density lipoprotein receptor‐related protein 12 (LRP12) have been demonstrated to cooperatively promote immune evasion by modulating the T‐cell receptor signaling pathway, and their combined evaluation enhances diagnostic specificity [989]. Additionally, hypermethylation‐mediated downregulation of arachidonate 5‐lipoxygenase‐activating protein (ALOX5AP) has been validated as a diagnostically significant alteration across multiple independent patient cohorts [990]. IDH2 and IDH1 are two isozymes of isocitrate dehydrogenase, which are closely related to the occurrence of AML. As biomarkers for AML detection, IDH2 and IDH1 were approved by the FDA in 2017 and 2018, respectively [991].

In addition, for the clinical feature of thrombocytopenia in AML, a panel of RNA markers including colony stimulating factor 1 receptor (*CSF1R*), TNF super family member 15 (*TNFSF15*) and C‐Type Lectin Domain Containing 10A (*CLEC10A*) integrated into a SVM model (AUC = 0.847) with high accuracy to identify the thrombocytopenic subtype was developed [992]. This model provides a solid basis for further mechanism exploration and targeted therapy development.

Prognostic biomarker evaluation predicts disease progression and recurrence risk, thereby guiding the development of treatment strategies. Among core gene mutation biomarkers, *FLT3‐ITD* promotes poor prognosis by activating STAT5, with its allele ratio negatively correlated with prognosis [985, 993]; tumor protein 53 (*TP53*) mutations lead to loss of DNA repair function, indicating an extremely poor prognosis [994]. The R882 mutation in DNA methyltransferase 3A (*DNMT3A*) causes abnormal hypomethylation, inhibiting the cell differentiation process [995]. Notably, coexistence of a double mutation in CCAAT enhancer binding protein alpha (*CEBPA*) and ten‐Eleven Translocation 2 (*TET2*) worsens prognosis [988]. At the protein marker level, high expression of *ARHGAP43* indicates poor prognosis [996], while hematopoietic stem cell transplantation can improve outcomes; cytohesin 4 (CYTH4) promotes cell growth through immunomodulation and serves as an independent adverse prognostic factor [997]; stathmin 1 (STMN1) activates the PI3K/AKT pathway, and its expression level may be linked to survival [986]. Regarding non‐coding RNA biomarkers, high expression of miR‐362‐5p [998] and low expression of miR‐204 [999] both indicate poor outcomes; lncRNA *CRNDE* is significantly associated with reduced complete remission rates [1000]; *LOC541471*, *GDAP1*, *SOD1*, and *STK25* promote apoptosis to inhibit progression, and the multi‐gene risk model they participate in enhances prognostic prediction accuracy [1001]. For immune microenvironment markers, high *FLVCR2* expression inhibits NK and CD4^+^ T cell infiltration, suggesting poor prognosis [1002]; FGF1 Intracellular Binding Protein (FIBP) regulates T cell tolerance, with high expression shortening overall survival [1003]; high interleukin 1 receptor type 1 (IL1R1) expression increases recurrence risk, but transplantation can improve survival in intermediate‐risk patients [1004].

Monitoring of therapeutic efficacy biomarkers provides a basis for treatment plan selection, efficacy evaluation and drug resistance monitoring. At the genetic mutation level, branched‐chain aminotransferase 1 (*BCAT1*) promotes leukemia through the *FLT3‐ITD*/*BCAT1*/*MYC* pathway, and its expression level can guide the use of FLT3 inhibitors [985]; At the protein level, the expression levels of trophinin‐associated protein (TROAP) are related to the sensitivity of various drugs. It indicates that TROAP may be used as a potential molecular marker to predict drug efficacy and guide individualized medication [1005]; Wilms tumor 1 protein (WT1). Dynamic changes in activin A reflect the therapeutic response, and the reverse fluctuation of C‐X‐C motif chemokine ligand 12 (CXCL‐12) indicates microenvironmental changes [1006]; In non‐coding RNA, *circZBTB46* mediates ferroptosis resistance by regulating stearoyl‐CoA desaturase (*SCD*) expression [1007]; high expression of *Unc‐93B1* Regulator Of TLR Signaling (*UNC93B1*) leads to venetoclax resistance, and myeloid cell leukemia sequence 1 (MCL‐1) inhibitors can reverse this resistant phenotype [1008]. In terms of efficacy and recurrence monitoring, abnormal expression of CD7^+,^ CD56^+^, *HLA‐DR*‐ and *CD38*‐ is a core indicator for monitoring minimal residual disease [1009]; OPNb/OPNc subtypes promote recurrence through the VEGFc/STAT3/CXCR4 pathway [1010]; downregulation of dipeptidyl peptidase 4 (DPP4) and upregulation of transferrin receptor protein 1 (TFRC) jointly guide the assessment of chemotherapy resistance risk [1011].

#### Current challenges and future development trends

In terms of current challenges, research on AML biomarkers continues to encounter several challenges: the heterogeneity of the disease results in subtype‐specific prognostic significance for identical biomarkers [1012]; there is a lack of standardization in detection technologies and inter‐platform consistency; difficulties persist in integrating multidimensional data and fully developing composite biomarker panels; and clinical translation is hindered by the absence of large‐scale cohort validation for most biomarkers.

Future research should prioritize the integration of multimodal biomarkers to establish a comprehensive evaluation system that combines “gene mutations + non‐coding RNA + proteins + immunology.” This approach should leverage artificial intelligence to enhance predictive accuracy. The use of single‐cell sequencing and spatial omics technologies should be encouraged to decode the dynamic characteristics of tumor microenvironments. Real‐time monitoring of minimal residual disease through liquid biopsy, coupled with organoid drug sensitivity testing, can inform personalized treatment strategies. Furthermore, the development of immune‐related biomarkers should be augmented to provide novel targets for immunotherapy combination therapies.

### Chronic myeloid leukemia

Chronic myeloid leukemia (CML) is a hematopoietic malignancy progressing through chronic, accelerated, and acute phases. Accurate biomarker detection is essential for disease management and prognosis. The advent of tyrosine kinase inhibitors has transformed CML into a manageable chronic condition, underscoring biomarkers’ key role in diagnosis, treatment monitoring, and prognosis assessment (Table S78).

#### The development of biomarkers

The exploration of CML biomarkers has transitioned from single‐gene detection to multi‐omics integration. In the 1960s, a deletion in the short arm of chromosome 22, known as the Philadelphia chromosome, was identified in over 90% of CML patients. This chromosomal abnormality arises from the t(9;22)(q34;q11) translocation, leading to the formation of the *BCR‐ABL* fusion gene. The resultant constitutively active tyrosine kinase promotes malignant cell proliferation, solidifying its role as the molecular foundation for CML diagnosis and therapy. With progress in genomics, transcriptomics, and proteomics, the field has entered an era of comprehensive marker investigation. Alongside *BCR‐ABL*, emerging markers encompass microRNAs (miR‐20, miR‐106, miR‐222) [1013], long non‐coding RNAs (*MALAT1*, *CCAT2*) [1014, 1015], proteins (CIP2A, RPL13) [1016, 1017], and epigenetic alterations (*HOXA4* methylation, *H3K36me3* irregularities) [1018, 1019]. These discoveries offer deeper understanding into disease variability and mechanisms of drug resistance. Contemporary studies emphasize the amalgamation of data from genomics, transcriptomics, proteomics, and epigenomics, augmented by single‐cell sequencing, liquid biopsy, and artificial intelligence tools, aiming to devise a comprehensive biomarker framework for early detection, prognostic evaluation, and tailored therapeutic strategies.

#### Main types and typical cases of biomarkers

Among the diagnosis and screening biomarkers, the *BCR‐ABL* fusion gene serves as the most specific diagnostic marker for CML, exhibiting a positivity rate exceeding 95% in patients during the chronic phase. This marker was officially approved in 2017 for clinical testing such as CML efficacy monitoring and minimal residual disease assessment. The transcript subtypes of this gene hold clinical predictive significance: p210 (e13a2/e14a2) is indicative of the typical CML phenotype [1020], while p190 (e1a2) suggests the aggressive attributes of Ph+ ALL‐like disease [1021]. Additionally, p230 (e19a2) correlates with neutrophil proliferation [1022]. In terms of protein‐based markers, elevated levels of lactate dehydrogenase show a direct correlation with tumor burden, making it an economical and effective auxiliary diagnostic tool [1023]. *CD203c*, recognized as an activation marker for basophilic granulocytes, has expression dynamics that can mirror therapeutic responses [1024]. Furthermore, non‐coding RNAs present diagnostic potential for CML. Specifically, miR‐486‐5p plays an anti‐tumor role by modulating downstream pathways of *BCR‐ABL*; its downregulation may be linked to disease progression [1025]. Meanwhile, miR‐20, miR‐106, and miR‐222 contribute to leukemogenesis by influencing the cell cycle process [1013].

Among CML protein markers, high expression of cellular inhibitor of PP2A (CIP2A) was significantly associated with treatment failure and disease progression [1016]; anti‐silencing function 1A histone chaperone (*ASF1A*) was abnormally highly expressed in patients at the blast crisis stage and promoted malignant transformation by activating the NOTCH pathway [1026]. In terms of genetic markers, ASXL Transcriptional Regulator 1 (*ASXL1*) mutation increased the risk of drug resistance and disease progression by 32 times and 63 times respectively [1027]; decreased H3K36me3 level could be used as an epigenetic warning indicator; mouse doubleminute 2 homolog (*MDM2*) and *TP53* specific SNPs were related to good prognosis [1028]. In terms of immune microenvironment indicators, changes in the proportion of effector regulatory T cells were closely related to remission status after discontinuation [1029]; eosinophilia was a clinical parameter with independent prognostic value. Spatial proteomics has revealed that in advanced CML, PD‐1^+^CD8^+^ T cells are specifically enriched in leukemic lesions and form direct physical contact with PD‐L1^+^ antigen‐presenting cells and leukemia cells, highlighting local immune exhaustion as a clinically relevant spatial marker [1030].

In terms of treatment of drug resistance‐related markers, studies on the mechanisms of drug resistance have identified several key biomarkers. The expression of alpha‐enolase (*ENO1*) is linked to tyrosine kinase inhibitor (TKI) sensitivity, and its inhibition can induce ferroptosis, thereby reversing drug resistance [1031]. Conversely, the deletion of histone‐lysine N‐methyltransferase 2D (KMT2D) contributes to the development of drug resistance, suggesting that restoring its expression could be a viable strategy for reversal [1032]. Epigenetically, elevated methylation of the Homeobox A4 (*HOXA4*) promoter is significantly correlated with imatinib resistance [1018]. Among non‐coding RNAs, increased expression levels of Colon Cancer Associated Transcript 2 (*CCAT2*) and metastasis associated lung adenocarcinoma transcript 1 (*MALAT1*) are indicative of resistance to imatinib and dasatinib, respectively [1014, 1015]. Additionally, downregulation of miR‐495‐3p plays a role in the mechanism underlying TKI resistance [1033].

A Mendelian randomization study revealed that 4‐methyl‐2‐oxoglutarate has the strongest positive causal relationship with the pathogenesis of CML, suggesting that this metabolite may play a key role through pro‐inflammatory processes, thereby emerging as the strongest risk factor for CML. It may promote disease progression via pro‐inflammatory responses and could serve as a key biomarker [991].

#### Current challenges and future development trends

In terms of existing challenges, the current research on biomarkers for drug resistance mechanisms in CML is fraught with numerous challenges [1034]. The complexity of these mechanisms, coupled with the fact that *BCR‐ABL*‐independent pathways such as metabolic reprogramming and epigenetic changes have yet to be fully elucidated, presents a significant hurdle. Additionally, there are no reliable markers available for predicting relapse following drug withdrawal. Furthermore, the clinical application of multi‐omics detection technologies is impeded by cost considerations and standardization issues.

Future research will concentrate on multimodal integration and precision medicine, aiming to construct a comprehensive biomarker panel comprising “*BCR‐ABL* + mutation spectrum + non‐coding RNA + proteome” [1035]. This will be combined with artificial intelligence for dynamic risk assessment. The heterogeneity of leukemia stem cells will be analyzed using spatial omics and single‐cell techniques. Additionally, the development of exosome and ctDNA detection methods for non‐invasive monitoring is planned. Finally, individualized combined treatment strategies will be developed based on immune markers and novel molecular targets.

### Hodgkin lymphoma

Hodgkin lymphoma (HL) is a malignant proliferative tumor of the lymphatic system. Despite a high overall cure rate, 10−30% of patients exhibit primary refractory or early recurrence [1036]. Overcoming current clinical limitations requires effective biomarkers to improve diagnosis, risk stratification, and therapeutic monitoring, ultimately optimizing patient outcomes (Table S79).

#### The development of biomarkers

The research on biomarkers for HL has evolved from single‐protein detection to multidimensional and multi‐technology integration. Cell surface protein markers such as *CD30* and *CD15* form the core basis of HL diagnosis. As a member of the tumor necrosis factor receptor superfamily*, CD30* is highly expressed in hodgkin‐reed‐sternberg (HRS) cells and serves not only as a key indicator for diagnosing classical HL but also as an important target for targeted therapy [1037]. During this stage, these markers were primarily detected using immunohistochemical techniques; although they facilitated disease classification and differential diagnosis, their utility for predicting treatment efficacy and providing precise prognostic assessments was limited. With advancements in molecular biology technology, research expanded into multiple dimensions, including nucleic acids, soluble proteins, and inflammation‐related indicators. Nucleic acid markers, such as circulating tumor DNA and miRNA, along with soluble proteins like thymic and activating regulatory chemokine (TARC) [1038] and soluble programmed cell death ligand 1 (PD‐L1) [1039], were subsequently identified. Hematological indicators, such as the platelet/lymphocyte ratio and the modified endothelial cell activation and stress index, also demonstrated potential for prognostic assessment. Concurrently, Detection techniques expanded to include RT‐qPCR, next‐generation sequencing, and flow cytometry.

Current research efforts are increasingly oriented towards the amalgamation of multiple markers and the incorporation of various technologies. This involves the fusion of imaging techniques with artificial intelligence algorithms to construct a highly accurate assessment system. For instance, the joint detection of exosomal miRNA and serum TARC, along with the integration of circulating tumor DNA and international prognostic scores, has significantly enhanced prediction accuracy [1040]. The utilization of spatial omics and single‐cell sequencing technologies has further elucidated the mechanisms of tumor microenvironment‐related markers, thereby facilitating the transition of these markers from mere detection tools to instruments for treatment guidance.

#### Main types and typical cases of biomarkers

Diagnostic and classifying biomarkers are primarily utilized for the diagnosis of HL and to differentiate it from other lymphoma types. *CD30* serves as the most specific diagnostic marker for HL, with Its expression in HRS cells marking a significant distinction from non‐Hodgkin lymphoma [1037]. When combined with *CD15* detection, diagnostic accuracy can be further enhanced: *CD15* positivity rates in HL range between 70 and 80%, whereas it is predominantly negative in other *CD30*‐positive tumors such as anaplastic large cell lymphoma. As auxiliary diagnostic markers, *CD20* typically exhibits negativity or weak positivity in HL tumor cells, aiding in the exclusion of B‐cell non‐Hodgkin lymphoma [1041]; moreover, serum thymidine kinase 1 and EBV DNA hold substantial reference value, with EBV DNA being particularly suitable for the non‐invasive detection of classic EBV‐positive HL, and Mendelian randomization analysis confirmed a significant causal relationship between EBV DNA load and increased risk of Hodgkin lymphoma (*p* = 1.8 × 10^−3^) [1042, 1043]. Technological advancements continue to introduce new diagnostic combinations. For instance, the use of electrochemical biosensors to detect serum CCL17 effectively distinguishes HL patients from healthy individuals [1044], while employing a high‐performance sliding micro‐nipple lateral flow immunoassay device to measure C‐reactive protein in interstitial fluid offers an innovative approach for the non‐invasive diagnosis of HL [1045].

Treatments and prognosis biomarkers are used to evaluate treatment efficacy, predict the risk of recurrence and guide individualized treatment. Among immune therapy‐related markers, phosphorylated PD‐1 can label dysfunctional T cells, and its dynamic changes in CD8‐positive tumor‐infiltrating lymphocytes can effectively evaluate the efficacy of PD‐1 immune checkpoint blockade therapy. At the same time, for patients with relapsed/refractory classical HL, those who received anti‐PD‐1 treatment with a platelet/neutrophil ratio ≥51.6 had significantly better complete response rates and progression‐free survival than those without treatment. In terms of circulating markers and minimal residual disease monitoring, the area under the curve of combined detection of plasma exosomal miRNA and serum TARC in predicting PET status reached 0.93, with sensitivity and specificity reaching 93.5%, which could effectively identify minimal residual disease [1046]. In addition, circulating tumor DNA load is an independent prognostic factor, and patients with high levels before treatment have significantly lower 3‐year progression‐free survival rates than those with low levels [1047]. Among inflammation‐ and hematology‐related markers, serum IL‐6 and hs‐CRP were positively correlated with the risk of recurrence [1048]; while platelet/lymphocyte ratio ≥245 and platelet/platelet distribution width <24.5 were independent risk factors for poor prognosis in advanced classical HL [1049]; and modified endothelial cell activation and stress index ≥17.28 could predict primary refractory disease [1050]. In terms of imaging biomarkers, quantitative Deauville score qPET further divided patients with traditional visual score 5 into high‐risk and low‐risk subgroups by quantifying tumor metabolic activity, providing a precise basis for adjusting treatment strategies [1051].

Target related biomarkers provide a molecular basis for the selection of targeted therapeutic agents and promote the development of individualized treatment. In CD30‐targeted therapy, the efficacy of anti‐CD30 monoclonal antibodies depends on the expression level of *CD30* in tumor cells, which needs to be determined by immunohistochemical methods [1037]. Among apoptosis regulation‐related markers, high BCL‐2 expression in HL provides a direction for targeting the apoptotic pathway [1052]. Furthermore, among signaling pathway‐related markers, abnormal activation of JAK/STAT pathway is common in HL, and colony stimulating factor 3 receptor (CSF3R) can serve as an alternative biomarker for hyperactivation of this pathway. Relevant JAK inhibitors have entered early clinical trials [1053].

#### Current challenges and future development trends

Current research on HL biomarkers faces several challenges: at the detection level, the sensitivity of a single biomarker is limited, making early warning of disease recurrence difficult; in terms of technology transfer, multi‐omics detection is hindered by high costs, complex operations, and a lack of standardized procedures, thus limiting its clinical application; some potential biomarkers remain in the research phase, and their clinical value requires validation through large‐scale studies.

Future research will focus on multi‐dimensional integration and the impetus provided by cutting‐edge technologies. By constructing a multimodal biomarker panel that integrates circulating tumor DNA, TARC, quantitative imaging, and clinical parameters, and incorporating artificial intelligence algorithms, assessment accuracy can be significantly improved. The in‐depth application of technologies such as spatial omics and single‐cell sequencing will offer new perspectives for elucidating the interactions within the tumor microenvironment and the functional mechanisms of biomarkers. A dynamic monitoring system based on liquid biopsy, combined with biomarker profiling analyses like organoid drug sensitivity and platelet/neutrophil ratio, will facilitate the advancement of treatment strategies toward individualization and real‐time optimization. Advancements in non‐invasive detection technologies, including interstitial fluid detection and the development of real‐time devices, will improve testing accessibility and patient compliance, collectively promoting the clinical translation of precision medicine.

### Non‐hodgkin lymphoma

Non‐Hodgkin lymphoma (NHL) is a highly heterogeneous malignancy derived from the lymphoid hematopoietic system, with aggressive subtypes like extranodal NK/T‐cell lymphoma (ENKTL) and relapsed/refractory diffuse large B‐cell lymphoma (R/R DLBCL) exhibiting poor clinical outcomes [1054]. Therefore, identifying effective biomarkers for precise diagnosis, prognostic stratification, and drug resistance monitoring is critical to advance NHL precision medicine (Table S80).

#### The development of biomarkers

Research on NHL biomarkers has systematically evolved from conventional single indicators to multi‐omics integration, and from tissue detection to liquid biopsy. Initial studies predominantly relied on protein biomarkers in tissue specimens. Ki‐67, a cell proliferation index, was found to be highly expressed in patients with diffuse large B‐cell lymphoma (DLBCL) and was associated with poor prognosis, thus becoming a routine pathological assessment indicator [1055]; *CD20* not only served as a marker for B‐cell NHL classification but also as the target of targeted drugs such as rituximab [1056]. The function of biomarkers at this stage was relatively simple, making it difficult to accurately predict treatment response and the risk of drug resistance. With the development of technologies such as gene sequencing and proteomics, NHL biomarker research entered the molecular level. Nucleic acid biomarkers such as miRNA (miR‐340‐5p, miR‐146a) [1057, 1058], lncRNA (*OR2A1‐AS1*) [1059], and mRNA (*PLA2G7*, *RFC3*) [1060, 1061] were successively discovered. These participate in tumorigenesis by regulating immune escape and cell proliferation through various mechanisms. The analysis of ceRNA networks (*has‐*miR‐20a‐5p/*SNHG6*) laid the theoretical foundation for multi‐molecule combined detection [1062]. Current research focuses on constructing an assessment system that integrates multiple biomarkers, including nucleic acids, proteins, immune cells, and cytokines. Novel biomarkers, such as immune cell markers (CD8^+^ NK cells) [1063], cytokine combinations (the ratio of IL‐10/IL‐6) [1064], and phosphorylated proteins (Ikaros‐pSer442/445), have continuously emerged [1065]. The maturation of liquid biopsy technology has facilitated the clinical application of non‐invasive samples, such as serum, plasma, and cerebrospinal fluid, marking a shift towards precision and individualization in the diagnosis and treatment of NHL.

#### Main types and typical cases of biomarkers

Diagnostic and classifying biomarkers provide molecular evidence for the early detection, subtype differentiation and complication identification of NHL. Among specific protein markers, *CD20* and *CD3* are the diagnostic basis of B‐cell and T‐cell NHL respectively [1066]; the nuclear staining rate of DNAJ heat shock protein family member C9 (DNAJC9) is increased in orbital adnexal zone lymphoma, suggesting its potential as a marker for rare subtypes; stimulator of interferon genes (STING) is specifically activated in T/NK cell NHL but downregulated in B‐cell subtypes, which can be used as an indicator to identify sources [1067]. In combination with nucleic acid and protein markers, serum levels of cathepsin S (CTSS) were significantly elevated in patients with NHL (AUC = 0.766), and positively correlated with specific HDL subtypes [1068]; meanwhile, 12 proteins such as *CD82*, *CD55* and *CD36* were overexpressed in R/R DLBCL regardless of cell origin, forming an exclusive diagnostic panel [1069]. Regarding complication diagnosis‐related markers, serum IL‐10 > 180.70 pg/mL has a diagnostic sensitivity of 87.50% and specificity of 96.08% for DLBCL combined with sHLH [1061]; an elevated ratio of IL‐10/IL‐6 in cerebrospinal fluid was associated with secondary central nervous system lymphoma and could assist in identifying neurological involvement.

Prognostic biomarker was used to predict the risk of disease progression and survival outcomes. Among the nucleic acid markers, high serum miR‐146a levels were associated with a favorable prognosis in patients treated with R‐CHOP [1070]; low expression of lncRNA *OR2A1‐AS1* was associated with shorter survival time in DLBCL patients and had independent prognostic significance; moreover, high expression of nuclear receptor subfamily 3 group C member 1 gene (*NR3C1*) was an independent factor for good prognosis in the CHOP treatment subgroup [1071]. In terms of protein and immune cell markers, high expression of chimerin 1 (CHN1) was associated with a favorable prognosis in DLBCL [1072], and this association was particularly significant in the germinal center B‐cell (GCB) subtype and advanced‐stage patients; meanwhile, baseline CD8^+^ NK cell level was closely related to response rate and survival time in relapsed/refractory DLBCL patients who received R2‐GDP treatment; in HIV‐related DLBCL, *CD4* count <150 cells/μL and neutrophilia were confirmed as independent risk factors for poor prognosis [1073]. Regarding ceRNA network‐associated markers, combined plasma *has‐*miR‐20a‐5p/*SNHG16* and *has‐*miR‐181a‐5p/*SNHG6* expression was independently associated with aggressive disease behavior and survival outcome; whereas high combined *has‐*miR‐150‐5p/*MALAT1* and *has‐*miR‐335‐5p/*NEAT1* suggested advanced disease progression and poor prognosis [1074].

^18^F‐FDG PET/CT plays an indispensable role in the diagnosis and treatment of NHL. The system provides accurate staging, utilizing parameters such as standardized uptake value (SUV), Deauville score, and total glycolysis. The SUVmax value can aid in identifying lymphoma subtypes, for instance, studies have demonstrated that SUVmax is particularly advantageous for distinguishing aggressive DLBCL from indolent follicular lymphoma (FL). Furthermore, baseline total lesion glycolysis (TLG) is an effective prognostic indicator for aggressive NHL. The Deauville score remains the core standard for interim and end‐of‐treatment efficacy assessment.

Therapeutic response and drug resistance prediction biomarkers guide the selection of treatment regimens and predict drug resistance. Among targeted therapy‐related biomarkers, low levels of miR‐340‐5p were associated with poor response to pembrolizumab in ENKTL patients; *BTK* ^C481S^ mutation led to mantle cell lymphoma resistance to conventional BTK inhibitors; *CD19* was persistently expressed during B‐NHL relapse and was an important alternative target after anti‐CD19 failure [1075]. In chemotherapy‐ and epigenetic therapy‐related biomarkers, high expression of B‐cell lymphoma 6 (*BCL6*) conferred resistance to idelalisib in DLBCL [1076], which could be reversed by lenalidomide; high expression of *PLA2G7* was related to tumor microenvironment characteristics, and its inhibitor induced apoptosis of DLBCL cells; SH3 domain containing GRB2 like 1 (SH3GL1) inhibited doxorubicin‐induced apoptosis through regulating ferroptosis [1077]. For immunotherapy‐related biomarkers, PD‐L1 was highly expressed in circulating lymphocytes in NHL and was a key mediator of immune escape [1078]; phosphorylation level of Ikaros‐pSer442/445 predicted the therapeutic response to novel BTK inhibitor TG‐1701.

#### Current challenges and future development trends

The use of NHL biomarkers is currently fraught with several challenges [1079]. These include inadequate sensitivity for early diagnosis, which complicates the identification of asymptomatic patients. Additionally, there is a lack of standardization in testing procedures, leading to poor comparability of results due to varying technologies and sample types. The significant heterogeneity of tumors further limits the predictive efficacy of these biomarkers. Moreover, the majority of new biomarkers are still in the research phase, and the widespread adoption of liquid biopsy and other technologies is hindered by cost and standardization issues.

Future research will focus on several key areas: the construction of a biomarker panel that integrates multiple omics data, and the use of artificial intelligence to develop combined detection methods [1080]. There is also a need to optimize liquid biopsy technology to facilitate the progression of non‐invasive detection towards standardization and high sensitivity. Additionally, individualized treatment plans can be formulated based on the characteristics of biomarkers such as *PLA2G7* and BTK mutations, as well as organoid models. Further research into the pathological mechanisms of biomarkers is required to promote the development of targeted drugs and their clinical translation (Figure S9).

### Dynamic monitoring network for biomarkers of blood and immune system diseases

**FIGURE 6:**
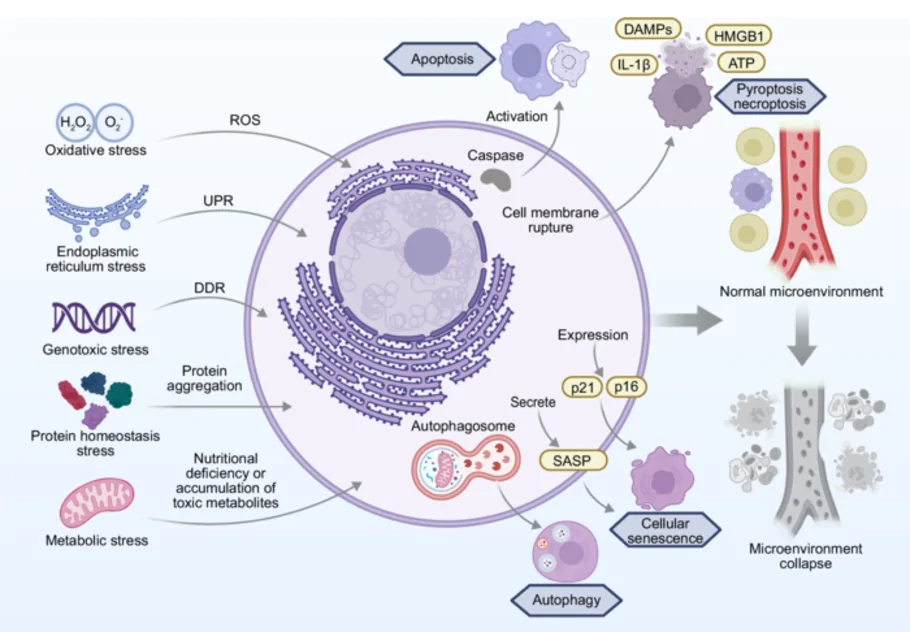
Schematic diagram of mechanisms leading to microenvironment collapse due to cellular stress and imbalance in cell fate. This figure illustrates how multiple forms of cellular stress disrupt intracellular homeostasis and drive pathological remodeling of the tissue microenvironment. Oxidative stress, endoplasmic reticulum stress, genotoxic stress, protein homeostasis stress and metabolic stress induce ROS, UPR, DDR, protein aggregation and toxic metabolite accumulation. These stress signals converge on key intracellular processes, including caspase activation, autophagosome formation, cell membrane rupture and the expression of senescence‐associated markers such as p21 and p16. Imbalanced cell fate decisions further promote apoptosis, autophagy, cellular senescence, pyroptosis and necroptosis. Senescent cells secrete SASP factors, while inflammatory cell death releases DAMPs, including HMGB1, ATP and IL‐1β. These mediators amplify inflammation, recruit immune cells and disturb vascular and stromal homeostasis, ultimately transforming a normal microenvironment into a collapsed pathological microenvironment.

As the cornerstone of physiological defense, the hematologic and immune systems play pivotal roles in resisting pathogens and maintaining homeostasis. The biomarkers involved in these two systems are characterized by high cellular specificity and molecular diversity, providing important evidence for exploring disease pathogenesis and precision treatment. This section focuses on major blood and immune system diseases, such as leukemia, lymphoma, and autoimmune diseases, to systematically explore the entire process from the differentiation of hematopoietic stem cells to the regulation of the immune response. Finally, an integrated research framework covering the “hematopoiesis–immunity” axis is constructed (Table S81 and Figure 6).

#### Hierarchical network of blood cell differentiation and functional markers

CD molecules, key membrane antigens expressed during leukocyte differentiation, are essential for the identification, isolation and functional characterization of immune cells. The dynamic ratio of CD4^+^ to CD8^+^ T cells reflects host immune homeostasis and exhibits characteristic changes under various pathological conditions, such as autoimmune diseases and immunodeficiency disorders. Therefore, this ratio is an important biomarker for evaluating immune status [1081, 1082]. *CD34*, a canonical antigen expressed on hematopoietic stem and progenitor cells, plays an indispensable role in clinical practice; it serves as a crucial biomarker for the diagnosis, classification and minimal residual disease (MRD) monitoring of leukemia and serves as an important indicator for assessing treatment response and hematopoietic reconstitution [1083]. In addition, surface antigens such as *CD20* and *CD19* not only constitute the basis for B‐cell immunophenotyping but also serve as key molecular targets for the treatment of various hematologic malignancies. Among them, *CD19* represents the most established target for chimeric antigen receptor T cell (CAR‐T) therapy [1084]. During myeloid differentiation, markers such as *CD13*, *CD33* and *CD14* are sequentially expressed, outlining the developmental trajectory from hematopoietic stem cells to mature granulocytes and monocytes. This characterization is crucial for the diagnosis, subtyping and longitudinal monitoring of myeloid leukemias [1085].

#### Immunoglobulins and antibody lineage markers

Serum protein electrophoresis and immunofixation electrophoresis are the main techniques for detecting monoclonal immunoglobulins in serum or urine. In SLE, ANAs are often used as screening markers, but anti‐double‐stranded DNA (anti‐dsDNA) antibodies have higher disease specificity; changes in anti‐dsDNA titers are related to renal involvement and disease activity, making them important indicators for monitoring [1086, 1087]. In rheumatoid arthritis (RA), rheumatoid factor (RF) and anti‐cyclic citrullinated peptide (anti‐CCP) antibodies are important diagnostic markers. RF has high sensitivity but low specificity, whereas anti‐CCP antibodies have high diagnostic specificity. The combined detection of these two markers can improve diagnostic accuracy, and their titers are often associated with joint destruction and disease activity [1088]. Anti‐cardiolipin antibodies and anti‐β2 glycoprotein I (AB2GP1) antibodies are important immunological markers for APS. Among them, medium‐to‐high‐titer IgG/IgM antibodies have higher diagnostic specificity. These markers are highly important for evaluating patient recurrence risk and guiding long‐term anticoagulation therapy and pregnancy management [1086].

#### Cytokine and inflammatory marker networks

The dynamic equilibrium between proinflammatory and anti‐inflammatory cytokines plays a crucial regulatory role in the pathogenesis and progression of inflammatory and autoimmune diseases. Proinflammatory cytokines, such as TNF‐α, IL‐6, and IL‐1β, are often significantly elevated in serum or tissue during systemic inflammatory responses and in various autoimmune diseases, including rheumatoid arthritis. These cytokines not only serve as key biomarkers of disease activity but also represent important therapeutic targets for multiple biologics, such as TNF‐α antagonists and IL‐6 receptor antagonists [1089]. Conversely, anti‐inflammatory cytokines, primarily IL‐10 and TGF‐β, play an indispensable role in maintaining immune homeostasis and inducing peripheral tolerance. An imbalance in proinflammatory and anti‐inflammatory cytokine expression is associated with the onset of autoimmune diseases [1090]. In immunotherapy, particularly in CAR‐T cell therapy, the dynamic monitoring of cytokines is critical because of the potential life‐threatening complication of cytokine release syndrome (CRS), also known as a “cytokine storm.” During this process, the levels of cytokines such as IL‐6 and interferon‐gamma (IFN‐γ) increase sharply, correlating closely with the severity of clinical manifestations. Therefore, timely monitoring and targeted intervention have become essential clinical strategies for managing such complications [1091].

#### Immune checkpoints and regulatory markers

Among the key mechanisms of tumor immune evasion, the PD‐1/PD‐L1 pathway serves as a central regulatory mechanism [1092]. This pathway has emerged as one of the most critical targets in contemporary tumor immunotherapy because of its role in inhibiting T cell function and promoting immune tolerance. PD‐1/PD‐L1 inhibitors have shown significant efficacy in the treatment of various solid tumors and hematologic malignancies. Conversely, cytotoxic T‐lymphocyte‐associated protein 4 (CTLA‐4), an early‐acting immune checkpoint molecule, is primarily negatively regulated during the initiation phase of T cell activation to prevent excessive immune responses. By blocking this inhibitory signal, CTLA‐4 inhibitors enhance T cell activation and proliferation, achieving remarkable therapeutic outcomes in the treatment of malignant tumors such as melanoma. These inhibitors are often used in combination with PD‐1/PD‐L1 inhibitors to amplify antitumor effects [1093]. Regulatory T cells (Tregs) play crucial roles in maintaining immune homeostasis and suppressing autoimmune responses. Their specific transcription factor FoxP3 not only serves as a primary marker for Treg cell differentiation and function but also acts as a critical biomarker for evaluating the tumor immune microenvironment and predicting immune therapy responses [1094].

#### Hematopoietic and immune genetic markers

In the molecular diagnosis and targeted therapy of hematologic malignancies, the *BCR‐ABL* fusion gene is a hallmark molecular marker for CML; it not only offers vital evidence for the diagnosis and classification of this disease but also serves as a primary therapeutic target for tyrosine kinase inhibitors (TKIs), such as imatinib. The quantitative monitoring of this fusion gene is crucial for assessing treatment efficacy and predicting disease progression [1095]. The *JAK2* V617F mutation is highly specific to myeloproliferative neoplasms (MPNs) and is frequently observed in conditions such as polycythemia vera, essential thrombocythemia, and essential myelofibrosis. Identifying this mutation provides essential molecular evidence for MPN diagnosis and lays the theoretical groundwork for targeted treatments, including JAK2 inhibitors, for these diseases [1096]. Evaluating lymphocyte clonality through the detection of immunoglobulin heavy chain (IgH) and T cell receptor (TCR) gene rearrangements is instrumental in differentiating reactive lymphocyte proliferation from malignant lymphoma [1096]. This method furnishes critical experimental data for the molecular subtyping of B‐cell or T‐cell lymphoma, monitoring minimal residual disease, and assessing recurrence risk.

### Blood and immune systems as the core hub of systemic defense and regulation

The blood and immune systems form a crucial regulatory network that maintains homeostasis within an organism. Through their extensive circulatory and lymphatic networks, which span the entire body, these systems interconnect various organs, creating a dynamic system for information exchange and functional synergy. In this process, the blood and immune systems not only act as sentinels for the body, constantly monitoring and responding to external threats and internal abnormalities, but also serve as critical regulatory centers. They integrate, transmit, and respond to biological signals from different systems, thereby facilitating the systematic coordination and precise regulation of physiological functions and pathological states (Table S82).

#### Interacting network of the immune–nervous–endocrine axis

The neuro–immune–endocrine network represents an intricately integrated physiological regulatory system, facilitating dynamic interactions between systems via a myriad of signaling molecules. Neuroendocrine factors, including cortisol, catecholamines, and acetylcholine, directly modulate the proliferation, differentiation, and functionality of immune cells by binding to their respective surface receptors [1097]. For example, under stress conditions, activation of the HPA axis results in the release of glucocorticoids. Glucocorticoids can induce T cell apoptosis and broadly inhibit the synthesis and secretion of proinflammatory cytokines, such as interleukin‐2 (IL‐2) and TNF‐α, thereby exerting significant immunosuppressive effects [1098]. Concurrently, the immune system reciprocates this regulation of neuroendocrine functions through cytokine signaling: inflammatory mediators such as IL‐1β, IL‐6, and TNF‐α, which are secreted by activated immune cells, can penetrate the central nervous system and influence brain regions, notably the hypothalamus and hippocampus, thus modulating neuroendocrine feedback and neurotransmitter metabolism [1099]. This perpetual bidirectional communication forms a sophisticated neuro–immune–endocrine regulatory axis, playing a pivotal role in the initiation and progression of stress, infection, autoimmune diseases, and psychiatric disorders.

#### Mutual regulation of the immune–metabolic axis

The immune and metabolic systems are interconnected through intricate signaling networks, establishing the “immune–metabolic axis.” The functional status of immune cells is intrinsically linked to their metabolic profiles, with metabolic reprogramming being pivotal in immune responses. For example, during T cell differentiation, the equilibrium between glycolysis and oxidative phosphorylation directly influences the functional trajectory of T cells. For their functionality, proinflammatory helper T cells (Th17) predominantly rely on glycolysis, whereas regulatory T cells (Treg) chiefly depend on fatty acid oxidation [1100]. Beyond energy metabolism pathways, intermediates such as lactate and succinate act as signaling entities, modulating the differentiation and function of immune cells by affecting epigenetic modifications and signaling cascades [1101]. This intricate network becomes especially evident under pathological conditions. In obesity‐induced chronic inflammation, adipose tissue macrophages become activated, perpetually releasing proinflammatory agents. These agents can disrupt insulin signaling, leading to systemic insulin resistance, thus underscoring the crucial role of immunometabolic imbalance in the etiology of metabolic disorders [1102].

#### Symbiotic relationship of the mucosal immune–microbial axis

The gut microbiota maintains intestinal homeostasis and systemic immune function through dynamic crosstalk with the intestinal mucosal immune system. This interaction is mediated primarily by pattern recognition receptors, including Toll‐like receptors (TLRs) and NOD‐like receptors (NODs), which modulate epithelial barrier integrity, immune cell differentiation, and cytokine secretion [1103]. Microbial metabolites also exert significant immunoregulatory effects, with SCFAs such as butyrate and propionate not only serving as crucial energy substrates for intestinal epithelial cells but also promoting Treg cell differentiation and function while suppressing excessive inflammatory responses through epigenetic modifications and G protein‐coupled receptor signaling pathways [1104, 1105]. Concurrently, the intestinal mucosal immune system actively sculpts microbial composition and spatial organization via mechanisms such as secretory immunoglobulin A (sIgA), thereby preserving mucosal barrier integrity [1106]. Disruption of this complex reciprocal network has been implicated in the pathogenesis of various intestinal inflammatory conditions and immune dysregulation.

#### Dynamic process of tumor immunoediting

The theory of tumor immunoediting describes three dynamic phases that evolve between the immune system and tumors—elimination, equilibrium, and escape. In the elimination phase, immune cells such as natural killer (NK) cells and cytotoxic T lymphocytes (CTLs) work in concert to recognize and destroy nascent tumor cells [1107]. As the process advances to the equilibrium phase, the immune system imposes continuous selective pressure on the tumor, allowing for the survival of tumor cell clones with low immunogenicity or resistance to immune attack [1108]. During the escape phase, tumor cells eventually evade immune surveillance through various mechanisms, including the upregulation of immune checkpoint molecule (e.g., PD‐L1) expression, the recruitment of immunosuppressive cells (e.g., Tregs and myeloid‐derived suppressor cells), and the downregulation of major histocompatibility complex (MHC) expression, leading to unchecked growth and metastasis [1109, 1110]. A comprehensive understanding of this dynamic process offers a crucial theoretical basis for the development of immunotherapeutic strategies aimed at different stages of the disease.

#### Rejection and tolerance markers in transplantation immunology

**FIGURE 7:**
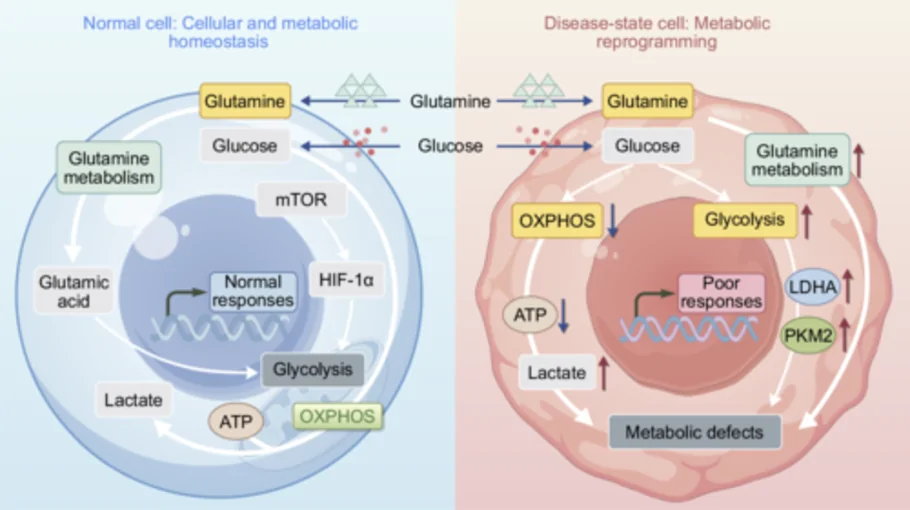
Schematic of cellular metabolic reprogramming and energy crisis. This figure illustrates the metabolic transition from cellular homeostasis to disease‐associated metabolic reprogramming. In normal cells, glucose and glutamine metabolism are coordinated to maintain balanced energy production and biosynthesis. Glutamine is converted into glutamic acid and supports mitochondrial OXPHOS, while glucose metabolism contributes to glycolysis and ATP production. Key regulators such as mTOR and HIF‐1α help maintain adaptive metabolic responses and normal cellular function. In disease‐state cells, metabolic balance is disrupted and cells undergo metabolic reprogramming. Glutamine uptake and glutamine metabolism are increased, glycolysis is enhanced, and lactate accumulation is elevated, accompanied by upregulation of glycolytic enzymes such as LDHA and PKM2. In contrast, OXPHOS and ATP production are reduced, resulting in mitochondrial dysfunction and an energy crisis. These metabolic defects weaken cellular responses and promote pathological progression.

In the context of transplant immunology, a variety of biomarkers are employed to gauge the risk of rejection and to monitor the status of immune tolerance. At the humoral immune level, donor‐specific antibodies (DSAs) serve as crucial predictors of antibody‐mediated rejection. Cellular immune compatibility can be assessed through in vitro assays such as the mixed lymphocyte reaction (MLR) [1111]. Regulatory cell populations, including regulatory T cells (Tregs) and myeloid‐derived suppressor cells (MDSCs), play pivotal roles in the induction and maintenance of transplant tolerance. For the dynamic monitoring of posttransplant immune status, functional immunological assays are highly valuable. For example, the ATP release assay provides a quantitative measure of CD4^+^ T cell activation, thereby facilitating individualized adjustments to immunosuppressive regimens [1112]. Furthermore, donor‐derived cfDNA (dd‐cfDNA) present in peripheral blood can serve as a biomarker for tissue injury indicative of rejection. Additionally, specific peripheral blood gene expression profiles, such as AlloMap®, can be utilized to monitor the risk of rejection following heart transplantation (Figure 7) [1113].

## INTEGRATION OF BIOMARKERS ACROSS ORGAN SYSTEMS

The preceding chapters have focused primarily on organizing and establishing a comprehensive knowledge base of diseases and biomarkers, effectively creating a “database” and an “encyclopedia.” By classifying and integrating a diverse range of biomarkers from a cross‐organ perspective, this knowledge framework can function as a “navigation system.” This system transforms information into actionable clinical strategies and intervention pathways.

**FIGURE 8:**
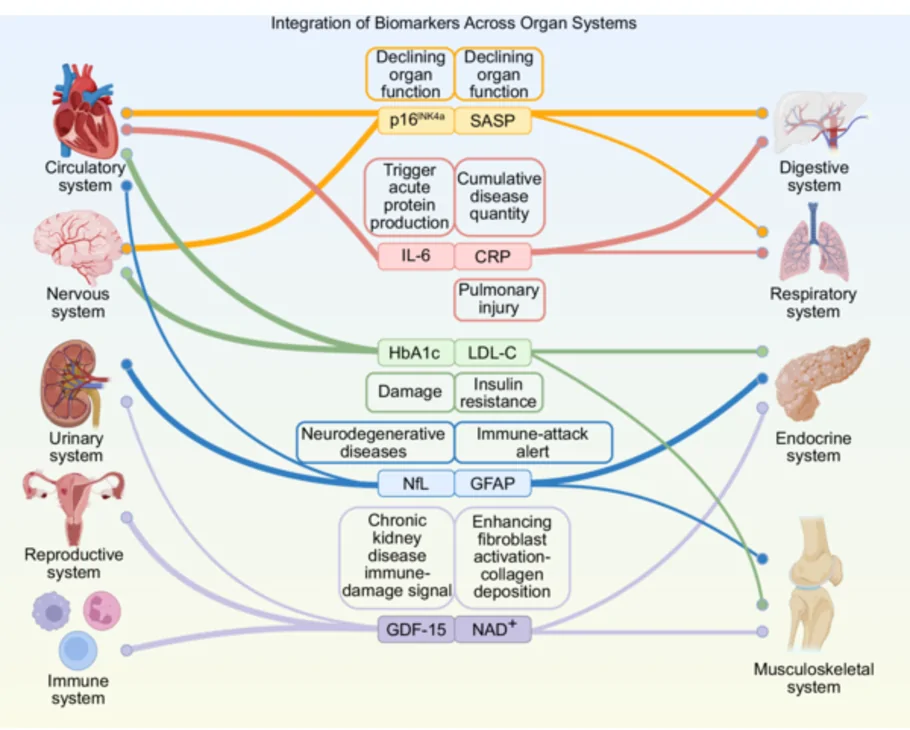
Integration of biomarkers across organ systems. This schematic summarizes shared and system‐specific biomarkers that connect multiple human organ systems, including the circulatory, nervous, urinary, reproductive, immune, digestive, respiratory, endocrine and musculoskeletal systems. Curved lines indicate cross‐organ associations between representative biomarkers and pathological processes. Senescence‐related markers, such as p16^INK4a^ and the SASP, are linked to declining organ function. Inflammatory biomarkers, including IL‐6 and CRP, reflect acute‐phase protein production, cumulative disease burden and pulmonary injury. Metabolic biomarkers, such as HbA1c and LDL‐C, are associated with tissue damage and insulin resistance across metabolic and vascular disorders. Neurological and immune‐related biomarkers, including NfL and GFAP, indicate neurodegenerative processes and immune‐attack alerts. Aging‐ and injury‐associated markers, such as GDF‐15 and NAD^+^, are connected with chronic kidney disease, immune‐damage signaling, fibroblast activation and collagen deposition.

With the acceleration of population aging, multimorbidity rather than comorbidity has become a central challenge in modern clinical practice and public health systems [1114]. This trend is driving a transition in disease research and management, from a “single‐disease” perspective to an “overall health” perspective. Chronic disorders, including cardiovascular, metabolic, and neurodegenerative diseases, do not develop in isolation but codevelop as components of a multimorbidity network through shared mechanisms such as chronic low‐grade inflammation, metabolic imbalance, and vascular immune interactions [1115]. Addressing this complexity requires moving beyond organ‐specific silos, integrating biomarkers at the system level, and constructing a “biomarker network” that can be dynamically reprogrammed throughout disease progression. In such a network, certain key molecules may serve as hubs across multiple organ systems, thereby providing actionable routes for multimorbidity risk prediction, early warning, and stratified intervention (Figure 8).

Within a cross‐organ biomarker framework, the continuum from health to subhealth and ultimately to overt disease progression can be delineated by dynamic changes in biomarkers. The healthy state is generally characterized by biomarker levels maintained under physiological homeostasis, such as low basal tone of inflammation, metabolic indicators within target ranges, and normal blood pressure and heart rate. Biomarkers at this stage show relatively stable fluctuations that reflect preserved physiological function and systemic resilience [1116]. When an individual enters the subhealthy state, biomarkers begin to deviate mildly without reaching clinical diagnostic thresholds, and cross‐organ biomarkers often exhibit coordinated low‐grade abnormalities. At this stage, constitutional heterogeneity gradually becomes evident: under identical external stressors or lifestyle interventions, individuals may differ in the magnitude of biomarker deviation and capacity for recovery, which reflects interindividual variation in metabolic resilience, immune responsiveness, and neuroendocrine regulation. Once such deviations continue to accumulate and manifest across multiple organ systems, the disease progresses. Persistent abnormalities in core hub biomarkers and effector node biomarkers at this point form a network of systemic injury that can be used for disease subtyping and risk assessment. Together with constitutional characteristics, these biomarker patterns may also inform disease susceptibility and intervention responsiveness. The same degree of biomarker abnormality may lead to distinct patterns of organ damage or different rates of disease progression in individuals with different constitutional backgrounds, thereby providing a basis for personalized intervention strategies and follow‐up planning.

### Network‐based identification and hierarchical construction of hub biomarkers

“Hub biomarkers” are defined as biomolecules or physiological indicators that occupy central positions within cross‐system pathological networks, serving as amplifiers of systemic signals, bridges between organ systems, or sentinels of localized injury. A systematic classification of cross‐organ biomarkers represents a crucial step toward addressing complex disease networks and moving from “knowledge enumeration” to “system navigation”. This framework is not simply a conceptual list but rather a hierarchical interpretation model based on systems biology that facilitates the understanding of multiorgan interactions and guides precision clinical practice. Its core significance lies in organizing vast heterogeneous biomarker information into a layered network with both biological logic and clinical maneuverability. The classification system is not based on subjective speculation but rather on the biological scope of action and radius of signal propagation of the biomarkers. On the basis of these guiding principles, we propose a three‐tier framework consisting of core hubs, regional hubs, and effector nodes. Core hub biomarkers have broad regulatory and amplifying effects and can induce concordant pathophysiological changes across multiple organ systems, making them particularly suitable for characterizing systemic driving forces and overall risk levels in the context of multimorbidity. Hub biomarkers reside at interfaces between systems and reflect whether key bridging processes are activated, such as spillover of metabolic burden to the vascular endothelium and microcirculation or propagation of immune inflammation to the nervous system. Their value lies in indicating the directionality of risk spread and identifying potentially vulnerable organs. Effector‐node biomarkers are more organ specific and capture local parenchymal injury, functional decline, or pressure overload, thereby directly identifying affected organs and severity of involvement. Interpretation of effector nodes in the context of hub signals helps distinguish isolated single‐organ events from systemic cascade responses and reduces misclassification. This framework jointly considers the breadth of action, regulatory hierarchy, signal propagation characteristics, clinical relevance, and topological position within a network with the aim of organizing heterogeneous biomarker information into an interpretable hierarchical network that separates systemic drivers from local effects and provides a structured basis for understanding multimorbidity mechanisms and enabling precision clinical navigation.

Notably, the biomarker interaction network proposed here is a conceptual framework derived from an extensive literature review and expert knowledge. It was not algorithmically inferred from raw data to discover previously unknown associations; rather, it systematically integrates established biological mechanisms, pathway connections, and clinically validated associations reported in previous studies. The value of this network lies in its ability to provide a panoramic and structured framework for knowledge integration, thereby visualizing fragmented cross‐system and cross‐disease relationships. Moreover, this framework explicitly supports testable scientific hypotheses regarding hierarchical biomarker relationships on the basis of current knowledge. It also provides a biologically informed prior framework for subsequent data‐driven multiomics network analysis, machine learning model development, and longitudinal cohort validation.

#### Core hub biomarkers

Core hub biomarkers are biomolecules or physiological indicators that exert broad systemic regulatory effects such that their abnormalities can simultaneously reflect and potentially drive pathophysiological processes across multiple organ systems. They function as “amplifiers” and “master regulators” of the systemic disease state. Their defining characteristics include global influence, cumulative and relative stability, and upstream driving capacity. Clinically, they are useful for assessing an individual's baseline systemic risk and identifying shared drivers of multimorbidity, making them prime targets for preventive intervention and whole‐body health management. Representative core hub biomarkers include the inflammatory markers IL‐6, CRP, TNF‐α and IL‐1β and the metabolic marker HbA1c. The deeper mechanistic basis for their role as ‘hub cores’ is that rather than being merely consequences of specific diseases, they occupy upstream positions within complex etiological networks and act as central signal amplifiers and common pathological pathways that drive downstream abnormalities in multiple systems through highly conserved cross‐tissue signaling cascades. For example, IL‐6 activates pathways such as the JAK/STAT pathway, which directly regulate immune cell function and also induces hepatic production of CRP while affecting endothelial function, insulin sensitivity and glial cell activity, thereby amplifying local inflammation into a chronic inflammatory burden spanning cardiovascular, metabolic and neural systems [1117, 1118]. By activating key transcription factors such as NF‐κB, TNF‐α and IL‐1β are broadly involved in the shared pathways underlying insulin resistance, atherosclerosis, and neuroinflammation [1119]. From a metabolic perspective, HbA1c, as a stable integrated indicator of chronic hyperglycemia exposure, has hub properties because the chronic glycometabolic stress it represents can cause widespread and persistent damage to the vascular endothelium, glomerular filtration barrier, neurons, and even immune cell function through mechanisms such as advanced glycation end product formation, oxidative stress amplification, and mitochondrial dysfunction [1120, 1121]. Therefore, abnormal levels of these core hub biomarkers essentially represent direct measurements of systemic pathophysiological states that drive multiorgan degeneration and disease, such as chronic low‐grade inflammation and persistent metabolic stress. These changes often precede or accompany downstream organ‐specific injury markers, which explains why they are strongly associated with cross‐system morbidity risk, functional decline, and all‐cause mortality and constitute primary logical targets for systemic risk intervention and overall health management.

#### Regional hub biomarkers

Regional hub biomarkers are those that establish functional links between specific organ systems and reflect whether key cross‐system interactive processes have been activated. They act as “bridges” and “interfaces” in pathophysiological networks and are characterized by interfacial positioning, process specificity, and directional indication. Clinically, they help to interpret specific multimorbidity phenotypes, predict the direction of complication or pathological risk transmission, and provide precise intervention targets for interrupting abnormal signal transmission between organ systems. In neurological disorders, representative regional hub biomarkers include neurofilament light chain (NfL) and GFAP. Their status as regional hubs lies in their role as neither systemic driving factors nor terminal injury products; rather, they are process‐associated signaling molecules released upon activation of specific anatomical–functional axes, such as the neuromuscular axis or neuroimmune axis, and can be detected in body fluids. As such, they accurately reflect pathological stress or injury that is propagating across conventional organ boundaries. For example, NfL is a major structural component of the neuronal axonal cytoskeleton. When axons in the central or peripheral nervous system are damaged by degeneration, inflammation, or trauma, NfL is released into the extracellular space and subsequently enters the cerebrospinal fluid and peripheral circulation [1122]. Its hub property lies in the fact that it not only serves as a direct readout of axonal injury within the nervous system but also acts as a “signal messenger,” conveying the status of the nervous system to the whole body because of its stable presence in blood. Elevated plasma NfL levels were not only associated with brain atrophy and cognitive decline but also linearly negatively correlated with reduced grip strength and muscle wasting, providing empirical evidence for coupled dysfunction along the neuromuscular axis in multimorbidity. NfL thus represents a core regional hub marker quantifying the degeneration of this cross‐system connection. Another exemplar marker is GFAP, which is specifically expressed by astrocytes in the central nervous system. Astrocytes become markedly activated and upregulate GFAP expression when the brain is subjected to neuroinflammation, neurodegeneration, or vascular injury, some of which may reach the peripheral blood through disruption of the blood–brain barrier or cerebrospinal fluid circulation [1123]. Therefore, peripheral blood GFAP levels serve as a sensitive indicator of neuroimmune axis mobilization. GFAP not only reflects astrocytic activation but also links pathological processes in the central nervous system, such as Aβ deposition and tauopathy, to peripheral immune‐inflammatory states. In the Alzheimer's disease continuum, increased plasma GFAP is significantly associated with increased amyloid plaque burden in the brain and subsequent cognitive decline, suggesting that astrocytes, as immunological interface cells of the brain, may influence peripheral immune responses through the release of GFAP and related molecules upon sensing central pathologic signals or alternatively reflect the impact of systemic inflammation on the CNS [1124]. Thus, regional hub biomarkers such as NfL and GFAP can track the directionality of pathological signal flow between organs by quantifying specific “axis” connectivity and identifying which organ systems are undergoing coordinated degeneration or mutual exacerbation, thereby enabling precision intervention along specific pathological transmission routes.

#### Effector‐node biomarkers

Effector‐node biomarkers are those that directly and specifically reflect acute injury, chronic functional decline or abnormal pressure overload in a specific organ or tissue. They represent the “terminal readout” and “local sensor” of pathophysiological cascade responses. Their key characteristics include high organ specificity, direct quantifiability of injury or function and sensitivity to upstream pathophysiological network regulation. Clinically, they underpin disease diagnosis, severity stratification, identification of acute events and therapeutic monitoring. More importantly, when interpreted together with core hub and regional hub biomarkers, they can effectively differentiate isolated organ events from target‐organ manifestations within systemic pathophysiological networks, thereby guiding fundamentally different treatment strategies. Representative effector‐node biomarkers include cardiac troponin, N‐terminal pro‐B‐type natriuretic peptide (NT‐proBNP), creatinine/eGFR and liver transaminases. These markers are either direct products released or altered as a result of stress, injury or death of specific cell types. Circulating concentrations generally show dose–response relationships with the severity of target‐organ pathology, but their occurrence and magnitude are profoundly influenced by upstream systemic pathophysiological status reflected by core and regional hub biomarkers. For example, cardiac troponin, a characteristic component of cardiomyocyte myofibrils, leaks into the circulation when cardiomyocyte membrane integrity is compromised, making it the gold standard biomarker for myocardial necrosis [1125]. Although highly specific to the heart, its broader pathophysiological relevance lies in the fact that cTn may increase under conditions such as sepsis, severe systemic inflammation or extreme metabolic stress even without coronary obstruction because systemic inflammatory mediators can induce myocardial stunning, microcirculatory dysfunctions or mitochondrial depression. In these settings, an increased cTn does not represent an isolated cardiac event but rather the response of the heart as an effector node and functional core of the circulation to a systemic inflammatory or stress network insult. NT‐proBNP is synthesized and released predominantly by ventricular cardiomyocytes in response to augmented wall stress and thus provides a direct measure of pressure or volume overload on the heart [1126]. Serum creatinine levels and eGFRs directly reflect glomerular filtration function and are core quantitative markers of renal function [1127]. Alanine aminotransferase and aspartate aminotransferase are located predominantly within the hepatocyte cytosol and are released into the circulation upon hepatocellular injury or necrosis, making them sensitive markers of parenchymal liver damage [1128]. These effector nodes share a common characteristic: they provide near real‐time, anatomically localized information on the status of specific organs and are therefore indispensable for clinical decision‐making. However, their interpretation must always be contextualized within the systemic background defined by hub biomarkers. A modest increase in cTn or creatinine is pathologically quite different when it occurs in the presence of marked systemic inflammation (high IL‐6 and CRP) than when core hub biomarkers remain normal. The former suggests that the organ is a vulnerable target within a systemic pathophysiologic network and that treatment should focus on upstream drivers; the latter more likely indicates primary or focal disease in that organ. Effector node biomarkers thus serve as critical coordinates for precision “navigation,” but accurate clinical interpretation depends on embedding them within the global pathophysiologic map delineated by hub biomarkers.

#### Biomarker combinations

Even broadly informative single‐category biomarkers, such as central hub biomarkers, have inherent limitations in complex multimorbidity settings. For example, the neurofilament light chain, although a sensitive indicator of neuronal axonal injury, cannot be used on its own to distinguish whether the underlying damage is due to Alzheimer's disease, vascular cognitive impairment, or other neurodegenerative disorders [1129]. Similarly, the levels of IL‐6 and CRP, which are central hub markers of systemic inflammation, effectively indicate that an individual is experiencing a chronic inflammatory burden associated with chronic illness but cannot specify whether the primary driver is metabolic dysregulation, autoimmune activation, or an underlying neoplastic process. Accordingly, the core operational logic of this navigation framework does not depend on any single biomarker. Instead, it promotes a hierarchical and progressive combination biomarker strategy to enable the transition from generalized risk warning to precise etiologic discrimination and target organ localization.

In this strategy, biomarkers at different hierarchical levels play interlocking roles. Core hub biomarkers serve as the “first alarm” and “quantitative baseline”, with their abnormalities triggering evaluation of systemic status and multimorbidity risk. Regional hub biomarkers then act as “directional indicators of pathological transmission” helping to identify the specific organ axes involved. Finally, highly specific effector‐node biomarkers provide decisive evidence for etiological confirmation and localization, clarifying the exact pathological type of injury and target organs involved.

This integrative logic is particularly important in the differential diagnosis and management of cross‐order diseases. Taking neurodegenerative disorders as an example, when plasma NfL is strongly elevated, reflecting widespread axonal injury, the navigation system can guide subsequent combination testing. If combined testing reveals both a decreased plasma Aβ42/40 ratio and increased p‐tau181, which are Alzheimer's disease‐specific effector nodes, this strongly suggests AD‐driven pathology [1130]. If the α‐synuclein seed amplification assay is positive, a synucleinopathy is indicated. If abnormal vascular‐inflammatory hub markers such as hs‐CRP coexist with white matter hyperintensities on MRI, then the contribution from vascular pathology should also be considered. In this context, NfL provides quantitative information on lesion load, while subsequent disease‐specific biomarker combinations precisely reveal the etiological nature of the lesions.

Another instructive example concerns the attribution of causality in multimorbidity. In a patient with heart failure and coexisting renal dysfunction, an isolated increase in NT‐proBNP (a cardiac effector node) or creatinine (a renal effector node) is insufficient to determine which was the initial pathogenic driver [1131]. Therefore, the navigation logic requires that HbA1c (which represents chronic metabolic burden as a central hub), IL‐6 (which represents inflammatory burden as a central hub), and potential regional hub biomarkers for cardiorenal crosstalk, such as FGF‐23, should be assessed simultaneously. If HbA1c is the dominant abnormality and is accompanied by concordant abnormalities in both cardiac and renal effector nodes, then longstanding dysregulation of metabolism is likely the shared upstream driver. If IL‐6 is the dominant abnormality, then chronic inflammatory stress should be suspected as the major factor mediating coordinated injury to the heart and kidney. If FGF‐23 is markedly abnormal, this highlights the pathological endocrine feedback axis linking the heart and kidney. Such combinatorial analyses effectively distinguish the relative contributions of competing pathophysiologic mechanisms and directly inform therapeutic prioritization.

In summary, the clinical utility of the proposed “human biomarker navigator” is fundamentally dependent on the dynamic and hierarchical integration of core hub, regional hub, and effector node biomarkers. Core hubs provide panoramic risk maps and early warning coordinates, while targeted combinations of biomarkers guided by these coordinates delineate routes toward precise etiology and therapeutic targets. This integrative logic connects early risk detection with systematic health management. It provides an operational blueprint for the present framework to address multimorbidity era complexity and to reorient from disease treatment toward health management.

#### Network‐based rationale for multidimensional mechanistic interconnectivity

Biomarker network construction relies on multilayered mechanisms of interconnectivity. These are interwoven across spatial distribution, temporal dynamics and functional coordination to form a hierarchically structured regulatory network with feedback properties. At the spatial level, blood circulation distributes metabolites, cytokines, hormones and other signaling molecules throughout the body, allowing for local perturbations to spill over into peripherally detectable readouts. Barrier structures determine the intensity and threshold for cross‐compartmental transmission. For example, changes in blood–brain barrier integrity can influence the extent to which central nervous system‐derived molecules enter the periphery while also altering the range over which peripheral inflammatory and metabolic signals act upon the central nervous system [1132]. Lymphatic drainage and lymph node networks provide routes for immune cell migration and antigen delivery that facilitate progression from localized inflammation to a systemic inflammatory milieu under certain conditions. At the temporal level, network signals often emerge in a staged manner [1133]. Upstream metabolic burden or stress responses generally show longitudinal directional deviation first, followed by progressive amplification of inflammatory and endothelium‐related signals. If the systemic load continues to accumulate, downstream organ‐specific effector nodes are more likely to become chronically dysfunctional and ultimately indicate damage or functional decline. At the functional level, biomarkers may interact synergistically or antagonistically. Proinflammatory pathways can form amplification loops, whereas anti‐inflammatory and metabolically protective signals can constrain their expansion and facilitate recovery. Metabolism and immunity mutually shape each other: inflammation can reduce insulin sensitivity and alter lipid handling, whereas metabolic dysregulation can in turn increase the inflammatory tone. Incorporating these three mechanistic dimensions into the interpretative framework elevates cross‐organ associations from mere statistical correlations to mechanistically interpretable evidence, thereby providing a basis for subsequent model development and dynamic monitoring.

#### Spatially organized pathway architecture

Anatomical and barrier pathways provide the physical basis for cross‐organ biomarker correlations. The blood–brain barrier is a critical interface between the CNS and periphery, and changes in its permeability can directly influence the extent to which CNS‐derived molecules enter the peripheral circulation. Once blood–brain barrier integrity declines or transport capacity is altered, the threshold at which brain‐derived proteins and inflammatory mediators enter the circulation changes accordingly, with direct effects on the correlation between circulating blood biomarkers and central pathology. Therefore, the status of the blood–brain barrier should be incorporated as an important contextual factor when cross‐organ biomarker relationships are interpreted [1134]. In traumatic brain injury, for example, plasma or serum GFAP and NfL levels may increase early after injury, and these levels are associated with structural brain damage and a risk of subsequent poor outcomes [1135]. The lymphatic vasculature, in turn, provides a conduit for the systemic trafficking of immune cells and mediators, enabling the rapid translation of local inflammation into a whole‐body response [1133]. In models of sepsis‐induced acute respiratory distress syndrome, enhanced pulmonary lymphatic drainage promotes the trafficking of inflammatory cells to draining lymph nodes and accelerates clearance of lung edema fluid, thereby reducing lung inflammation and facilitating its resolution. These findings suggest that the efficiency of pulmonary lymphatic drainage itself is a key limiting step in determining whether localized inflammation persists and expands into a systemic response [1136].

#### Dynamic evolution across the temporal dimension

Biomarker networks exhibit clear temporal characteristics, with signals at different hierarchical levels often appearing in sequence and becoming increasingly manifested as the disease progresses. In early stages, alterations in metabolic burden are frequently detected first. For instance, a progressive increase in HbA1c may persist for an extended period before clinical intervention is initiated, indicating that chronic glycemic exposure can accumulate and enter a detectable window prior to overt symptom onset [1137]. As metabolic dysregulation continues, the organism gradually transitions to a state of chronic low‐grade inflammation. Studies have demonstrated that the levels of proinflammatory cytokines such as IL‐6 and IL‐1β begin to increase even before the onset of overt diabetes and are associated with an increased future risk of T2D [1138]. Concurrently, associations between IL‐6 and adverse cardiovascular outcomes have been consistently observed across various cohorts, suggesting that inflammatory signaling becomes progressively more prominent as the disease progresses [1139, 1140]. Persistent inflammatory signaling is not merely a bystander phenomenon; it may actively diminish compensatory capacity and lower injury thresholds in multiple organs through mechanisms such as endothelial dysfunction, microcirculatory impairment, elevated oxidative stress, and immunometabolic reprogramming. This process subsequently promotes abnormalities in more organ‐specific effector nodes, including markers of myocardial injury, cardiac pressure overload, or renal dysfunction. Such temporal progression offers an actionable entry point for risk stratification: when inflammatory biomarkers remain persistently elevated but organ‐specific markers are still within subclinical ranges, intensified assessment and intervention may help prevent progression toward overt organ damage or multiorgan involvement, thereby enabling early warning of disease.

#### Functional synergy and coordinated regulation

Different biomarkers achieve functional integration through complex synergistic and antagonistic relationships. TNF‐α rapidly initiates an inflammatory transcriptional program centered on NF‐κB, while IL‐6 mainly promotes the acute phase response and inflammation‐related gene expression via the JAK/STAT3 pathway. IL‐6 and TNF‐α synergistically amplify the inflammatory response that drives inflammation to become more intense and longer lasting [1141, 1142]. In contrast, adiponectin functions as an important negative regulatory signal that restrains excessive expansion of inflammation. Studies have shown that adiponectin can suppress IκB‐associated processes and NF‐κB activation induced by TNF‐α, reduce endothelial adhesion molecule expression, and alleviate inflammatory cell recruitment. Adiponectin also inhibits lipopolysaccharide‐induced NF‐κB activation and downregulates IL‐6 expression in adipocytes and related models [1143]. Overall, proinflammatory amplification and anti‐inflammatory suppression form a functional counterregulatory system that allows inflammation to be sufficiently activated under stress but still capable of being constrained and resolved at an appropriate time to maintain systemic homeostasis.

#### Core pathological node networks

Within the cross‐organ biomarker framework, systemic inflammation, metabolic reprogramming and oxidative stress are core hub nodes underlying chronic multimorbidity. These nodes affect multiple tissues and organs across systems through interconnected molecular signaling pathways. The inflammatory network is centered on IL‐6 and CRP as core hub biomarkers that promote inflammatory gene expression via JAK/STAT3 signaling while also activating complement and endothelial adhesion molecules. In the cardiovascular system, these signals promote endothelial dysfunction and atherosclerotic lesion formation and increase monocyte and macrophage infiltration. In the metabolic system, elevated IL‐6 and CRP impair PI3K/AKT signaling, reduce glucose uptake by muscle and adipose tissue, induce insulin resistance, and exacerbate abnormalities in glucose and lipid metabolism. In the nervous system, chronic inflammation activates microglia and induces the release of inflammatory mediators such as TNF‐α and IL‐1β, thereby promoting neuronal injury and cognitive decline. In the immune system, persistently elevated IL‐6/CRP levels disrupt immune homeostasis, drive T cells toward inflammatory phenotypes such as Th17 cells, and suppress regulatory immune mechanisms, thereby increasing leukocyte chemotaxis and inflammatory cell infiltration. This inflammatory network not only amplifies localized inflammatory responses through molecular signaling but also affects multiple organ systems through cross‐organ communication, thus forming a vicious cycle of systemic inflammation that underlies chronic diseases and providing a scientific basis for early deviation detection, risk assessment, and individualized intervention [1144, 1145].

Elevated HbA1c levels within the metabolic network reflect chronic disruption of glucose homeostasis. Sustained hyperglycemia is largely a result of reduced insulin sensitivity in target tissues such as muscle, adipose tissue and liver, resulting in decreased glucose uptake and increased hepatic gluconeogenesis. The resulting chronic hyperglycemia increases HbA1c levels, which not only indicates abnormal glycemia but also exacerbates tissue damage through multiple mechanisms. Hyperglycemia promotes mitochondrial ROS production, drives advanced glycation end‐product formation, and activates proinflammatory responses via NF‐κB signaling, thereby increasing the burden of chronic low‐grade systemic inflammation [1146].

The oxidative stress network, centered on ROS, represents a common pathological basis for multiple degenerative diseases and aging. ROS are primarily derived from leakage of the mitochondrial electron transport chain, activation of NADPH oxidase in inflammatory cells, and free fatty acids and glycometabolic byproducts generated during metabolic dysregulation. Excessive ROS can directly oxidize DNA, proteins, and lipids, leading to cellular damage, apoptosis, and a decline in mitochondrial function. In the nervous system, ROS induce neuronal mitochondrial injury, activate microglial inflammatory responses, and promote neuronal apoptosis, which constitute significant mechanisms in neurodegenerative disorders such as Alzheimer's disease and Parkinson's disease. In the cardiovascular system, ROS promote low‐density lipoprotein oxidation, endothelial injury, and vascular stiffening, thereby increasing the risk of atherosclerosis. In the liver and kidney, ROS accelerate lipid peroxidation and hepatocellular or renal cell injury, ultimately contributing to functional decline [1147].

### Expansion of emerging axes and frontier perspectives

Beyond classical pathways, emerging interorgan axes introduce new interpretive layers into biomarker networks. These axes highlight routes of information transfer and feedback regulation between different tissues such that biomarkers are no longer viewed merely as passive readouts of single‐organ states but rather as reflections of shared cross‐organ drivers and reciprocal interactions. Incorporating these axes into network analysis can help explain why synchronous abnormalities arise across multiple organs within the same time window while also facilitating the detection of early subclinical deviations because many axis‐related alterations precede overt symptoms and manifest as changes in metabolites, neuroendocrine signals, or immune tone. Moreover, an axis‐based perspective enhances the directionality of intervention strategies by shifting clinical management from single‐marker correction toward the identification of key transmission pathways and feedback nodes, thereby providing clearer mechanistic guidance for multitarget intervention, treatment evaluation, and warning of relapse.

#### Microbiota–organ axes

The gut microbiota can transmit local metabolic and immune messages from the intestine to peripheral circulation and distant organs through small molecules such as SCFAs, bile acid derivatives, and tryptophan metabolites, thereby establishing multiple axes, including the gut–brain, gut–liver, and gut–heart axes. For example, SCFAs such as butyrate can affect blood–brain barrier integrity and regulate central inflammation levels; therefore, their levels may serve as intuitive indicators for assessing the functional state of the gut–brain axis [97]. At the level of the gut–liver axis and systemic metabolism, the gut microbiota converts primary bile acids secreted by the liver into a variety of secondary bile acids via deconjugation, dehydroxylation, and other reactions, thereby remodeling the composition of circulating bile acids. Bile acids not only participate in lipid absorption but also act as signaling molecules that activate nuclear receptors such as FXR, thereby regulating the feedback of bile acid synthesis and enterohepatic circulation, while also influencing pathways involved in glucose metabolism, lipid metabolism, and immune inflammation. Accordingly, changes in secondary bile acid profiles and their associated receptor signaling may serve as important quantitative indicators of cross‐organ metabolic status and help to explain how alterations in intestinal ecology impact systemic metabolism [1148, 1149, 1150]. In addition, studies have shown that microbiota‐derived metabolites such as TMAO are linked to atherosclerotic risk, supporting the incorporation of microbial metabolic features into cardiovascular multimorbidity risk stratification and mechanistic interpretation [1151]. As these microbiome‐derived molecules are highly sensitive to dietary intake, microbial composition, and environmental changes, their levels may dynamically fluctuate with physiological state and therefore could serve as novel biomarkers reflecting axis function, providing complementary data for early multimorbidity diagnosis and intervention monitoring.

#### Mechanobiochemical coupling

Physical force signals can be converted to biochemical signals through mechanosensitive ion channels, thereby influencing cell function and biomarker expression. Hemodynamic shear stress is one of the most important mechanical stimuli acting on the vascular endothelium. Through mechanosensitive channels and membrane cytoskeleton‐associated transduction pathways, it triggers downstream signaling, promotes endothelial nitric oxide synthase activation, and increases nitric oxide production and release. Nitric oxide not only directly regulates vascular tone but also suppresses leukocyte adhesion and platelet aggregation at the endothelial level, maintaining an anti‐inflammatory and antithrombotic endothelial phenotype. As a result, changes in the hemodynamic environment are often associated with nitric oxide‐related pathways and vascular functional biomarkers [1152, 1153]. In barrier tissues such as the alveolar epithelium, mechanical stretching and microenvironmental perturbations can activate TRPV4 channels, which trigger calcium influx, alter intercellular junctions and barrier permeability, and generate phenotypes consistent with pulmonary edema or inflammatory amplification. Relevant studies further suggest that TRPV4‐associated calcium signaling can drive protease‐mediated shedding of membrane proteins, thereby compromising epithelial barrier integrity. Therefore, in disease settings where barrier disruption is a central mechanism, the TRPV4 pathway provides a testable mechanistic route linking mechanical stimulation to barrier‐relevant biomarkers [1154].

#### Neuroendocrine‐immune networks

The autonomic nervous system can directly regulate immune cell activation, migration and cytokine release through neurotransmitters such as norepinephrine and acetylcholine. Sympathetic signaling is largely mediated by β2‐adrenergic receptors and can alter the direction and magnitude of immune responses in different tissue microenvironments, thereby affecting the intensity and duration of inflammation. Stress also activates the HPA axis and disrupts cortisol circadian rhythms; such rhythmic perturbations can remodel the diurnal oscillations of peripheral immune cell populations. At the metabolic level, sympathetic outflow also promotes lipolysis in adipose tissue and regulates hepatic glucose production and output, thus influencing blood glucose and lipid homeostasis [1155, 1156, 1157].

#### Clinical integration and translational pathways

Translating biomarker‐network theory into clinical practice requires the development of a multilayered integrated strategy that includes detection, interpretation, intervention and follow‐up, as well as the transformation of network information into actionable clinical outputs. In real‐world clinical practice, biomarkers from different organs often fluctuate simultaneously. Such cofluctuation may reflect shared upstream drivers or alternatively indicate cascade effects after the initiation of cross‐system bridging processes. If interpreted in isolation, it is usually difficult to determine whether an abnormality represents a localized organ event or an external manifestation of systemic processes, let alone identify the major risk propagation pathway and which other organ systems are likely to be affected next. The network‐based perspective emphasizes the organization of systemic driving signals, cross‐system bridging signals and organ‐specific injury signals into interpretable combinations, allowing for clinicians to capture both the overall risk background and the direction of risk expansion during a single assessment, as well as the currently involved organs, thereby improving the clarity of multimorbidity subtyping and prognostic evaluation.

At the implementation level, a network framework can transform testing strategies from simple stacking of indicators to modular assessment. The systemic driver module reflects the inflammatory tone, metabolic burden and neuroendocrine stress intensity. The bridging module indicates whether key interfaces such as the endothelium, barrier function or immune cell trafficking are activated. The organ module localizes functional impairment or tissue damage in the heart, kidney, liver, brain and other target organs through effector nodes. On the basis of this structured combination, clinical interpretation can simultaneously provide conclusions at three levels: overall risk level, principal driving axis and vulnerable organs involved, and current degree of organ involvement together with complication risk. In addition, repeated measurements and trajectory analysis can determine whether the network status remains persistently deviated, shows accelerated upward shifts, and returns to individual baseline after intervention. This approach enables earlier recognition of disease turning points and heterogeneity in treatment response while avoiding over‐ or undertreatment based on single‐point measurements alone. Finally, this transition from static values to dynamic trajectories transforms biomarker networks from an interpretive tool into a management tool and provides a more reliable basis for staged intervention, efficacy monitoring and relapse warning.

#### Multidimensional biomarker combination models

In cross‐organ biomarker applications, single general biomarkers such as NfL are sensitive indicators of tissue damage but lack etiological specificity and therefore cannot be used alone for precise diagnosis. A network perspective integrates general and disease‐specific biomarkers into multidimensional combination models that combine early warning with etiological discrimination. Core hubs summarize the intensity of systemic driving forces and background signal amplification; regional hubs indicate whether cross‐system bridging processes have been mobilized; and effector nodes localize organ involvement and the extent of injury. In neurodegenerative diseases, the general biomarker NfL provides quantitative information on the burden of injury, whereas disease‐specific biomarkers such as Aβ42/40, p‐tau, and α‐synuclein provide qualitative etiological information. This combinatorial strategy enables more accurate distinction between disease types while also clarifying the role of hub biomarkers as “sentinels” for warning, with definitive diagnosis relying on joint assessment of downstream disease‐specific biomarkers. Compared with traditional single‐marker approaches, this layered combination strategy is more consistent with parallel fluctuation patterns commonly observed in clinical practice and facilitates the translation of test results into phenotypic and prognostic information by distinguishing, for example, whether risk is primarily driven by metabolic dysregulation or inflammatory stress and simultaneously identifying vulnerable organ systems potentially entering cascade processes. A two‐tier strategy comprising a baseline panel and targeted panels is more appropriate for clinical implementation. The baseline panel, intended for all individuals, enables initial screening and global risk stratification and captures common information on metabolic burden, inflammatory tone, and key organ function. Targeted panels are then added according to the patient's chief complaints and multimorbidity profile to improve resolution for priority organs and critical pathways. Model outputs should emphasize interpretability: in addition to providing an overall level of risk, they should indicate the directionality of the contribution of principal driving axes and the organ systems that they suggest may be affected. Robustness should be validated in external cohorts wherever possible so that network maps can ultimately be translated into clear and actionable clinical conclusions [1158].

#### Dynamic network monitoring strategies

Biomarker networks exhibit marked temporal variability, and a single measurement is often insufficient to distinguish transient fluctuations from persistent remodeling. A stratified dynamic monitoring strategy is therefore needed. In the short term, relatively dense repeat measurements may be performed around key windows, such as disease flares, treatment initiation, infection or other stress exposures, to determine whether hub signals are rising rapidly, whether regional bridging processes have been mobilized, and whether effector nodes show new organ dysfunction or shift from reversible fluctuation to persistent deviation. In the long term, trend assessment at fixed intervals should be used to track the accumulation of chronic burden and determine whether an individual's baseline drifts over time. Interpretation should not focus solely on whether values exceed thresholds; rather, rates of change, magnitudes of fluctuation, and speeds of recovery should be incorporated into the same framework because delayed recovery or failure to return to an individual's homeostatic range often indicates reduced network resilience and may warrant more frequent follow‐up or stronger intervention. Repeated measures data can also be used to construct time‐updated dynamic risk prediction models to support personalized reassessment scheduling.

At the operational level, dynamic monitoring should emphasize the comparability of measurement conditions and interpretability of trajectory‐based indices. Repetitive sampling should ideally be performed using the same analytical platform and under similar collection conditions, with priority given to reporting trajectory features that best reflect the state of the network. Clinical application studies also suggest that single‐point biomarkers measured at admission often have limited discriminative value for outcome stratification in acute critical illnesses, whereas biomarker trajectories over several consecutive days more effectively reveal progressively diverging physiological paths between survival and adverse outcomes. This provides direct evidence supporting short‐term intensive monitoring.

#### Selection of timing for individualized intervention

**FIGURE 9:**
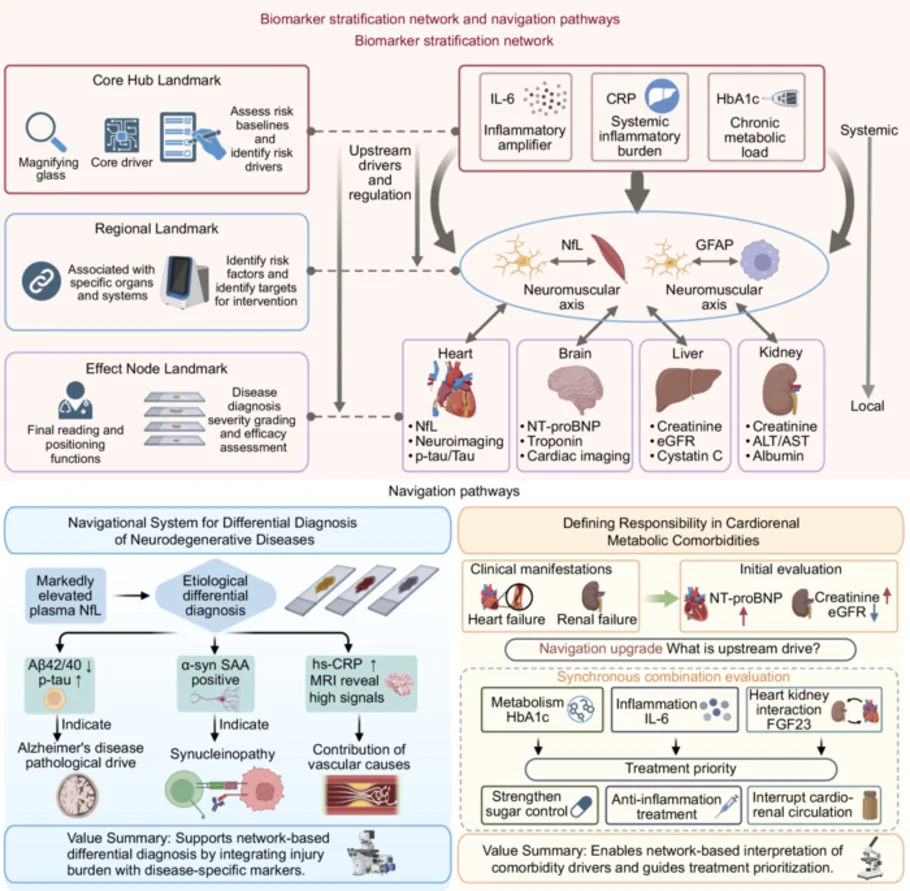
Biomarker stratification network and navigation pathways. This schematic illustrates a hierarchical biomarker framework for network‐based disease interpretation and clinical decision‐making. Biomarkers are stratified into three levels: core hub landmarks for assessing baseline risk and upstream drivers, regional landmarks for linking biomarkers to specific organs or systems, and effect node landmarks for diagnosis, severity grading and efficacy evaluation. The central network connects systemic markers, including IL‐6, CRP and HbA1c, with inflammatory amplification, systemic inflammatory burden and chronic metabolic load. These drivers interact with intermediate neuroimmune markers such as NfL and GFAP and extend to organ‐specific panels in the heart, brain, liver and kidney, including NT‐proBNP, troponin, p‐tau/tau, creatinine, eGFR, cystatin C, ALT/AST and albumin. The lower panels show two representative navigation pathways: differential diagnosis of neurodegenerative diseases based on NfL, Aβ42/40, p‐tau, α‐synuclein seed amplification, hs‐CRP and MRI findings; and interpretation of cardiorenal metabolic comorbidities using NT‐proBNP, creatinine/eGFR, HbA1c, IL‐6 and FGF23 to identify upstream drivers and guide treatment priorities.

The overall system state reflected by integrated biomarker patterns can provide a clearer stratified basis for choosing the timing of intervention. In the early stage, systemic hub signals are only mildly elevated, and organ injury or functional decline indicators have not yet become persistently abnormal. At this point, lifestyle modification and risk factor control should be the main focus of intervention, with an emphasis on reducing metabolic burden and inflammatory tone to prevent abnormal trends from becoming fixed and progressing downstream. During the progressive stage, hub signals remain persistently deviated, and cross‐system bridging markers gradually increase, indicating that risks have spread along critical pathways. At this stage, pharmacological treatment and organ protection strategies should be intensified as soon as possible, combined with comprehensive management of multiple risk factors, to reduce the likelihood of a systemic background state evolving into overt organ involvement. If the condition further develops into persistent or rapidly worsening abnormalities in multiple organ‐specific effector markers, it generally indicates that a systemic injury cascade has already formed. At this time, therapeutic priorities should shift toward coordinated multisystem support and comprehensive management while continuously monitoring whether systemic signals are declining and whether organ‐specific markers show recovery trends, thereby dynamically adjusting treatment intensity and resource allocation (Figure 9).

## CHALLENGES AND FUTURE PERSPECTIVES

The grand vision of constructing a “Human Biomarker Navigator” represents a crucial shift in medical research, transitioning from discrete phenomenological observation to the systematic regulation of biological networks. However, translating this blueprint into reality necessitates confronting profound challenges related to both the scientific paradigm and the health care ecosystem. This section aims to move beyond a mere list of technical bottlenecks, instead providing a macroscopic examination of the core constraints within the field and outlining a future trajectory toward “panoramic” precision health [1159].

### Core contemporary challenges

Current research and clinical practice paradigms reveal deep limitations when confronted with the extreme complexity of biomarker networks [1160]. These challenges are not isolated but are intricately intertwined, forming a cluster of problems that must be addressed systematically.

Technologically, we remain in the early stages, akin to “the blind men and the elephant.” First, existing detection technologies face inherent sensitivity and specificity ceilings. To detect very early‐stage lesions or trace amounts of circulating biomarkers, the current lower limits of detection are often insufficient to provide a reliable window for ultra‐early intervention [1161]. Second, powerful spatial heterogeneity is obscured by technological “averaging.” Bulk detection methods cannot resolve differences in biomarker expression among various cellular subpopulations within a tumor or the tissue microenvironment; therefore, a critical spatial context essential for understanding cell–cell communication and immune dynamics is lost. Finally, there is a critical lack of technologies for dynamic, continuous monitoring. Single‐time‐point “snapshot” measurements fail to capture the continuous dynamics of disease progression, limiting their value for real‐time efficacy assessment and early warning.

The expansion of multiomics data has resulted in unprecedented volume but also immense challenges in terms of integration and interpretation [1162]. First, data silos and a lack of standardization persist. Data generated from diverse platforms and laboratories, including genomic, proteomic, and metabolomic data, frequently lack unified quality control standards and formatting protocols. This situation results in the formation of isolated data islands, impeding cross‐study and cross‐center integration and validation. Second, a significant “gap” exists between correlation and causation. The vast number of biomarkers identified through high‐throughput screening are often correlated signals; their biological functions, regulatory pathways, and causal roles within disease networks remain largely unknown. A list of markers without functional validation has limited clinical translational value. Third, computational models suffer from “black box opacity” and poor generalizability. While AI algorithms can construct complex predictive models, their decision‐making processes are often non‐transparent, and their performance frequently deteriorates when they are applied to independent cohorts or diverse populations, hampering direct clinical application.

The path from discovery to clinical application is fraught with obstacles. The most significant barrier is the discontinuity in the evidence chain [1163]. Many promising biomarkers lack support from prospective, large‐scale, multicenter interventional clinical trials demonstrating that their use in guiding clinical decisions ultimately improves hard endpoints. Second, rigorous health economic scrutiny is paramount [1164]. The high cost of multiomics testing demands robust cost‐effectiveness analyses to prove its incremental value over standard markers before it is accepted by health care systems. Finally, clinical pathway inertia and physician cognition pose significant barriers to adoption. Integrating new biomarkers into clinical guidelines and altering deeply ingrained practice patterns is a slow and challenging process.

Beyond these well‐appreciated roadblocks, a critical bottleneck is analytical validity and standardization. Interplatform variability represents a major problem: the same biomarker measured by different technology platforms (mass spectrometry vs. SIMOA vs. ELISA) can yield very different absolute values, making it difficult to compare, let alone integrate, results across studies and clinical laboratories. As a result, reference intervals and universal cutoffs are lacking for most emerging biomarkers. Without well‐validated, widely accepted clinical decision thresholds, physicians cannot reliably interpret a given test result, severely limiting direct clinical application. Interassay and interlaboratory variability further compound this problem; even when the same platform is used, differences in reagents, calibration protocols, lot‐to‐lot variation, and operator expertise introduce significant noise. Well‐designed harmonization studies and proficiency testing programs are urgently needed. Notably, the current Human Biomarker Navigator framework, by systematically aggregating knowledge from diverse sources, could serve as a foundational platform to enable future standardization efforts and consensus cutoff derivation. However, it is important to recognize that the paucity of standardized protocols and validated decision thresholds remains a primary obstacle to translating the Navigator's theoretical network into routine clinical practice.

### Future directions

Addressing these challenges requires not only iterative technological improvements but also a comprehensive paradigm revolution encompassing concepts, technologies, models, and systems.

Future technological breakthroughs will focus on achieving continuous, spatial, and dynamic profiling of biological processes. First is the maturation and democratization of “spatiotemporal omics” [1165]. Technologies capable of resolving gene expression within the context of spatial architecture at single‐cell resolution will revolutionize our understanding of tumor heterogeneity and the tumor microenvironment, revealing the spatial organization of biomarkers. Second, the iteration of “liquid biopsy” technologies is needed [1166]. The focus will shift from detecting ctDNA mutations to comprehensive, highly sensitive, and quantitative analysis of exosomes, circulating RNAs, and fragmentation patterns, truly realizing its potential as a “liquid biopsy” for monitoring disease evolution. Third is the rise of “flexible wearable and implantable sensors” [1167]. These devices will enable noninvasive, continuous, long‐term dynamic monitoring of physiological parameters and specific molecules, providing unprecedented temporal data for early warning and personalized therapy.

Beyond these technological breakthroughs, three emerging directions are poised to reshape the field: multimodal biomarker integration, artificial intelligence‐driven discovery, and biomarker‐based patient stratification.

The key challenge is not just more data but meaningful fusion of molecular, imaging, digital, and pathology‐based biomarkers. For example, deep learning models that jointly analyze plasma proteomic profiles with magnetic resonance imaging radiomic features have shown superior performance in predicting early conversion to neurodegenerative disease. The development of cross‐modal alignment and representation learning frameworks will uncover systemic pathological associations invisible to any single modality, thereby enriching the Navigator graph with more informative nodes and edges.

Machine learning and deep learning excel at feature selection and dimensionality reduction in high‐dimensional omics data. Random forests, graph neural networks, and other algorithms can identify the core panels of biomarkers most relevant to disease progression. Furthermore, AI can be used to construct dynamic network models, such as training a hidden Markov model or recurrent neural network on longitudinal data, to simulate the probabilistic transition of biomarkers across disease stages. These computational tools accelerate the discovery of novel markers and provide an algorithmic backbone for the Navigator's predictive functions.

Future practice will shift from single‐marker cutoffs to multimarker risk scores and subtype classification. Unsupervised clustering or supervised learning can partition individuals into subgroups with distinct prognostic features or treatment response patterns based on their multiomics profiles. In oncology, for example, classifiers integrating driver mutations, immune microenvironment markers, and radiomic features have already enabled more precise identification of patients who are likely to benefit from specific immunotherapies. This stratification strategy is equally applicable in multimorbidity scenarios, facilitating both “same disease, different treatments” and “different diseases, shared treatments” within the precision medicine framework.

The ultimate goal of the Navigator is to catalyze a fundamental shift in the medical paradigm. First, we define “primordial prevention.” By prospectively mapping the multiomics landscape of high‐risk individuals, it will be possible to identify early perturbations in disease networks years or even decades before clinical symptoms appear, enabling genuine primordial prevention. Second, “personalized dynamic navigation” should be achieved [1168]. Integrating continuous monitoring data with AI models will facilitate the creation of “digital twins” for individuals, dynamically simulating disease trajectories and predicting responses to various interventions, thereby enabling real‐time optimization of treatment strategies. Third, “disease taxonomy” should be redefined [1169]. Future disease classification will move beyond organ‐based and histopathological criteria toward a system based on shared molecular pathways and biomarker networks (“biotypes”), guiding drug repurposing and novel drug development across traditional disease boundaries.

Achieving this grand vision relies on a supportive ecosystem of collaborative innovation. A priority is the initiation of an “International Biomarker Network Consortium” [1170]. Like the Human Genome Project, this requires top global research institutions, hospitals, and industry partners to establish standards, share data and samples, and conduct very large‐scale prospective cohort studies. Concurrently, building a “Biomedical Data Highway” is essential [1171]. This involves creating secure, compliant, and interoperable national and global data platforms that integrate multiomics, EHR, imaging, and real‐time monitoring data to fuel AI‐driven discovery. Critically, cultivating a new generation of “clinical–computational biologists” is paramount. These individuals, who are deeply versed in both clinical needs and data science, will be key to bridging the “last mile” from algorithm to bedside application.

## CONCLUSION

Biomarkers are pivotal in the realm of human health and act as crucial conduits for objectively gauging biological processes and establishing connections between microscopic mechanisms and macroscopic health outcomes. By encapsulating multidimensional data at the genomic, proteomic, and metabolomic levels, biomarkers can sensitively detect underlying organ damage or systemic compensatory states before clinical symptoms appear. This provides a scientific foundation for early disease detection, accurate diagnosis, personalized treatment options, and dynamic therapeutic oversight. In today's public health landscape, which is characterized by a high incidence of chronic diseases and widespread comorbidities, biomarkers form the bedrock for shifting from disease treatment to prevention, facilitating comprehensive health management throughout an individual's life cycle. Current research and clinical application of biomarkers face profound challenges and bottlenecks. First, most studies are confined to single diseases or organs and lack an integrated perspective across systems and diseases that could address the reality of the human body as a complex system. Second, many biomarkers remain at the level of mechanistic associations, demonstrating insufficient generalizability in complex real‐world scenarios such as those involving comorbidities and aging. Furthermore, numerous promising biomarkers struggle to transition from laboratory discovery to routine clinical practice because of the absence of standardized detection methods, unified thresholds, and multicenter validation. These collective bottlenecks result in fragmented knowledge of existing biomarkers, preventing the formation of a dynamic navigation system for guiding health management. In this review, a panoramic biomarker atlas spanning nine major human physiological systems is constructed, and the evolutionary trajectories of various biomarkers along the continuum from health to disease are systematically elucidated. More importantly, it reveals intersystem, interorgan, and interdisease interaction networks centered on key hub biomarkers, providing molecular‐level insights into the shared pathological foundations of comorbidities. The main goal of this work is to achieve a transition from static knowledge enumeration to a dynamic systems approach, thereby contributing to the theoretical basis for establishing a predictive and individualized health management framework. Additionally, the newly developed Human Biomarker Navigator website (http://www.hbiomarker.com) will serve as a practical platform for biomarker discovery across diseases and the construction of interaction networks.

## AUTHOR CONTRIBUTIONS

**Meng‐Yao Li:** Conceptualization; writing—original draft; writing—review and editing. **Qian Zhang:** Conceptualization; writing—original draft; visualization; writing—review and editing. **Zijie Wang:** Conceptualization; writing—original draft; visualization; writing—review and editing. **Zeping Gui:** Conceptualization; writing—original draft; visualization; writing—review and editing. **Jing Liu:** Conceptualization; writing—original draft; visualization; writing—review and editing. **Jingda Li:** Conceptualization; writing—original draft; visualization; writing—review and editing. **Maojun Jiang:** Conceptualization; visualization; writing—review and editing. **Zukang Chang:** Writing—original draft; visualization; writing—review and editing. **Qingqing Han:** Writing—original draft; writing—review and editing. **Bendong Yang:** Writing—original draft; writing—review and editing. **Tariq Saeed:** Writing—review and editing. **Jun Li:** Writing—original draft; writing—review and editing. **Jinghan Jia:** Writing—original draft; visualization. **Chenghu Song:** Writing—original draft; writing—review and editing. **Tong Shao:** Writing—review and editing. **Tianhuan Peng:** Writing—review and editing. **Xueying Zhang:** Writing—original draft; visualization. **Pingping Guo:** Conceptualization; writing—original draft; visualization. **Jun Luo:** Conceptualization; funding acquisition; visualization. **Gangao Yang:** Writing—original draft; visualization. **Yuan Wan:** Writing—review and editing. **Gokhan Zengin:** Conceptualization; writing—review and editing. **Quan Yuan:** Conceptualization; writing—review and editing. **Lingru Li:** Conceptualization; writing—review and editing; writing—original draft. **Xiaohui Fan:** Conceptualization; writing—original draft; writing—review and editing. **Yang Luo:** Conceptualization; writing—original draft; writing—review and editing. **Lvyun Zhu:** Conceptualization; writing—review and editing. **Yanfei Zheng:** Conceptualization; writing—review and editing; writing—original draft. **Wenjun Mao:** Conceptualization; writing—original draft; funding acquisition; writing—review and editing. **Wenlong Sun:** Conceptualization; writing—original draft; funding acquisition; writing—review and editing.

## CONFLICT OF INTEREST STATEMENT

The authors declare no conflicts of interest.

## ETHICS STATEMENT

No new animal or human experiments were involved in this study.

## Supporting information


**Figure S1:** Comprehensive landscape of nervous system biomarkers.
**Figure S2:** Comprehensive landscape of cardiovascular system biomarkers.
**Figure S3:** A panoramic view of biomarkers in the human digestive system.
**Figure S4:** Schematic diagram of biomarkers related to the human endocrine and metabolic systems.
**Figure S5:** Panoramic view of urinary system biomarkers.
**Figure S6:** Panoramic view of respiratory biomarkers.
**Figure S7:** Panoramic view of biomarkers in the human locomotor system.
**Figure S8:** Panoramic view of biomarkers in the human reproductive system.
**Figure S9:** Blood and immune system biomarkers panorama.

**Table S1:** Summary of core biomarkers for Alzheimer's disease.
**Table S2:** Panorama of biomarkers in Parkinson's disease.
**Table S3:** Integrated biomarker profile for amyotrophic lateral sclerosis.
**Table S4:** Spectrum of biomarkers in frontotemporal dementia.
**Table S5:** Systematic analysis of biomarkers in depression.
**Table S6:** Multidimensional biomarkers in schizophrenia.
**Table S7:** Compendium of molecular markers in glioblastoma.
**Table S8:** Overview of genetic and molecular markers in meningioma.
**Table S9:** Systemic overview of biomarkers in multiple sclerosis.
**Table S10:** Classification of biomarkers in epilepsy.
**Table S11:** Neurological system cross‐disease biomarkers.
**Table S12:** Panorama of biomarkers in coronary heart disease.
**Table S13:** Integrated and emerging biomarkers in heart failure.
**Table S14:** Systematic biomarkers in hypertension.
**Table S15:** Clinical classification of biomarkers in myocarditis.
**Table S16:** Overview of molecular and immune markers in angiosarcoma.
**Table S17:** Biomarkers across cardiovascular diseases.
**Table S18:** Cross‐system biomarkers related to the cardiovascular system.
**Table S19:** Summary of biomarkers for non‐alcoholic fatty liver disease.
**Table S20:** Summary of biomarkers for viral hepatitis.
**Table S21:** Summary of biomarkers for liver cirrhosis.
**Table S22:** Summary of biomarkers for inflammatory bowel disease.
**Table S23:** Summary of biomarkers for pancreatitis.
**Table S24:** Summary of biomarkers for esophageal carcinoma.
**Table S25:** Summary of biomarkers for gastric cancer.
**Table S26:** Summary of biomarkers for colorectal cancer.
**Table S27:** Summary of biomarkers for hepatocellular carcinoma.
**Table S28:** Summary of biomarkers for pancreatic cancer.
**Table S29:** Summary of biomarkers for cholangiocarcinoma.
**Table S30:** Biomarkers across digestive system diseases.
**Table S31:** Cross‐system biomarkers related to the digestive system.
**Table S32:** Systematic analysis of biomarkers in diabetes.
**Table S33:** Systematic analysis of biomarkers in thyroid dysfunction.
**Table S34:** Systematic analysis of biomarkers in metabolic syndrome.
**Table S35:** Systematic analysis of biomarkers in thyroid cancer.
**Table S36:** Systematic analysis of biomarkers in pituitary adenomas.
**Table S37:** Systematic analysis of biomarkers in adrenocortical carcinoma.
**Table S38:** Systematic analysis of biomarkers in pancreatic neuroendocrine tumors.
**Table S39:** Biomarkers across diseases of the endocrine and metabolic systems.
**Table S40:** Cross‐system biomarkers related to the endocrine and metabolic systems.
**Table S41:** Summary of biomarkers in chronic kidney disease.
**Table S42:** Classification and evaluation of biomarkers in acute kidney injury.
**Table S43:** Summary of biomarkers in glomerulonephritis.
**Table S44:** Classification and clinical application of biomarkers in renal cell carcinoma.
**Table S45:** Comprehensive table of biomarkers in bladder cancer.
**Table S46:** Biomarkers across diseases of the urinary system.
**Table S47:** Cross‐system biomarkers related to the urinary system.
**Table S48:** Classification and evaluation of biomarkers in pneumonia.
**Table S49:** Diagnostic and monitoring biomarkers in tuberculosis.
**Table S50:** Comprehensive table of biomarkers in chronic obstructive pulmonary disease.
**Table S51:** Classification and clinical application of biomarkers in asthma.
**Table S52:** Clinical application of biomarkers in non‐small cell lung cancer.
**Table S53:** Diagnostic and prognostic biomarkers in small cell lung cancer.
**Table S54:** Summary of biomarkers in pleural mesothelioma.
**Table S55:** Biomarkers across respiratory diseases.
**Table S56:** Cross‐system biomarkers related to the respiratory system.
**Table S57:** Systematic classification and clinical application of biomarkers in osteoporosis.
**Table S58:** Diagnostic subtyping and treatment prediction biomarkers in rheumatoid arthritis.
**Table S59:** Classification and evaluation of biomarkers in osteoarthritis.
**Table S60:** Diagnostic and resistance prediction biomarkers in osteosarcoma.
**Table S61:** Comprehensive table of biomarkers in multiple myeloma.
**Table S62:** Biomarkers of the musculoskeletal system across diseases.
**Table S63:** Cross‐system biomarkers related to the motor system.
**Table S64:** Endocrine and metabolic evaluation biomarkers in polycystic ovary syndrome.
**Table S65:** Non‐invasive diagnostic and pain assessment biomarkers in endometriosis.
**Table S66:** Classification and evaluation of biomarkers in benign prostatic hyperplasia.
**Table S67:** Early diagnosis and targeted therapy guidance biomarkers in ovarian cancer.
**Table S68:** Screening, diagnostic, and immunotherapy predictive biomarkers in cervical cancer.
**Table S69:** Molecular subtyping and prognostic evaluation biomarkers in endometrial cancer.
**Table S70:** Comprehensive table of molecular subtyping and therapeutic biomarkers in breast cancer.
**Table S71:** Diagnostic and therapeutic guidance biomarkers in prostate cancer.
**Table S72:** Diagnostic and prognostic stratification biomarkers in testicular cancer.
**Table S73:** Biomarkers of reproductive system across diseases.
**Table S74:** Cross‐system biomarkers related to the reproductive system.
**Table S75:** Detection and application of biomarkers in anemia.
**Table S76:** Diagnostic and monitoring biomarkers in autoimmune diseases.
**Table S77:** Comprehensive evaluation of biomarkers in acute myeloid leukemia.
**Table S78:** Diagnostic and prognostic evaluation of biomarkers in chronic myeloid leukemia.
**Table S79:** Classification and clinical application of biomarkers in hodgkin lymphoma.
**Table S80:** Comprehensive overview of biomarkers in non‐hodgkin lymphoma.
**Table S81:** Biomarkers of blood and immune systems across diseases.
**Table S82:** Cross‐system biomarkers related to the blood and immune system.

## Data Availability

Data sharing is not applicable to this article as no new data were created or analyzed in this study. “Human Biomarker Navigator” web platform may be found in http://www.hbiomarker.com. Supplementary materials (tables, graphical abstract, slides, videos, Chinese translated version, and update materials) may be found in the online DOI or iMeta Science http://www.imeta.science/.
