# Supplementary material for: Human biomarker navigator
Source: Imeta. 2026 Aug 20;5(5):e70157. doi: 10.1002/imt2.70157 (PMC13494139; doi:10.1002/imt2.70157)
Supplement: Supplementary file 1 [file IMT2-5-e70157-s002.docx]

**Human biomarker navigator**

**Running title:** Human biomarker navigator

Meng-Yao Li^1,2#^, Qian Zhang^3#^, Zijie Wang^4#,^ Zeping Gui^4#^, Jing Liu^3#^, Jingda Li^5#^, Maojun Jiang^1#^, Zukang Chang^1,6#^, Qingqing Han^1^, Bendong Yang^1^, Tariq Saeed^7^, Jun Li^3^, Jinghan Jia^6^, Chenghu Song^8^, Tong Shao^9^, Tianhuan Peng^10^, Xueying Zhang^3^, Pingping Guo^6^, Jun Luo^3^, Gangao Yang^6^, Yuan Wan^11^, Gokhan Zengin^12^, Quan Yuan^13*^, Lingru Li^1,14*^, Xiaohui Fan^15*^, Yang Luo^16*^, Lvyun Zhu^9*^, Yanfei Zheng^1,13*^, Wenjun Mao^8*^, Wenlong Sun^1,6*^

^1^Innovation Institute of Integrated Traditional Chinese and Western Medicine, Shandong First Medical University & Shandong Academy of Medical Sciences, Jinan 250117, China

^2^Renji Hospital Affiliated to Shanghai Jiao Tong University School of Medicine, Shanghai 200017, China

^3^College of Pharmacy, Inner Mongolia Medical University, Hohhot 010110, China

^4^Department of Urology, Jiangsu Key Laboratory of Urological Disease Prevention and Treatment, The Second Affiliated Hospital of Nanjing Medical University, Nanjing Medical University, Nanjing 210011, China

^5^School of Life Sciences, Yangtze University, Jingzhou 434023, China

^6^School of Life Sciences and Medicine, Shandong University of Technology, Zibo 255000, China

^7^Department of Diet and Nutritional Sciences, Ibadat International University, Islamabad 45750, Pakistan

^8^Department of Thoracic Surgery, the Affiliated Wuxi People's Hospital of Nanjing Medical University, Wuxi People's Hospital, Wuxi Medical Center, Nanjing Medical University, Wuxi 214023, China.

^9^College of Science, National University of Defense Technology, Changsha 410073, China

^10^Molecular Science and Biomedicine Laboratory (MBL), State Key Laboratory of Chemo and Biosensing, College of Biology, Hunan University, Changsha 410082, China

^11^The Pq Laboratory of BiomeDx/Rx, Department of Biomedical Engineering, Binghamton University, Binghamton 13850, USA

^12^Department of Biology, Science Faculty, Selcuk University, Konya 42130, Turkey

^13^College of Chemistry and Molecular Sciences, Wuhan University, Wuhan 430072, China

^14^National Institute of TCM Constitution and Preventive Medicine, Beijing University of Chinese Medicine, Beijing 100029, China

^15^State Key Laboratory of Chinese Medicine Modernization, College of Pharmaceutical Sciences, Zhejiang University, Hangzhou, 310058, China; School of Pharmaceutical Sciences, Zhejiang Chinese Medical University, Hangzhou 310053, China

^16^Department of Laboratory Medicine, Chongqing Center for Clinical Laboratory, Chongqing Academy of Medical Sciences, Chongqing General Hospital, School of Medicine, Chongqing University, Chongqing 400030, China

^#^These authors contributed equally: Mengyao Li, Qian Zhang, Zijie Wang, Zeping Gui, Jing Liu, Jingda Li, Maojun Jiang and Zukang Chang.

^*^Correspondence: Quan Yuan [(yuanquan@whu.edu.cn),](mailto:(yuanquan@whu.edu.cn),) Lingru Li (lilingru912@163.com), Xiaohui Fan (fanxh@zju.edu.cn), Yang Luo (Luoy@cqu.edu.cn), Lvyun Zhu (zhulvyun@nudt.edu.cn), Yanfei Zheng (yanfei_z@163.com), Wenjun Mao (maowenjun1@njmu.edu.cn). Wenlong Sun (Wenlongsun0814@163.com).

**Figure S1.** Comprehensive landscape of nervous system biomarkers. (A) Anatomical localization of major neurological and psychiatric diseases, including Alzheimer’s disease , Parkinson’s disease, frontotemporal dementia, amyotrophic lateral sclerosis, depression, schizophrenia, glioblastoma, meningioma, multiple sclerosis and epilepsy. (B) Timeline of nervous system biomarker development. Early biomarkers mainly relied on traditional pathology and imaging, such as Aβ plaques, neurofibrillary tangles and brain atrophy, which were invasive, costly and difficult to apply for early diagnosis or large-scale screening. With the development of multi-omics technologies, cerebrospinal fluid, blood and genetic biomarkers emerged, including Aβ42/40, total tau, phosphorylated tau, plasma Aβ, NfL, GFAP, *APOE* ε4 and *APP/PSEN1* methylation. Recent progress emphasizes omics integration and clinical translation, including composite biomarker models, blood test kits and dynamic NfL monitoring. (C) Representative biomarker matrix for neurological diseases. Diagnostic/screening and prognostic/monitoring biomarkers are summarized for AD, PD, ALS, FTD, depression, SCZ, GBM, meningioma, MS and epilepsy, including Aβ42/40 ratio, p-tau217, α-synuclein seeds, plasma NfL, GFAP, *IDH1/2* mutations, *MGMT* promoter methylation, CSF oligoclonal bands, MOG-IgG, LGI1 antibody and EEG spike-and-wave complexes. (D) Summary of current challenges and future trends in nervous system biomarker research.

**Figure S2.** Comprehensive landscape of cardiovascular system biomarkers. (A) Schematic distribution of major cardiovascular organs and representative diseases, including hypertension, cardiac angiosarcoma, heart failure, myocarditis, coronary artery disease and myocardial infarction. (B) Timeline of cardiovascular biomarker development. The field has progressed from traditional enzymology and imaging approaches, such as electrocardiography, echocardiography and enzyme activity assays, with classic biomarkers including CK-MB and myoglobin, to a multi-omics-driven exploration stage involving high-sensitivity cardiac troponins, proteomics and metabolomics. More recent advances emphasize artificial intelligence, composite models, wearable devices and liquid biopsy technologies, with emerging biomarkers such as *KLRD1*, *FOSL2*, BNP/NT-proBNP and ctDNA. (C) Representative biomarker matrix for major cardiovascular diseases. Diagnostic/screening and prognostic/monitoring biomarkers are summarized across coronary disease, heart failure, hypertension, myocarditis and angiosarcoma. (D) Current challenges and future trends in cardiovascular biomarker research.

**Figure S3.** A panoramic view of biomarkers in the human digestive system**.** (A) Anatomical distribution of major digestive organs and representative diseases. The schematic highlights key disease locations, including metabolic dysfunction-associated steatotic liver disease/metabolic dysfunction-associated steatohepatitis, liver cirrhosis, hepatocellular carcinoma, viral hepatitis, cholangiocarcinoma, esophageal cancer, gastric cancer, pancreatitis, pancreatic ductal adenocarcinoma, inflammatory bowel disease and colorectal cancer. (B) Evolution of digestive system biomarkers driven by technological advances. Biomarker discovery has progressed from traditional biochemical and pathological approaches, such as liver function testing, serum tumor marker detection and endoscopic biopsy, to molecular biomarker exploration involving proteins, nucleic acids and microorganisms, including AFP, GP73, ctDNA, *KRAS*, *IDH1/2*, miR-21, *Fusobacterium nucleatum* and *Helicobacter pylori*. Recent developments emphasize integrated models, liquid biopsy, minimal residual disease monitoring and microbiome-based intervention strategies, including GALAD score, FIB-4/NAFLD fibrosis score, ctDNA detection, microbial transplantation and probiotic interventions. (C) Core biomarker matrix for diagnosis, prognosis and monitoring across digestive diseases. Diagnostic/screening and prognostic/monitoring biomarkers are summarized for MAFLD/liver cirrhosis, HCC, esophageal cancer, gastric cancer, pancreatic cancer, CCA, CRC, IBD, viral hepatitis and pancreatitis. (D) Current challenges and future trends in digestive system biomarker research.

**Figure S4.** Schematic diagram of biomarkers related to the human endocrine and metabolic systems. (A) Anatomical distribution and disease localization of major endocrine and metabolic disorders, including metabolic syndrome, diabetes mellitus, thyroid dysfunction, thyroid carcinoma, pituitary adenoma, adrenal cortical carcinoma and pancreatic neuroendocrine neoplasms. (B) Evolution of endocrine and metabolic biomarkers driven by technological advances. Early biomarker discovery mainly relied on traditional pathology, imaging and standardized biochemical or hormonal assays, including glucose, HbA1c, TSH, FT3/FT4, PRL, GH, ACTH, cortisol and DHEAS. With the development of multi-omics technologies, autoantibodies, driver mutations and metabolic molecules, such as GADA, TPO-Ab, TRAb, *TP53*, *MEN1*, branched-chain amino acids and ceramides, have been increasingly explored. Recent progress emphasizes omics integration and clinical translation, including exosomal miRNAs, circRNAs, ctDNA, NETest, molecular grading and multi-biomarker models. (C) Core biomarker matrix for diagnosis, prognosis and monitoring across endocrine and metabolic diseases. Representative biomarkers are summarized for DM, thyroid dysfunction, TC, PA, ACC, MetS and pNENs, covering diagnostic markers, prognostic indicators and monitoring-related biomarkers such as HbA1c, C-peptide, NGAL, miRNAs, Ki-67, *TERT* promoter mutation, *AIP*, *MEG3*, β-catenin, adiponectin, hs-CRP, MDA, DAXX/ATRX and SSTR2A. (D) Current challenges and future trends in endocrine and metabolic systems.

**Figure S5.** Panoramic view of urinary system biomarkers. (A) Anatomical distribution of major urinary system organs and representative diseases, including glomerulonephritis, acute kidney injury, chronic kidney disease, renal cell carcinoma and bladder cancer. Enlarged schematic views highlight kidney, glomerular, nephron and bladder lesions associated with biomarker discovery. (B) Timeline of urinary biomarker development. Early biomarkers mainly relied on biochemical analysis, urinary microscopy and cytology, including Scr, BUN, urinary protein quantification and urinary cytology. Subsequent advances introduced immunoassay-based markers such as NGAL, KIM-1, NMP22, TIMP-2 × IGFBP7 and BTA, supported by ELISA and immunoturbidimetry. More recent technologies, including high-throughput sequencing, mass spectrometry, liquid biopsy and AI-based radiomics, have enabled the discovery of emerging biomarkers such as 5-MTP, urinary exosomal miRNAs, ctDNA in RCC and urinary circRNAs in bladder cancer. (C) Representative biomarker matrix for urinary system diseases. Diagnostic and prognostic biomarkers are summarized for CKD, AKI, bladder cancer, RCC and glomerulonephritis, including urinary albumin, cystatin C, KIM-1, NGAL, u*TERT*, NMP22, CA-IX, PAX8, u-sCD163, uNAG, uromodulin, galectin-3, CCL14, DKK3, CTC count, PD-L1, Ki-67, NLR, PLA2R-IgG4 and sIL-2Rα. (D) Current challenges and future trends.

**Figure S6.** Panoramic view of respiratory biomarkers. (A) Anatomical distribution of representative benign and malignant respiratory diseases. Benign pulmonary diseases include asthma, tuberculosis, chronic obstructive pulmonary disease and pneumonia , while malignant diseases include small-cell lung cancer, non-small-cell lung cancer and pleural mesothelioma. (B) Timeline of respiratory biomarker development. Early diagnosis mainly relied on traditional imaging, pulmonary function testing and inflammatory markers, including chest X-ray/CT, FEV1/FVC, CRP and sputum smear or culture. The molecular biomarker exploration stage introduced inflammation- and immunity-related markers, such as FeNO, sputum eosinophils and serum IgE, as well as tumor markers including NSE, ProGRP, CEA and CYFRA21-1. Recent advances emphasize multi-omics and precision medicine, involving *EGFR/ALK/ROS1* mutations, PD-L1, TMB, ctDNA, exosomes, circulating tumor cells, transcriptomic and metabolomic signatures, high-throughput sequencing, liquid biopsy and AI-assisted diagnosis. (C) Representative biomarker matrix for major respiratory diseases. Diagnostic and predictive biomarkers are summarized for COPD, asthma, pneumonia, NSCLC and PM, including pulmonary function, CRP, eosinophils, imaging-based emphysema assessment, FeNO, serum IgE, periostin, PCT, Pro-ADM, ctDNA, PD-L1, SMRP, fibulin-3, OPN and VEGF. (D) Current challenges and future development trends.

**Figure S7.** Panoramic view of biomarkers in the human locomotor system. (A) Anatomical overview of the locomotor system and representative diseases involving muscle, bone, cartilage and joints. The schematic highlights major skeletal and joint-related disorders, including osteoporosis, osteosarcoma, multiple myeloma, rheumatoid arthritis and osteoarthritis. (B) Development timeline of locomotor system biomarkers. Early biomarker discovery mainly relied on traditional pathology, serological testing and imaging approaches, including osteocalcin, alkaline phosphatase, PINP, β-CTX, X-ray and bone mineral density assessment. With the development of multi-omics technologies, proteomic, transcriptomic, lipidomic and imaging-omics biomarkers have emerged, such as OPG, DKK-1, miR-133a, S1P and MRI-PDFF. Recent progress emphasizes multi-omics integration and clinical translation, including combined diagnostic panels and dynamic monitoring systems such as ctDNA clonal evolution. (C) Representative biomarker matrix for locomotor system diseases. Diagnostic, prognostic and monitoring biomarkers are summarized for osteoporosis, rheumatoid arthritis, osteoarthritis, osteosarcoma and multiple myeloma. Representative biomarkers include PINP, β-CTX, sclerostin, RF, anti-CCP, anti-MCV, urinary CTX-II, serum COMP, ALP, LDH, SAA, β2-MG, FLC, sBCMA, *TP53* mutation, *RB1* deletion, ctDNA, MRD, M protein and MRI cartilage volume. (D) Current challenges and future trends of the locomotor system.

**Figure S8.** Panoramic view of biomarkers in the human reproductive system. (A) Anatomical distribution of representative reproductive system diseases. The schematic highlights breast cancer, polycystic ovary syndrome, endometriosis, ovarian cancer, endometrial cancer, cervical cancer, prostate cancer and testicular germ cell tumors. (B) Development timeline of reproductive system biomarkers. Early biomarker discovery mainly relied on traditional hormone and serum markers, including testosterone, LH/FSH, AMH, CA-125 and PSA, which were broadly used but limited by low specificity, low sensitivity and susceptibility to interference. With the development of omics technologies, genomics, metabolomics and proteomics have enabled the identification of molecular biomarkers, including HPV DNA/RNA, *BRCA1/2* mutations, ctDNA, miR-146a, miR-155, miR-371a-3p, HE4, inflammatory factors and metabolic fingerprints. Recent advances emphasize multi-omics integration and clinical translation, involving AI algorithms, organoid models, liquid biopsy and spatial omics, supporting diagnostic models, molecular classification and treatment guidance. (C) Representative biomarker matrix for reproductive system diseases. Diagnostic, prognostic/predictive and monitoring biomarkers are summarized for PCOS, endometriosis, ovarian cancer, cervical cancer, breast cancer, prostate cancer and TGCT. Representative biomarkers include AMH, testosterone, CA-125, HE4, miRNAs, HPV DNA/RNA, p16, ER/PR/HER2, Ki-67, PSA, PHI, *PCA3*, AFP, β-hCG, LDH, *BRCA1/2*, HRD, PD-L1, TMB, *AR*-V7, *GSTP1*, *KIT* and ctDNA. (D) Current challenges and future trends of the reproductive system.

**Figure S9.** Blood and immune system biomarkers panorama. (A) Anatomical distribution of major blood and immune organs, cells and representative diseases. The schematic highlights lymph glands, peripheral blood, immune cells, thymus, spleen, bone marrow, erythrocytes, leukocytes and lymphocytes, as well as related disorders including anemia, acute myeloid leukemia, chronic myeloid leukemia, Hodgkin lymphoma, non-Hodgkin lymphoma and autoimmune diseases. (B) Timeline of biomarker development in the blood and immune system. Early biomarker discovery mainly relied on traditional morphology and cytogenetics, including blood smear, bone marrow smear, karyotype analysis, serum immunoglobulins, complements and autoantibodies. With the development of molecular and omics technologies, flow cytometry, FISH and sequencing enabled the identification of fusion genes, gene mutations, immunophenotypes and disease-specific autoantibodies, such as *BCR-ABL*, *PML-RARA*, *NPM1*, *FLT3*-ITD, *JAK2*, CD-series markers, anti-dsDNA and anti-CCP. Recent advances emphasize multi-omics integration and precision medicine, including NGS, ctDNA-based liquid biopsy, single-cell sequencing, AI-assisted analysis, MRD monitoring, ELN risk stratification and targeted therapies against *FLT3*, *IDH1/2* and *BTK*. (C) Representative biomarker matrix for major hematologic and immune diseases. Diagnostic, prognostic/predictive and monitoring biomarkers are summarized for anemia, AML, CML, HL, NHL and systemic lupus erythematosus (SLE), including hemoglobin, ferritin, vitamin B12, NPM1 and FLT3 mutations, BCR-ABL fusion genes, CD30/CD15-positive HRS cells, ANA, anti-dsDNA, anti-Sm, MRD, *WT1*, ctDNA, PET-CT, C3/C4 and urine protein. (D) Current challenges and future trends.
